# Supplementary material for: Pillararene incorporated metal–organic frameworks for supramolecular recognition and selective separation
Source: Nat Commun. 2023 Aug 15;14:4927. doi: 10.1038/s41467-023-40594-2 (PMC10427641; doi:10.1038/s41467-023-40594-2)
Supplement: Supplementary file 1 — Supplementary Information [file 41467_2023_40594_MOESM1_ESM.pdf]

Supplementary Information

Pillararene incorporated metal−organic frameworks for supramolecular recognition and selective separation

Yitao Wu[†,1,2], Meiqi Tang[†,1], Zeju Wang[1,2], Le Shi[1,2], Zhangyi Xiong[1,2], Zhijie Chen*[,1,2], Jonathan L. Sessler*[,3] and Feihe Huang*[,1,2]

[1]Stoddart Institute of Molecular Science, Department of Chemistry, Zhejiang University, Hangzhou 310058, P. R. China.

[2]ZJU-Hangzhou Global Scientific and Technological Innovation Center-Hangzhou Zhijiang Silicone Chemicals Co., LTD Joint Lab, Zhejiang-Israel Joint Laboratory of Self-Assembling Functional Materials, ZJU-Hangzhou Global Scientific and Technological Innovation Center, Zhejiang University, Hangzhou 311215, P. R. China.

[3]Department of Chemistry, The University of Texas at Austin, Austin, Texas 78712-1224, United States.

[†]Yitao Wu and Meiqi Tang contribute equally to this work

* Corresponding authors: zhijiechen@zju.edu.cn; sessler@cm.utexas.edu; fhuang@zju.edu.cn

Table of Contents (123 pages)

1. Supplementary materials S3
2. Supplementary methods S3
3. Synthesis of pillar[5]arene struts S6
4. Synthesis of MOFs S10
5. ^{1}H NMR spectroscopic studies of MOFs S14
6. Single crystal X-ray data S21
7. Single crystal structure analysis S38
8. Powder X-ray diffraction patterns of MOFs S61
9. Guest binding studies of pillar[5]arene struts S65
10. Molecular recognition studies of MOFs S73
11. Molecular separation studies of MOFs S82
12. Supplementary references S123

1. Supplementary materials

All starting materials, including *N*,*N*-dimethylformamide (DMF), *N*,*N*-dimethylacetamide (DMA), 1,4-di(4-pyridyl)benzene (**PBPy**), 4,4'-(2,5-dimethoxy-1,4-phenylene)dipyridine (**MePBPy**), 4,4',4'',4'''-(ethene-1,1,2,2-tetrayl)tetrabenzoic acid (**H_4TPE**), 4',4''',4''''',4'''''''-(ethene-1,1,2,2-tetrayl)tetrakis(([1,1'-biphenyl]-4-carboxylic acid)) (**H_4TPPE**) and 1,2,4,5-tetracyanobenzene (**TCN**), were purchased commercially and used as received. Pyridine terminated pillar[5]arene-struts (**MeP5BPy** and **MeP5BPPy**), paraquat-hexafluorophosphate (**PQT**·PF_6), 1,4-dimethoxypillar[5]arene (**MeP5**) and 1,4-diethoxypillar[5]arene (**P5**) were synthesized as described previously [S1–S5].

2. Supplementary methods

2.1. Solution ^{1}H NMR spectroscopy

Proton nuclear magnetic resonance (^{1}H NMR) spectra were recorded using a Bruker Avance III DMX 400 spectrometer, a Bruker Avance III DMX 500 spectrometer, an Agilent DD2-600 spectrometer, or a JNM-ECZ500R/M1 instrument.

2.2. High performance liquid chromatography

The resolution of *racemic*-**MeP5** was performed by Daicel Chiral Technologies (China) Co., Ltd. on chiral a high performance liquid chromatography (HPLC) instrument equipped with a CHIRALPAK IC (IC00CD-NA012) preparative column using dichloromethane : methanol = 4 : 6 as the eluent. The column size was 0.46 cm I.D. × 15 cm L. The flow rate was 1.0 mL/min at 25 °C.

2.3. Single crystal growth

Single crystals of **MeP5BPPy** were grown by dissolving 5.00 mg of dry **MeP5BPPy** powder in chloroform, heating until all the powder was dissolved and allowing to evaporate about one week. Single crystals of (**Py**)$_2$@**P5** were grown by placing 5.00 mg of dry **P5** powder in a small vial, adding 1 mL of **Py**, heating until all the powder was dissolved, and allowing to evaporate at room temperature about one week. Single crystals of **Tol@MeP5-MOF-2** were obtained by immersing **MeP5-MOF-2** in a solution consisting of DMF and **Tol** (5:1 *v*/*v*) for a day.

General procedure for preparing single crystals of MOFs taking **MeP5-MOF-1** as an example: A DMF suspension (1.5 mL) of **MeP5BPy** (8.50 mg, 10.0 μmol), **H_4TPPE** (8.10 mg, 10.0 μmol), and $Zn(NO_3)_2 \cdot 6H_2O$ (5.97 mg, 20.0 μmol) was prepared in a small vial. This suspension was sonicated two minutes and then passed through a syringe filter to give a transparent solution, which was sealed, heated at a constant rate of 1 °C min^{-1} to 90 °C, kept at that temperature for 48 h and cooled to room temperature at a constant cooling rate of 0.2 °C min^{-1}. Transparent flaxen-colored single crystals of **MeP5-MOF-1** suitable for single crystal X-ray diffraction (SCXRD) were obtained and followed by immersion in 12 mL of acetone for 3 days, with the solvent topped off twice daily.

2.4. Single crystal X-ray diffraction analyses

SCXRD data were collected on a Bruker D8 VENTURE TXS PHOTON 100 diffractometer or a Bruker D8 VENTURE Metaljet PHOTON II diffractometer. Samples of single crystals were kept at 193 K or 262 K during the course of data collection. Structures were solved by an intrinsic phasing method and refined anisotropically with weighted full-matrix least squares on F^2 using the SHELXT[S6] and SHELXL[S7] programs with an Olex 2 graphic interface[S8].

2.5. Powder X-Ray diffraction

The powder X-ray diffraction (PXRD) data were collected on a Rigaku Ultimate-IV X-Ray diffractometer operating at 40 kV/30 mA using the Cu Kα line (λ = 1.5418 Å). Data were measured over the range of 3–30° in 13°/min steps over two minutes.

2.6. Fluorescence emission spectrascopy

Fluorescence emission spectra were collected on a RF-5301 spectrofluorophotometer (Shimadzu Corporation, Japan).

2.7. Optical photos

Optical photos of the single crystals were obtained using a SOPTOP ICX41M stereozoom microscope coupled to a digital camera and PC (video monitor).

2.8. Gas chromatography

Gas chromatographic (GC) analysis: GC measurements were carried out using an Agilent 7890B instrument configured with an FID detector and an HP-chiral *β* column (30 m × 0.32 mm × 0.25 μm). Samples were analyzed using headspace injections and performed by incubating the samples at 120 °C for 30 minutes followed by sampling 1 mL of the headspace. The total volume of the container is 10 mL; the mass of the solid in the container is about 10.0 mg; the total volume of the headspace is 1 mL. The following GC method was used: The oven was programmed from 60 °C, and ramped in 20 $°C·min^{-1}$ increments to 200 °C with a 5 minute hold; the total run time was 33 minutes; the injection temperature was 250 °C; the detector temperature was 280 °C with hydrogen, air, and make-up flow-rates of 35, 350, and 35 $mL·min^{-1}$, respectively; the helium (carrier gas) flow-rate was 3 $mL·min^{-1}$. The samples were injected using the split mode (30:1).

2.9. Theoretical calculation and simulation details

Structural model of **MeP5-MOF-1** was performed *via* geometry optimization. To predict the rotation ability of pillar[5]arene units in the MOF frameworks and the preferential locations of pyridine molecules within the pores, we carried out location simulations using the sorption module within Material Studio[S9]. The Metropolis Monte Carlo method was selected for the calculation of the global minimum location. The COMPASS force field was chosen for energy calculations, and the charge equilibration method was used to calculate the point atomic charges. The PXRD patterns were also predicted by the reflex module of Material Studio.

2.10. Gas sorption measurements

Supercritical CO_2 drying was performed using a SCD-350M (ShiAnjia Biotechnology Company) supercritical CO_2 dryer. CO_2 and N_2 adsorption/desorption isotherms for the activated materials were measured at 195 K and 77 K using a JW-BK200C 2 instrument.

2.11. Thermogravimetric analysis

Thermogravimetric analysis (TGA) analyses were carried out using a Q5000IR analyzer (TA Instruments) with an automated vertical overhead thermobalance. The heating range was from 25 °C to 600 °C. The samples were heated at 10 °C/min using N_2 as the protective gas.

2.12. Differential scanning calorimetry

Differential scanning calorimetry (DSC) was carried out using a DSC Q100 analyzer (TA Instruments). The samples were heated at 10 °C/min using N_2 as the protective gas.

3. Synthesis of pillar[5]arene struts

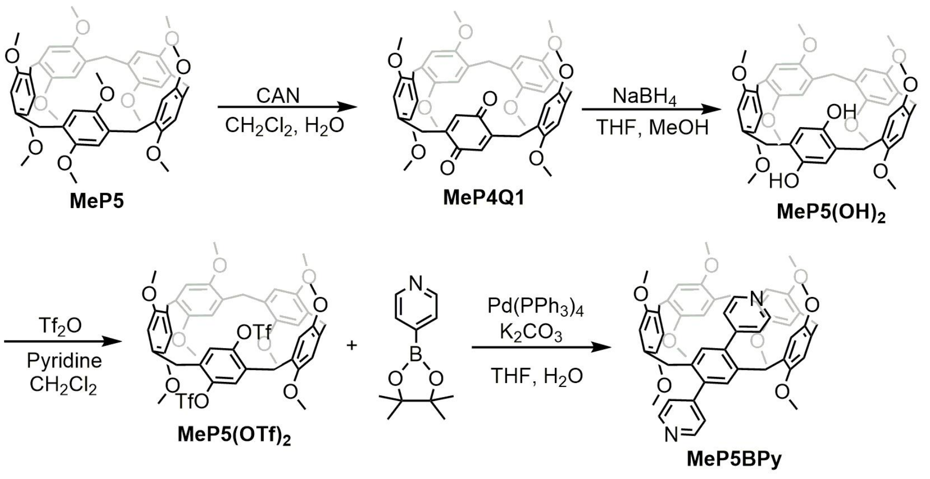

Supplementary Fig. 1 The synthetic route of **MeP5BPy**.

The starting material for the present syntheses was **MeP5**, which was prepared according to a previous report[S4].

MeP4Q1: **MeP5** (7.50 g, 10.0 mmol) was dissolved in 250 mL of dichloromethane (CH_2Cl_2). Cerium(IV) diammonium nitrate (CAN, 10.9 g, 20.0 mmol) in 20 mL of water was added drop by drop. The mixture was stirred at room temperature for 30 min. The reaction was quenched by adding 200 mL of water. The organic layer was separated, washed with water three times and then concentrated to obtain a dark red solid (6.80 g), which was found to contain **MeP4Q1** relatively free of multi-oxidized side products. The mixture was used in the next step without further purification. All impurities were easily eliminated in the third step.

MeP5(OH)$_2$: The dark red solid got in the previous step was dissolved in 200 mL of tetrahydrofuran (THF) and a solution of sodium borohydride ($NaBH_4$, 1.90 g, 50.0 mmol) in 200 mL of methanol was added. The mixture was stirred under an argon atmosphere at room temperature. The red solution turned light yellow almost

immediately. The reaction was quenched with water until no **MeP4Q1** was left as monitored by thin layer chromotography (TLC). The organic layer was separated and washed with water three times. After removal of the volatiles, a light yellow solid was obtained (5.23 g) that was used in the next step immediately and without further purification.

MeP5(OTf)₂: Trifluoromethanesulfonic anhydride (Tf_2O, 20 mL) was added dropwise to a mixture of **MeP5(OH)₂** (5.02 g, 6.90 mmol) and pyridine (dry, 10 mL) in CH_2Cl_2 (dry, 200 mL) at 0 °C under an argon atmosphere. The mixture was then allowed to stir at room temperature for 24 h before being quenched with water. After washing the organic phase with water three times, the orgainc layer was concentrated under vacuum and subjected to silca gel chromatography (1:1 petroleum ether/CH_2Cl_2) to give **MeP5(OTf)₂** as a white powder (5.31 g, 78%). The ^1H NMR spectrum of **MeP5(OTf)₂** is shown below. ^1H NMR (500 MHz, $CDCl_3$, 298 K) δ = 7.33 (s, 2H), 6.80 (s, 2H), 6.78 (s, 2H), 6.76 (s, 2H), 6.69 (s, 2H), 3.85–3.79 (m, 10H), 3.72 (s, 6H), 3.68 (s, 6H), 3.66 (s, 6H), 3.61 (s, 6H).

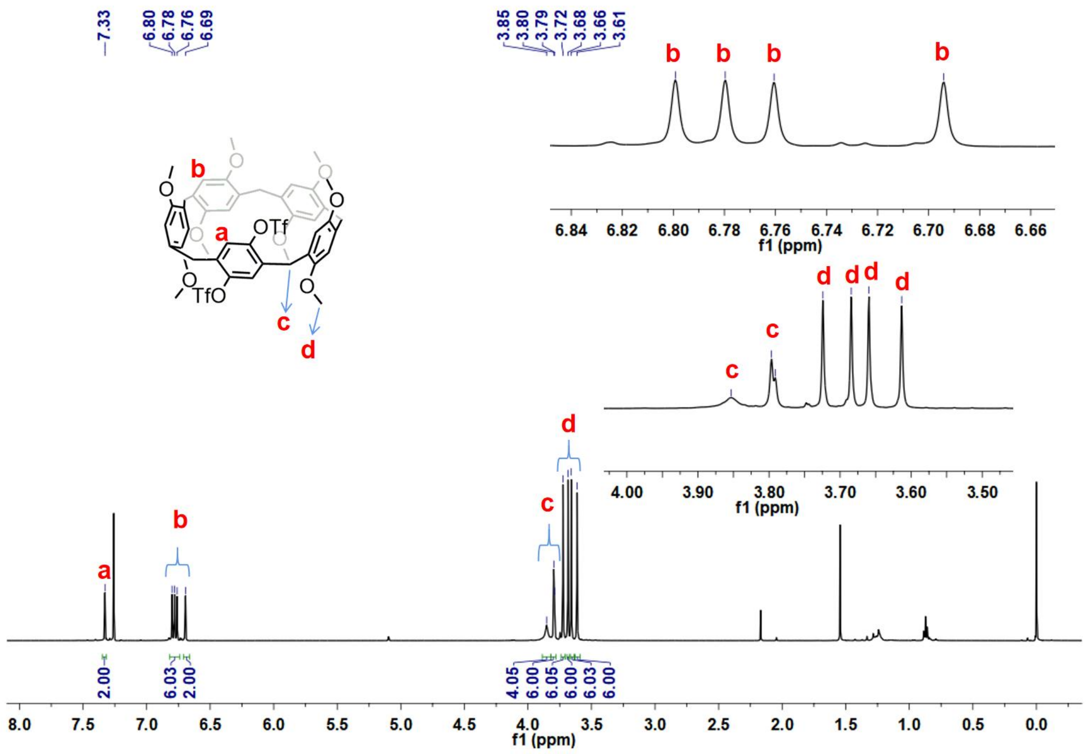

Supplementary Fig. 2 ^1H NMR (500 MHz, $CDCl_3$, 298 K) spectrum of **MeP5(OTf)₂**.

MeP5BPy: Tetrakis(triphenylphosphine)palladium [$Pd(PPh_3)_4$, 266 mg, 230 μmol] was added to a mixture of **MeP5(OTf)$_2$** (1.00 g, 1.00 mmol), 4-pyridineboronic acid pinacol ester (1.20 g, 6.00 mmol), and potassium carbonate (K_2CO_3, 2.50 g, 1.80 mmol) in 80 mL of a mixed solvent (THF/H_2O, 3:1 *v*/*v*). The mixture was stirred at 100 °C for 24 h. After cooling to room temperature, the excess solvent was removed on a rotary evaporator under reduced pressure. After adding 200 mL of CH_2Cl_2, the solution was washed with deionized water (2 × 100 mL) and brine (100 mL), dried (Na_2SO_4), concentrated under vacuum, and finally recrystallized ($CHCl_3$/petroleum ether) to give **MeP5BPy** as light red crystals (621 mg, 73%). The ^{1}H NMR spectrum of **MeP5BPy** is shown below. ^{1}H NMR (500 MHz, $CDCl_3$, 298 K) δ = 8.43–8.42 (d, J = 5.0 Hz, 4H), 6.87–6.86 (m, 6H), 6.82 (s, 2H), 6.73 (s, 2H), 6.53 (s, 2H), 5.97 (s, 2H), 3.91−3.33 (m, 34H).

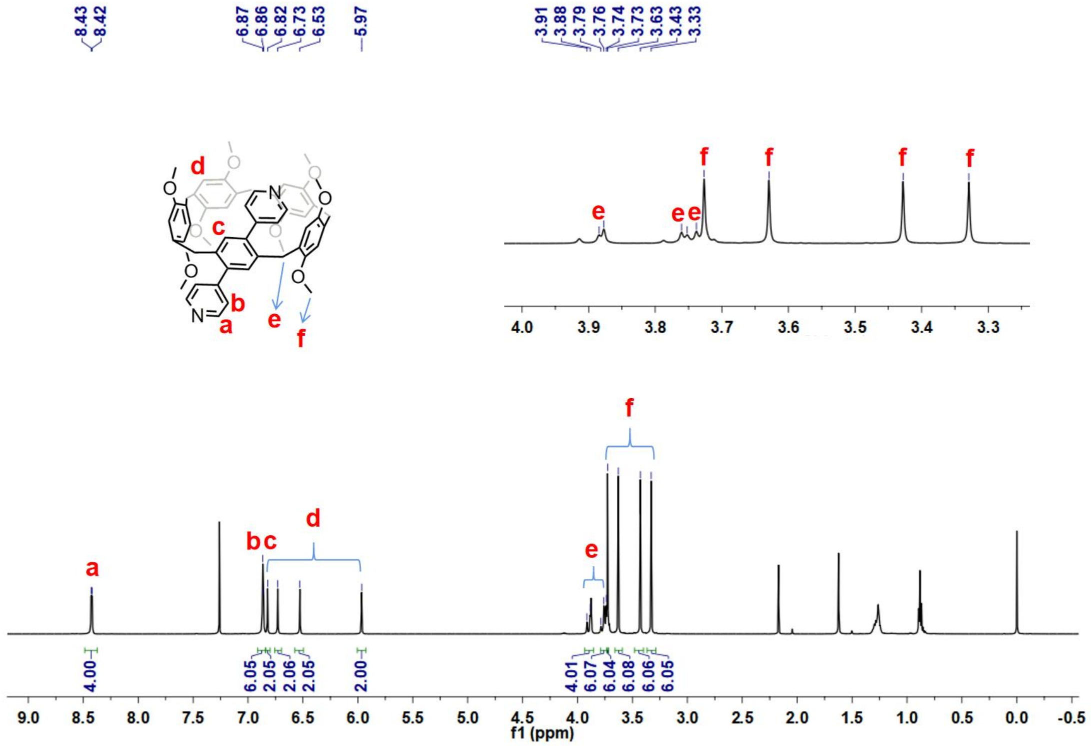

Supplementary Fig. 3 ^{1}H NMR (500 MHz, $CDCl_3$, 298 K) spectrum of **MeP5BPy**.

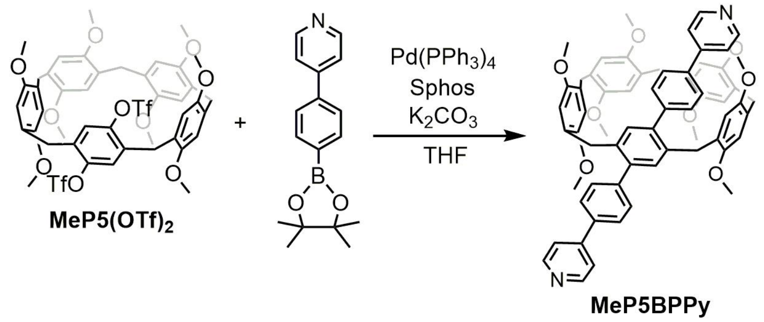

Supplementary Fig. 4 Preparation of **MeP5BPPy**.

MeP5BPPy: MeP5BPPy was synthesized *via* a Suzuki coupling procedure. **MeP5(OTf)$_2$** (988 mg, 1.00 mmol), 4-(4'-pyridyl)phenylboronic ester (843 mg, 3.00 mmol), K_2CO_3 (414 mg, 3.00 mmol), dicyclohexyl(2',6'-dimethoxy-2-biphenylyl)phosphine (Sphos, 205 mg, 500 μmol) and $Pd(PPh_3)_4$ (231 mg, 200 μmol) were mixed in 10 mL of THF. The mixture was heated at reflux for 24 h under an argon atmosphere. After cooling, the crude product was concentrated and recrystallized ($CHCl_3$/petroleum ether) to obtain **MeP5BPPy** as light yellow crystals (523 mg, yield 52%). ^1H NMR (500 MHz, $CDCl_3$, 298 K) (ppm): 8.72–8.71 (d, J = 5 Hz, 4H), 7.58–7.56 (d, J = 10 Hz, 4H), 7.48–7.46 (d, J = 10 Hz, 4H), 7.09–7.08 (d, J = 5 Hz, 4H), 6.97 (s, 2H), 6.70 (s, 2H), 6.64 (s, 2H), 6.57 (s, 2H), 5.91 (s, 2H), 3.94–3.82 (m, 10H), 3.74 (s, 6H), 3.51 (s, 6H), 3.33–3.31 (m, 12H).

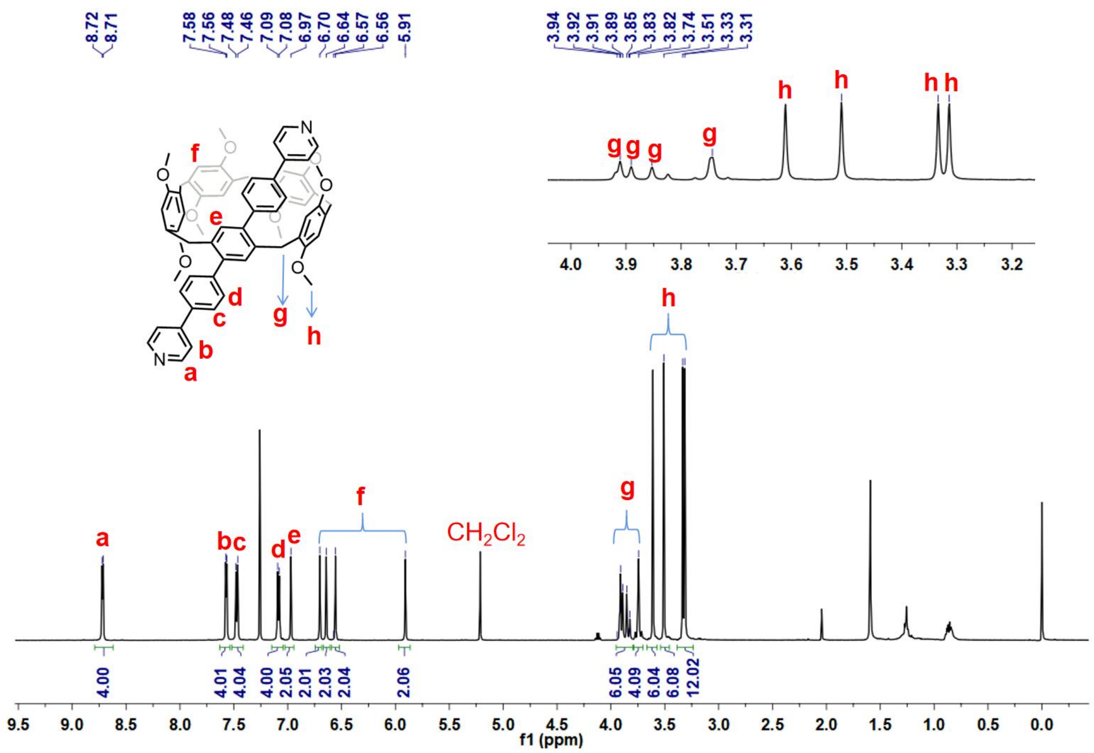

Supplementary Fig. 5 ^{1}H NMR (500 MHz, $CDCl_3$, 298 K) spectrum of **MeP5BPPy**.

4. Synthesis of MOFs

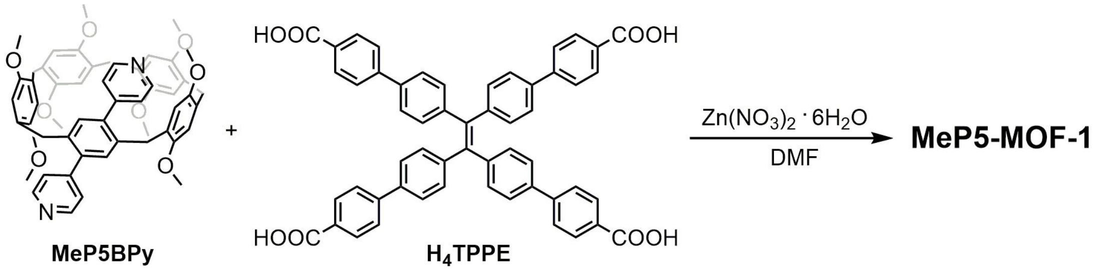

Supplementary Fig. 6 Preparation of **MeP5-MOF-1**.

MeP5-MOF-1: A DMF suspension (1.5 mL) of **MeP5BPy** (8.50 mg, 10.0 μmol), **H_4TPPE** (8.10 mg, 10.0 μmol) and $Zn(NO_3)_2{\cdot}6H_2O$ (5.95 mg, 20.0 μmol) was prepared in a small vial. This suspension was sonicated two minutes and then passed through a syringe filter to give a transparent solution, which was sealed, heated at a constant rate of 1 °C min^{-1} to 90 °C, kept at that temperature for 48 h, and cooled to room temperature at a constant cooling rate of 0.2 °C min^{-1}. Single crystals suitable for SCXRD measurements were obtained and washed with 12 mL of anhydrous DMF six times over three days. These single crystals were sequentially immersed in 12 mL of acetone three days, during which time the acetone was topped off twice daily. This

yielded the product in the form of transparent light yellow crystals (~6 mg, yield ~27%).

In a larger scale reaction, a DMF suspension (10 mL) of **MeP5BPy** (84.5 mg, 100 μmol), **H_4TPPE** (81.2 mg, 100 μmol) and $Zn(NO_3)_2{\cdot}6H_2O$ (59.5 mg, 200 μmol) was prepared in a 20-dram vial. The same procedure as described above was then followed.

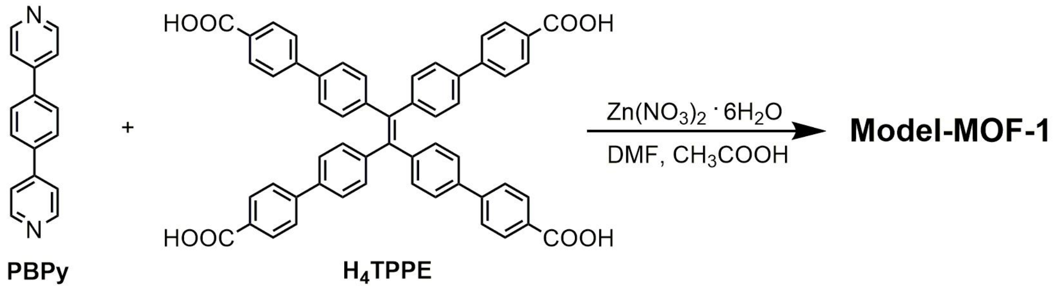

Supplementary Fig. 7 Preparation of **Model-MOF-1**.

Model-MOF-1: A DMF suspension (1.5 mL) of **PBPy** (2.30 mg, 10.0 μmol), **H_4TPPE** (8.10 mg, 10.0 μmol) and $Zn(NO_3)_2{\cdot}6H_2O$ (5.95 mg, 20.0 μmol) were prepared in a 2-dram vial. Then, acetic acid (50 μL) was added. The mixture was sonicated two minutes and then passed through a syringe filter to give a transparent solution, which was sealed, heated at a constant rate of 1 °C min^{-1} to 90 °C, kept at that temperature for 48 h and cooled to room temperature at a constant cooling rate of 0.2 °C min^{-1}. Single crystals suitable for SCXRD measurements were obtained and washed with 12 mL of fresh DMF six times over three days. These crystals were sequentially immersed in 12 mL of acetone three days, during which time the acetone was topped off twice daily. The product was given as transparent crystals (~4 mg, yield ~25%).

In a larger scale reaction, a DMF suspension (10 mL) of **PBPy** (23.2 mg, 100 μmol), **H_4TPPE** (81.2 mg, 100 μmol) and $Zn(NO_3)_2{\cdot}6H_2O$ (59.5 mg, 200 μmol) were prepared in a 20-dram vial. Acetic acid (200 μL) was then added before the same procedure as described above was followed.

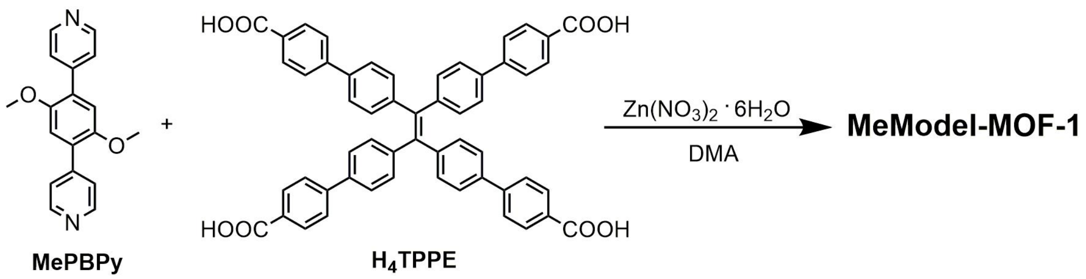

Supplementary Fig. 8 Preparation of **MeModel-MOF-1**.

MeModel-MOF-1: A DMA suspension (1.5 mL) of **MePBPy** (2.90 mg, 10.0 μmol), **H$_4$TPPE** (8.10 mg, 10.0 μmol) and $Zn(NO_3)_2{\cdot}6H_2O$ (5.95 mg, 20.0 μmol) was prepared in a 2-dram vial. The mixture was sonicated two minutes and then passed through a syringe filter to give a transparent solution, which was sealed, heated at a constant rate of 1 °C min^{-1} to 90 °C, kept at that temperature for 48 h, and cooled to room temperature at a constant cooling rate of 0.2 °C min^{-1}. Single crystals suitable for SCXRD measurements were obtained and washed with 12 mL of anhydrous DMA six times over three days. These crystals were sequentially immersed in 12 mL of acetone three days, during which time the acetone was topped off twice daily. The product was obtained as transparent crystals (~4 mg, yield ~24%).

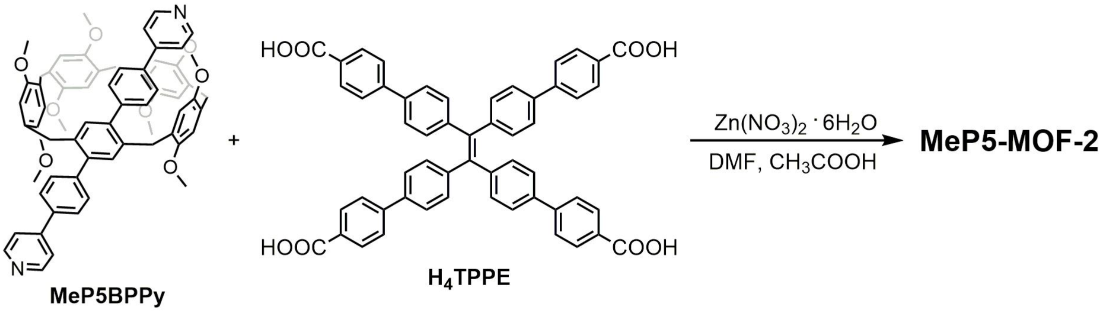

Supplementary Fig. 9 Preparation of **MeP5-MOF-2**.

MeP5-MOF-2: A DMF suspension (1.5 mL) of **MeP5BPPy** (10.0 mg, 10.0 μmol), **H$_4$TPPE** (8.10 mg, 10.0 μmol) and $Zn(NO_3)_2{\cdot}6H_2O$ (5.95 mg, 20.0 μmol) was prepared in a 2-dram vial. Then, acetic acid (20 μL) was added. The mixture was sonicated two minutes and then passed through a syringe filter to give a transparent solution, which was sealed, heated at a constant rate of 1 °C min^{-1} to 90 °C, kept at that temperature for 48 h and cooled to room temperature at a constant cooling rate of 0.2 °C min^{-1}. Single crystals suitable for SCXRD measurements were obtained and

washed with 12 mL of anhydrous DMF six times over three days. These crystals were sequentially immersed in 12 mL of acetone three days, during which time the acetone was topped off twice daily. The product was obtained as transparent flaxen crystals (~6 mg, yield ~24%).

In a larger scale reaction, a DMF suspension (10 mL) of **MeP5BPPy** (99.7 mg, 100 μmol), **H_4TPPE** (81.2 mg, 100 μmol) and $Zn(NO_3)_2{\cdot}6H_2O$ (59.5 mg, 200 μmol) was prepared in a 20-dram vial. Acetic acid (200 μL) was added and the same procedure as described above was then followed. The synthesis of *pS*-**MeP5-MOF-2** and *pR*-**MeP5-MOF-2** followed the same protocols as **MeP5-MOF-2**, with acetic acid (20 μL) being replaced by HNO_3 (5 μL) in the case of *pR*-**MeP5-MOF-2** so as to obtain better quality single crystals.

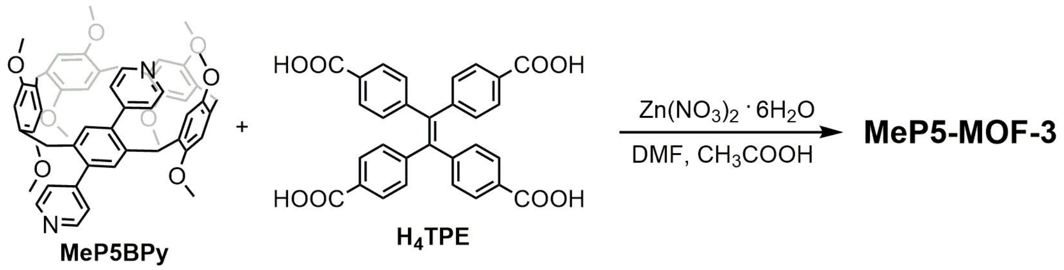

Supplementary Fig. 10 Preparation of **MeP5-MOF-3**.

MeP5-MOF-3: A DMF suspension (1.5 mL) of **MeP5BPy** (8.50 mg, 10.0 μmol), **H_4TPE** (5.10 mg, 10.0 μmol) and $Zn(NO_3)_2{\cdot}6H_2O$ (5.95 mg, 20.0 μmol) was prepared in a 2-dram vial. Then, acetic acid (100 μL) was added. The mixture was sonicated two minutes and then passed through a syringe filter to give a transparent solution, which was sealed, heated at a constant rate of 1 °C min^{-1} to 90 °C, kept at that temperature for 48 h and cooled to room temperature at a constant cooling rate of 0.2 °C min^{-1}. Single crystals suitable for SCXRD measurements were obtained and washed with 12 mL of anhydrous DMF six times over three days. These crystals were sequentially immersed in 12 mL of acetone three days, during which time the acetone was topped off twice daily. The product was obtained as transparent crystals (~2 mg, yield ~11%).

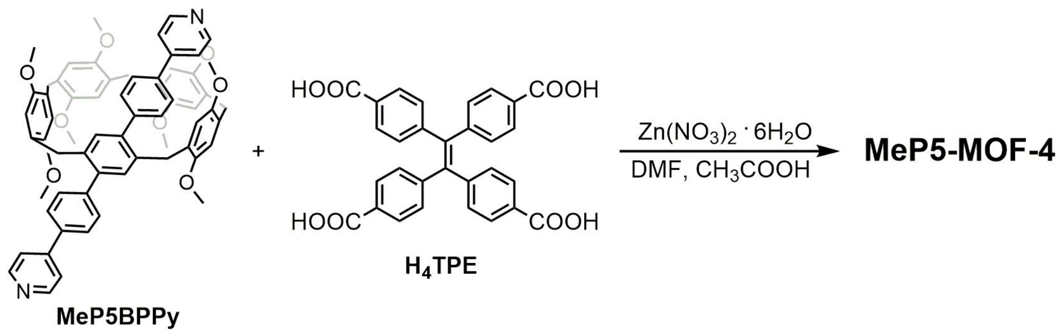

Supplementary Fig. 11 Preparation of **MeP5-MOF-4**.

MeP5-MOF-4: A DMF suspension (1.5 mL) of **MeP5BPPy** (10.0 mg, 10.0 μmol), **H_4TPE** (5.10 mg, 10.0 μmol) and $Zn(NO_3)_2{\cdot}6H_2O$ (5.95 mg, 20.0 μmol) was prepared in a 2-dram vial. Then, acetic acid (20 μL) was added. The mixture was sonicated two minutes and then passed through a syringe filter to give a transparent solution, which was sealed, heated at a constant rate of 1 °C min^{-1} to 90 °C, kept at that temperature for 48 h and cooled to room temperature at a constant cooling rate of 0.2 °C min^{-1}. Single crystals suitable for SCXRD measurements were obtained and washed with 12 mL of anhydrous DMF six times over three days. These crystals were sequentially immersed in 12 mL of acetone three days, during which time the acetone was topped off twice daily. The product was obtained as transparent light yellow crystals (~2 mg, yield ~10%).

5. ^{1}H NMR spectroscopic studies of MOFs

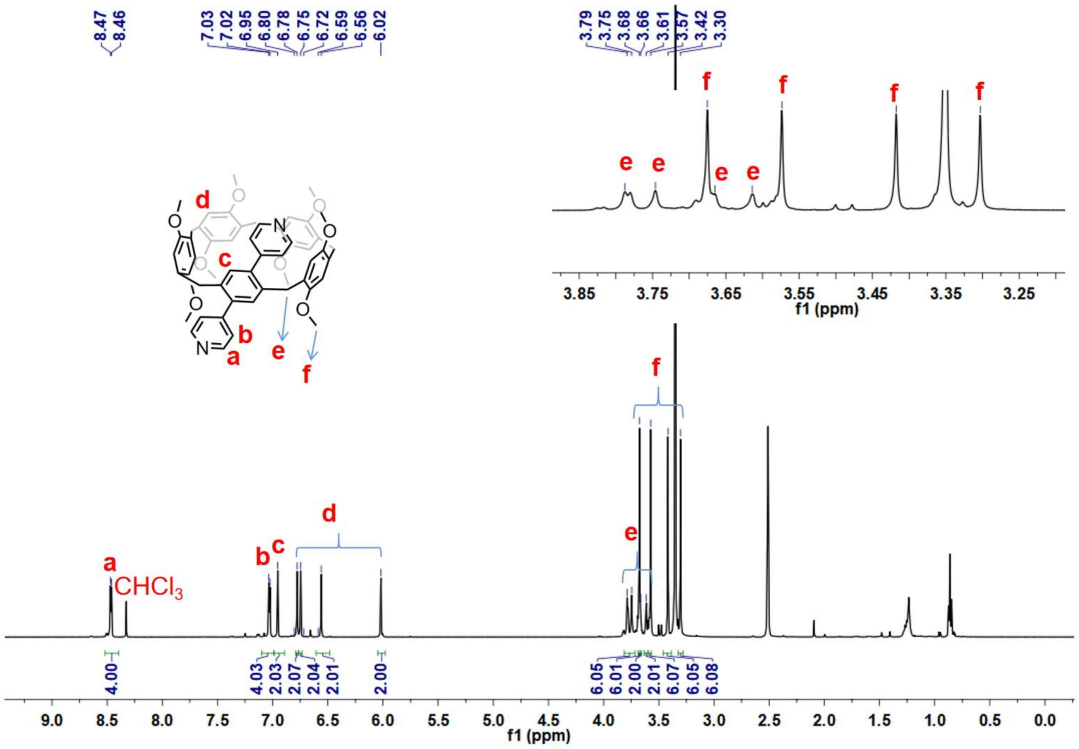

Supplementary Fig. 12 ^{1}H NMR (500 MHz, DMSO-d_6, 298 K) spectrum of **MeP5BPy**.

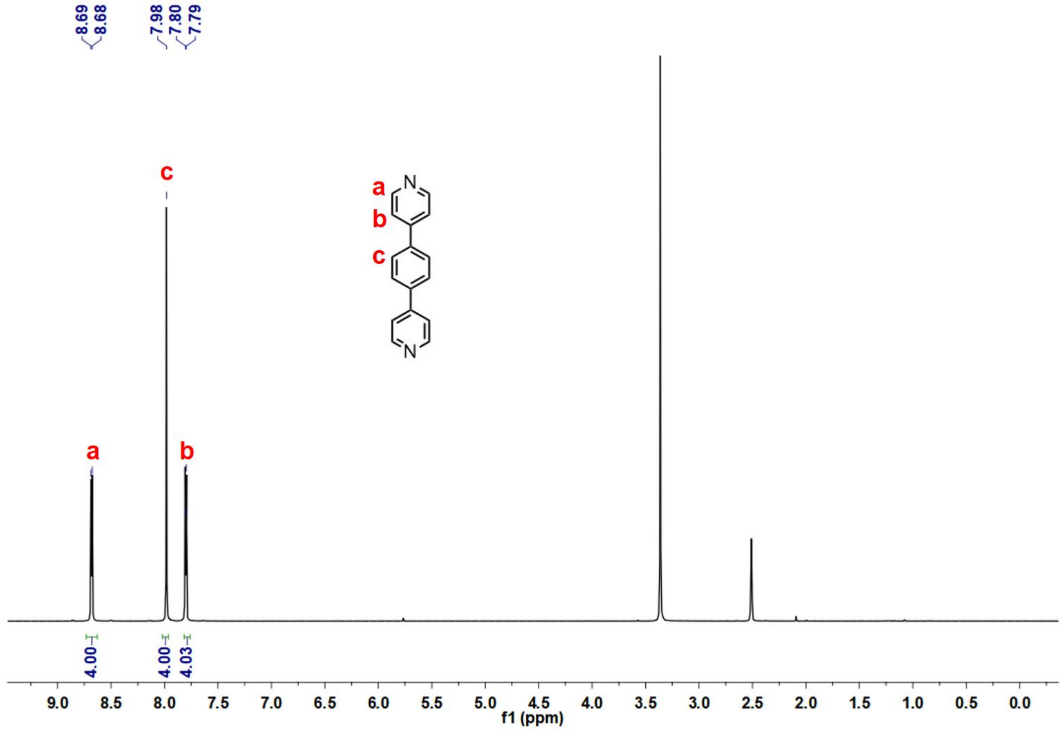

Supplementary Fig. 13 ^{1}H NMR (500 MHz, DMSO-d_6, 298 K) spectrum of **PBPy**.

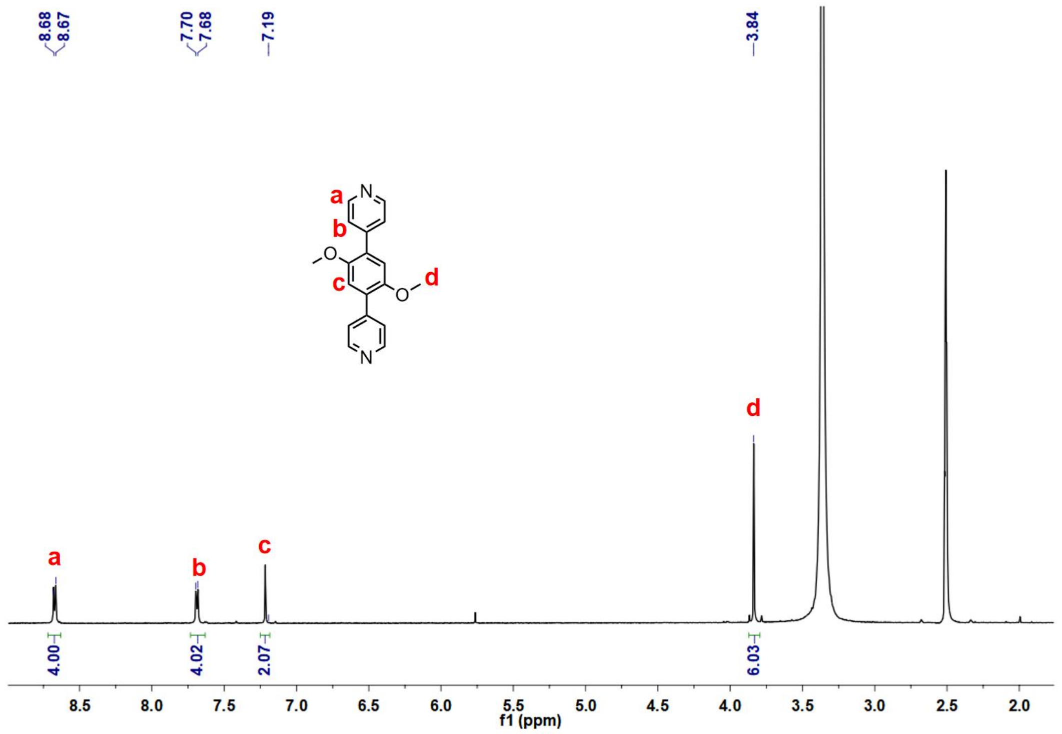

Supplementary Fig. 14 ^{1}H NMR (400 MHz, DMSO-d_6, 298 K) spectrum of **MePBPy**.

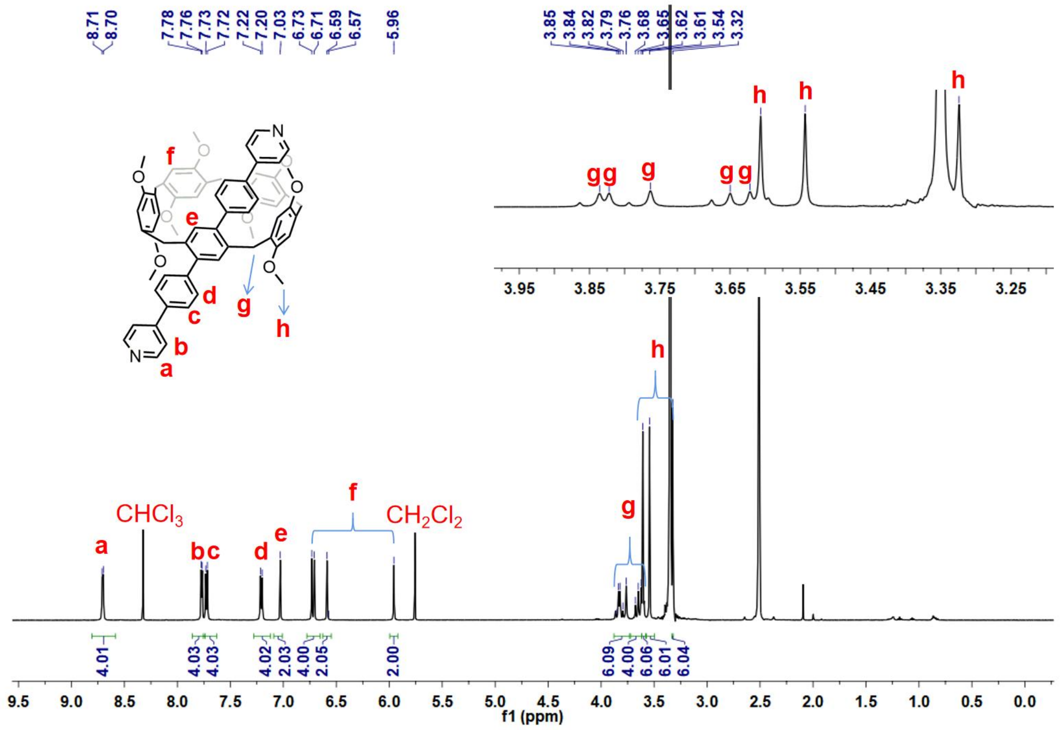

Supplementary Fig. 15 ^{1}H NMR (500 MHz, DMSO-d_6, 298 K) spectrum of **MeP5BPPy**. Here one signal of proton **h** has been merged into the signal of H_2O at 3.30–3.40 ppm.

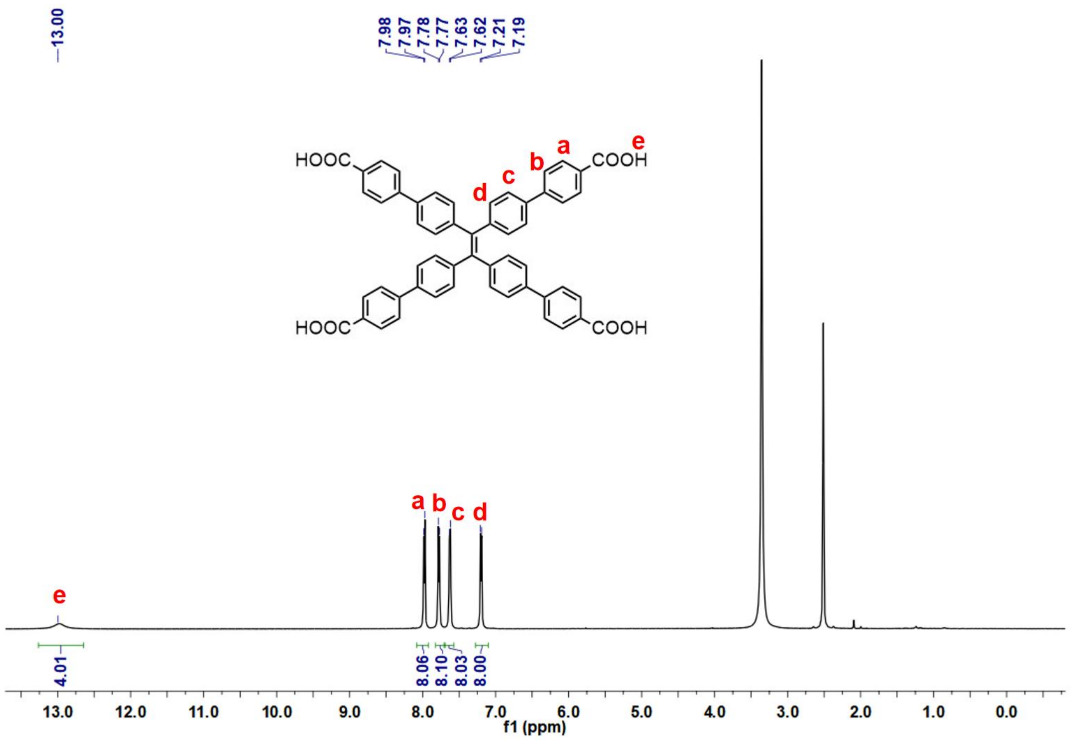

Supplementary Fig. 16 ^{1}H NMR (500 MHz, DMSO-d_6, 298 K) spectrum of **H_4TPPE**.

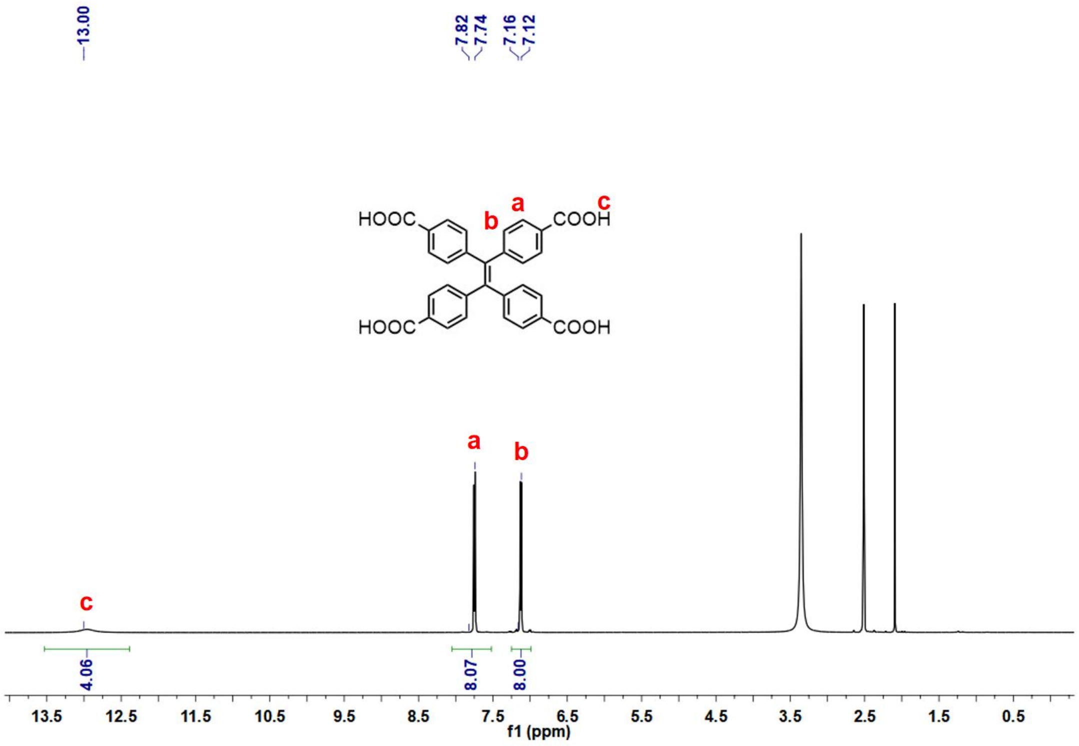

Supplementary Fig. 17 ^{1}H NMR (500 MHz, DMSO-d_6, 298 K) spectrum of **H_4TPE**.

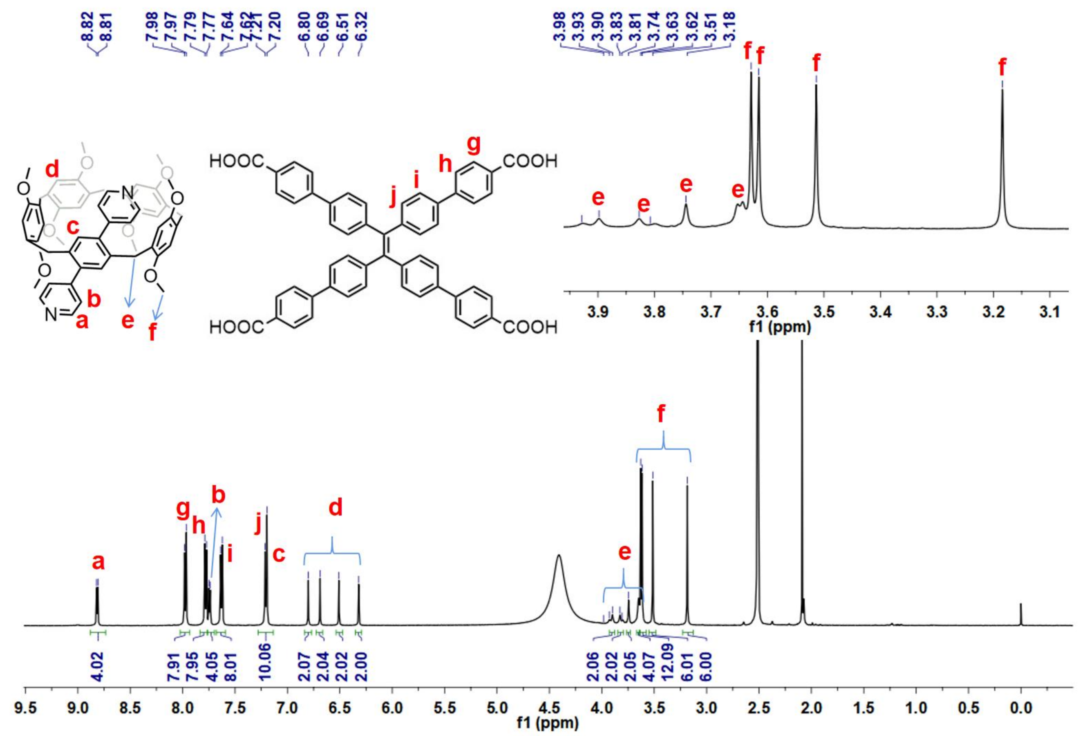

Supplementary Fig. 18 ^{1}H NMR (500 MHz, DMSO-d_6:DCl = 100:1, 298 K) spectrum of **MeP5-MOF-1** after digestion.

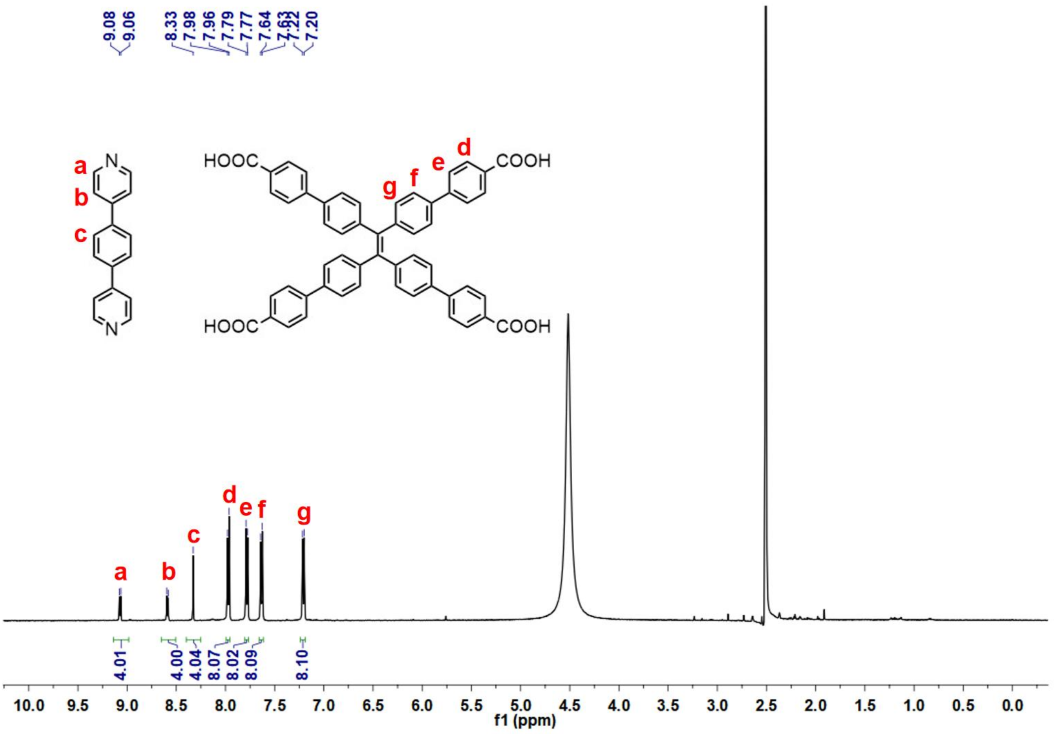

Supplementary Fig. 19 ^{1}H NMR (500 MHz, DMSO-d_6:DCl = 100:1, 298 K) spectrum of **Model-MOF-1** after digestion.

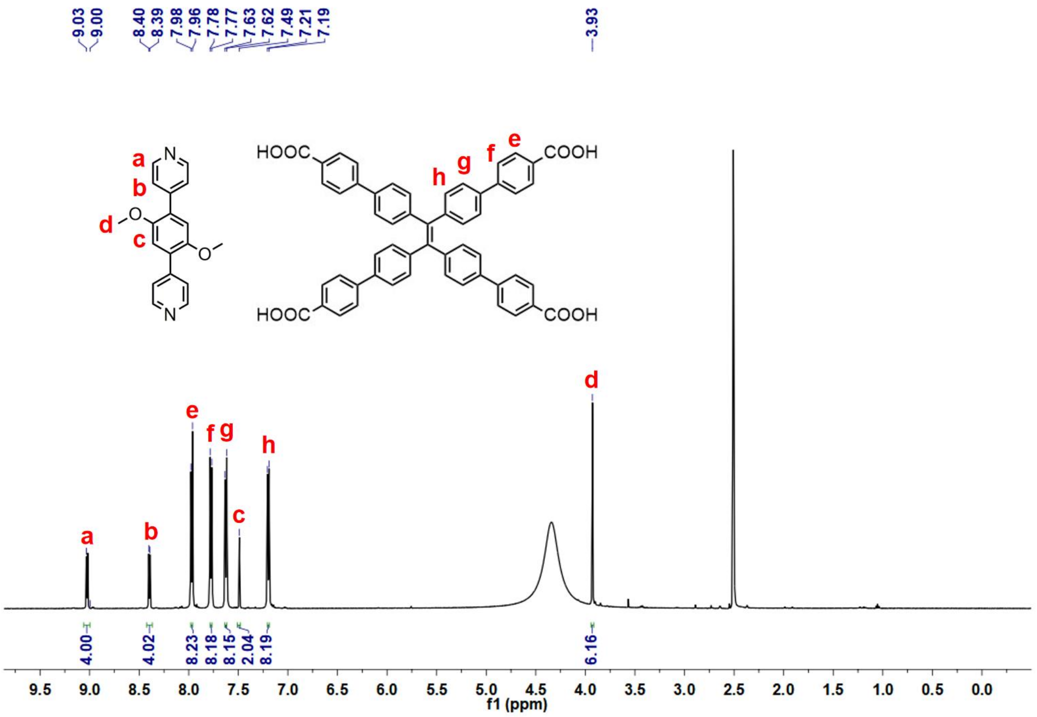

Supplementary Fig. 20 ^{1}H NMR (400 MHz, DMSO-d_6:DCl = 100:1, 298 K) spectrum of **MeModel-MOF-1** after digestion.

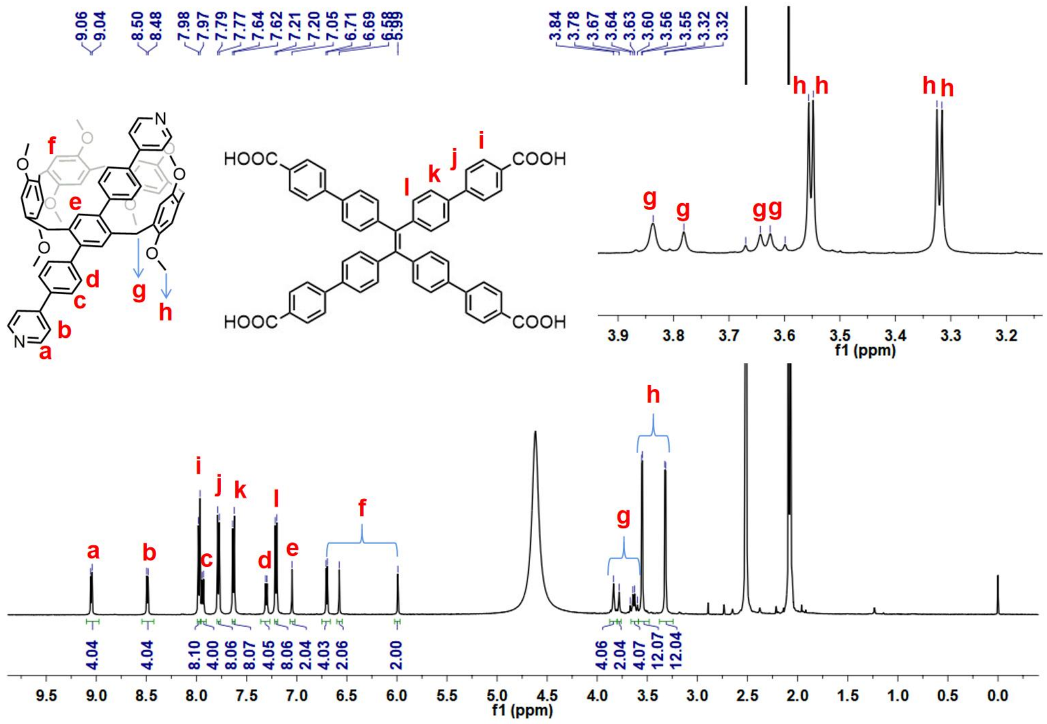

Supplementary Fig. 21 ^{1}H NMR (500 MHz, DMSO-d_6:DCl = 100:1, 298 K) spectrum of **MeP5-MOF-2** after digestion.

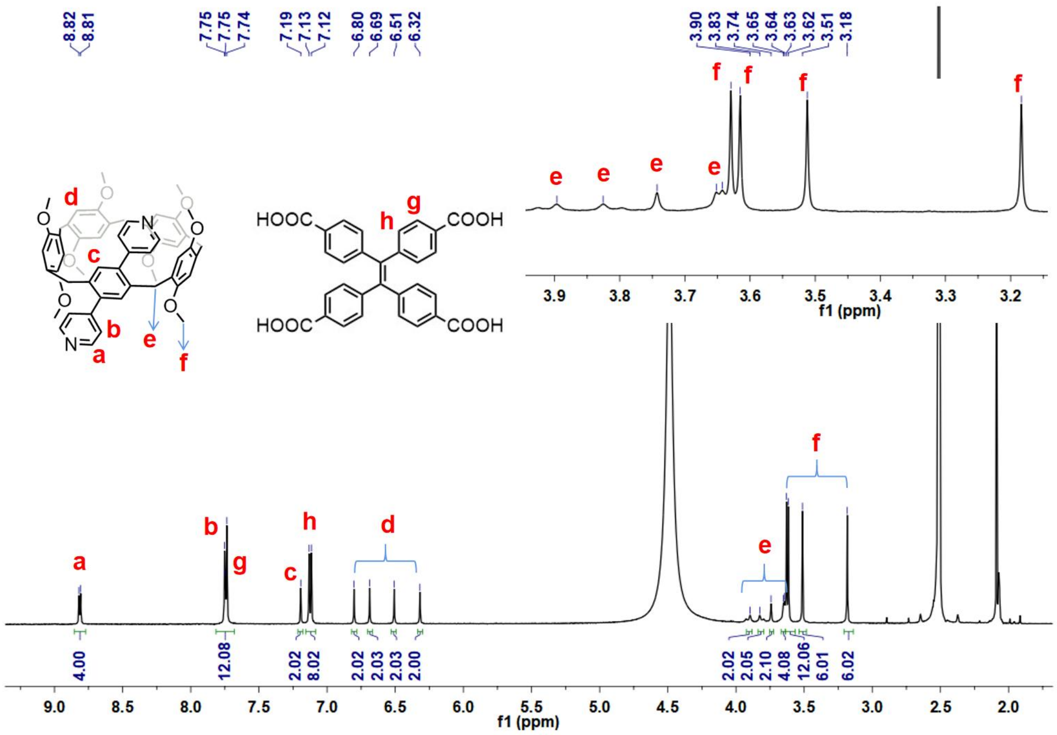

Supplementary Fig. 22 ^{1}H NMR (500 MHz, DMSO-d_6:DCl = 100:1, 298 K) spectrum of **MeP5-MOF-3** after digestion.

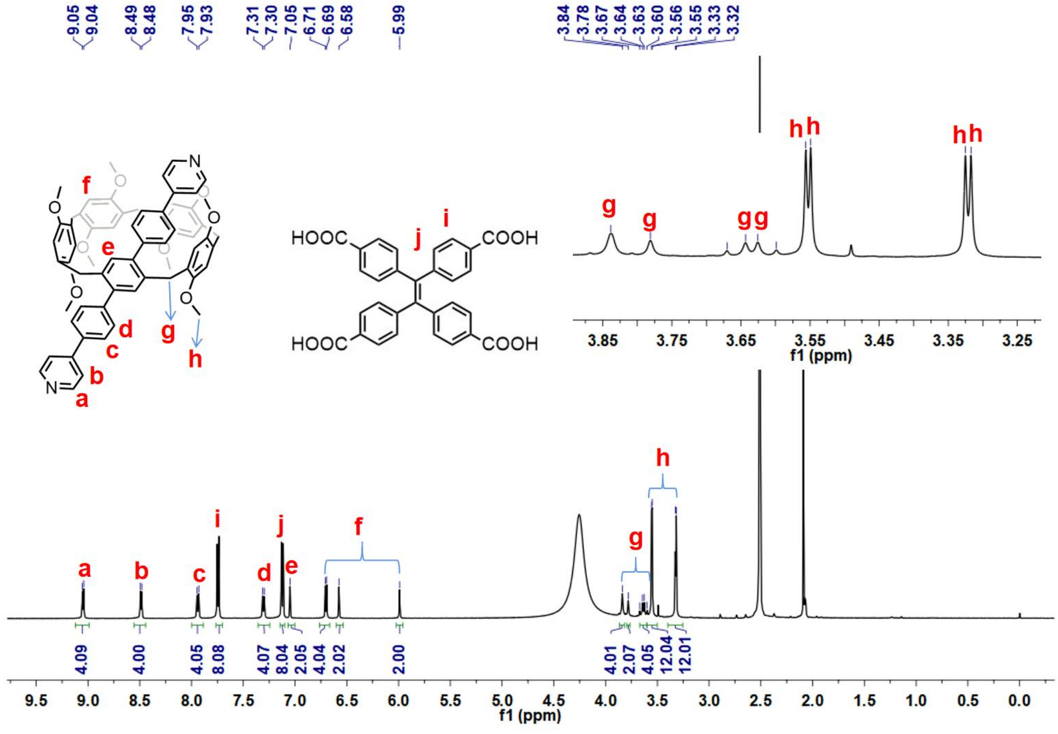

Supplementary Fig. 23 ^{1}H NMR (500 MHz, DMSO-d_6:DCl = 100:1, 298 K) spectrum of **MeP5-MOF-4** after digestion.

6. Single crystal X-ray data

Supplementary Table 1 Experimental single crystal X-ray data of **MeP5BPPy**

Empirical formula	$C_{65}H_{61}N_2O_8$
Formula weight	998.15
Temperature/K	193
Crystal system	monoclinic
Space group	$P2_1/c$
a/Å	23.0941(8)
b/Å	17.3444(6)
c/Å	13.6476(4)
α/°	90
β/°	106.3310(10)
γ/°	90
Volume/Å^3	5246.0(3)
Z	4
ρ_{calc}(g/cm^3)	1.264
μ/mm^{-1}	0.083
F(000)	2116
Crystal size/mm^3	0.13 × 0.12 × 0.1
Radiation	MoKα (λ = 0.71073)
2θ range for data collection/°	3.676 to 52.784
Index ranges	$-24 \le h \le 28$, $-21 \le k \le 21$, $-17 \le l \le 16$
Reflections collected	43798
Independent reflections	10737 [R_{int} = 0.0627, R_{sigma} = 0.0604]
Data/restraints/parameters	10737/36/764
Goodness-of-fit on F^2	1.071
Final R indexes [$I \geq 2\sigma (I)$]	R_1 = 0.0696, wR_2 = 0.1315
Final R indexes [all data]	R_1 = 0.1209, wR_2 = 0.1541
Largest difference peak/hole/e.Å^{-3}	0.25/–0.42
CCDC-number	2211420

Supplementary Table 2 Experimental single crystal X-ray data of **MeP5-MOF-1**

Formula	[Zn_2(**MeP5BPy**)(**TPPE**)]
Empirical formula	$C_{70}H_{44}N_2O_8Zn_2$
Formula weight	1171.81
Temperature/K	193
Crystal system	orthorhombic
Space group	*Pmmm*
a/Å	16.449(3)
b/Å	18.309(3)
c/Å	20.203(4)
α/°	90
β/°	90
γ/°	90
Volume/Å^3	6084.6(17)
Z	1
ρ_{calc}(g/cm^3)	0.32
μ/mm^{-1}	0.211
F(000)	602
Crystal size/mm^3	0.13 × 0.12 × 0.1
Radiation	MoKα (λ = 0.71073)
2θ range for data collection/°	3.892 to 52.854
Index ranges	$-19 \le h \le 20$, $-22 \le k \le 22$, $-25 \le l \le 13$
Reflections collected	24329
Independent reflections	6854 [R_{int} = 0.0856, R_{sigma} = 0.0850]
Data/restraints/parameters	6854/53/145
Goodness-of-fit on F^2	1.011
Final R indexes [$I \geq 2\sigma (I)$]	R_1 = 0.0688, wR_2 = 0.1817
Final R indexes [all data]	R_1 = 0.0868, wR_2 = 0.1911
Largest difference peak/hole/e Å^{-3}	1.12/–0.62
CCDC-number	2211421

Supplementary Table 3 Experimental single crystal X-ray data of $(DMF)_2$@**Model-MOF-1**

Formula	$[Zn_2$(**PBPy**)(**TPPE**)]$(DMF)_2$
Empirical formula	$C_{76}H_{58}N_4O_{10}Zn_2$
Formula weight	1318.00
Temperature/K	193
Crystal system	triclinic
Space group	$P\bar{1}$
a/Å	16.4239(13)
b/Å	18.2159(13)
c/Å	20.2865(15)
α/°	78.748(4)
β/°	89.927(4)
γ/°	77.576(4)
Volume/Å^3	5807.9(8)
Z	2
ρ_{calc}(g/cm^3)	0.754
μ/mm^{-1}	0.521
F(000)	1364
Crystal size/mm^3	0.13 × 0.12 × 0.1
Radiation	GaKα (λ = 1.34139)
2θ range for data collection/°	3.868 to 107.812
Index ranges	$-19 \le h \le 19$, $-21 \le k \le 21$, $0 \le l \le 24$
Reflections collected	20969
Independent reflections	20969 [R_{int} = 0.0996, R_{sigma} = 0.0986]
Data/restraints/parameters	20969/84/834
Goodness-of-fit on F^2	1.13
Final R indexes [$I \geq 2\sigma (I)$]	R_1 = 0.1388, wR_2 = 0.3246
Final R indexes [all data]	R_1 = 0.1814, wR_2 = 0.3489
Largest difference peak/hole/e Å^{-3}	1.17/–0.74
CCDC-number	2217237

Supplementary Table 4 Experimental single crystal X-ray data of $(DMA)_2$@**MeModel-MOF-1**

Formula	$[Zn_2($**MePBPy**$)($**TPPE**$)](DMA)_2$
Empirical formula	$C_{80}H_{66}N_4O_{12}Zn_2$
Formula weight	1406.10
Temperature/K	262
Crystal system	triclinic
Space group	$P\bar{1}$
a/Å	16.380(3)
b/Å	18.015(3)
c/Å	20.210(4)
α/°	102.337(8)
β/°	90.337(9)
γ/°	115.845(8)
Volume/Å^3	5209.9(17)
Z	2
ρ_{calc}(g/cm^3)	0.896
μ/mm^{-1}	0.604
F(000)	1460
Crystal size/mm^3	0.13 × 0.12 × 0.1
Radiation	GaKα (λ = 1.34139)
2θ range for data collection/°	3.918 to 107.814
Index ranges	$-19 \le h \le 19$, $-20 \le k \le 21$, $-24 \le l \le 24$
Reflections collected	97206
Independent reflections	18977 [R_{int} = 0.1097, R_{sigma} = 0.0935]
Data/restraints/parameters	18977/134/917
Goodness-of-fit on F^2	1.031
Final R indexes [$I \ge 2\sigma (I)$]	R_1 = 0.0999, wR_2 = 0.2486
Final R indexes [all data]	R_1 = 0.1676, wR_2 = 0.2852
Largest difference peak/hole/e Å^{-3}	0.96/–0.75
CCDC-number	2213797

Supplementary Table 5 Experimental single crystal X-ray data of **MeP5-MOF-2**

Formula	[Zn_2(**MeP5BPPy**)(**TPPE**)]
Empirical formula	$C_{119}H_{92}N_2O_{16}Zn_2$
Formula weight	1936.68
Temperature/K	193
Crystal system	triclinic
Space group	$P\bar{1}$
a/Å	17.0634(12)
b/Å	19.7634(14)
c/Å	26.7805(19)
α/°	85.077(3)
β/°	72.719(3)
γ/°	89.875(4)
Volume/Å^3	8589.1(11)
Z	2
ρ_{calc}(g/cm^3)	0.749
μ/mm^{-1}	0.432
F(000)	2016
Crystal size/mm^3	0.13 × 0.12 × 0.1
Radiation	GaKα (λ = 1.34139)
2θ range for data collection/°	3.018 to 121.084
Index ranges	$-22 \le h \le 22$, $-25 \le k \le 25$, $-34 \le l \le 34$
Reflections collected	347480
Independent reflections	38737 [R_{int} = 0.0667, R_{sigma} = 0.0370]
Data/restraints/parameters	38737/368/1211
Goodness-of-fit on F^2	1.128
Final R indexes [$I \ge 2\sigma (I)$]	R_1 = 0.1181, wR_2 = 0.3115
Final R indexes [all data]	R_1 = 0.1323, wR_2 = 0.3200
Largest difference peak/hole/e Å^{-3}	1.02/–1.55
CCDC-number	2211422

Supplementary Table 6 Experimental single crystal X-ray data of DMF@**MeP5-MOF-2**

Formula	[Zn_2(**MeP5BPPy**)(**TPPE**)](DMF)
Empirical formula	$C_{122}H_{99}N_3O_{17}Zn_2$
Formula weight	2009.78
Temperature/K	193
Crystal system	triclinic
Space group	$P\bar{1}$
a/Å	16.9571(13)
b/Å	19.8151(14)
c/Å	26.719(3)
α/°	83.462(6)
β/°	71.545(4)
γ/°	89.884(4)
Volume/Å^3	8455.3(12)
Z	2
ρ_{calc}(g/cm^3)	0.789
μ/mm^{-1}	0.45
F(000)	2096
Crystal size/mm^3	0.13 × 0.12 × 0.1
Radiation	GaKα (λ = 1.34139)
2θ range for data collection/°	3.054 to 107.812
Index ranges	$-20 \le h \le 19$, $-23 \le k \le 23$, $-31 \le l \le 32$
Reflections collected	70402
Independent reflections	30837 [R_{int} = 0.1081, R_{sigma} = 0.1590]
Data/restraints/parameters	30837/866/1322
Goodness-of-fit on F^2	0.999
Final R indexes [$I \ge 2\sigma (I)$]	R_1 = 0.1049, wR_2 = 0.2898
Final R indexes [all data]	R_1 = 0.1815, wR_2 = 0.3402
Largest difference peak/hole/e Å^{-3}	0.96/–1.32
CCDC-number	2211423

Supplementary Table 7 Experimental single crystal X-ray diffraction data of $(DMF)_3$@**MeP5-MOF-2**

Formula	$[Zn_2$(**MeP5BPPy**)(**TPPE**)]$(DMF)_3$
Empirical formula	$C_{128}H_{113}N_5O_{19}Zn_2$
Formula weight	2155.97
Temperature/K	193
Crystal system	triclinic
Space group	$P\bar{1}$
a/Å	17.335(3)
b/Å	19.597(3)
c/Å	26.871(4)
α/°	85.269(7)
β/°	78.160(7)
γ/°	89.939(6)
Volume/Å^3	8903(2)
Z	2
ρ_{calc}(g/cm^3)	0.804
μ/mm^{-1}	0.449
F(000)	2256
Crystal size/mm^3	0.13 × 0.12 × 0.1
Radiation	GaKα (λ = 1.34139)
2θ range for data collection/°	2.934 to 107.814
Index ranges	$-20 \leq h \leq 20$, $-23 \leq k \leq 22$, $-32 \leq l \leq 32$
Reflections collected	84336
Independent reflections	31475 [R_{int} = 0.0628, R_{sigma} = 0.0668]
Data/restraints/parameters	31475/171/1400
Goodness-of-fit on F^2	1.168
Final R indexes [$I \geq 2\sigma (I)$]	R_1 = 0.0995, wR_2 = 0.2931
Final R indexes [all data]	R_1 = 0.1263, wR_2 = 0.3209
Largest difference peak/hole/e Å^{-3}	1.40/–1.28
CCDC-number	2217239

Supplementary Table 8 Experimental single crystal X-ray diffraction data of **Model-MOF-2**

Formula	$[Zn_2(\mathbf{TPPE})(H_2O)_2]$
Empirical formula	$C_{54}H_{36}O_{10}Zn_2$
Formula weight	975.57
Temperature/K	193
Crystal system	orthorhombic
Space group	$P2_12_12_1$
a/Å	20.2233(14)
b/Å	51.597(4)
c/Å	16.3953(11)
α/°	90
β/°	90
γ/°	90
Volume/Å^3	17108(2)
Z	4
ρ_{calc}(g/cm^3)	0.379
μ/mm^{-1}	0.314
F(000)	2000
Crystal size/mm^3	0.13 × 0.12 × 0.1
Radiation	GaKα (λ = 1.34139)
2θ range for data collection/°	4.082 to 105.962
Index ranges	$-17 \leq h \leq 23, -61 \leq k \leq 57, -11 \leq l \leq 19$
Reflections collected	60353
Independent reflections	28610 [R_{int} = 0.1506, R_{sigma} = 0.2182]
Data/restraints/parameters	28610/848/502
Goodness-of-fit on F^2	1.046
Final R indexes [$I \geq 2\sigma (I)$]	R_1 = 0.1288, wR_2 = 0.2990
Final R indexes [all data]	R_1 = 0.2302, wR_2 = 0.3454
Largest difference peak/hole/e Å^{-3}	0.30/–0.95
CCDC-number	2211425

Supplementary Table 9 Experimental single crystal X-ray diffraction data of *pS*-**MeP5-MOF-2**

Formula	[Zn_2(*pS*-**MeP5BPPy**)(**TPPE**)]
Empirical formula	$C_{119}H_{92}N_2O_{16}Zn_2$
Formula weight	1936.68
Temperature/K	193
Crystal system	orthorhombic
Space group	$P2_12_12$
a/Å	20.313(2)
b/Å	50.970(6)
c/Å	16.226(2)
α/°	90
β/°	90
γ/°	90
Volume/Å^3	16800(4)
Z	4
ρ_{calc}(g/cm^3)	0.766
μ/mm^{-1}	0.441
F(000)	4032
Crystal size/mm^3	0.13 × 0.12 × 0.1
Radiation	GaKα (λ = 1.34139)
2θ range for data collection/°	4.972 to 114.294
Index ranges	$-25 \leq h \leq 23$, $-62 \leq k \leq 56$, $-20 \leq l \leq 20$
Reflections collected	92506
Independent reflections	32229 [R_{int} = 0.0974, R_{sigma} = 0.1208]
Data/restraints/parameters	32229/420/1288
Goodness-of-fit on F^2	0.911
Final R indexes [$I \geq 2\sigma (I)$]	R_1 = 0.0540, wR_2 = 0.1226
Final R indexes [all data]	R_1 = 0.0962, wR_2 = 0.1359
Largest difference peak/hole/e Å^{-3}	0.27/–0.30
Flack parameter	0.28(2)
CCDC-number	2217240

Supplementary Table 10 Experimental single crystal X-ray diffraction data of *pR*-**MeP5-MOF-2**

Formula	[Zn_2(*pR*-**MeP5BPPy**)(**TPPE**)]
Empirical formula	$C_{119}H_{92}N_2O_{16}Zn_2$
Formula weight	1936.68
Temperature/K	193
Crystal system	orthorhombic
Space group	$P2_12_12$
a/Å	20.4547(12)
b/Å	50.956(3)
c/Å	16.1010(9)
α/°	90
β/°	90
γ/°	90
Volume/Å^3	16782.0(17)
Z	4
ρ_{calc}(g/cm^3)	0.767
μ/mm^{-1}	0.442
F(000)	4032
Crystal size/mm^3	0.13 × 0.12 × 0.1
Radiation	GaKα (λ = 1.34139)
2θ range for data collection/°	3.016 to 120.648
Index ranges	$-26 \le h \le 24, -65 \le k \le 61, -16 \le l \le 20$
Reflections collected	132422
Independent reflections	34787 [R_{int} = 0.0666, R_{sigma} = 0.0687]
Data/restraints/parameters	34787/551/1198
Goodness-of-fit on F^2	0.999
Final R indexes [$I \geq 2\sigma (I)$]	R_1 = 0.0681, wR_2 = 0.1848
Final R indexes [all data]	R_1 = 0.1037, wR_2 = 0.2038
Largest difference peak/hole/e Å^{-3}	0.71/–0.58
Flack parameter	0.26(3)
CCDC-number	2217241

Supplementary Table 11 Experimental single crystal X-ray diffraction data of **MeP5-MOF-3**

Formula	[Zn_2(**MeP5BPy**)(**TPE**)]
Empirical formula	$C_{46}H_{28}N_2O_8Zn_2$
Formula weight	867.44
Temperature/K	193
Crystal system	monoclinic
Space group	*P*2/*m*
a/Å	13.2174(14)
b/Å	11.3745(14)
c/Å	18.201(2)
α/°	90
β/°	98.905(6)
γ/°	90
Volume/Å^3	2703.3(6)
Z	1
ρ_{calc}(g/cm^3)	0.533
μ/mm^{-1}	0.473
F(000)	442
Crystal size/mm^3	0.13 × 0.12 × 0.1
Radiation	GaKα (λ = 1.34139)
2θ range for data collection/°	4.276 to 107.8
Index ranges	$-15 \le h \le 15$, $0 \le k \le 13$, $0 \le l \le 21$
Reflections collected	5203
Independent reflections	5203 [R_{int} = 0.1290, R_{sigma} = 0.0934]
Data/restraints/parameters	5203/114/203
Goodness-of-fit on F^2	1.089
Final *R* indexes [$I \geq 2\sigma(I)$]	R_1 = 0.0977, wR_2 = 0.2639
Final *R* indexes [all data]	R_1 = 0.1365, wR_2 = 0.2986
Largest difference peak/hole/e Å^{-3}	0.75/–1.01
CCDC-number	2211424

Supplementary Table 12 Experimental single crystal X-ray diffraction data of **MeP5-MOF-4**

Formula	[Zn_2(**MeP5BPPy**)(**TPPE**)]
Empirical formula	$C_{58}H_{36}N_2O_8Zn_2$
Formula weight	1019.63
Temperature/K	193
Crystal system	monoclinic
Space group	$P2/m$
a/Å	13.1993(9)
b/Å	11.4758(7)
c/Å	26.8418(19)
α/°	90
β/°	95.824(4)
γ/°	90
Volume/Å^3	4044.8(5)
Z	1
ρ_{calc}(g/cm^3)	0.419
μ/mm^{-1}	0.333
F(000)	522
Crystal size/mm^3	0.13 × 0.12 × 0.1
Radiation	GaKα (λ = 1.34139)
2θ range for data collection/°	2.878 to 107.804
Index ranges	$-15 \le h \le 15$, $-13 \le k \le 11$, $-24 \le l \le 32$
Reflections collected	22366
Independent reflections	7649 [R_{int} = 0.0826, R_{sigma} = 0.0784]
Data/restraints/parameters	7649/514/339
Goodness-of-fit on F^2	1.143
Final R indexes [$I \geq 2\sigma (I)$]	R_1 = 0.1498, wR_2 = 0.3265
Final R indexes [all data]	R_1 = 0.1806, wR_2 = 0.3435
Largest difference peak/hole/e Å^{-3}	2.14/–1.14
CCDC-number	2217410

Supplementary Table 13 Experimental single crystal X-ray diffraction data of **(Py)$_2$@P5**

Empirical formula	$C_{65}H_{80}N_2O_{10}$
Formula weight	1049.31
Temperature/K	193
Crystal system	orthorhombic
Space group	*Pccn*
a/Å	12.1492(5)
b/Å	19.8813(7)
c/Å	24.9952(10)
α/°	90
β/°	90
γ/°	90
Volume/Å^3	6037.4(4)
Z	4
ρ_{calc}(g/cm^3)	1.154
μ/mm^{-1}	0.392
F(000)	2256
Crystal size/mm^3	0.13 × 0.12 × 0.1
Radiation	GaKα (λ = 1.34139)
2θ range for data collection/°	6.152 to 120.35
Index ranges	$-15 \le h \le 15$, $-25 \le k \le 24$, $-25 \le l \le 31$
Reflections collected	42666
Independent reflections	6718 [R_{int} = 0.0759, R_{sigma} = 0.0507]
Data/restraints/parameters	6718/104/393
Goodness-of-fit on F^2	1.026
Final R indexes [$I \geq 2\sigma (I)$]	R_1 = 0.0617, wR_2 = 0.1528
Final R indexes [all data]	R_1 = 0.1145, wR_2 = 0.1824
Largest difference peak/hole/e Å^{-3}	0.48/–0.21
CCDC-number	2216441

Supplementary Table 14 Experimental single crystal X-ray diffraction data of **Tol@MeP5-MOF-2**

Formula	[Zn_2(**MeP5BPPy**)(**TPPE**)](**Tol**)
Empirical formula	$C_{126}H_{100}N_2O_{16}Zn_2$
Formula weight	2028.81
Temperature/K	193
Crystal system	triclinic
Space group	$P\bar{1}$
a/Å	15.652(2)
b/Å	20.687(3)
c/Å	23.936(3)
α/°	67.358(4)
β/°	80.274(4)
γ/°	89.931(4)
Volume/Å^3	7032.3(16)
Z	2
ρ_{calc}(g/cm^3)	0.958
μ/mm^{-1}	0.392
F(000)	2116
Crystal size/mm^3	0.13 × 0.12 × 0.1
Radiation	MoKα (λ = 0.71073)
2θ range for data collection/°	3.75 to 50.7
Index ranges	$-18 \le h \le 18, -24 \le k \le 24, -28 \le l \le 25$
Reflections collected	48026
Independent reflections	25536 [R_{int} = 0.0506, R_{sigma} = 0.1088]
Data/restraints/parameters	25536/458/1365
Goodness-of-fit on F^2	0.982
Final R indexes [$I \geq 2\sigma (I)$]	R_1 = 0.0983, wR_2 = 0.2760
Final R indexes [all data]	R_1 = 0.1560, wR_2 = 0.3162
Largest difference peak/hole/e Å^{-3}	1.14/–0.56
CCDC-number	2217238

Supplementary Table 15 Experimental single crystal X-ray diffraction data of **MeP5-MOF-1** collected at 105 K

Formula	[Zn_2(**MeP5BPy**)(**TPPE**)]
Empirical formula	$C_{35}H_{22}NO_4Zn$
Formula weight	585.9
Temperature/K	105
Crystal system	orthorhombic
Space group	*Pmmm*
a/Å	16.181(12)
b/Å	18.240(10)
c/Å	20.371(15)
α/°	90
β/°	90
γ/°	90
Volume/Å^3	6012(7)
Z	2
ρ_{calc}(g/cm^3)	0.324
μ/mm^{-1}	0.236
F(000)	602
Crystal size/mm^3	0.13 × 0.12 × 0.1
Radiation	GaKα (λ = 1.34139)
2θ range for data collection/°	4.752 to 107.812
Index ranges	$-19 \leq h \leq 19$, $-21 \leq k \leq 21$, $-24 \leq l \leq 24$
Reflections collected	100053
Independent reflections	6087 [R_{int} = 0.0749, R_{sigma} = 0.0315]
Data/restraints/parameters	6087/66/151
Goodness-of-fit on F^2	0.957
Final R indexes [$I \geq 2\sigma (I)$]	R_1 = 0.1298, wR_2 = 0.3036
Final R indexes [all data]	R_1 = 0.1402, wR_2 = 0.3135
Largest difference peak/hole/e Å^{-3}	1.28/–0.91
CCDC-number	2267727

Supplementary Table 16 Experimental single crystal X-ray diffraction data of **MeP5-MOF-1-G**

Formula	[Zn_2(**MeP5BPy**)(**TPPE**)]
Empirical formula	$C_{35}H_{22}NO_4Zn$
Formula weight	585.9
Temperature/K	193
Crystal system	orthorhombic
Space group	*Pmmm*
a/Å	16.143(3)
b/Å	18.713(5)
c/Å	20.483(3)
α/°	90
β/°	90
γ/°	90
Volume/Å^3	6187(2)
Z	2
ρ_{calc}(g/cm^3)	0.314
μ/mm^{-1}	0.35
F(000)	602
Crystal size/mm^3	0.13 × 0.12 × 0.1
Radiation	CuKα (λ = 1.54178)
2θ range for data collection/°	4.722 to 136.472
Index ranges	$-11 \leq h \leq 19$, $-19 \leq k \leq 22$, $-20 \leq l \leq 24$
Reflections collected	34982
Independent reflections	6097 [R_{int} = 0.1780, R_{sigma} = 0.1153]
Data/restraints/parameters	6097/181/145
Goodness-of-fit on F^2	1.037
Final R indexes [$I \geq 2\sigma (I)$]	R_1 = 0.1476, wR_2 = 0.3305
Final R indexes [all data]	R_1 = 0.1878, wR_2 = 0.3522
Largest difference peak/hole/e Å^{-3}	1.31/–1.10
CCDC-number	2267730

Responses to B level check cif alerts for $(DMF)_2$@**Model-MOF-1** crystal structure:

Alert Level B

PLAT341_ALERT_3_B Low Bond Precision on C−C Bonds .... 0.01834 Ang.

Author Response: Due to poor crystal quality and very weak diffraction.

Responses to B level check cif alerts for **MeP5-MOF-2** crystal structure:

Alert Level B

PLAT220_ALERT_2_B NonSolvent Resd 1 C Ueq(max)/Ueq(min) Range 7.2 Ratio.

Author Response: This Level B alert arises because the macrocycle repeating units undergo fast rotation even though the data were collected at 193 K.

Responses to B level check cif alerts for $(DMF)_3$@**MeP5-MOF-2** crystal structure:

Alert Level B

PLAT973_ALERT_2_B Check Calcd Positive Resid. Density on Zn1 1.73 e $Å^{-3}$

Author Response: Such residual density may be an effect of twinning (that has not been identified) or problems related to absorption.

Responses to B level check cif alerts for **Model-MOF-2** crystal structure:

Alert Level B

PLAT026_ALERT_3_B Ratio Observed/Unique Reflections (too) Low.. 32% Check

Author Response: The low ratio of observed/unique reflections is due to the low electron dose and short exposure time used for data collection in order to minimize the beam damage.

Alert Level B

PLAT242_ALERT_2_B Low 'MainMol' Ueq as Compared to Neighbors of Zn2 Check

Author Response: As one of the central atoms of the secondary building unit (inorganic Zn_2O_4 cluster), the structure tends to be rigid compared to the surrounding ligands.

Alert Level B

PLAT420_ALERT_2_B D-H Bond Without Acceptor O9 --H9A . Please Check

PLAT420_ALERT_2_B D-H Bond Without Acceptor O9 --H9B . Please Check

PLAT420_ALERT_2_B D-H Bond Without Acceptor O10 --H10A . Please Check

PLAT420_ALERT_2_B D-H Bond Without Acceptor O10 --H10B . Please Check

Author Response: Possibly a potential hydrogen bond to the unmodelled solvent, or the possible absence of a hydrogen bond for this coordinated water molecule.

Responses to B level check cif alerts for *pR*-**MeP5-MOF-2** crystal structure:

Alert Level B

PLAT220_ALERT_2_B NonSolvent Resd 1 C Ueq(max)/Ueq(min) Range 9.8 Ratio

Author Response: This Level B alert arises because the macrocycle repeating units undergo fast rotation even though the data were collected at 193 K.

Alert Level B

PLAT220_ALERT_2_B NonSolvent Resd 1 O Ueq(max)/Ueq(min) Range 6.6 Ratio

Author Response: This Level B alert arises because the macrocycle repeating units undergo fast rotation even though the data were collected at 193 K.

Responses to B level check cif alerts for **MeP5-MOF-4** crystal structure:

Alert Level B

PLAT971_ALERT_2_B Check Calcd Resid. Dens. 0.91Ang From N2 2.60 e Å^{-3}

Author Response: There is no chemically sensible species corresponding to this residual density peak. The electron density appears to be around phenyl ring, such residual density is likely not from unaccounted atom types.

Responses to B level check cif alerts for **Tol@MeP5-MOF-2** crystal structure:

Alert Level B

PLAT910_ALERT_3_B Missing # of FCF Reflection(s) Below Theta(Min). 12 Note

Author Response: Low angle reflections are probably missing due to the beamstop.

7. Crystal structure analysis

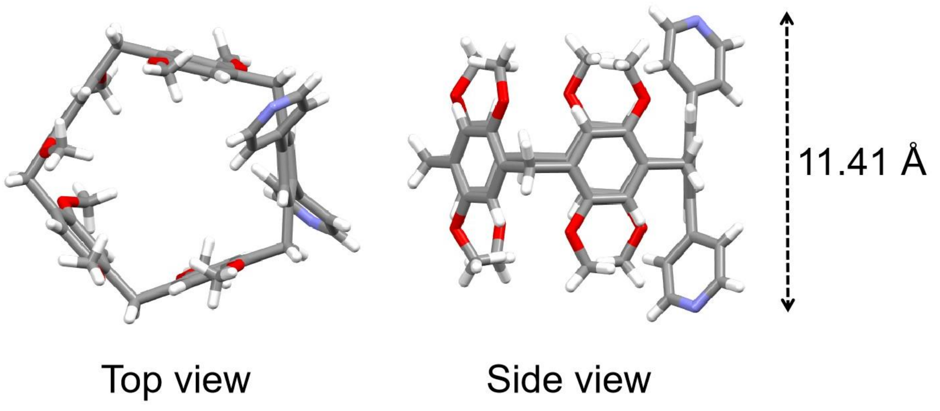

Supplementary Fig. 24 Capped-stick representation of the single crystal structure of **MeP5BPy**. Carbon atoms are grey, hydrogen atoms are white, oxygen atoms are red, and nitrogen atoms are blue. The CCDC number of **MeP5BPy** is 2096367 according to a previous report[S2].

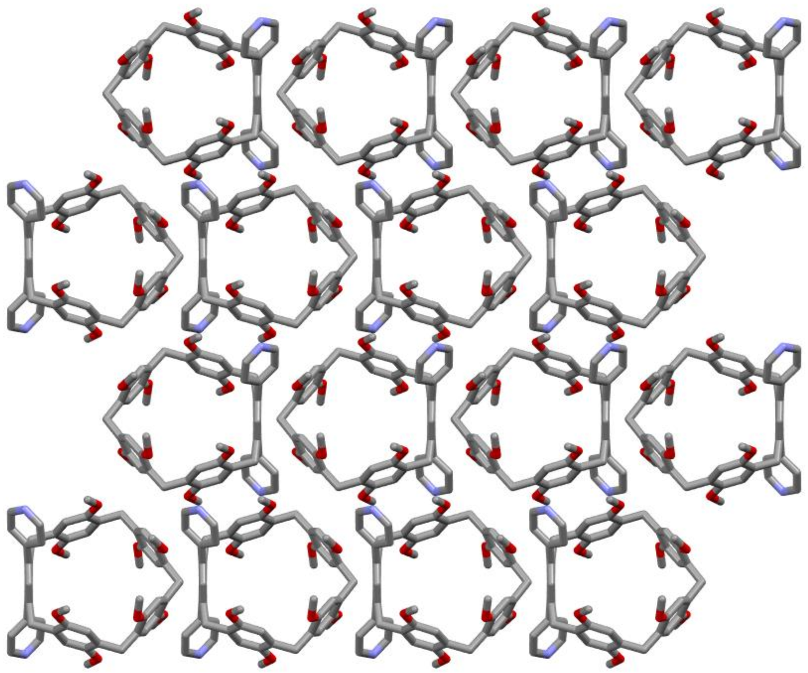

Supplementary Fig. 25 Capped-stick representation of the single crystal structure of **MeP5BPy** showing the packing arrangement. Carbon atoms are grey, oxygen atoms are red, and nitrogen atoms are blue. Hydrogen atoms are omitted for clarity. The CCDC number of **MeP5BPy** is 2096367 according to a previous report[S2].

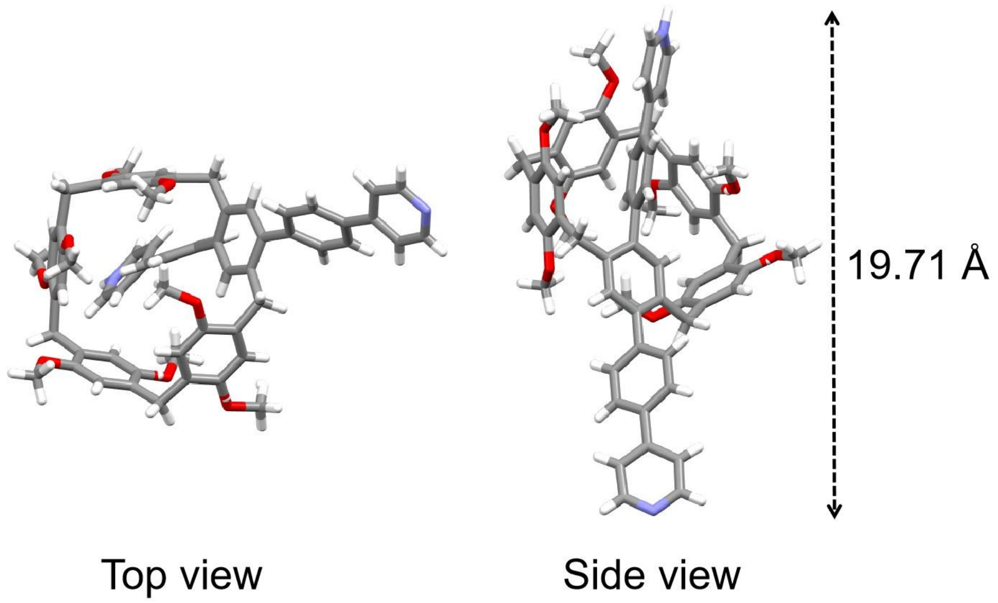

Supplementary Fig. 26 Capped-stick representation of the single crystal structure of **MeP5BPPy**. Carbon atoms are grey, hydrogen atoms are white, oxygen atoms are red, and nitrogen atoms are blue.

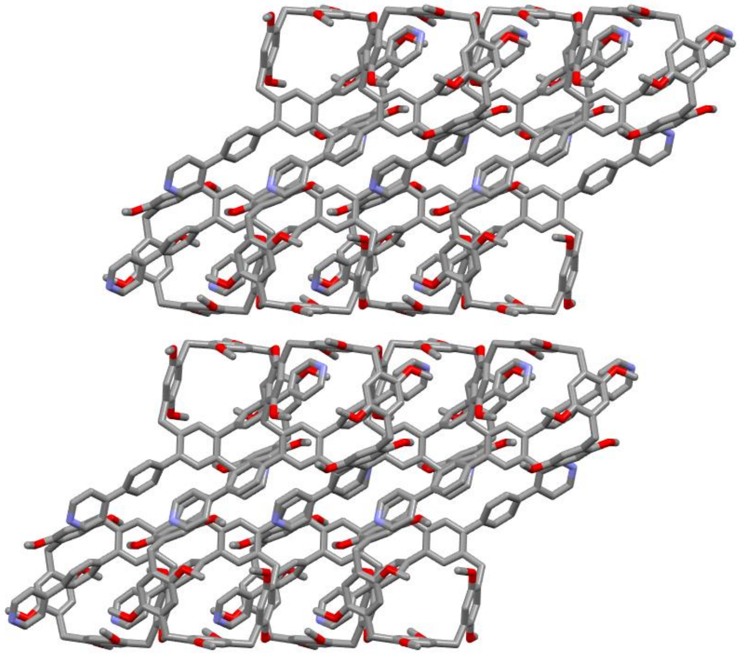

Supplementary Fig. 27 Capped-stick representation of the single crystal structure of **MeP5BPPy** showing the packing arrangement. Carbon atoms are grey, oxygen atoms are red, and nitrogen atoms are blue. Hydrogen atoms are omitted for clarity.

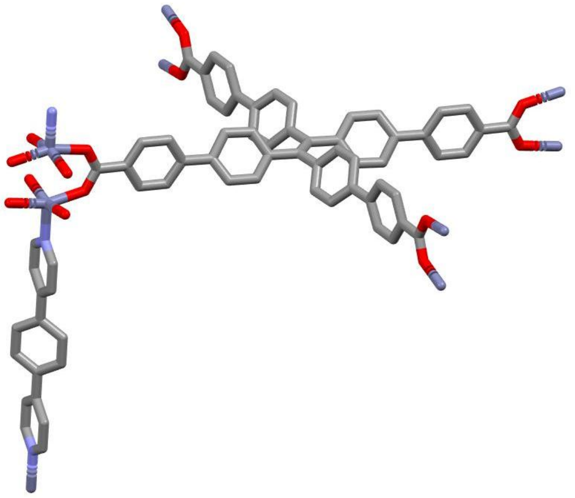

Supplementary Fig. 28 Capped-stick representation of the single crystal structure of **MeP5-MOF-1**. The pillar[5]arene units on the pillared struts are disordered and not resolved. Carbon atoms are grey, oxygen atoms are red, nitrogen atoms are blue, and zinc atoms are dark blue. Hydrogen atoms are omitted for clarity.

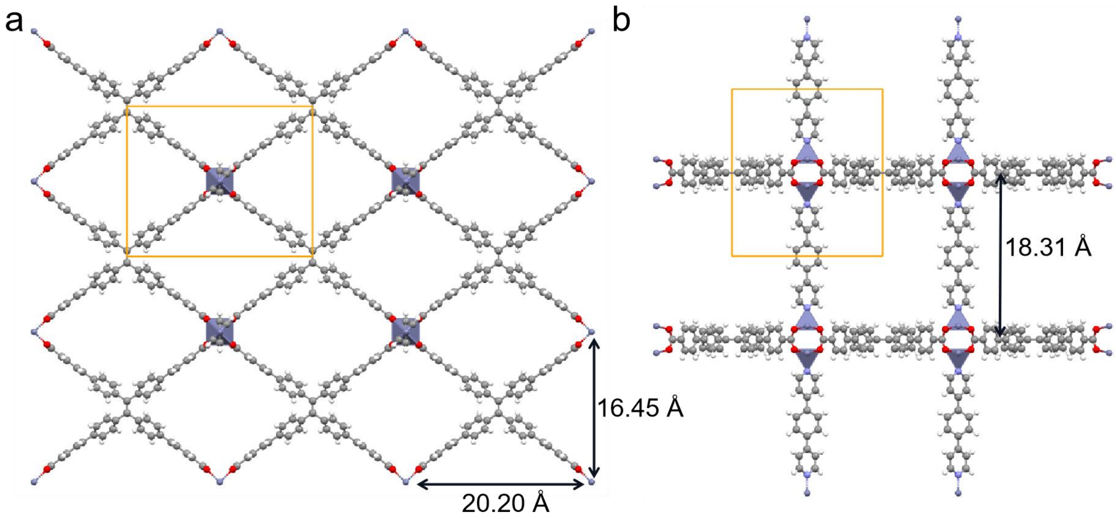

Supplementary Fig. 29 Polyhedral representation of the single crystal structure of **MeP5-MOF-1** along the *c* axis (a) and the *b* axis (b). The elementary cell is marked with an orange cuboid (a = 16.45 Å, b = 20.20 Å, c = 18.31 Å). The pillar[5]arene units on the pillared struts are disordered and not resolved. Carbon atoms are grey, hydrogen atoms are white, oxygen atoms are red, nitrogen atoms are blue, and zinc atoms are dark blue.

In order to get the whole structure of **MeP5-MOF-1**, a calculated structure named **MeP5-MOF-1a** was constructed. **MeP5-MOF-1** was used as the starting model for building the backbone of **MeP5-MOF-1a**. The pillar[5]arene units were illustrated based on structure modeling with a *Pmmm* space group, devoid of any solvent molecules. We then incorporated the pillar[5]arene units into the backbone linkers using the Materials Studio program[S9], keeping the rest of the atoms and unit cell dimensions unchanged. Thereafter, the DMol^3 module was subject to geometry optimization based on molecular mechanics calculations, modifying the atomic positions, as well as the lattice parameters. To get a stable configuration for **MeP5-MOF-1a**, the DMol^3 module was utilized to provide for geometry optimization based on molecular mechanics calculations. The final predicted structure for **MeP5-MOF-1**. Note that in the resulting structural model, the orientations of the pillar[5]arene rings are uniformly distributed. The PXRD pattern of the calcultaed structure of **MeP5-MOF-1a** matched well with that from a experimental single crystal sample.

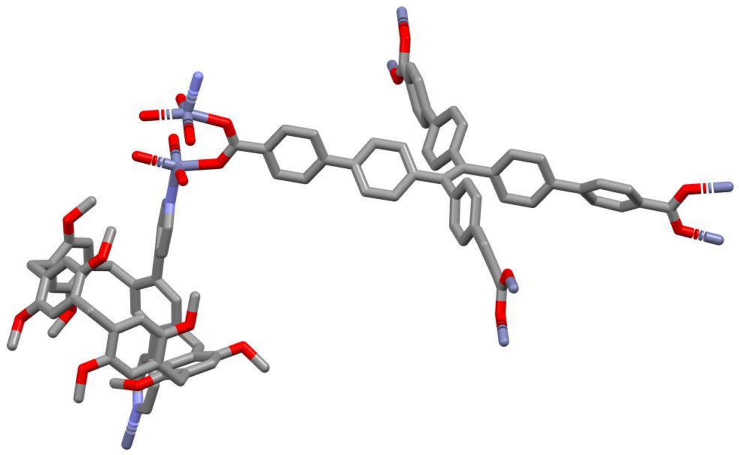

Supplementary Fig. 30 Capped-stick representation of the calculated structure **MeP5-MOF-1a** based on the backbone of **MeP5-MOF-1**. The pillar[5]arene units on the struts are shown. Carbon atoms are grey, oxygen atoms are red, nitrogen atoms are blue, and zinc atoms are dark blue. Hydrogen atoms are omitted for clarity.

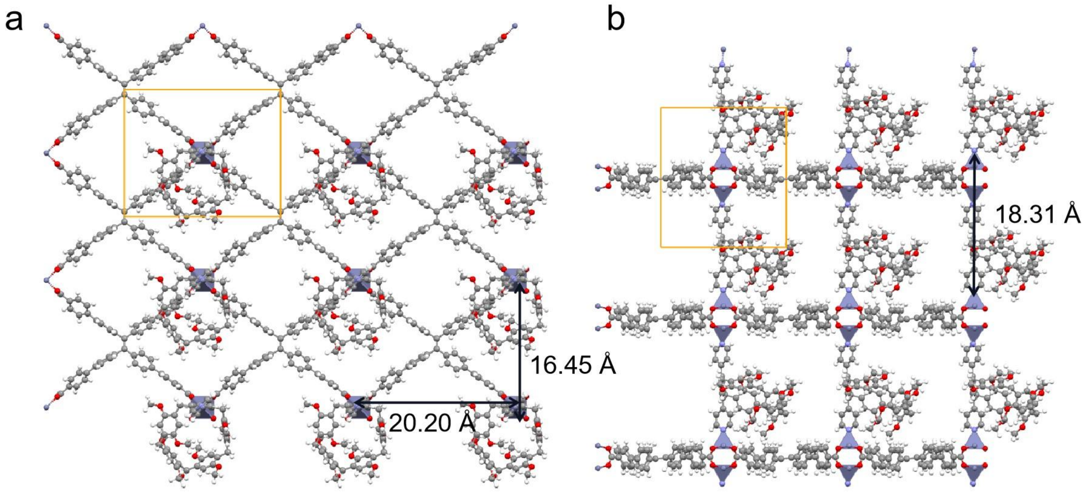

Supplementary Fig. 31 Polyhedral representation of the calculated structure **MeP5-MOF-1a** along the *c* axis (a) and the *b* axis (b). The elementary cell is marked with an orange cuboid (a = 16.45 Å, b = 20.20 Å, c = 18.31 Å). Carbon atoms are grey, hydrogen atoms are white, oxygen atoms are red, nitrogen atoms are blue, and zinc atoms are dark blue.

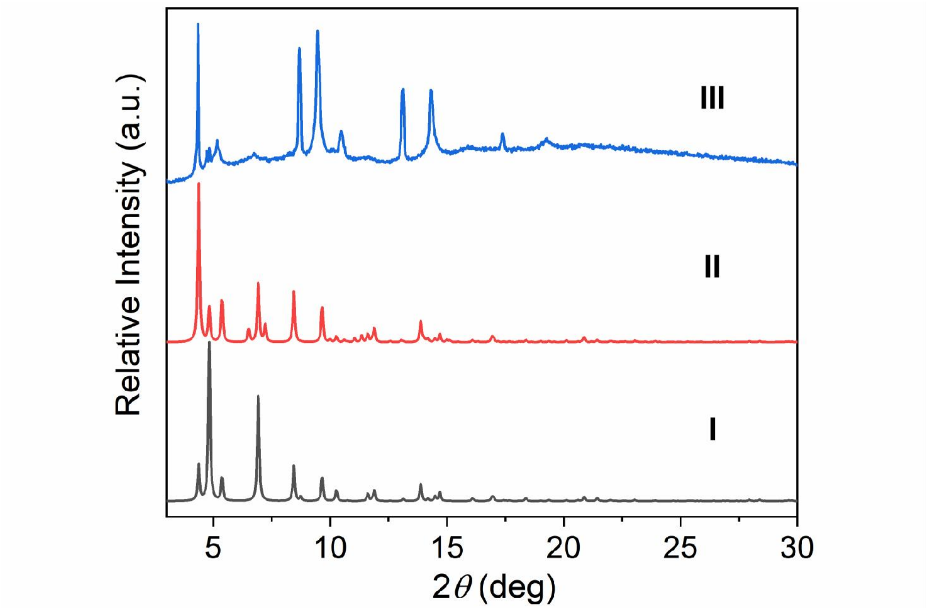

Supplementary Fig. 32 PXRD patterns: **I**, simulated from the single crystal structure of **MeP5-MOF-1**; **II**, simulated from the calculated structure of **MeP5-MOF-1a**; **III**, **MeP5-MOF-1** from a single crystal sample.

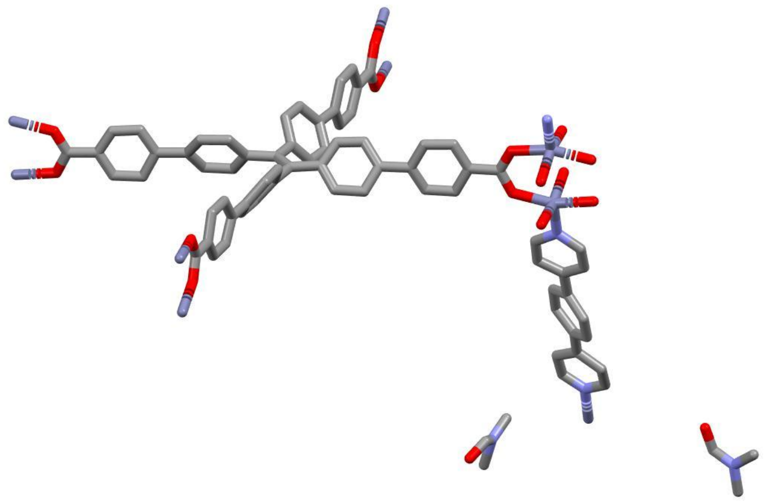

Supplementary Fig. 33 Capped-stick representation of the single crystal structure of $(DMF)_2$@**Model-MOF-1**. Carbon atoms are grey, oxygen atoms are red, nitrogen atoms are blue, and zinc atoms are dark blue. Hydrogen atoms are omitted for clarity.

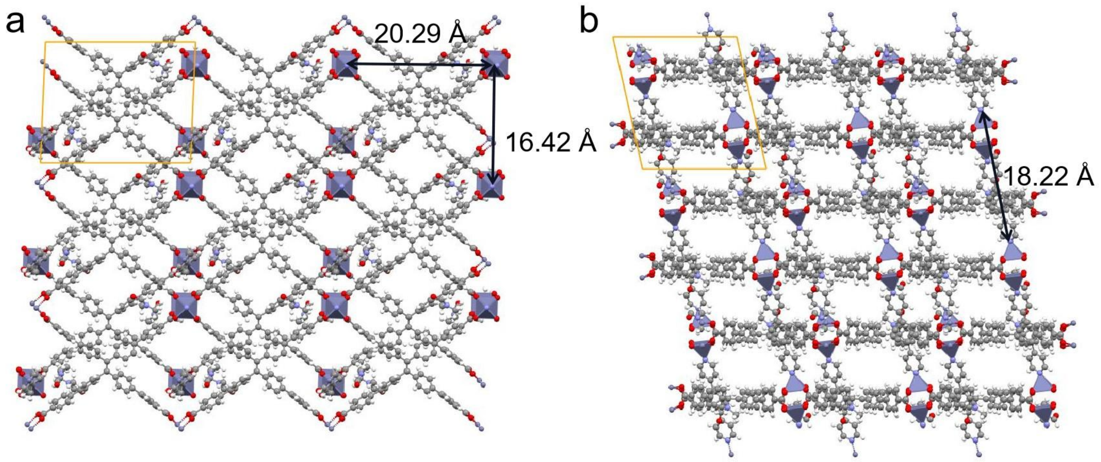

Supplementary Fig. 34 Polyhedral representation of the single crystal structure of $(DMF)_2$@**Model-MOF-1** along the *c* axis (a) and the *b* axis (b). The elementary cell is marked with an orange cuboid (*a* = 16.42 Å, *b* = 20.29 Å, *c* = 18.22 Å). Carbon atoms are grey, hydrogen atoms are white, oxygen atoms are red, nitrogen atoms are blue, and zinc atoms are dark blue.

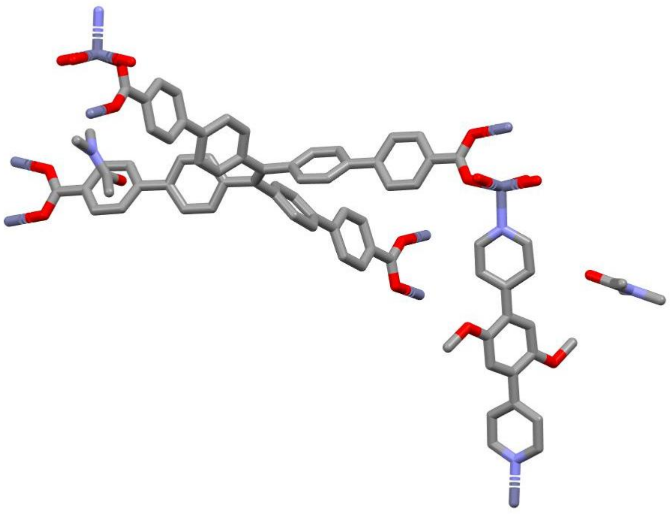

Supplementary Fig. 35 Capped-stick representation of the single crystal structure of $(DMA)_2$@**MeModel-MOF-1**. Carbon atoms are grey, oxygen atoms are red, nitrogen atoms are blue, and zinc atoms are dark blue. Hydrogen atoms are omitted for clarity.

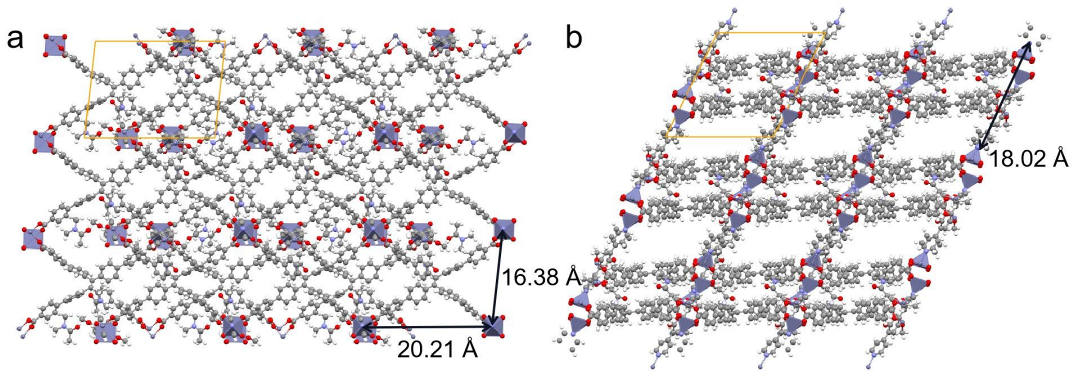

Supplementary Fig. 36 Polyhedral representation of the single crystal structure of $(DMA)_2$@**MeModel-MOF-1** along the *c* axis (a) and the *b* axis (b). The elementary cell is marked with an orange cuboid (*a* = 16.38 Å, *b* = 20.21 Å, *c* = 18.02 Å). Carbon atoms are grey, hydrogen atoms are white, oxygen atoms are red, nitrogen atoms are blue, and zinc atoms are dark blue.

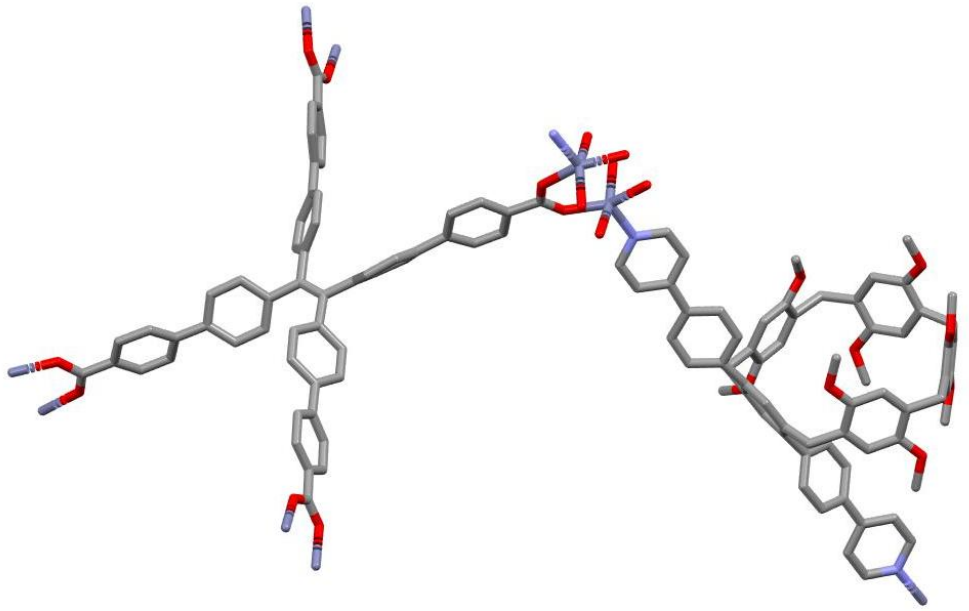

Supplementary Fig. 37 Capped-stick representation of the single crystal structure of **MeP5-MOF-2**. Carbon atoms are grey, oxygen atoms are red, nitrogen atoms are blue, and zinc atoms are dark blue. Hydrogen atoms are omitted for clarity.

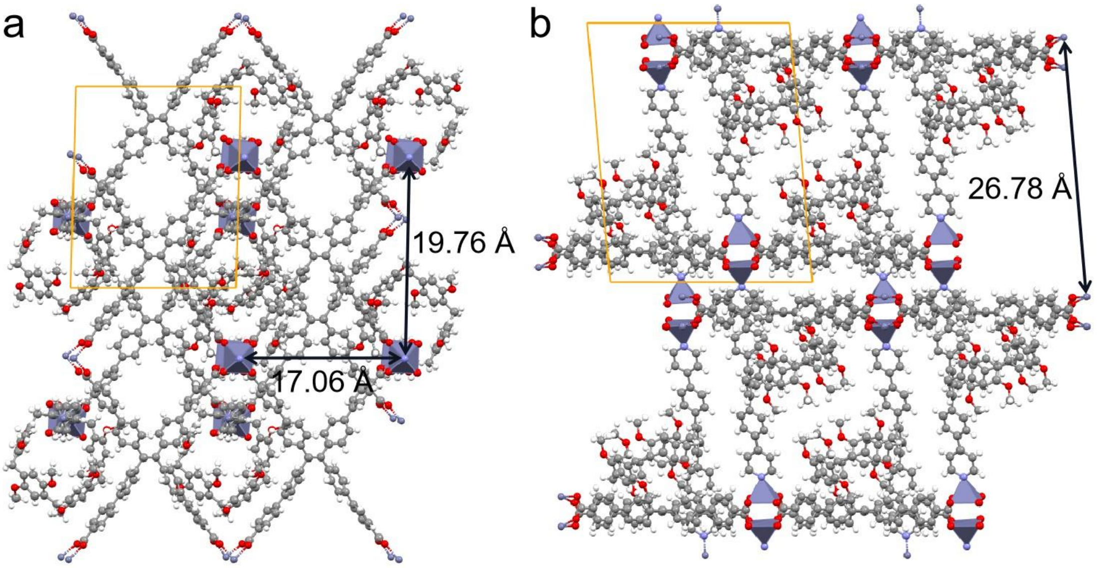

Supplementary Fig. 38 Polyhedral representation of the single crystal structure of **MeP5-MOF-2** along the *a* axis (a) and the *c* axis (b). The elementary cell is marked with an orange cuboid (*a* = 19.76 Å, *b* = 17.06 Å, *c* = 26.78 Å). Carbon atoms are grey, hydrogen atoms are white, oxygen atoms are red, nitrogen atoms are blue, and zinc atoms are dark blue.

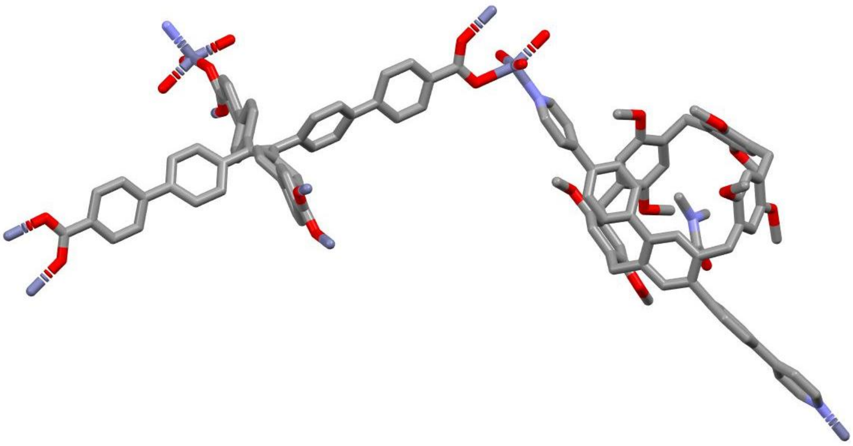

Supplementary Fig. 39 Capped-stick representation of the single crystal structure of DMF@**MeP5-MOF-2**. Carbon atoms are grey, oxygen atoms are red, nitrogen atoms are blue, and zinc atoms are dark blue. Hydrogen atoms are omitted for clarity.

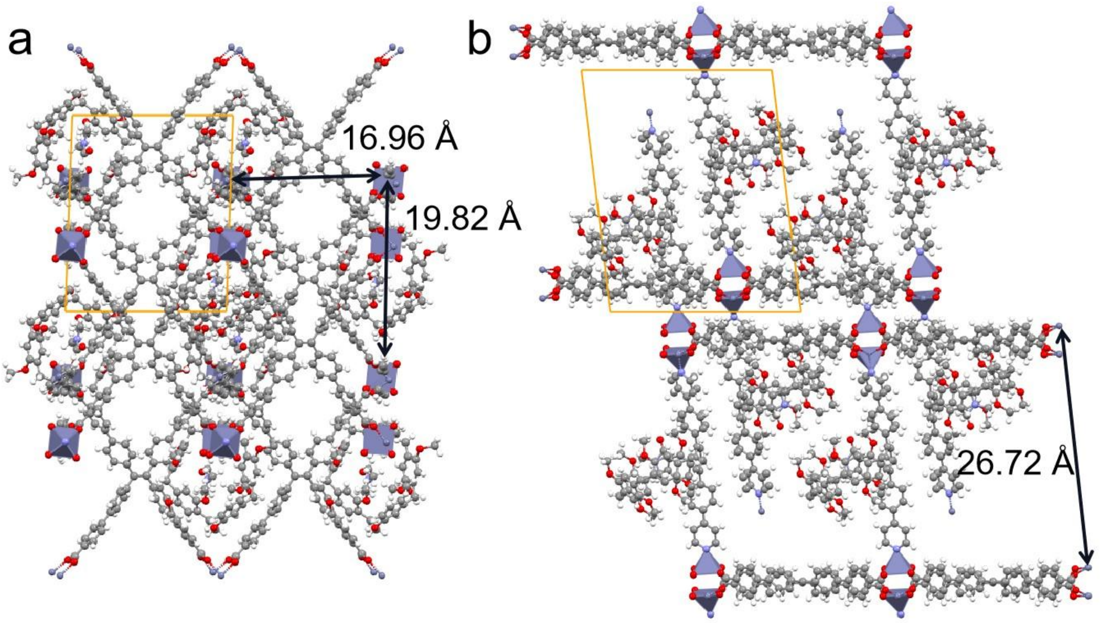

Supplementary Fig. 40 Polyhedral representation of the single crystal structure of DMF@**MeP5-MOF-2** along the *a* axis (a) and the *c* axis (b). The elementary cell is marked with an orange cuboid (*a* = 19.82 Å, *b* = 16.96 Å, *c* = 26.72 Å). Carbon atoms are grey, hydrogen atoms are white, oxygen atoms are red, nitrogen atoms are blue, and zinc atoms are dark blue.

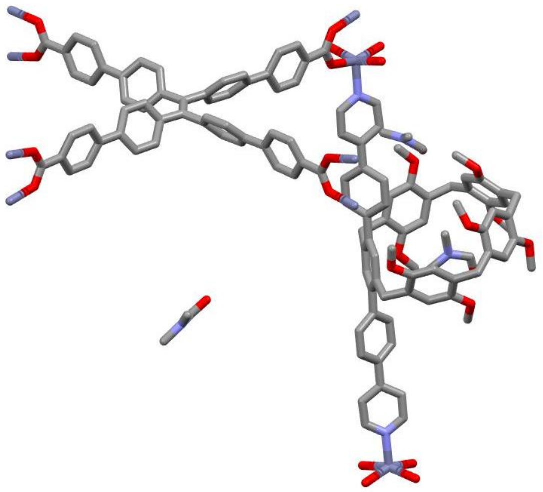

Supplementary Fig. 41 Capped-stick representation of the single crystal structure of $(DMF)_3$@**MeP5-MOF-2**. Carbon atoms are grey, oxygen atoms are red, nitrogen atoms are blue, and zinc atoms are dark blue. Hydrogen atoms are omitted for clarity.

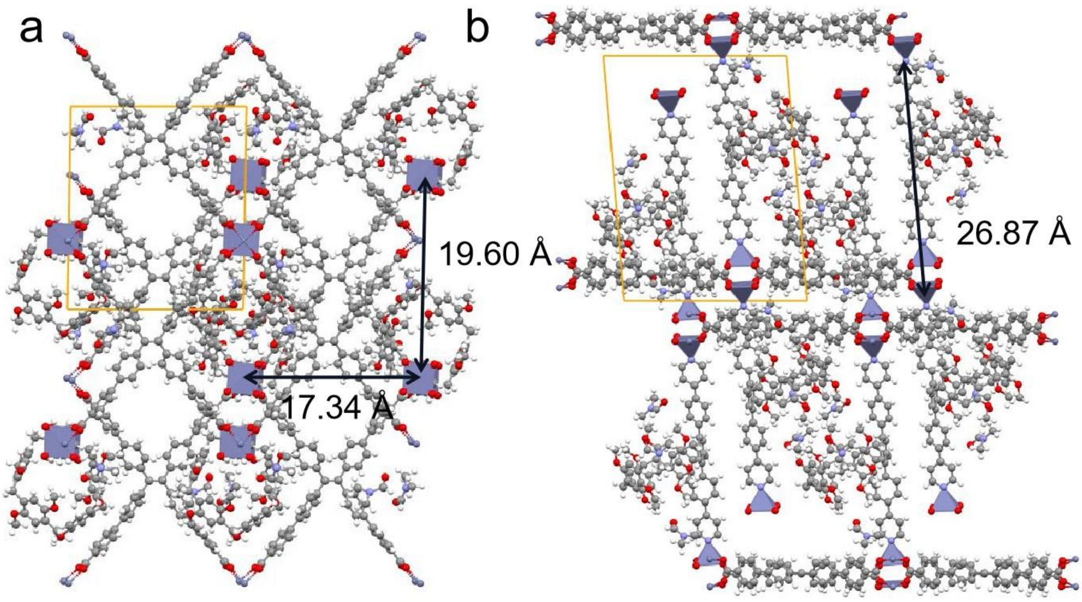

Supplementary Fig. 42 Polyhedral representation of the single crystal structure of $(DMF)_3$@**MeP5-MOF-2** along the *a* axis (a) and the *c* axis (b). The elementary cell is marked with an orange cuboid (a = 19.60 Å, b = 17.34 Å, c = 26.87 Å). Carbon atoms are grey, hydrogen atoms are white, oxygen atoms are red, nitrogen atoms are blue, and zinc atoms are dark blue.

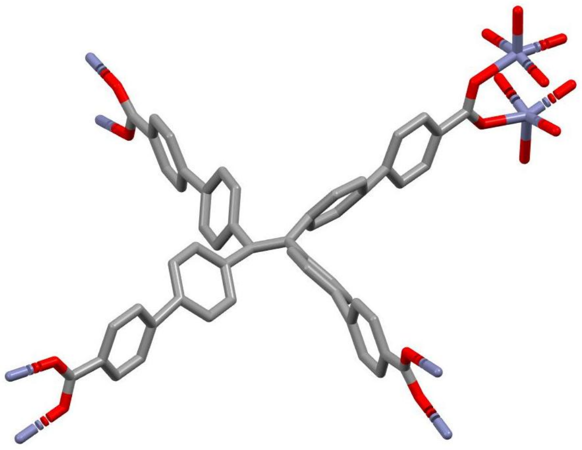

Supplementary Fig. 43 Capped-stick representation of the single crystal structure of **Model-MOF-2**. Carbon atoms are grey, oxygen atoms are red, and zinc atoms are dark blue. Hydrogen atoms are omitted for clarity. The crystal structure of **Model-MOF-2** was based on SCXRD of a single crystal sample obtained in the preparation of **MeP5-MOF-2**, which was composed of **TPPE** and terminated by H_2O molecules in the direction of pillared struts. This finding likely reflects the presence of excess H_2O molecules during the preparation.

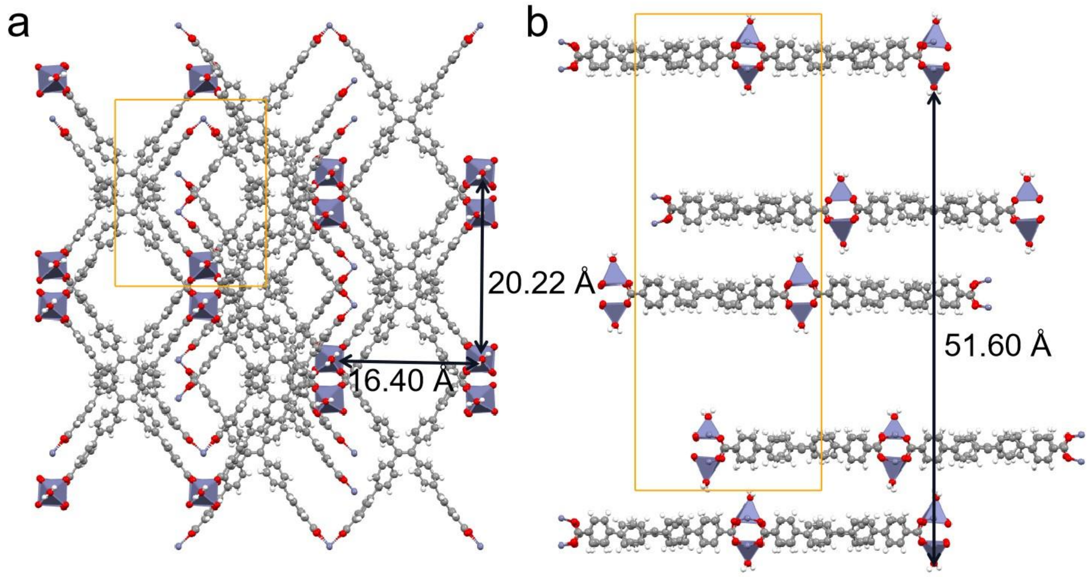

Supplementary Fig. 44 Polyhedral representation of the single crystal structure of **Model-MOF-2** along the *a* axis (a) and the *b* axis (b). The elementary cell is marked with an orange cuboid (*a* = 20.22 Å, *b* = 16.40 Å, *c* = 51.60 Å). Carbon atoms are grey, hydrogen atoms are white, oxygen atoms are red, and zinc atoms are dark blue.

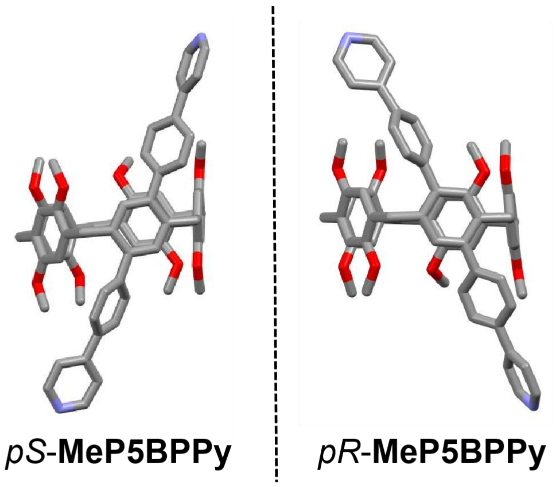

Supplementary Fig. 45 Capped-stick representation of the single crystal structures of *pS*-**MeP5BPPy** and *pR*-**MeP5BPPy**, showing the planar chirality of pillar[5]arenes. Carbon atoms are grey, oxygen atoms are red, and nitrogen atoms are blue. Hydrogen atoms are omitted for clarity.

Determination of the absolute conformations of **MeP5BPPy**: The resolution of *racemic*-**MeP5BPPy** was performed *via* chiral HPLC. The chromatogram after

resolution is shown below. Optical rotation: *pR*-**MeP5BPPy** $[\alpha]_D^{20} = -70^0$, *pS*-**MeP5BPPy** $[\alpha]_D^{20} = +69^0$ (in dichloromethane, concentration = 0.05 g/100 mL, tube length = 1.00 dm).

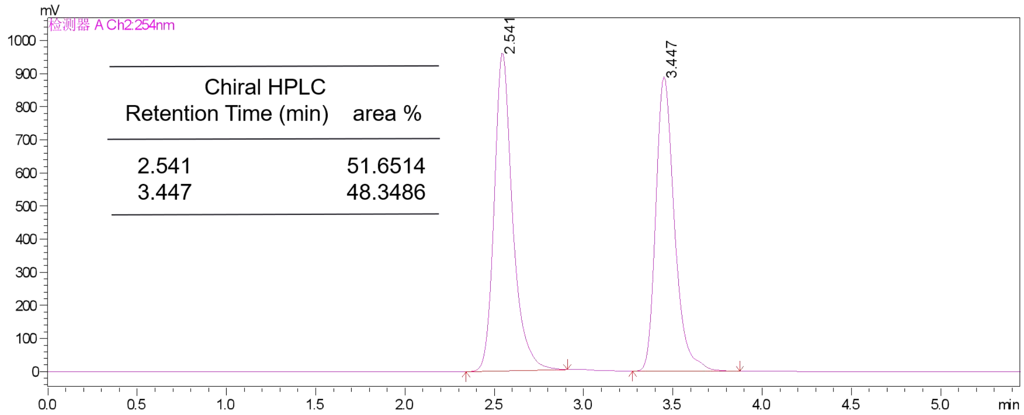

Supplementary Fig. 46 Chromatogram of *racemic*-**MeP5BPPy**.

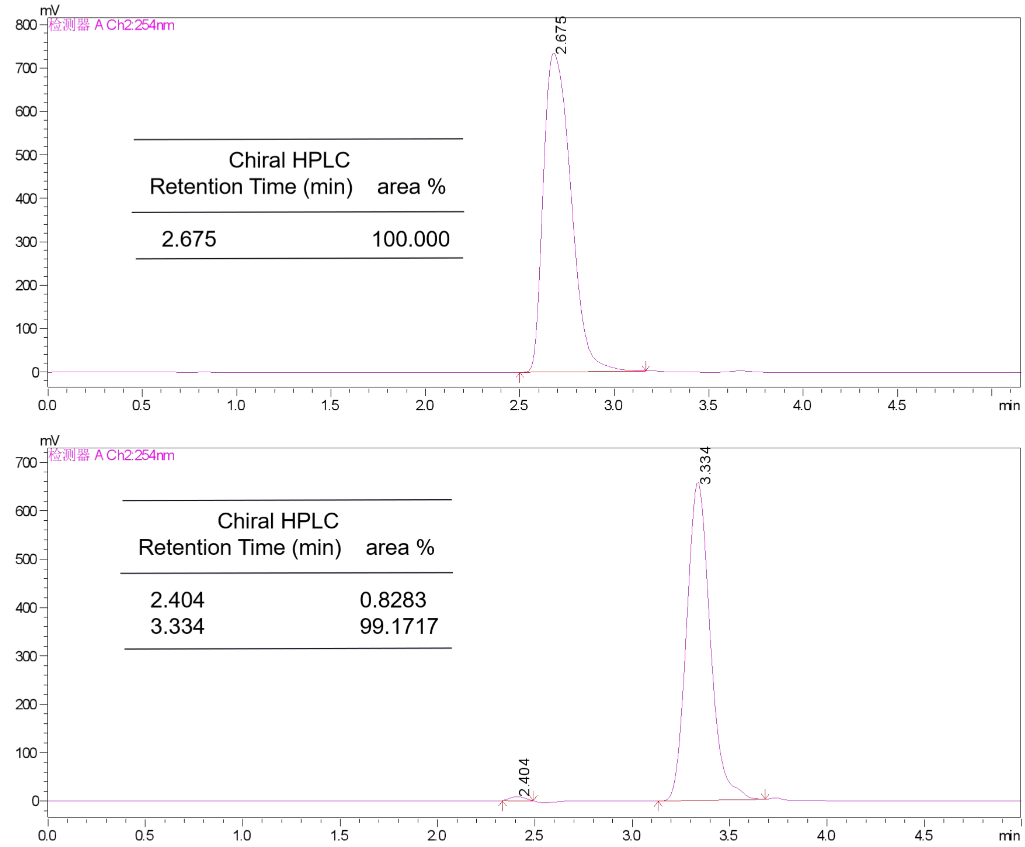

Supplementary Fig. 47 Chromatograms of *pS*-**MeP5BPPy** (top) and *pR*-**MeP5BPPy** (bottom). ee = 100% (*pS*-**MeP5BPPy**); ee = 98% (*pR*-**MeP5BPPy**).

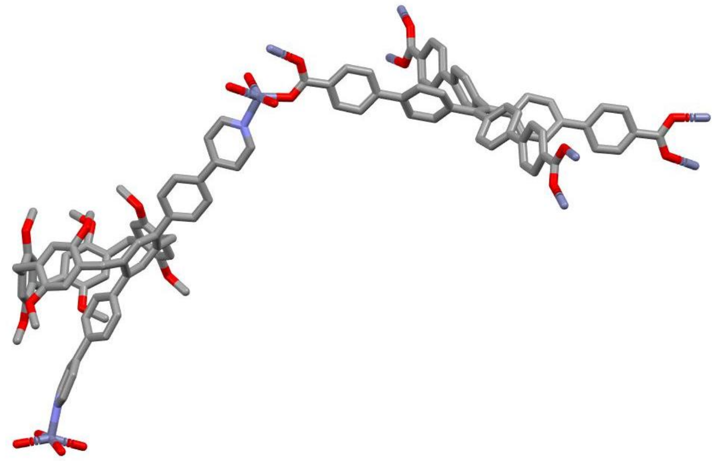

Supplementary Fig. 48 Capped-stick representation of the single crystal structure of *pS*-**MeP5-MOF-2**. Carbon atoms are grey, oxygen atoms are red, nitrogen atoms are blue, and zinc atoms are dark blue. Hydrogen atoms are omitted for clarity.

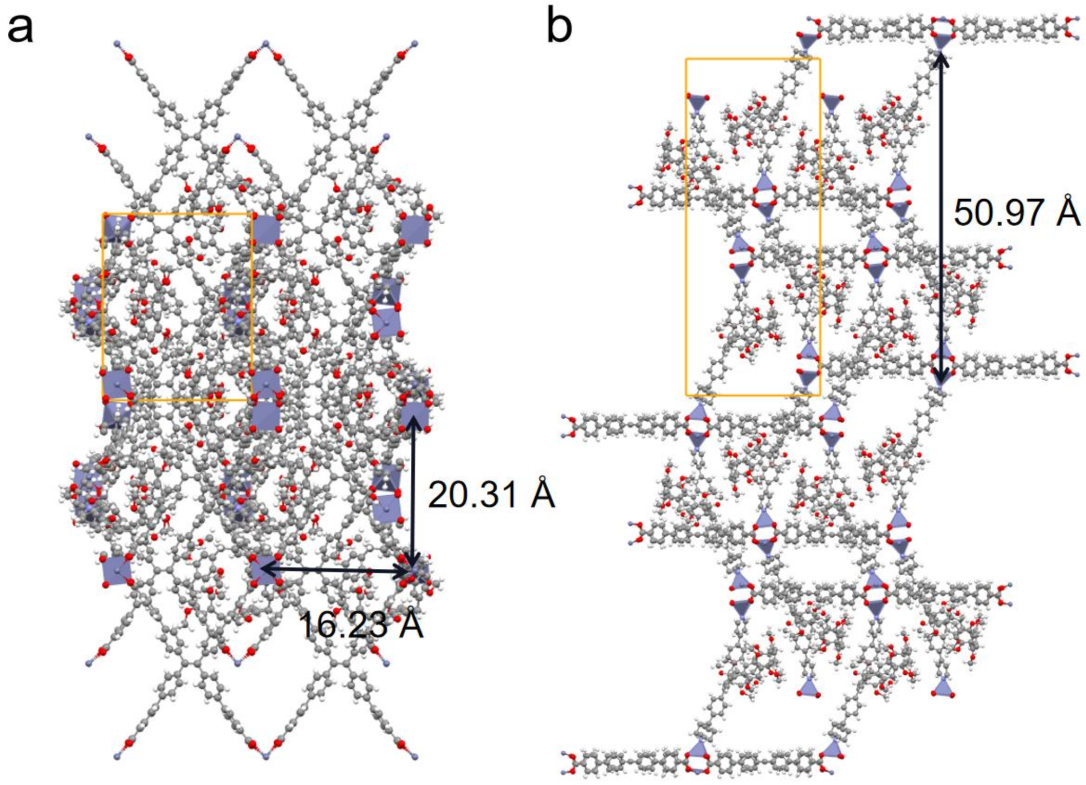

Supplementary Fig. 49 Polyhedral representation of the single crystal structure of *pS*-**MeP5-MOF-2** along the *a* axis (a) and the *c* axis (b). The elementary cell is marked with an orange cuboid (a = 20.31 Å, b = 16.23 Å, c = 50.97 Å). Carbon atoms are grey, hydrogen atoms are white, oxygen atoms are red, nitrogen atoms are blue, and zinc atoms are dark blue.

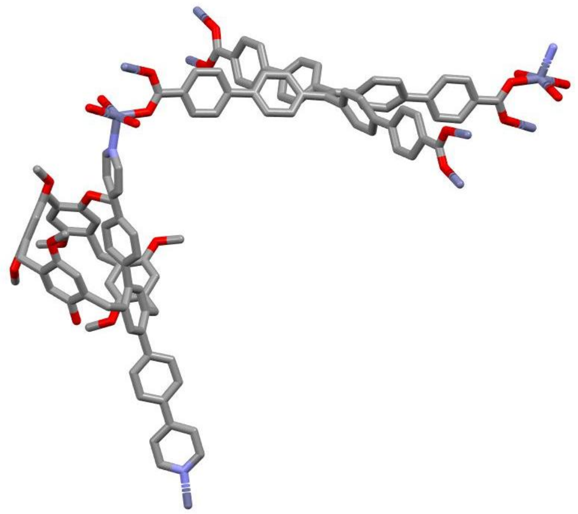

Supplementary Fig. 50 Capped-stick representation of the single crystal structure of *pR*-**MeP5-MOF-2**. Carbon atoms are grey, oxygen atoms are red, nitrogen atoms are blue, and zinc atoms are dark blue. Hydrogen atoms are omitted for clarity.

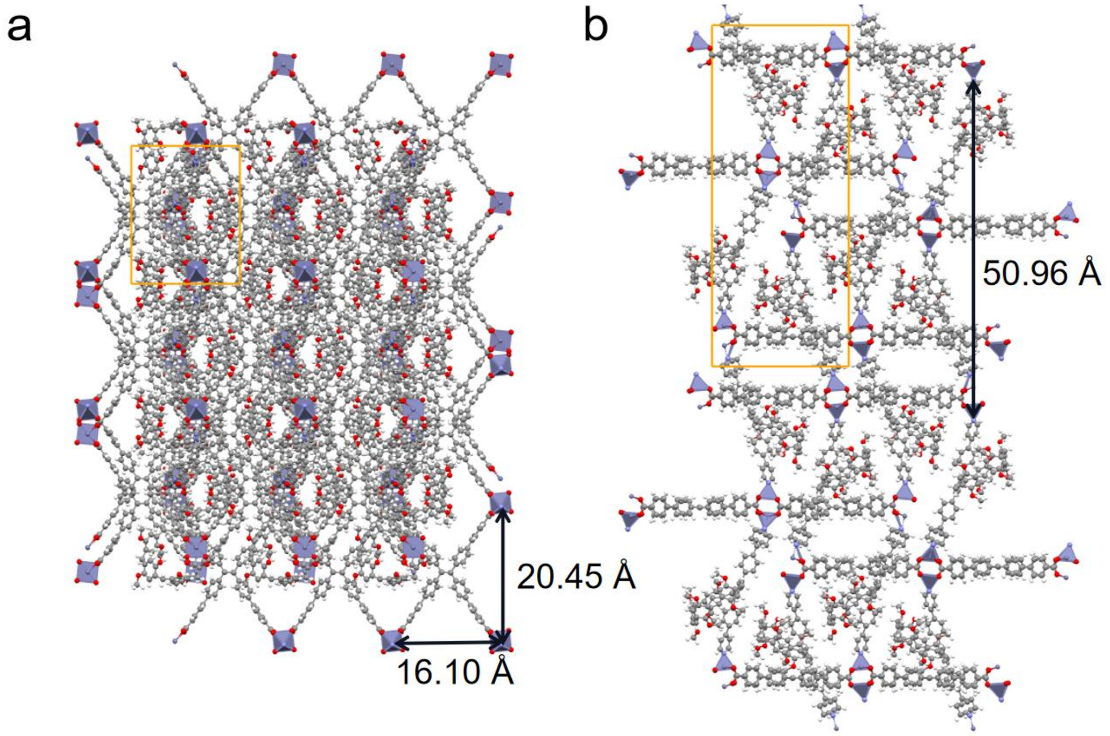

Supplementary Fig. 51 Polyhedral representation of the single crystal structure of *pR*-**MeP5-MOF-2** along the *a* axis (a) and the *c* axis (b). The elementary cell is marked with an orange cuboid (a = 20.45 Å, b = 16.10 Å, c = 50.96 Å). Carbon atoms are grey, hydrogen atoms are white, oxygen atoms are red, nitrogen atoms are blue, and zinc atoms are dark blue.

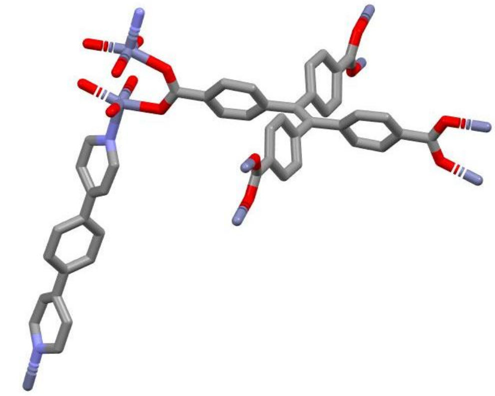

Supplementary Fig. 52 Capped-stick representation of the single crystal structure of **MeP5-MOF-3**. The pillar[5]arene units on the pillared struts are disordered and not resolved. Carbon atoms are grey, oxygen atoms are red, nitrogen atoms are blue, and zinc atoms are dark blue. Hydrogen atoms are omitted for clarity.

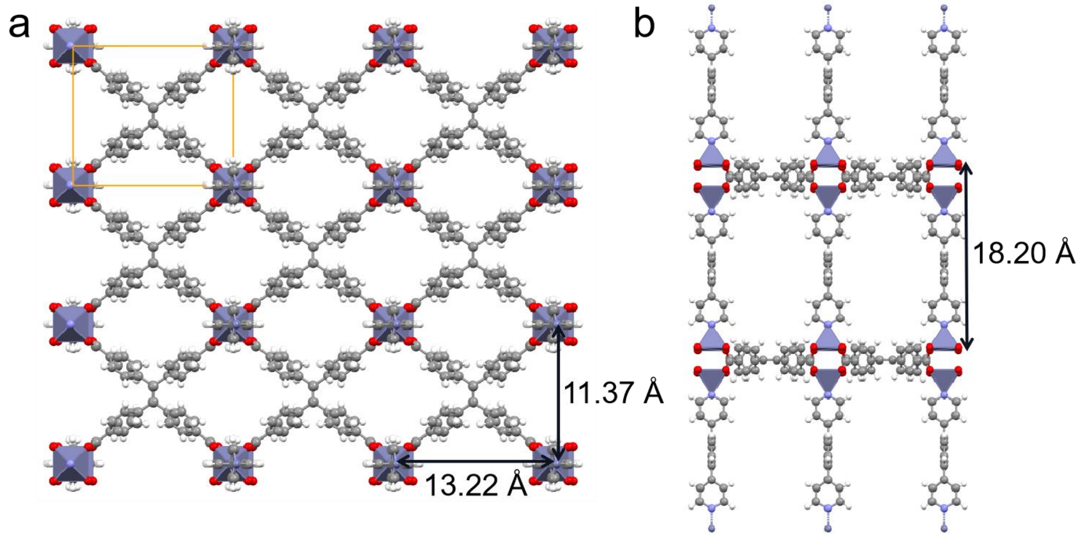

Supplementary Fig. 53 Polyhedral representation of the single crystal structure of **MeP5-MOF-3** along the *c* axis (a) and the *b* axis (b). The elementary cell is marked with an orange cuboid (a = 11.37 Å, b = 13.22 Å, c = 18.20 Å). The pillar[5]arene units on the pillared struts are disordered and not resolved. Carbon atoms are grey, hydrogen atoms are white, oxygen atoms are red, nitrogen atoms are blue, and zinc atoms are dark blue.

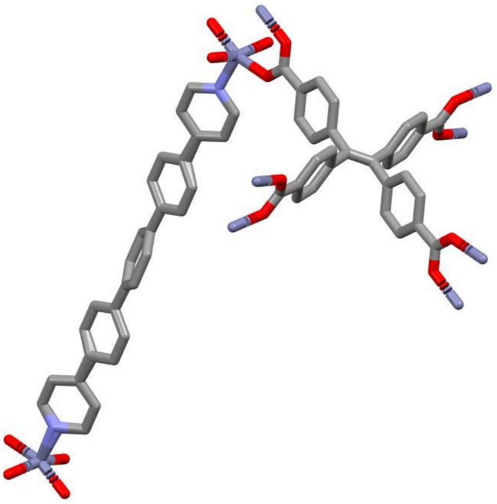

Supplementary Fig. 54 Capped-stick representation of the single crystal structure of **MeP5-MOF-4**. The pillar[5]arene units on the pillared struts are disordered and not resolved. Carbon atoms are grey, oxygen atoms are red, nitrogen atoms are blue, and zinc atoms are dark blue. Hydrogen atoms are omitted for clarity.

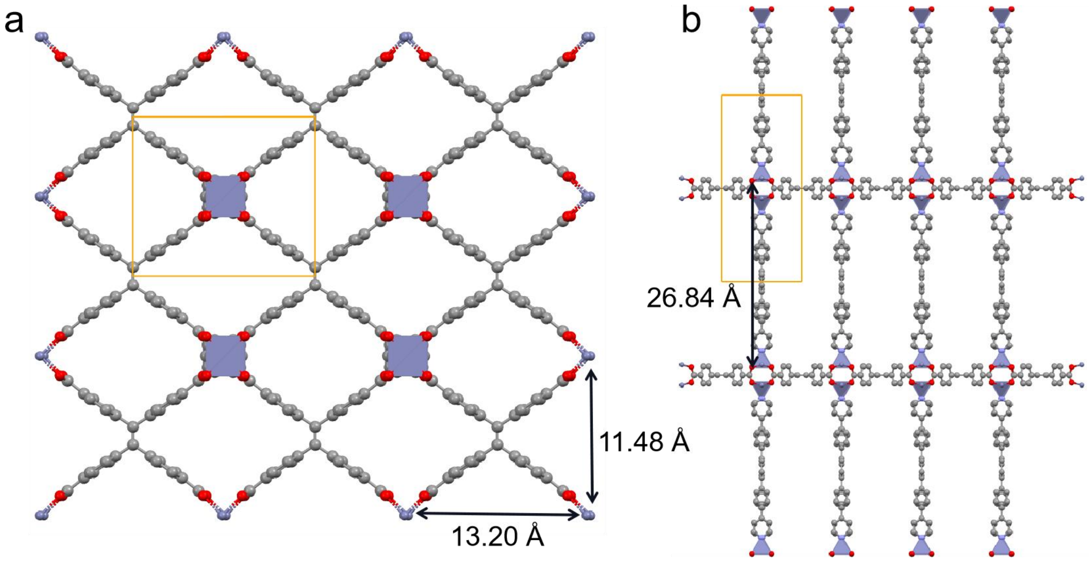

Supplementary Fig. 55 Polyhedral representation of the single crystal structure of **MeP5-MOF-4** along the *c* axis (a) and the *b* axis (b). The pillar[5]arene units on the pillared struts are disordered and not resolved. The elementary cell is marked with orange cuboid (a = 11.48 Å, b = 13.20 Å, c = 26.84 Å). Carbon atoms are grey, oxygen atoms are red, nitrogen atoms are blue, and zinc atoms are dark blue. Hydrogen atoms are omitted for clarity.

Dynamic analysis of MeP5-MOF-1 and MeP5-MOF-2

In order to obtain insights into the dynamics of the pillar[5]arene units in **MeP5-MOF-1** and **MeP5-MOF-2** frameworks, the number of pillar[5]arene unit equivalents in a 1 nm^3 volume element were calculated for the single crystal strutures[S10]. The single crystal structures of the struts **MeP5BPy** and **MeP5BPPy** are characterized by 0.80 and 0.76 pillar[5]arene unit per this 1 nm^3 volume element, respectively. In contrast, **MeP5-MOF-1** has only 0.16 pillar[5]arene unit per 1 nm^3 volume element, while the corresponding value for **MeP5-MOF-2** is 0.23. Note: the CCDC number of **MeP5BPy** is 2096367 per a previous report[S2].

Supplementary Table 17 Density calculation of pillar[5]arene units

Substance	Number of pillar[5]arene units per unit cell	Volume of unit cell (nm^3)	Number of pillar[5]arene units per cubic nanometer (nm^{-3})
**MeP5BPy**	4	4.99	0.80
**MeP5-MOF-1**	1	6.08	0.16
**MeP5BPPy**	4	5.25	0.76
**MeP5-MOF-2**	2	8.59	0.23

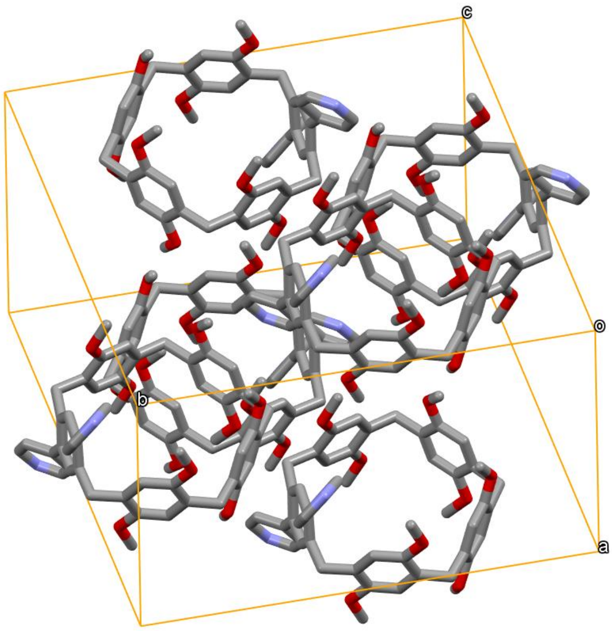

Supplementary Fig. 56 Capped-stick representation of the single crystal structure of **MeP5BPy** in a unit cell. Carbon atoms are grey, oxygen atoms are red, and nitrogen atoms are blue. Hydrogen atoms are omitted for clarity.

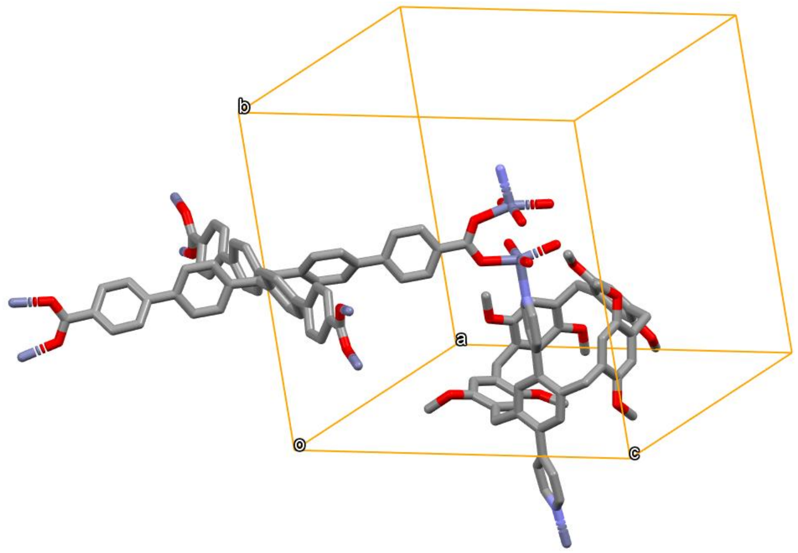

Supplementary Fig. 57 Capped-stick representation of the calculated structure for **MeP5-MOF-1a** based on the backbone of **MeP5-MOF-1** in a unit cell. Carbon atoms are grey, oxygen atoms are red, nitrogen atoms are blue, and zinc atoms are dark blue. Hydrogen atoms are omitted for clarity.

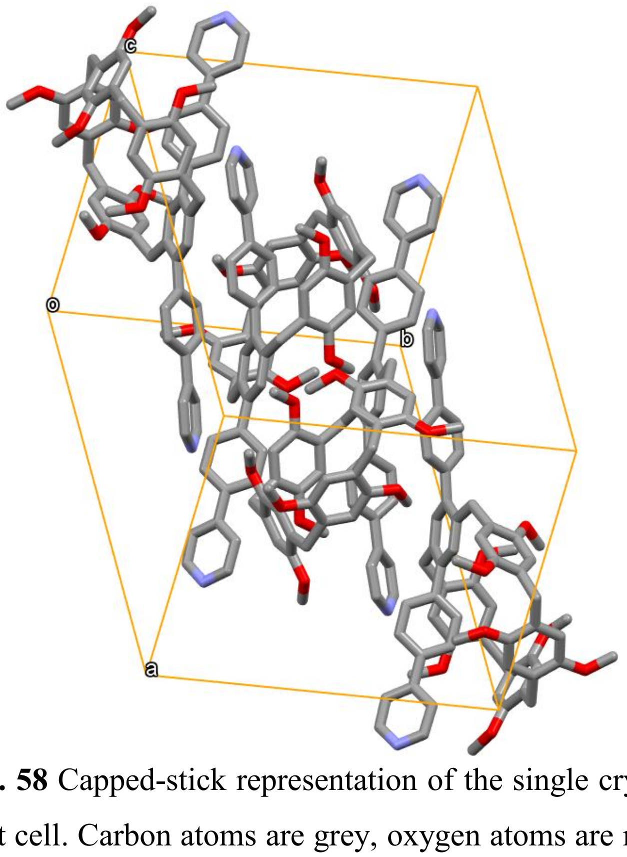

Supplementary Fig. 58 Capped-stick representation of the single crystal structure of **MeP5BPPy** in a unit cell. Carbon atoms are grey, oxygen atoms are red, and nitrogen atoms are blue. Hydrogen atoms are omitted for clarity.

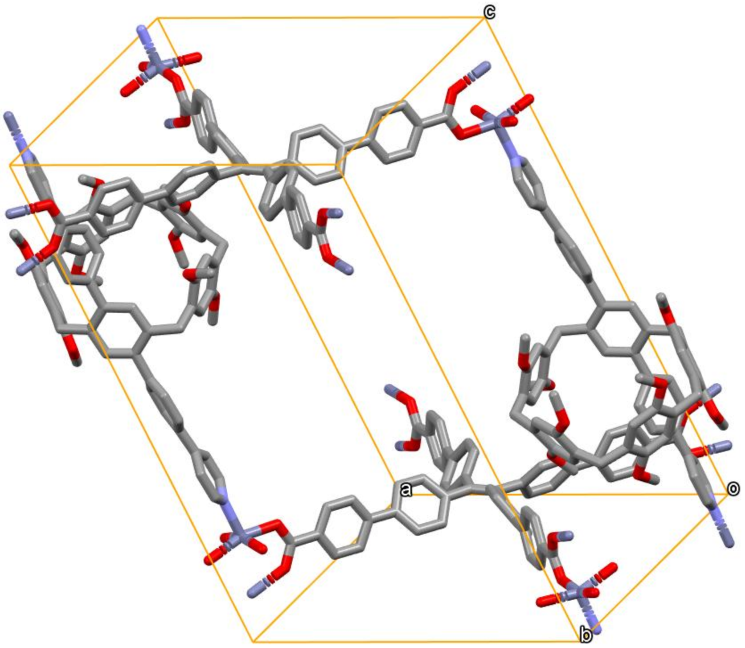

Supplementary Fig. 59 Capped-stick representation of the single crystal structure of **MeP5-MOF-2**. Carbon atoms are grey, oxygen atoms are red, nitrogen atoms are blue, and zinc atoms are dark blue. Hydrogen atoms are omitted for clarity.

Calculations involving rotations of the pillar[5]arene units in **MeP5-MOF-1** and **MeP5-MOF-2** were also carried out. From the calculated structures, the pillar[5]arene units in **MeP5-MOF-1** can rotate 45° at a minimum potential energy point after optimization while the corresponding value is only 10° in **MeP5-MOF-2**. The implications of this difference are discussed in the main text.

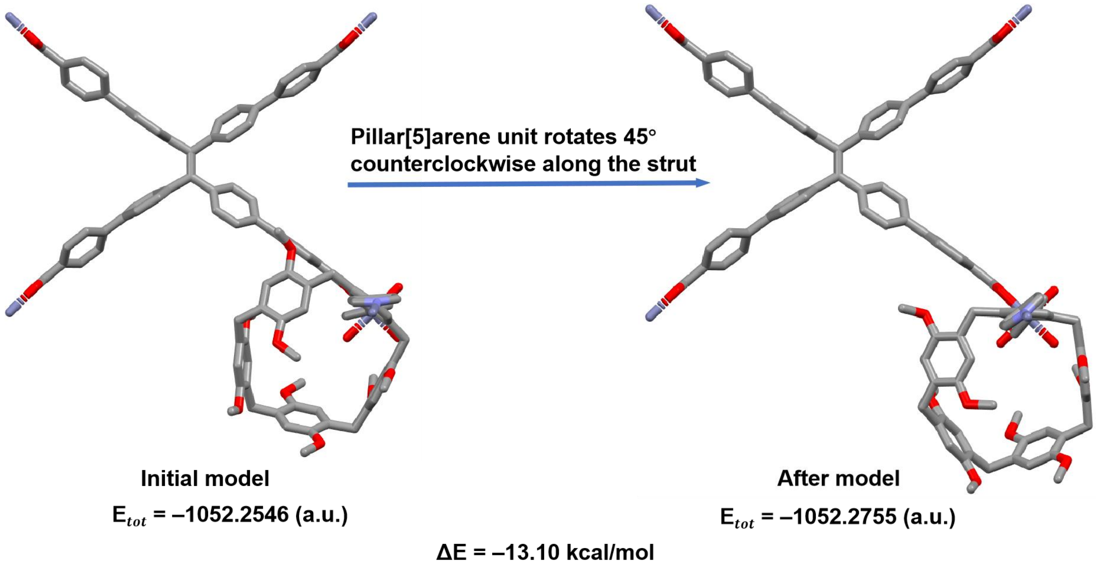

Supplementary Fig. 60 Capped-stick representation of the calculated structure **MeP5-MOF-1a** based on the backbone of **MeP5-MOF-1**. Here, the pillar[5]arene unit on the strut is optimized to rotate 45° at a minimum potential energy point. Carbon atoms are grey, oxygen atoms are red, nitrogen atoms are blue, and zinc atoms are dark blue. Hydrogen atoms are omitted for clarity, 1 a.u. = 627.51 kcal/mol.

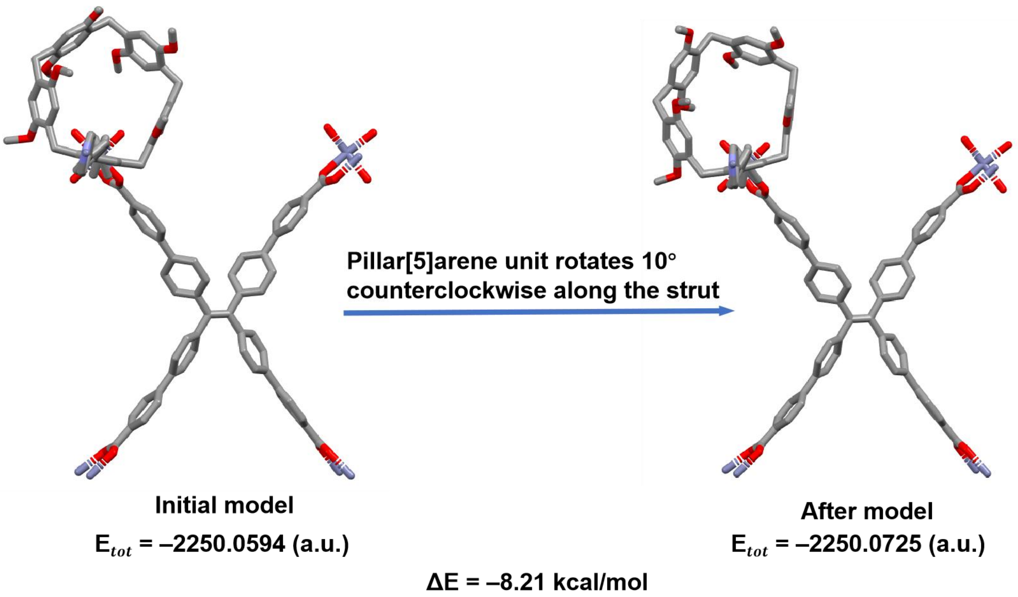

Supplementary Fig. 61 Capped-stick representation of the calculated structure based on the single crystal structure of **MeP5-MOF-2**. Here, the pillar[5]arene unit on the strut is optimized to rotate 10° at a minimum potential energy point. Carbon atoms are grey, oxygen atoms are red, nitrogen atoms are blue, and zinc atoms are dark blue. Hydrogen atoms are omitted for clarity, 1 a.u. = 627.51 kcal/mol.

The intrinsic dynamics of the pillar[5]arene units created challenges in terms of solving the crystal structure based on the diffraction data. It is well known that the *p*-dimethoxy phenyl rings have very low rotation barrier and thus bring rotational disorder to the pillared struts[S11]. Pillar[5]arene building blocks have planar chirality and their racemic mixtures were used for the MOF synthesis. This provides a complication that compounds problems arising from the inherent flexibility of the pillar[5]arenes. The resulting disorder makes **MeP5-MOF-1** an example of a system with "robust dynamics", in which each pillar[5]arene unit enjoys a high degree of freedom and the pillar[5]arene units are anchored to a periodic robust structure. The pillar[5]arene disorder makes the electron density very diffuse in the SCXRD data set.

Most of the carbon atoms in the pillared strut units show high atomic displacement parameters, while the other carbon atoms in the pillar[5]arenes could not be located.

The pillar[5]arene units attached to the struts show rotational disorder around the struts, planar chirality (racemic disorder), and inherent flexibility; collectively, this makes the electron density very diffuse. Diffractions at high theta range were very weak, making the R_{int} value a higher than deemed satisfactory. Therefore, only the positions of all the atoms in the zinc nodes, the layers and the struts could be unambiguously determined, even after several restraints were applied.

The SCXRD data of **MeP5-MOF-1** was collected at 105 K to investigate whether lower temperature could limit the dynamics of the pillar[5]arene units in the frameworks. From the single crystal structure of **MeP5-MOF-1**, the pillar[5]arene units still could not be visually characterized even at 105 K.

An effort was made to use an extra moiety to locate the pillar[5]arene units in the MOFs. Here, **MeP5-MOF-1** was taken as a representative case. Pillar[5]arene is known to capture **TCN** in its cavity[S12]. Therefore, **TCN** was firstly used to locate the pillar[5]arene units in the frameworks. A single crystal of **MeP5-MOF-1** was immersed in a saturated **TCN** solution in acetone for 24 h, and the single crystal was subject to SCXRD data collection. Unfortunately, no effective diffraction was detected at high theta range. Tetrahydrofuran (THF) was also tested as an ancillary moiety to locate the pillar[5]arene units in the frameworks; this was done by immersing the crystals of **MeP5-MOF-1** in THF at room temperature for 24 h. However, the crystal structure data for **MeP5-MOF-1** after THF solvent exchange (denoted as **MeP5-MOF-1-G**) revealed that the THF molecules could not be detected within the frameworks. Moreover, the pillar[5]arene units on the struts were still not resolved.

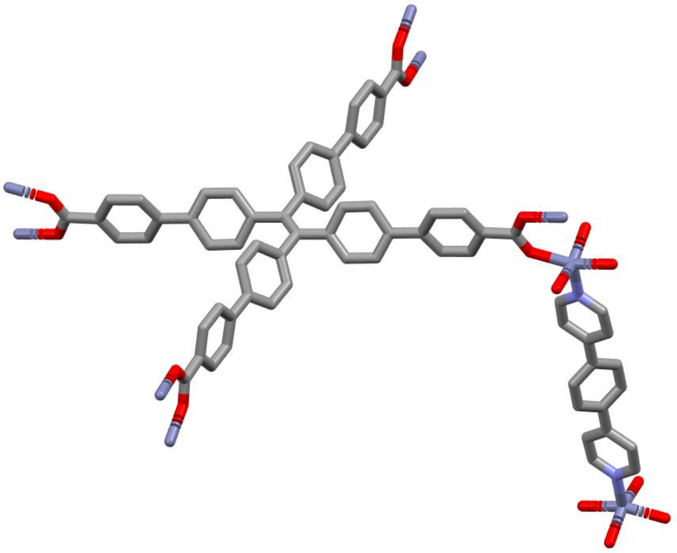

Supplementary Fig. 62 Capped-stick representation of the single crystal structure of **MeP5-MOF-1** measured at 105 K. The pillar[5]arene units on the struts are disordered and not resolved. Carbon atoms are grey, oxygen atoms are red, nitrogen atoms are blue, and zinc atoms are dark blue. Hydrogen atoms are omitted for clarity.

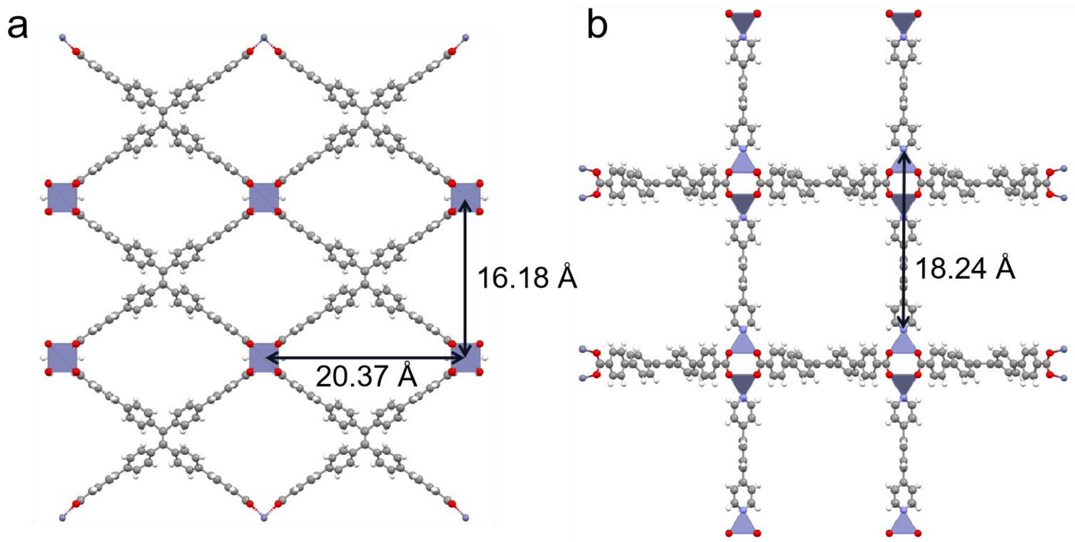

Supplementary Fig. 63 Polyhedral representation of the single crystal structure of **MeP5-MOF-1** measured at 105 K along the *c* axis (a) and the *b* axis (b). The elementary cell is marked with an orange cuboid (a = 16.18 Å, b = 20.37 Å, c = 18.24 Å). The pillar[5]arene units on the struts are disordered and not resolved. Carbon atoms are grey, hydrogen atoms are white, oxygen atoms are red, nitrogen atoms are blue, and zinc atoms are dark blue.

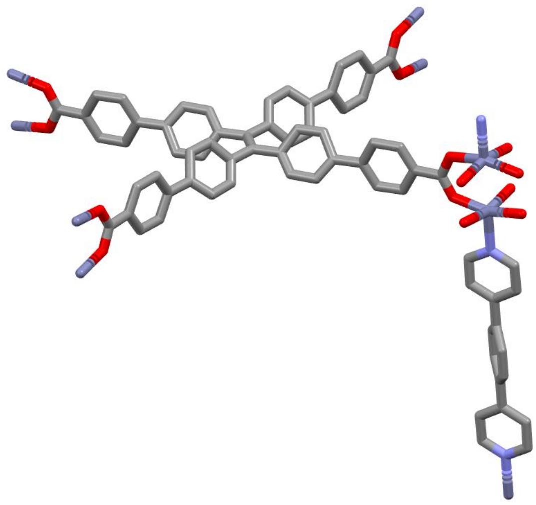

Supplementary Fig. 64 Capped-stick representation of the single crystal structure of **MeP5-MOF-1-G**. The pillar[5]arene units on the struts are disordered and not resolved. Carbon atoms are grey, oxygen atoms are red, nitrogen atoms are blue, and zinc atoms are dark blue. Hydrogen atoms are omitted for clarity.

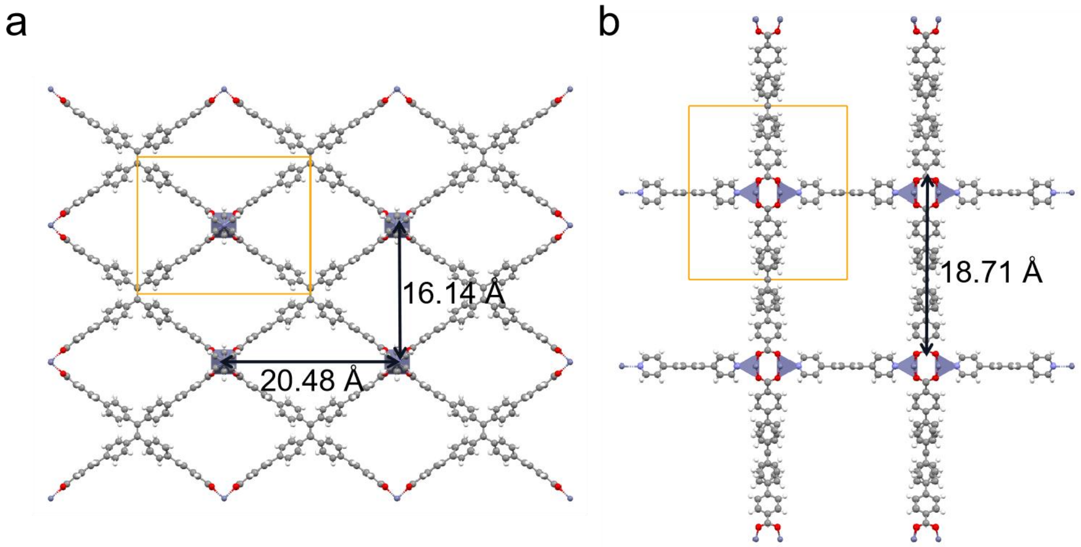

Supplementary Fig. 65 Polyhedral representation of the single crystal structure of **MeP5-MOF-1-G** along the *c* axis (a) and the *b* axis (b). The elementary cell is marked with an orange cuboid (a = 16.14 Å, b = 20.48 Å, c = 18.71 Å). The pillar[5]arene units on the struts are disordered and not resolved. Carbon atoms are grey, hydrogen atoms are white, oxygen atoms are red, nitrogen atoms are blue, and zinc atoms are dark blue.

8. Powder X-ray diffraction patterns of MOFs

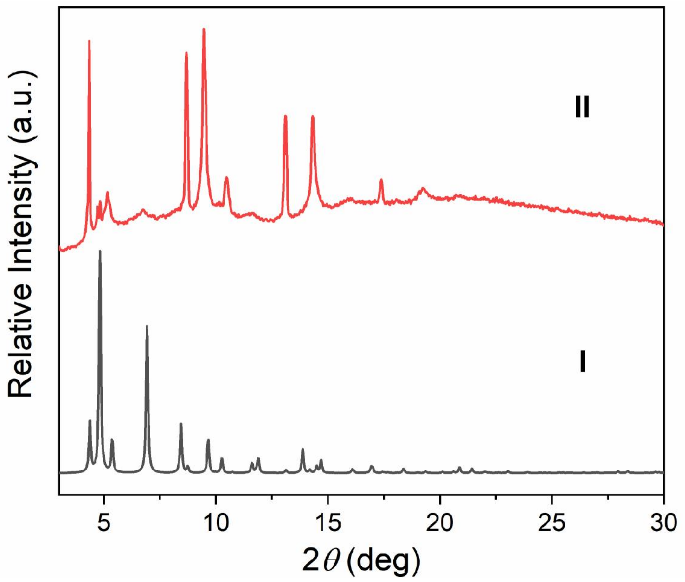

Supplementary Fig. 66 PXRD patterns of **MeP5-MOF-1**: **I**, simulated from the single crystal structure; **II**, from a single crystal sample.

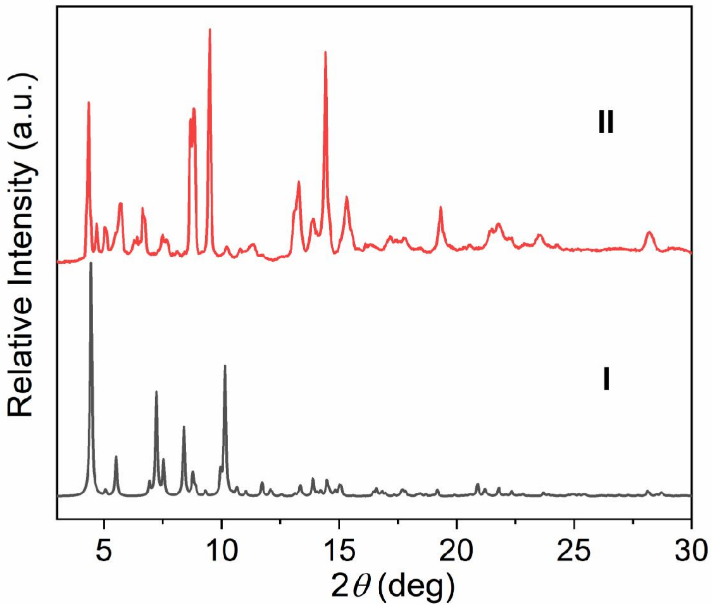

Supplementary Fig. 67 PXRD patterns of **Model-MOF-1**: **I**, simulated from the single crystal structure; **II**, from a single crystal sample.

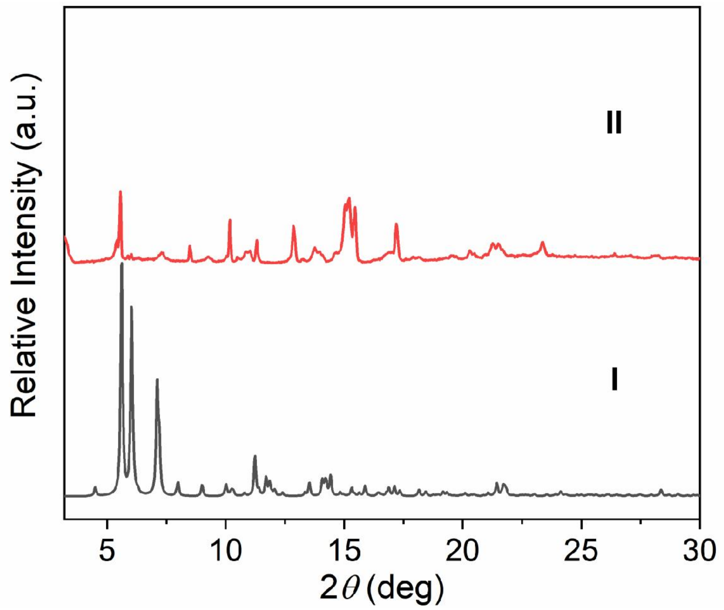

Supplementary Fig. 68 PXRD patterns of **MeModel-MOF-1**: **I**, simulated from the single crystal structure; **II**, from a single crystal sample.

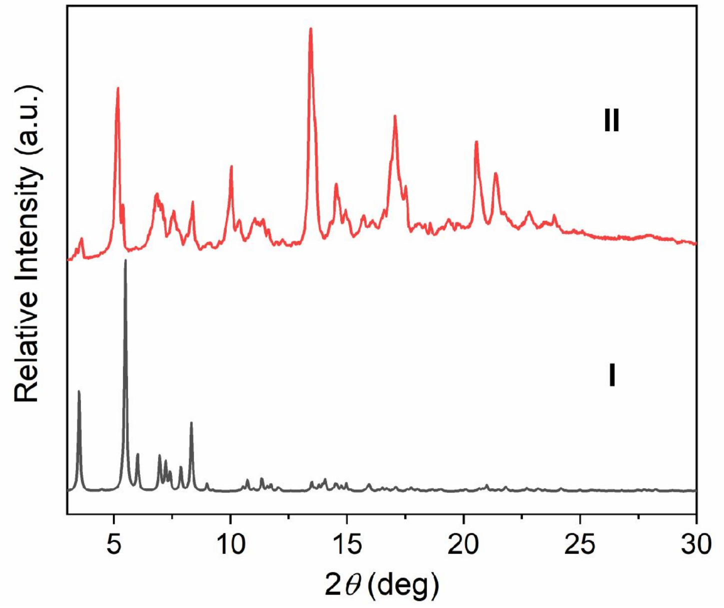

Supplementary Fig. 69 PXRD patterns of **MeP5-MOF-2**: **I**, simulated from the single crystal structure; **II**, from a single crystal sample.

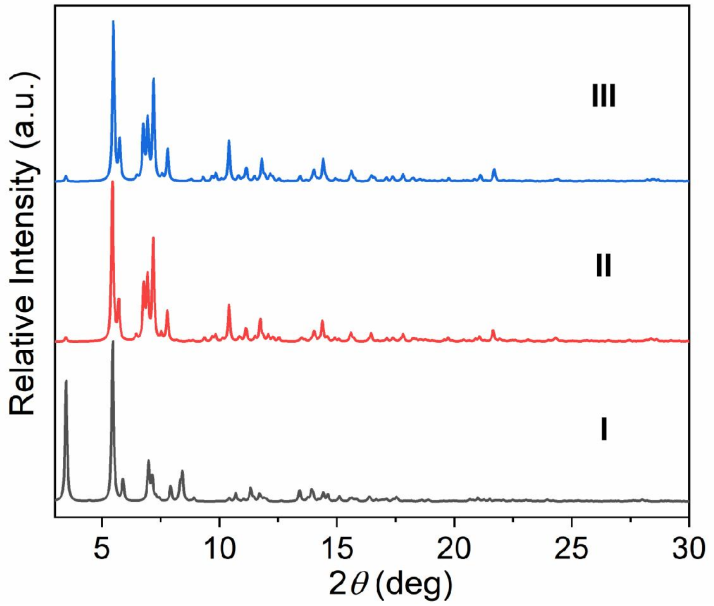

Supplementary Fig. 70 Comparison of PXRD patterns simulated from their single crystal structures: **I**, *racemic*-**MeP5-MOF-2**; **II**, *pS*-**MeP5-MOF-2**; **III**, *pR*-**MeP5-MOF-2**. These results reflect the various structural arrangements of the pillar[5]arene repearting units in *racemic*-**MeP5-MOF-2** and *pS*-**MeP5-MOF-2**/*pR*-**MeP5-MOF-2**.

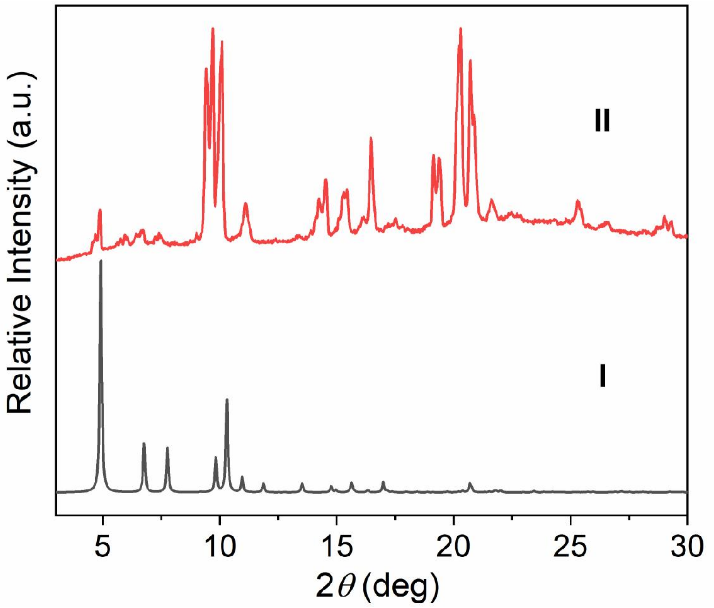

Supplementary Fig. 71 PXRD patterns of **MeP5-MOF-3**: **I**, simulated from the single crystal structure; **II**, from a single crystal sample.

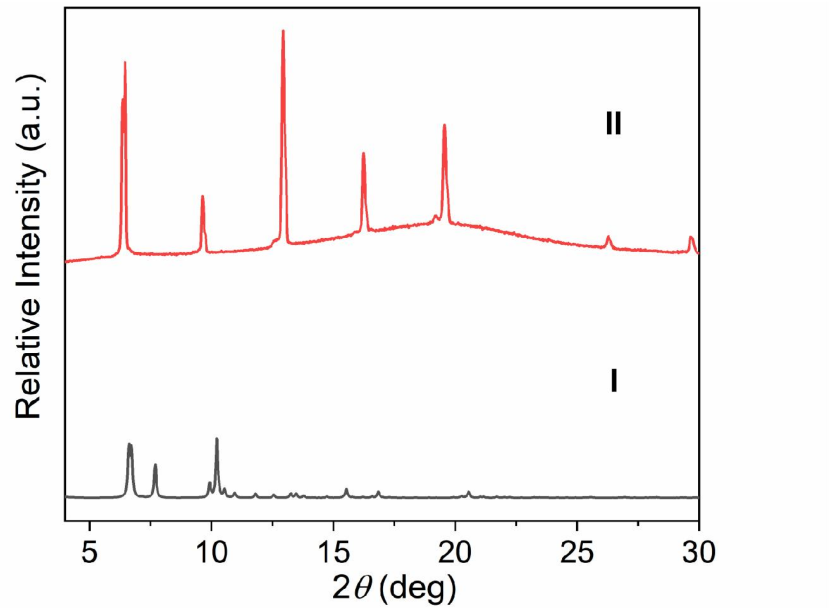

Supplementary Fig. 72 PXRD patterns of **MeP5-MOF-4**: **I**, simulated from the single crystal structure; **II**, from a single crystal sample.

9. Guest binding studies of pillar[5]arene struts

9.1. Spectroscopic studies of pillar[5]arene struts

The charge-transfer absorption (λ = 430–480 nm) of the presumed host–guest complexes overlaps with the emission (λ = 450–500 nm) of the pillar[5]arene-based struts. The fluorescence is thus quenched upon formation of a host–guest complex.

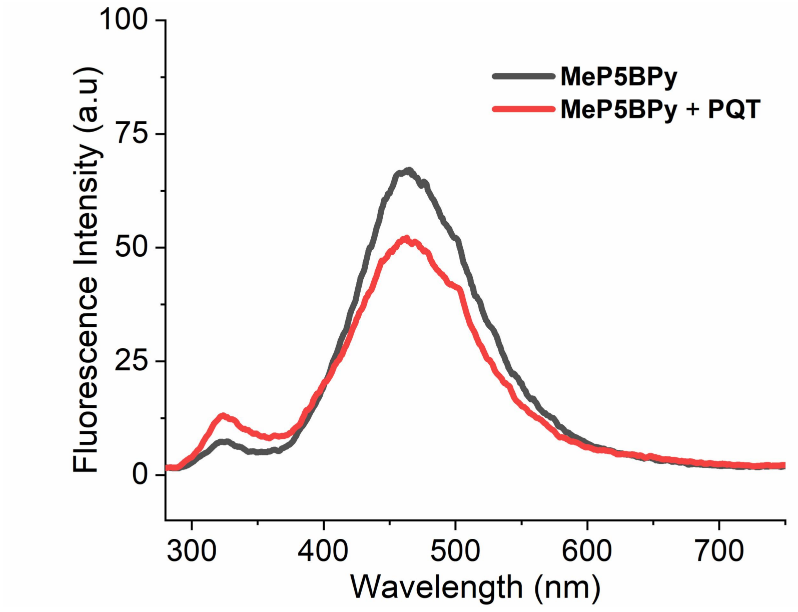

Supplementary Fig. 73 Fluorescence spectra of **MeP5BPy** (20.0 μM, black) before and after adding **PQT** (20.0 μM, red) in acetone at room temperature, λ_{ex} = 250 nm.

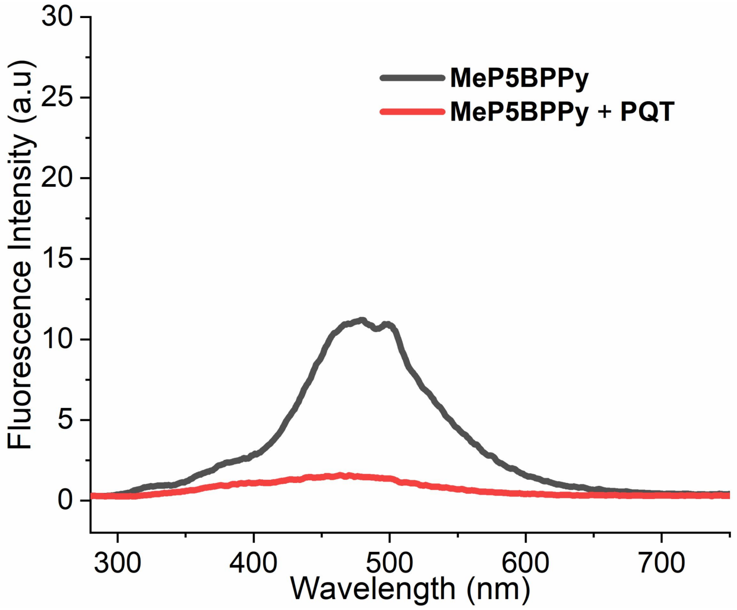

Supplementary Fig. 74 Fluorescence spectra of **MeP5BPPy** (20.0 μM, black) before and after adding **PQT** (20.0 μM, red) in acetone at room temperature, λ_{ex} = 250 nm.

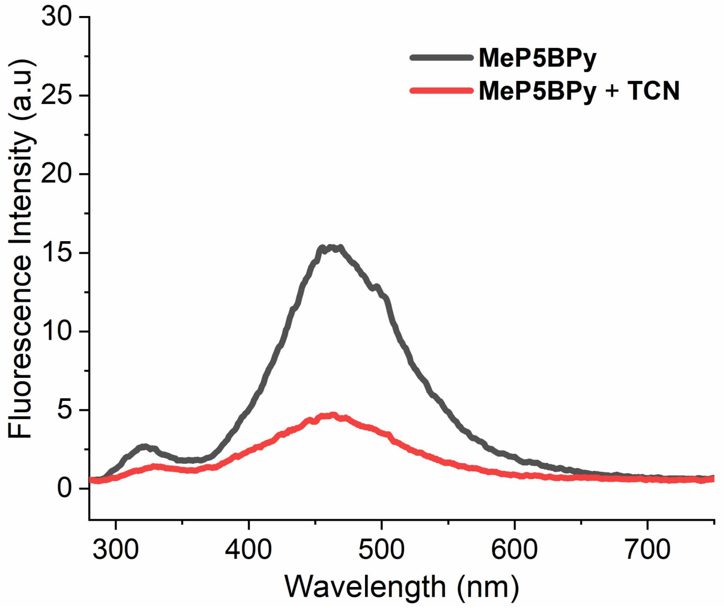

Supplementary Fig. 75 Fluorescence spectra of **MeP5BPy** (20.0 μM, black) before and after adding **TCN** (20.0 μM, red) in acetone at room temperature, λ_{ex} = 250 nm.

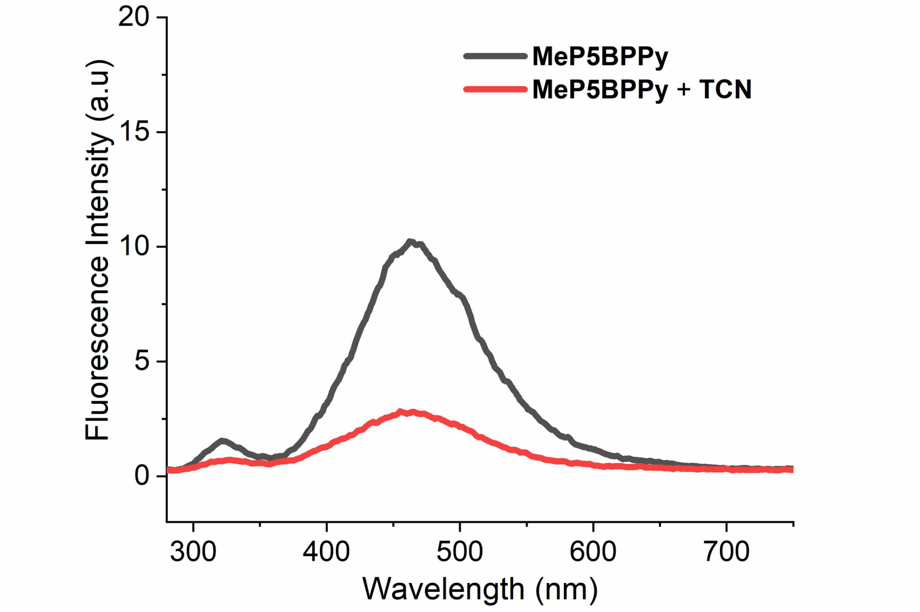

Supplementary Fig. 76 Fluorescence spectra of **MeP5BPPy** (20.0 μM, black) before and after adding **TCN** (20.0 μM, red) in acetone at room temperature, $\lambda_{ex} = 250$ nm.

9.2. Determination of binding constants of pillar[5]arene struts

To determine the association constants and binding stoichiometries of the complexes formed between the pillar[5]arene struts and the guests considered in this study, fluorescence spectral titrations were performed with solutions that contained a constant concentration of hosts, **MeP5BPy** and **MeP5BPPy** (20.0 μM), and varying concentrations of guests, **PQT** and **TCN**. The association constants (K_a) were determined using a non-linear curve-fitting method. Stoichiometries were obtained for the complexation between hosts and guests by utilizing a mole ratio plot.

The non-linear curve-fitting was based on the equation (1):[S13]

$$\Delta\delta = (\Delta\delta_{\infty}/[H]_0)\ (0.5[G]_0 + 0.5([H]_0 + 1/K_a) - (0.5([G]_0^2 + (2[G]_0\ (1/K_a - [H]_0)) + (1/K_a + [H]_0)^2)^{0.5}))$$

where $\Delta\delta$ is the change of fluorescence intensity of the struts, $\Delta\delta_{\infty}$ is the change of fluorescence intensity when the hosts are completely complexed, $[G]_0$ is the initial concentration of the guests, and $[H]_0$ is the fixed initial concentration of the hosts.

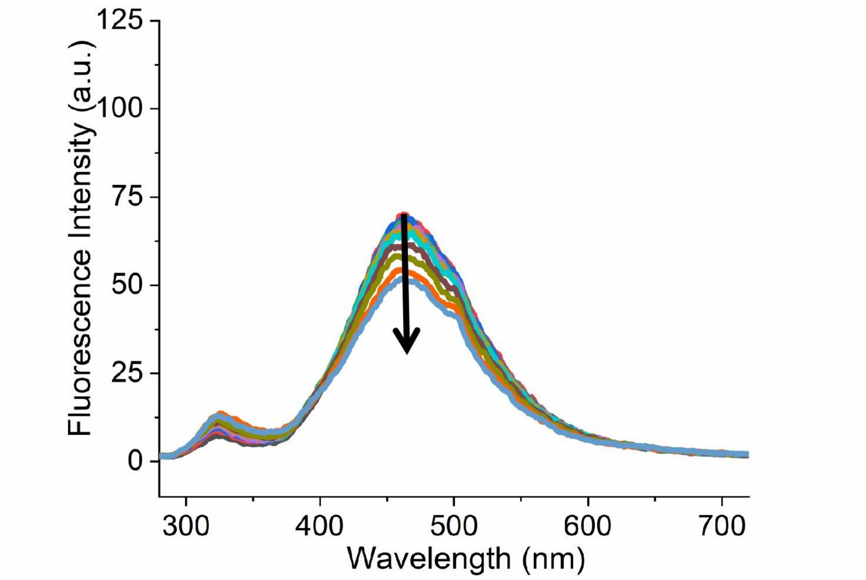

Supplementary Fig. 77 Fluorescence spectra of **MeP5BPy** at a constant concentration of 20.0 μM upon addition of **PQT** at various concentrations: 0.00 μM, 1.00 μM, 2.00 μM, 3.00 μM, 6.00 μM, 10.0 μM, 15.0 μM, 23.0 μM, 31.0 μM, 44.0 μM, 55.0 μM and 69.0 μM. The excitation wavelength is 250 nm.

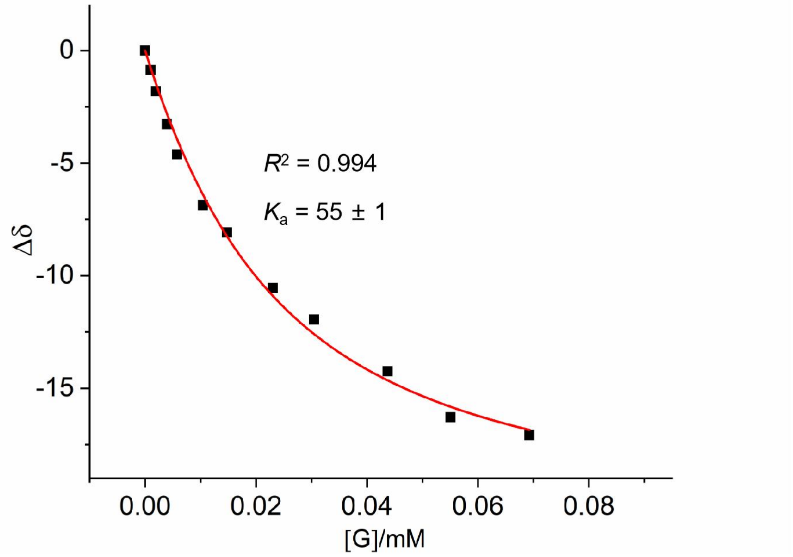

Supplementary Fig. 78 The changes of fluorescence intensity of **MeP5BPy** upon addition of **PQT**. The red solid line was obtained from the non-linear curve-fitting using Eq. S1. The association constant (K_a) of **MeP5BPy** and **PQT** was calculated to be 55 ± 1 M^{-1}.

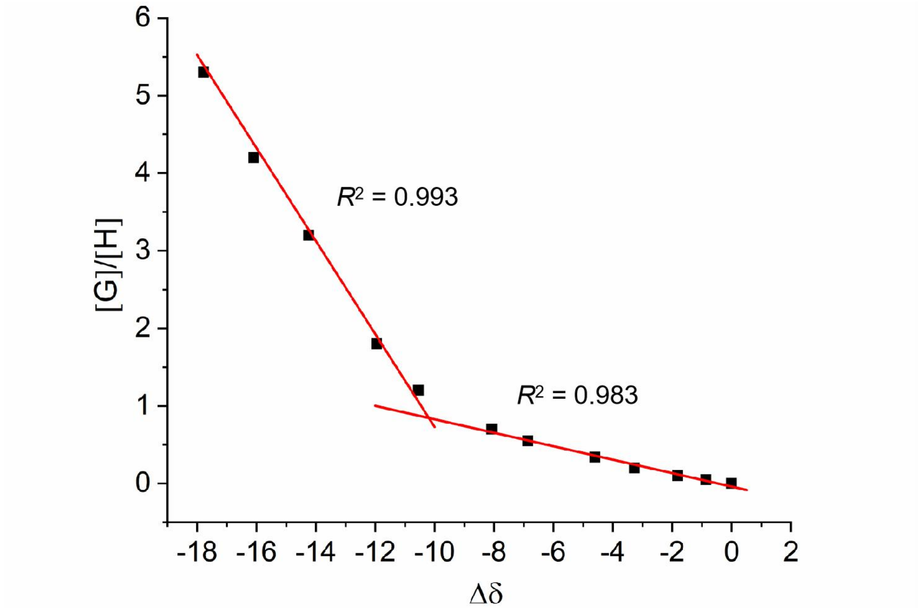

Supplementary Fig. 79 Mole ratio plot of **MeP5BPy** and **PQT**, consistent with a 1:1 stoichiometry.

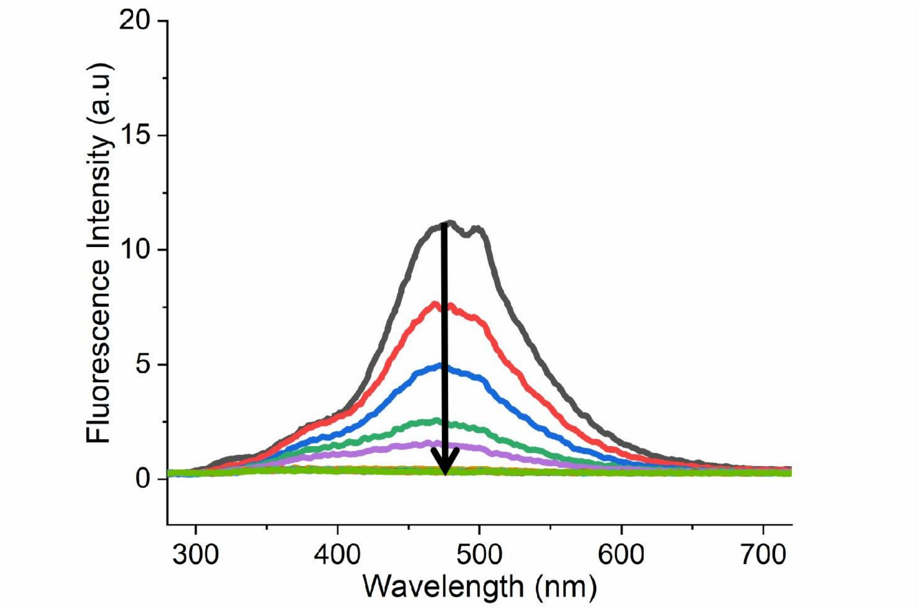

Supplementary Fig. 80 Fluorescence spectra of **MeP5BPPy** at a constant concentration of 20.0 μM upon addition of **PQT** at various concentrations: 0.00 μM, 1.00 μM, 2.00 μM, 3.00 μM, 6.00 μM, 10.0 μM, 15.0 μM, 23.0 μM, 31.0 μM, 44.0 μM, 55.0 μM and 77.0 μM. The excitation wavelength is 250 nm.

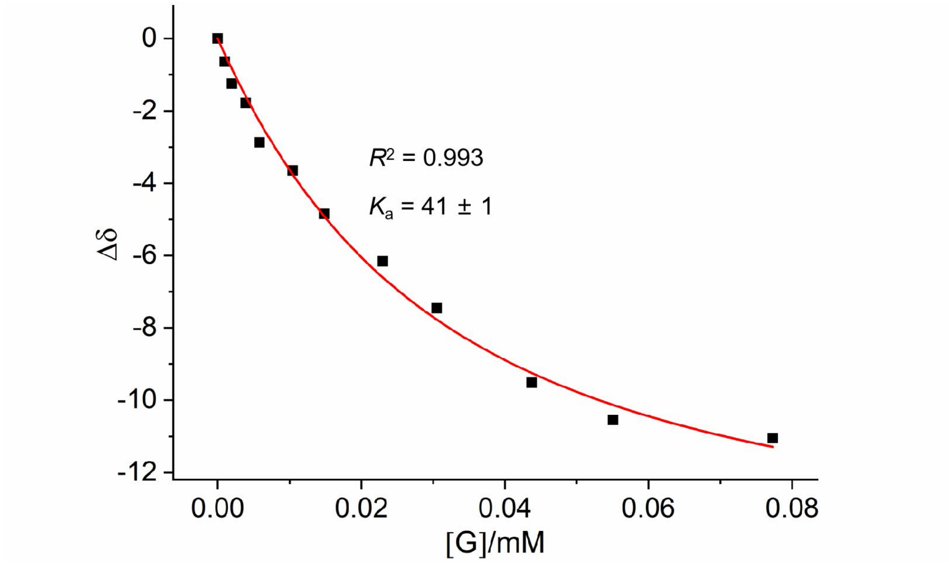

Supplementary Fig. 81 The changes of fluorescence intensity of **MeP5BPPy** upon addition of **PQT**. The red solid line was obtained from the non-linear curve-fitting using Eq. S1. The association constant (K_a) of **MeP5BPPy** and **PQT** was calculated to be 41 ± 1 M^{-1}.

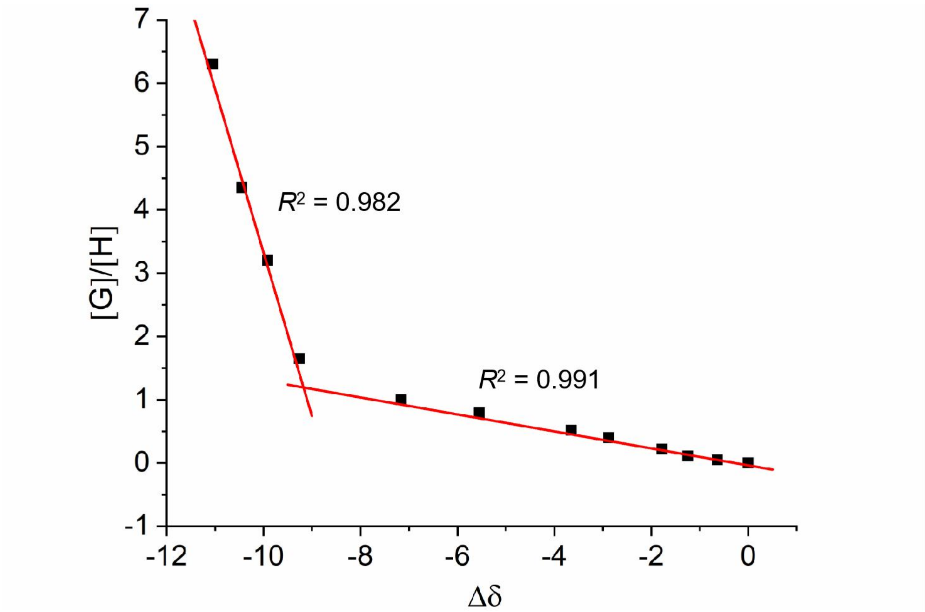

Supplementary Fig. 82 Mole ratio plot of **MeP5BPPy** and **PQT**, consistent with a 1:1 stoichiometry.

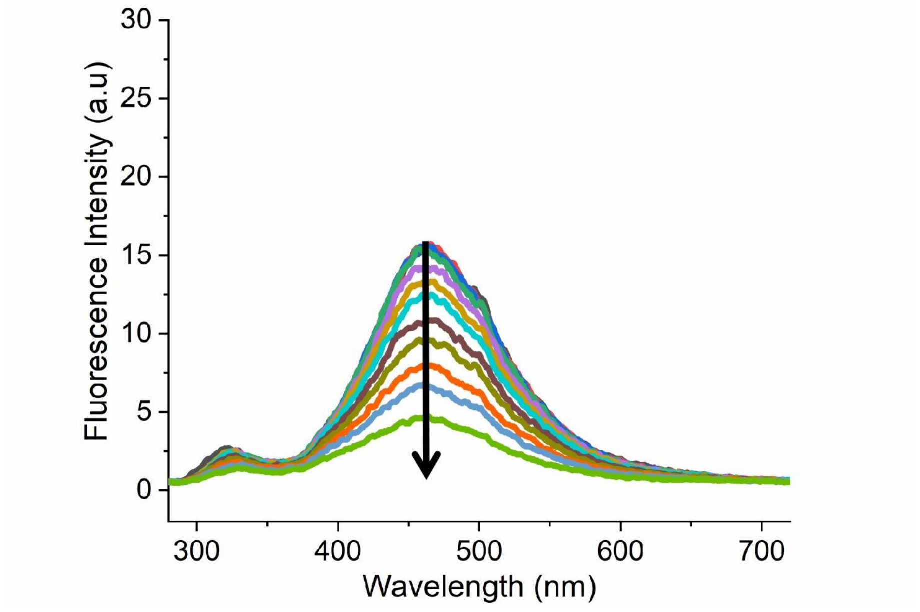

Supplementary Fig. 83 Fluorescence spectra of **MeP5BPy** at a constant concentration of 20.0 μM upon addition of **TCN** at various concentrations: 0.00 μM, 1.00 μM, 2.00 μM, 3.00 μM, 6.00 μM, 10.0 μM, 15.0 μM, 23.0 μM, 31.0 μM, 44.0 μM, 55.0 μM and 77.0 μM. The excitation wavelength is 250 nm.

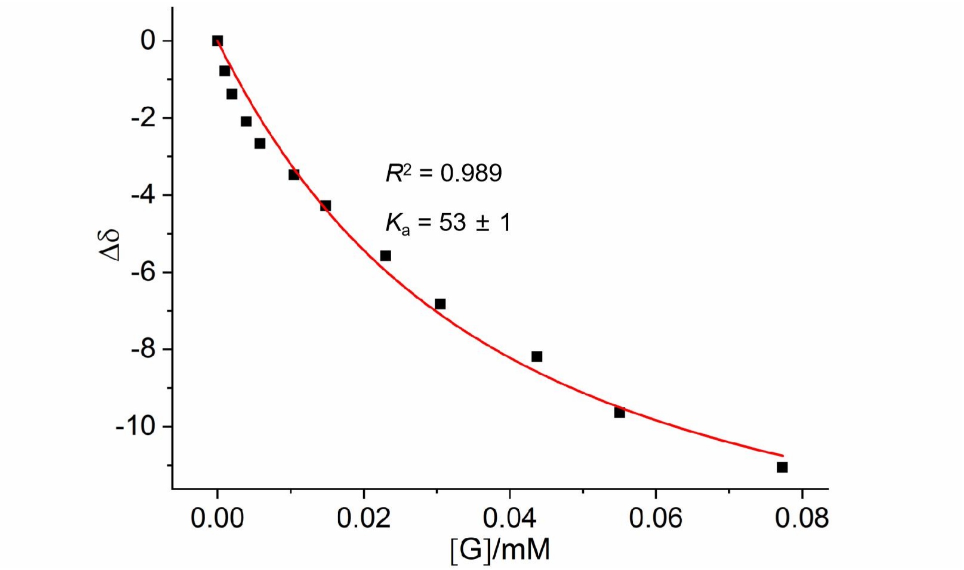

Supplementary Fig. 84 The changes of fluorescence intensity of **MeP5BPy** upon addition of **TCN**. The red solid line was obtained from the non-linear curve-fitting using Eq. S1. The association constant (K_a) of **MeP5BPy** and **TCN** was calculated to be 53 ± 1 M^{-1}.

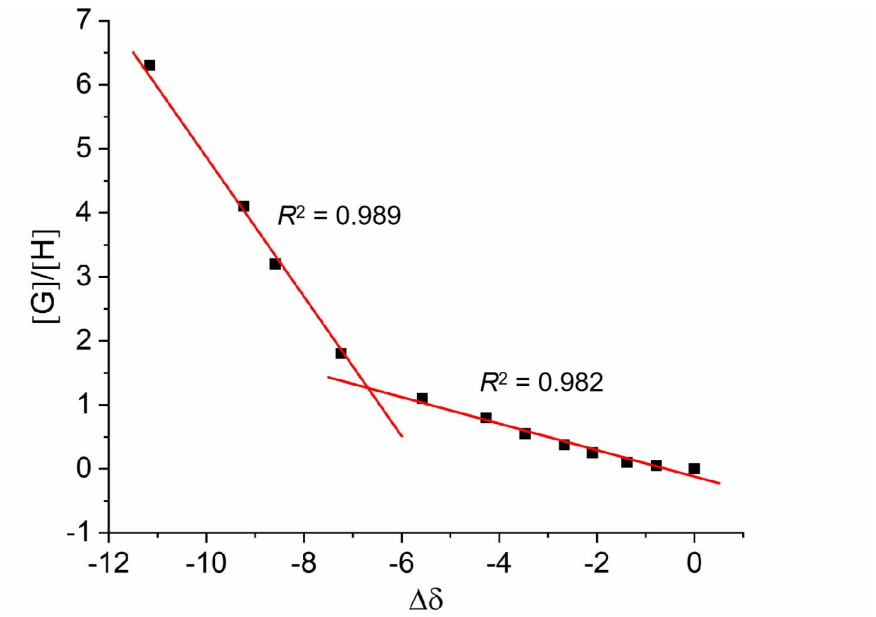

Supplementary Fig. 85 Mole ratio plot of **MeP5BPy** and **TCN**, consistent with a 1:1 stoichiometry.

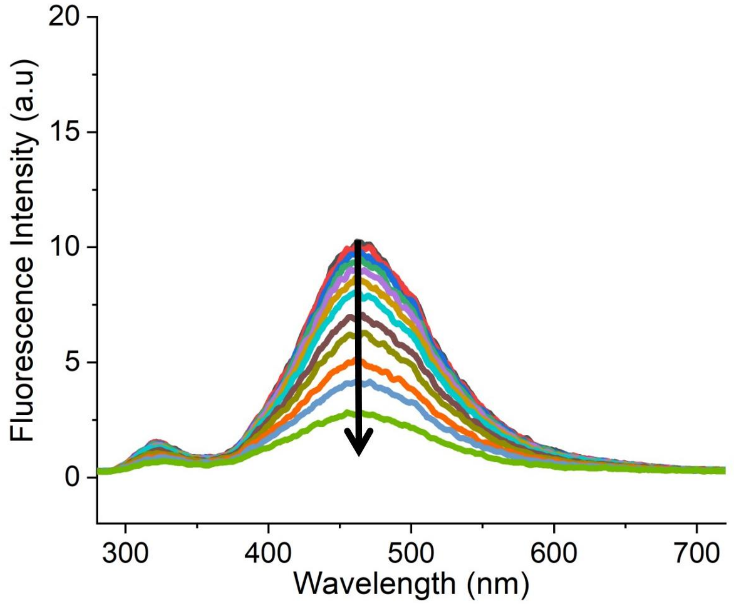

Supplementary Fig. 86 Fluorescence spectra of **MeP5BPPy** at a constant concentration of 20.0 μM upon addition of **TCN** at various concentrations: 0.00 μM, 1.00 μM, 2.00 μM, 3.00 μM, 6.00 μM, 10.0 μM, 15.0 μM, 23.0 μM, 31.0 μM, 44.0 μM, 55.0 μM and 77.0 μM. The excitation wavelength is 250 nm.

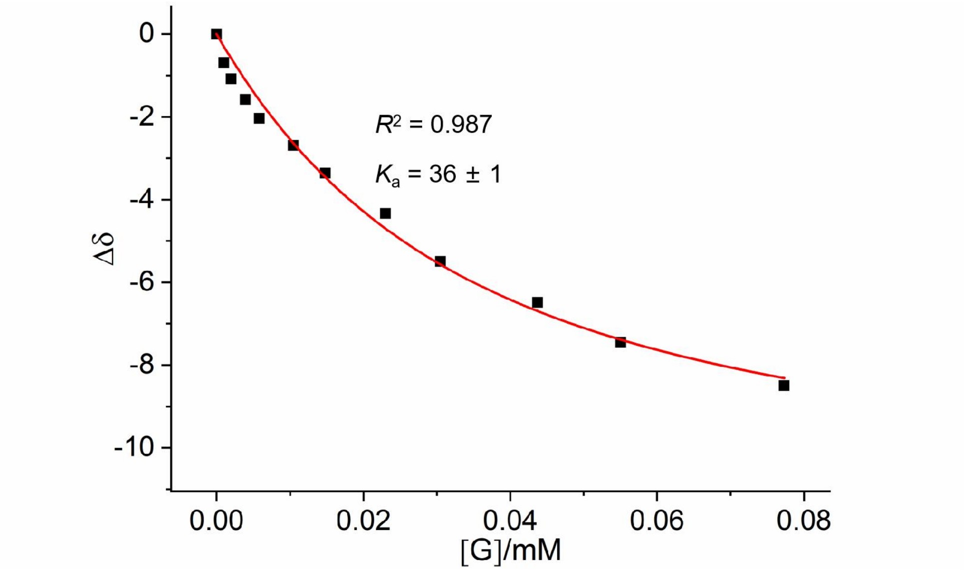

Supplementary Fig. 87 The changes of fluorescence intensity of **MeP5BPPy** upon addition of **TCN**. The red solid line was obtained from the non-linear curve-fitting using Eq. S1. The association constant (K_a) of **MeP5BPPy** and **TCN** was calculated to be 36 ± 1 M^{-1}.

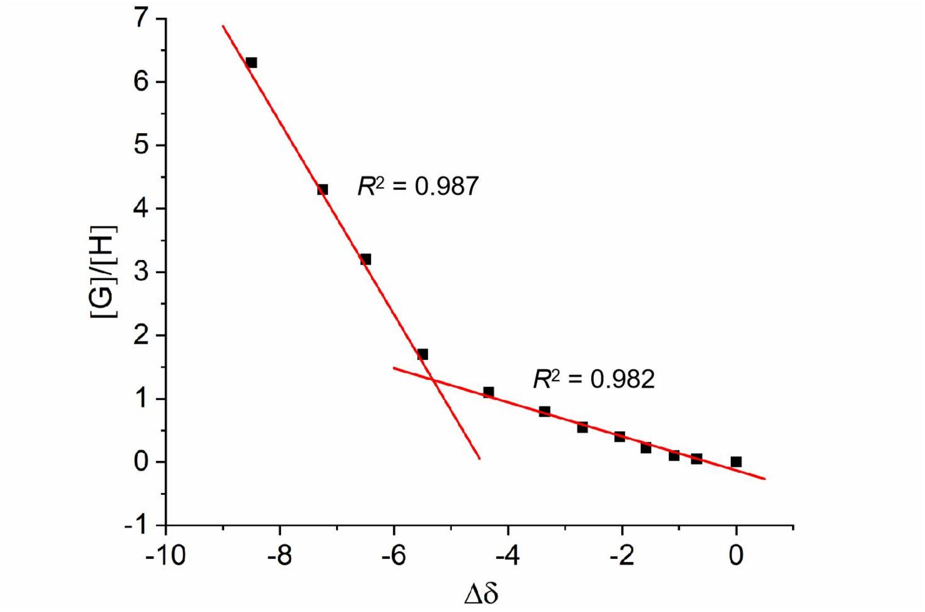

Supplementary Fig. 88 Mole ratio plot of **MeP5BPPy** and **TCN**, consistent with a 1:1 stoichiometry.

10. Molecular recognition studies of MOFs

The MOFs were allowed to cool to room temperature directly after their syntheses. The mother liquors were removed and washed with acetone. After 6 hours, the acetone was removed and replaced with fresh acetone. This process was repeated five

more times until all the mother liquors were removed from the crystals of MOFs. Crystals of MOFs were then added to a solution of the guest, saturated with a single guest, either paraquat (**PQT**) or 1,2,4,5-tetracyanobenzene (**TCN**). The crystals were allowed to take up the guest molecules for 12 hours at room temperature. The crystals were then filtered off and washed with fresh acetone three times (10 mL). The crystals were then dried under vacuum to remove excess acetone and dissolved in DMSO-d_6/DCl (100:1 *v*/*v*). The mole ratio of the guest to the organic linker was determined from the integrations of the corresponding ^{1}H NMR (500 MHz, 298 K) signals.

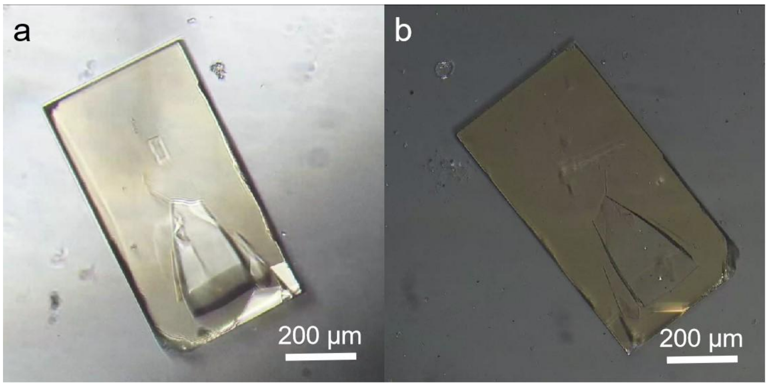

Supplementary Fig. 89 Optical microscopy images of single crystals of **MeP5-MOF-1** before (a) and after (b) immersing in a **PQT** solution (20.0 μM).

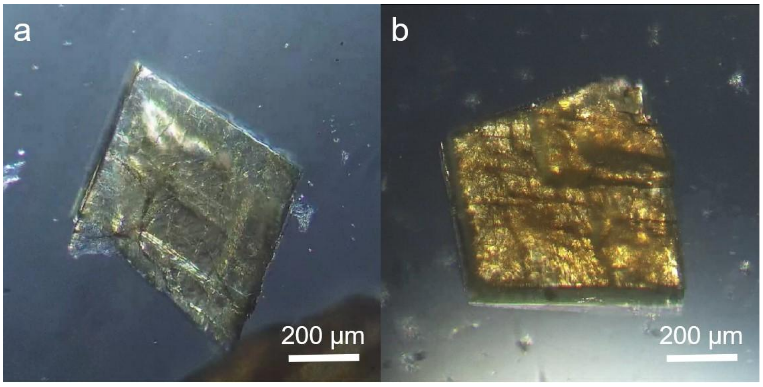

Supplementary Fig. 90 Optical microscopy images of single crystals of **MeP5-MOF-2** before (a) and after (b) immersing in a **PQT** solution (20.0 μM).

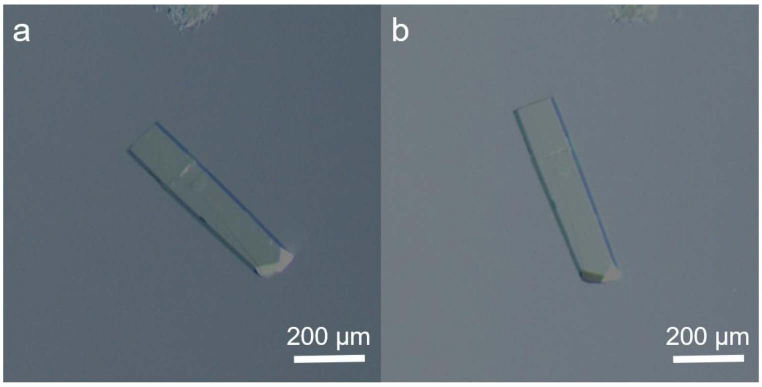

Supplementary Fig. 91 Optical microscopy images of single crystals of **Model-MOF-1** before (a) and after (b) immersing in a **PQT** solution (20.0 μM).

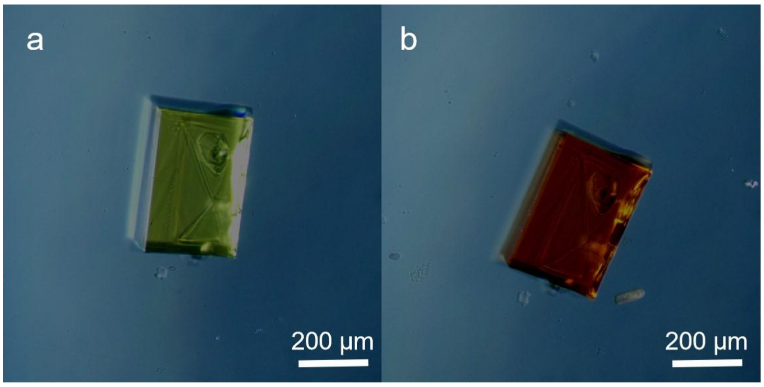

Supplementary Fig. 92 Optical microscopy images of single crystals of **MeP5-MOF-1** before (a) and after (b) immersing in a **TCN** solution (20.0 μM).

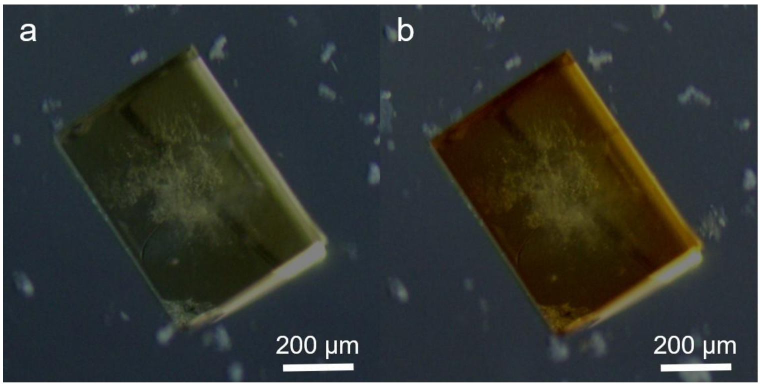

Supplementary Fig. 93 Optical microscopy images of single crystals of **MeP5-MOF-2** before (a) and after (b) immersing in a **TCN** solution (20.0 μM).

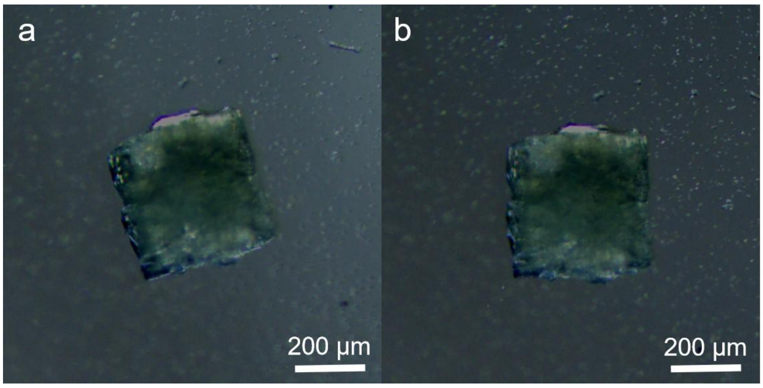

Supplementary Fig. 94 Optical microscopy images of single crystals of **Model-MOF-1** before (a) and after (b) immersing in a **TCN** solution (20.0 μM).

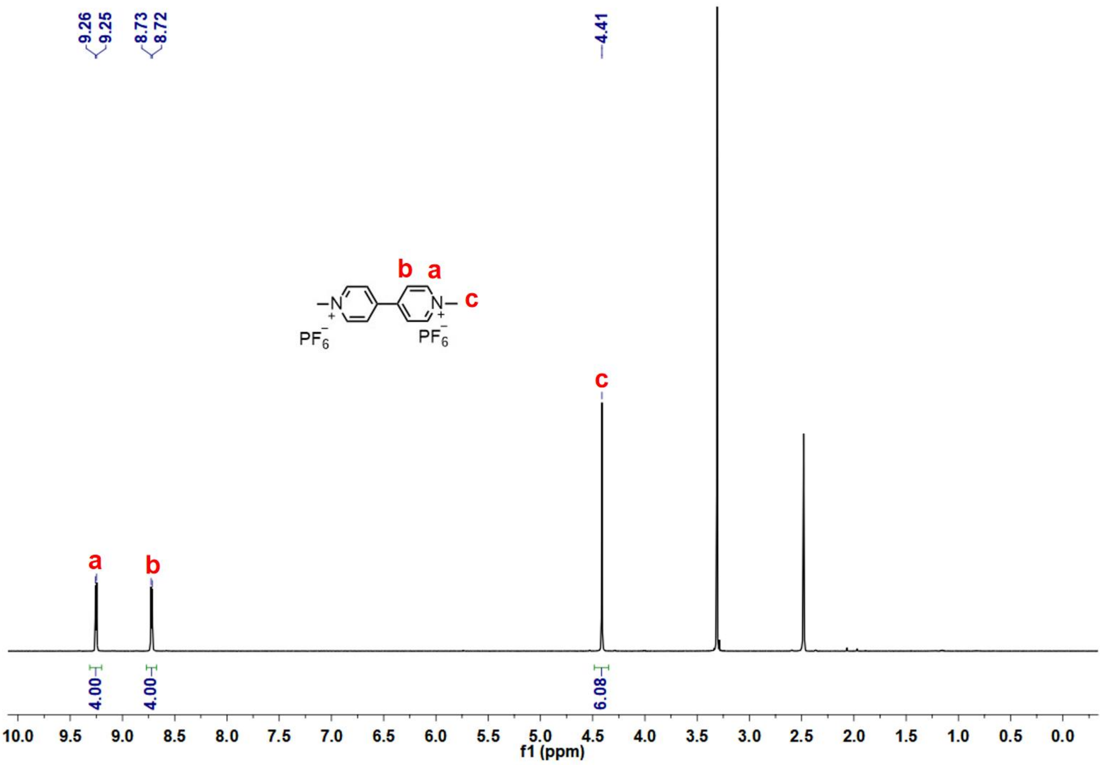

Supplementary Fig. 95 ^{1}H NMR (500 MHz, DMSO-d_6, 298 K) spectrum of **PQT**.

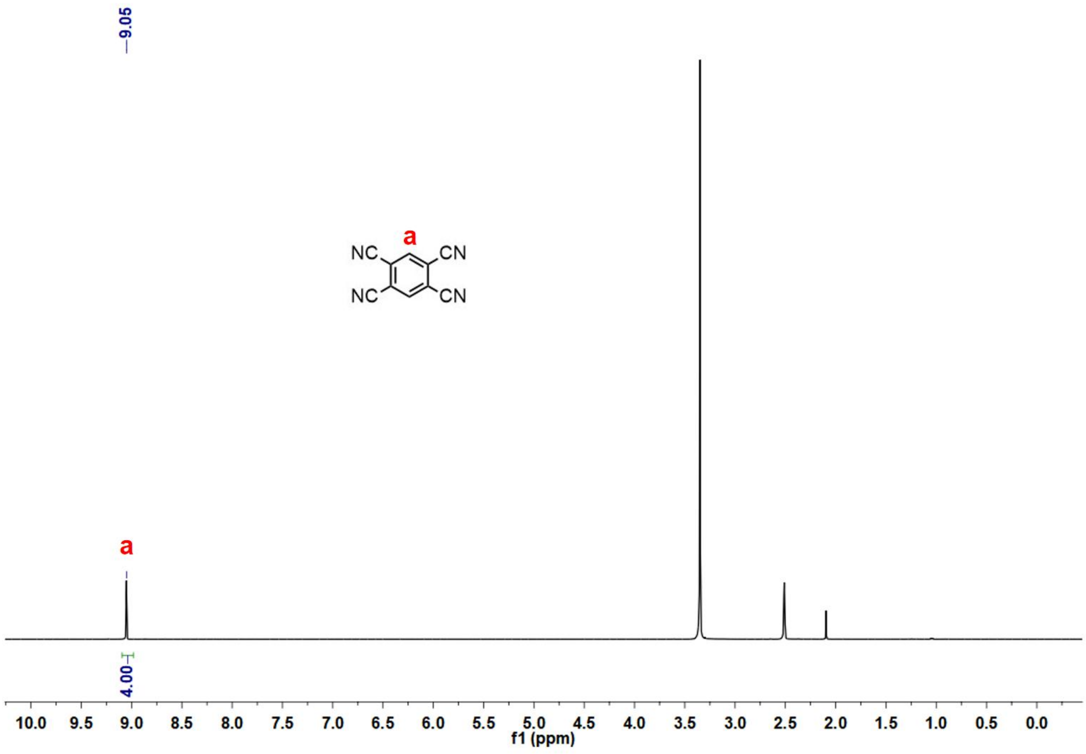

Supplementary Fig. 96 ^{1}H NMR (500 MHz, DMSO-d_6, 298 K) spectrum of **TCN**.

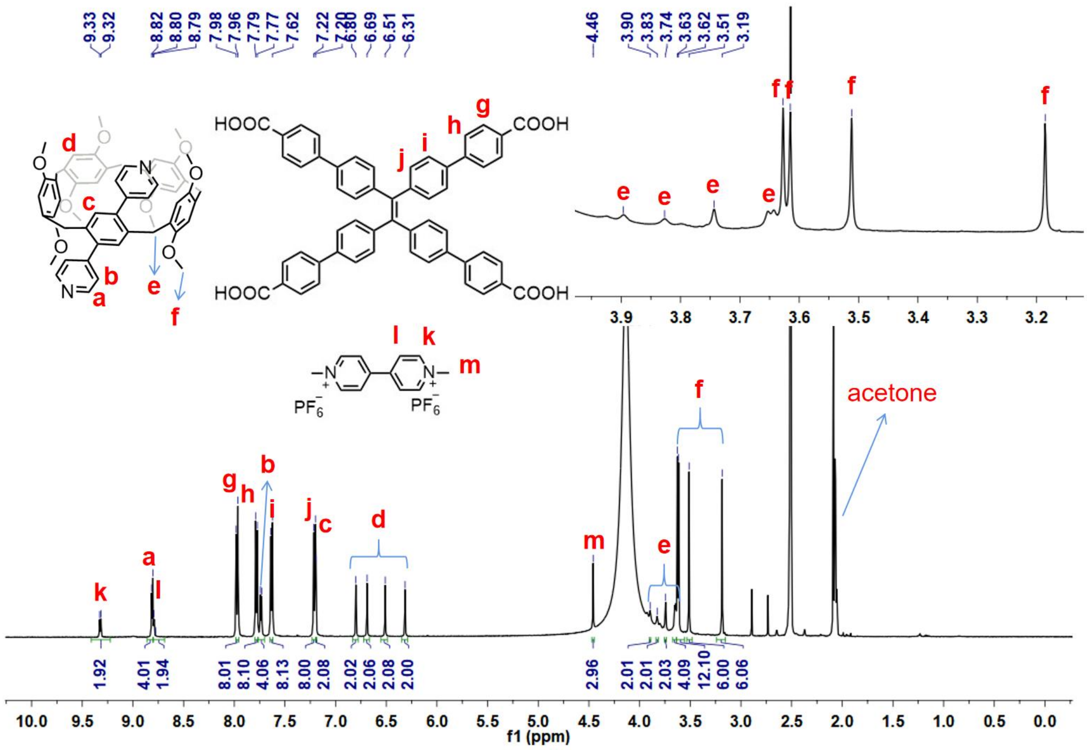

Supplementary Fig. 97 ^{1}H NMR (500 MHz, DMSO-d_6:DCl = 100:1, 298 K) spectrum of **MeP5-MOF-1** after digestion following uptake of **PQT**.

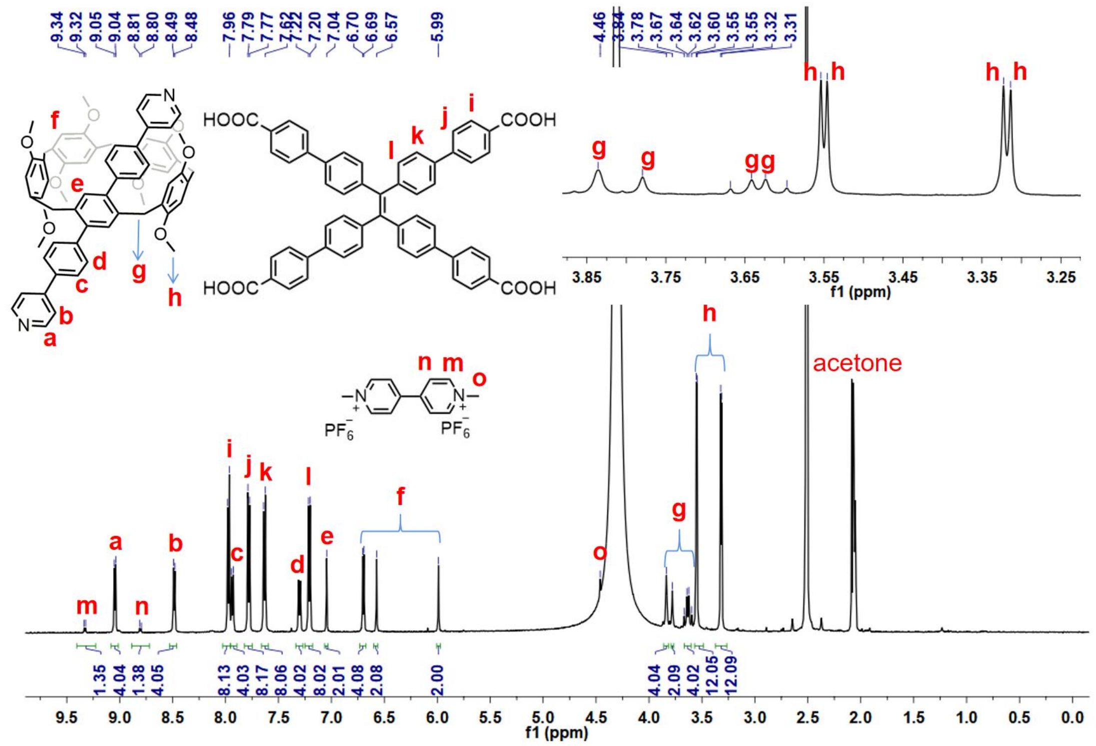

Supplementary Fig. 98 ^{1}H NMR (500 MHz, DMSO-d_6:DCl = 100:1, 298 K) spectrum of **MeP5-MOF-2** after digestion following uptake of **PQT**. Here one signal of proton **o** is merged into the signal of the acid at 4.40–4.50 ppm.

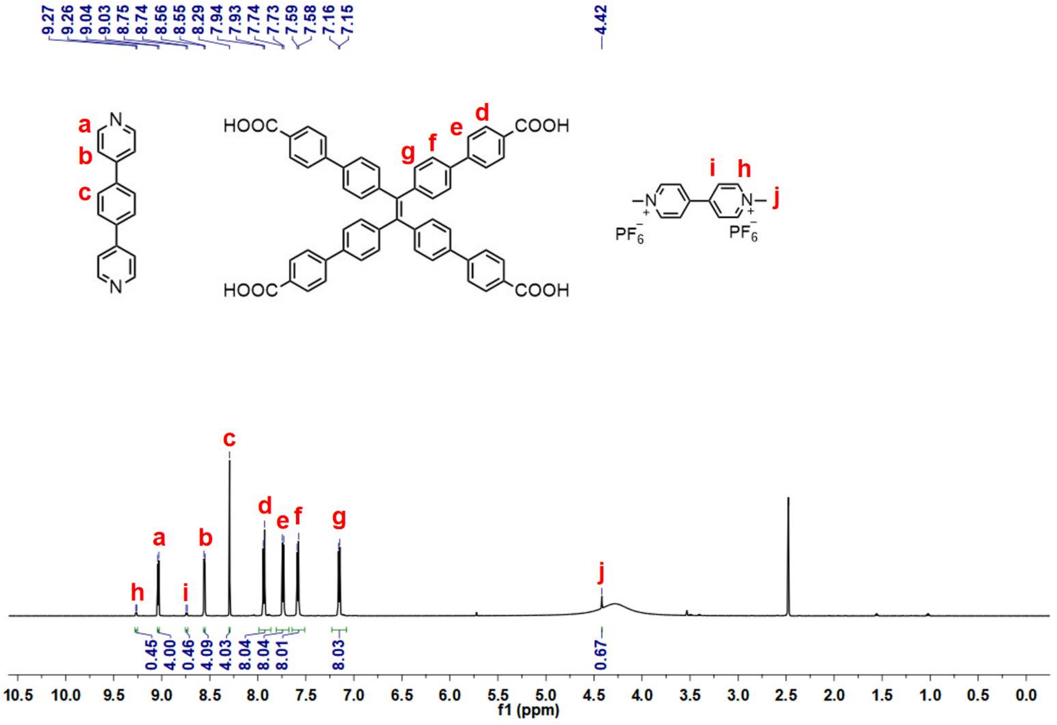

Supplementary Fig. 99 ^{1}H NMR (500 MHz, DMSO-d_6:DCl = 100:1, 298 K) spectrum of **Model-MOF-1** after digestion following uptake of **PQT**.

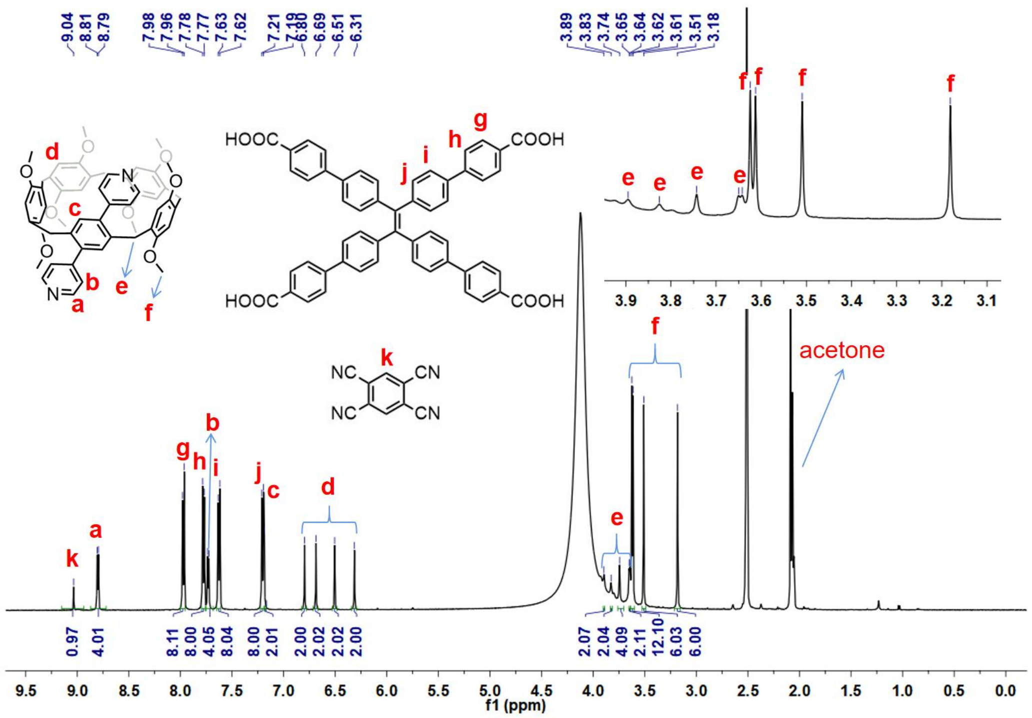

Supplementary Fig. 100 ^{1}H NMR (500 MHz, DMSO-d_6:DCl = 100:1, 298 K) spectrum of **MeP5-MOF-1** after digestion following uptake of **TCN**.

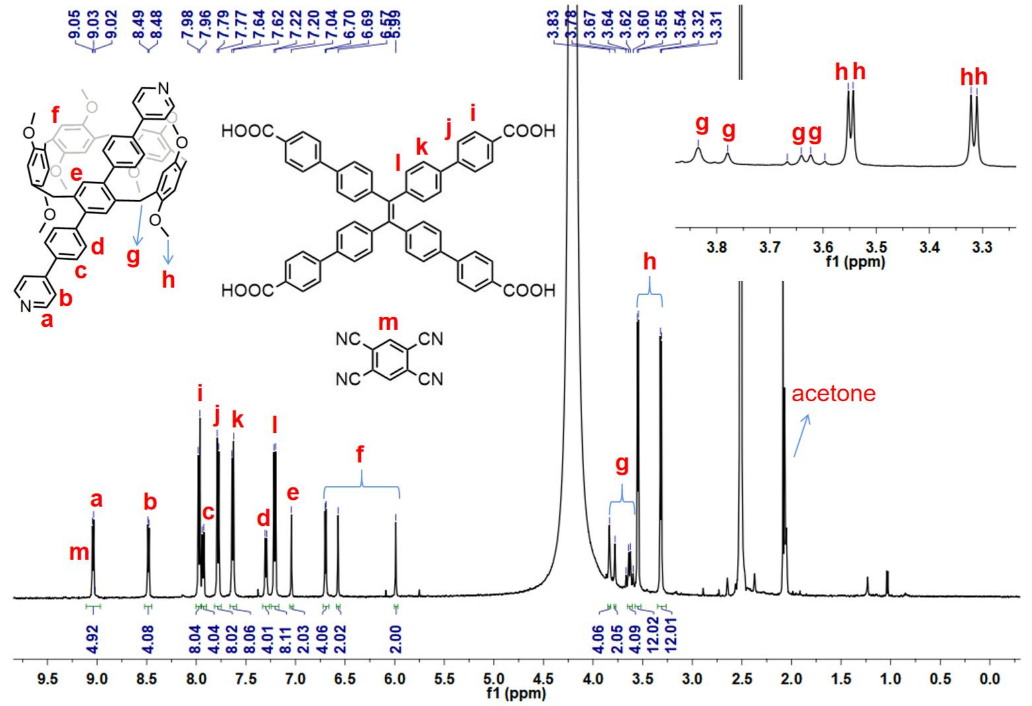

Supplementary Fig. 101 ^{1}H NMR (500 MHz, DMSO-d_6:DCl = 100:1, 298 K) spectrum of **MeP5-MOF-2** after digestion following uptake of **TCN**. Here the signal of proton **m** is merged into the signal of **a** at 9.00–9.10 ppm.

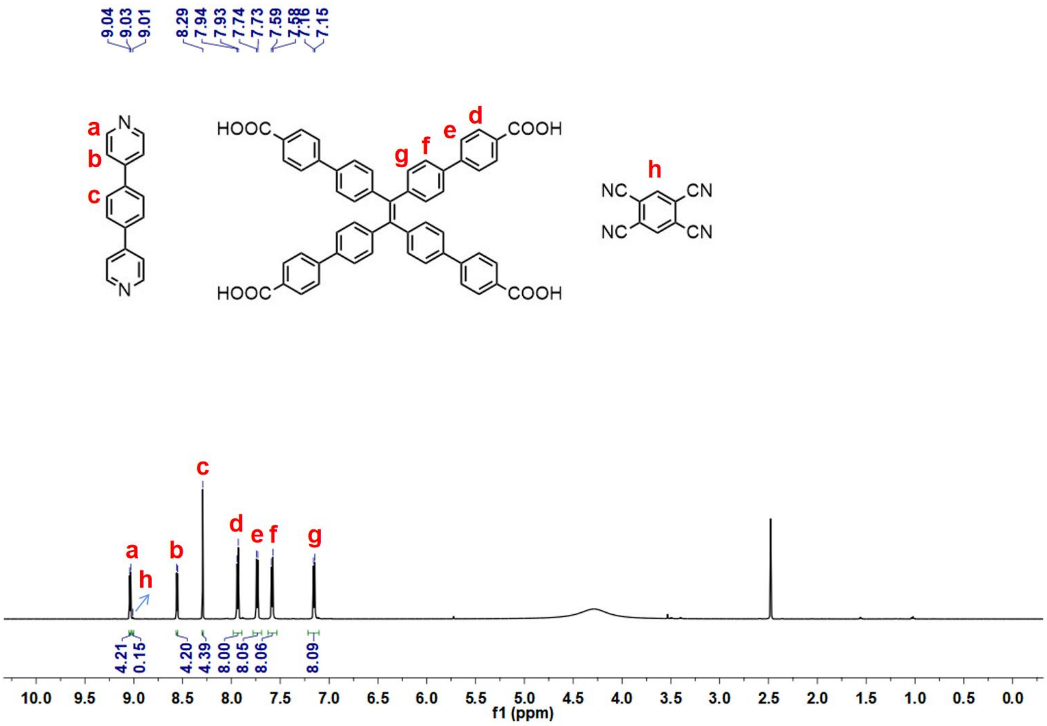

Supplementary Fig. 102 ^{1}H NMR (500 MHz, DMSO-d_6:DCl = 100:1, 298 K) spectrum of **Model-MOF-1** after digestion following uptake of **TCN**.

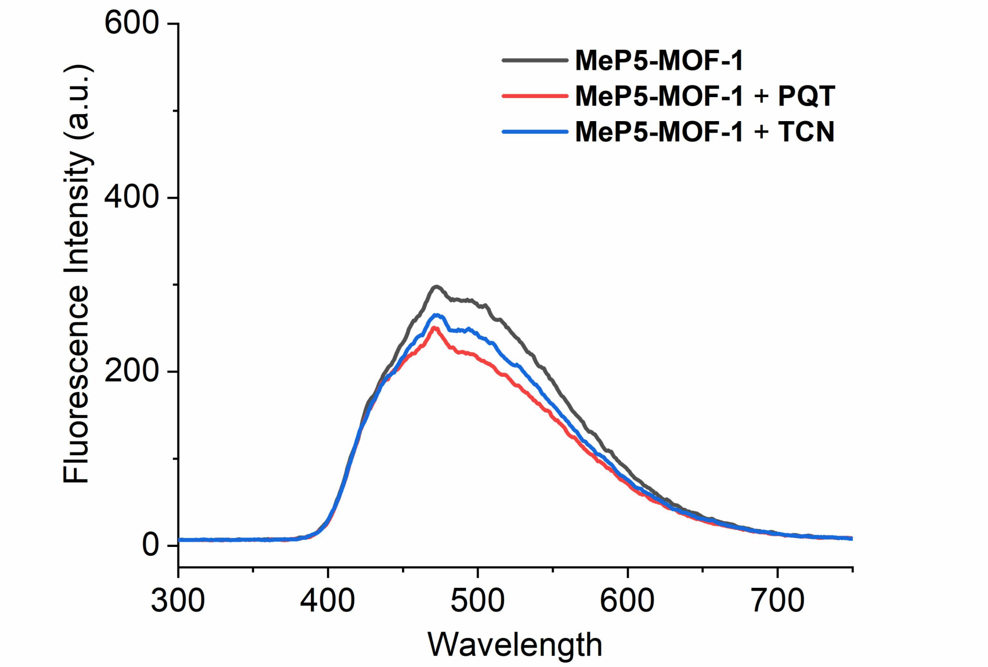

Supplementary Fig. 103 Solid-state fluorescence spectra of **MeP5-MOF-1** (~2 mg, black) before and after adding **PQT** (20.0 μM, red) or **TCN** (20.0 μM, blue) in acetone at room temperature, λ_{ex} = 250 nm.

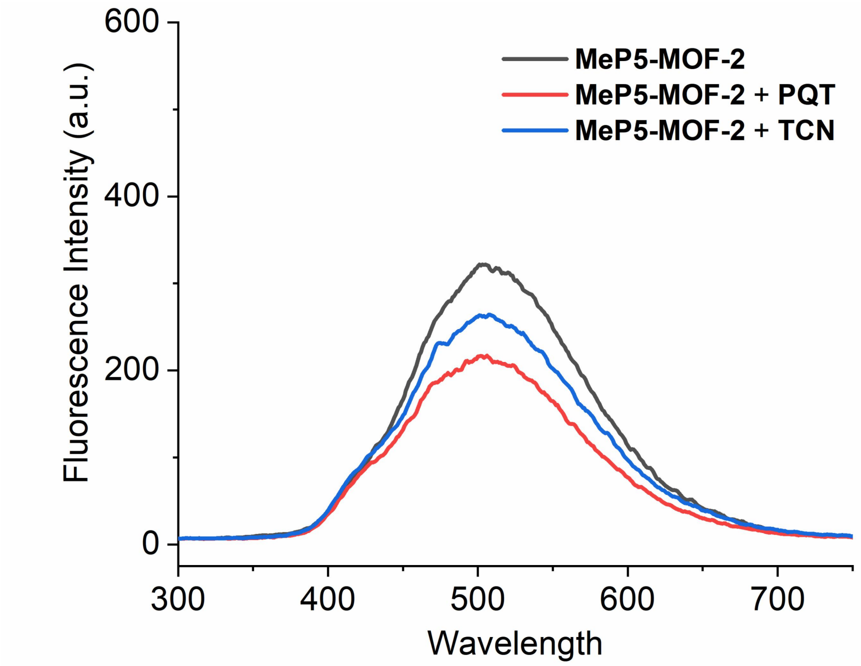

Supplementary Fig. 104 Solid-state fluorescence spectra of **MeP5-MOF-2** (~2 mg, black) before and after adding **PQT** (20.0 μM, red) or **TCN** (20.0 μM, blue) in acetone at room temperature, λ_{ex} = 250 nm.

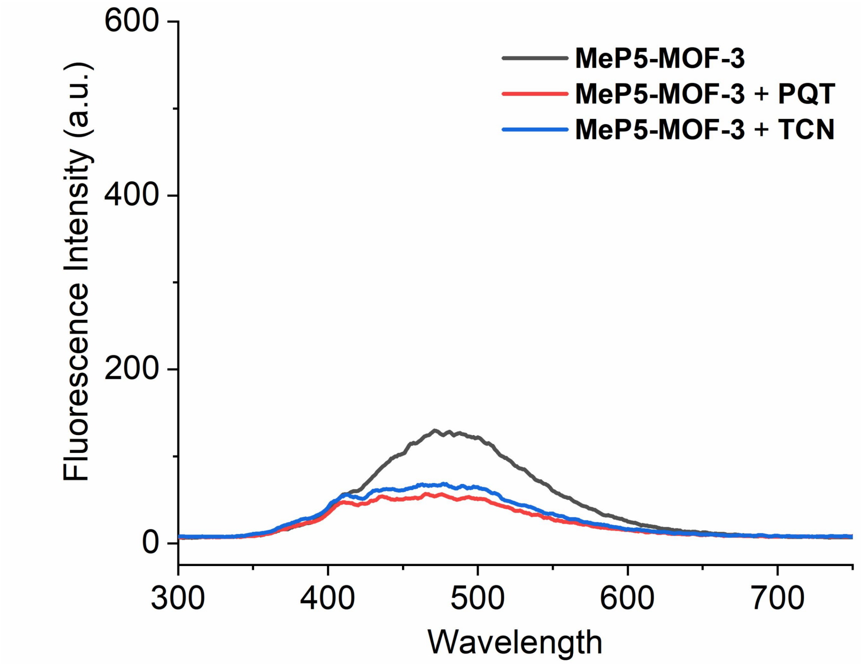

Supplementary Fig. 105 Solid-state fluorescence spectra of **MeP5-MOF-3** (~2 mg, black) before and after adding **PQT** (20.0 μM, red) or **TCN** (20.0 μM, blue) in acetone at room temperature, λ_{ex} = 250 nm.

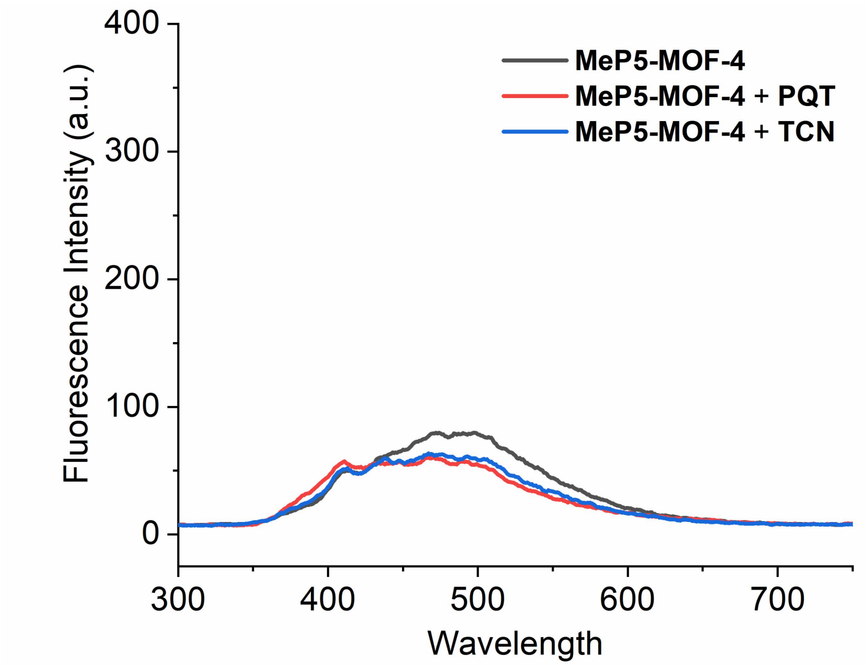

Supplementary Fig. 106 Solid-state fluorescence spectra of **MeP5-MOF-4** (~2 mg, black) before and after adding **PQT** (20.0 μM, red) or **TCN** (20.0 μM, blue) in acetone at room temperature, λ_{ex} = 250 nm.

11. Molecular separation studies of MOFs

Supplementary Table 18 Physical properties of **Tol** and **Py**[S14]

Substance	Melting point (°C)	Boiling point (°C)	Saturated vapor pressure at 298 K (kPa)
**Tol**	–94.9	110.60	3.9
**Py**	–41.6	115.50	2.8

11.1. Uptake from Tol and Py by MeP5-MOF-1

Prior to carrying out solid–liquid mixture experiments, the mother liquor associated with as-synthesized **MeP5-MOF-1** material was decanted off. The resulting crystals were washed with DMF (10 mL) five times and subject to solvent exchange with acetone (10 mL) five times. The crystals obtained in this way were then activated at room temperature under a nitrogen stream for 12 h. This procedure yielded ~20 mg of the **MeP5-MOF-1** adsorbent that was placed in a sealed 2 mL vial containing 100 μL of a 90:10 or 99:1 *v/v* **Tol**/**Py** mixture and allowed to stand for two minutes. The adsorbent was filtered off, and dried under air at 45 °C for one hour to remove the adsorbed molecules on the crystal surfaces. The solid material was then digested in

DMSO-d_6/DCl (100:1 *v*/*v*) for NMR measurements. The relative uptake of **Tol** or **Py** by **MeP5-MOF-1** was measured by calculating the mole ratios of **Tol** or **Py** relative to the struts which as determined from the integrations of the corresponding ^{1}H NMR spectral signals (500 MHz, 298 K). Quantitative analyses were performed by heating the crystals to release the adsorbed guest molecules and analyzing the volatiles using gas chromatography.

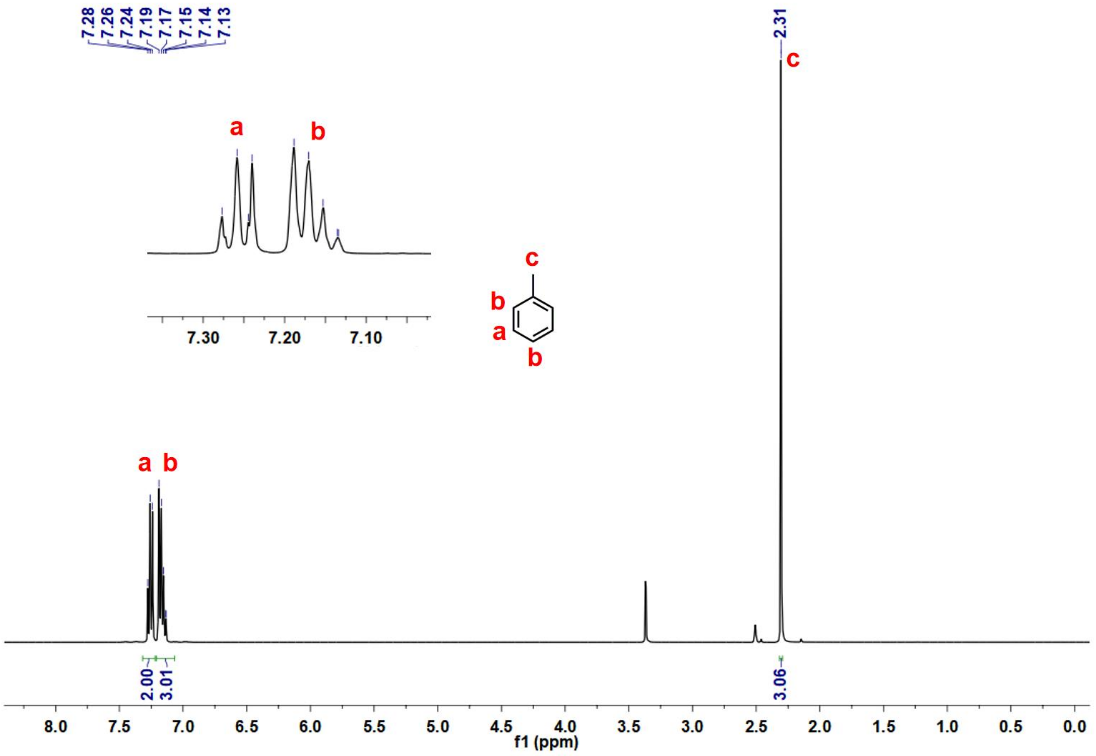

Supplementary Fig. 107 ^{1}H NMR spectrum (500 MHz, DMSO-d_6, 298 K) of **Tol**.

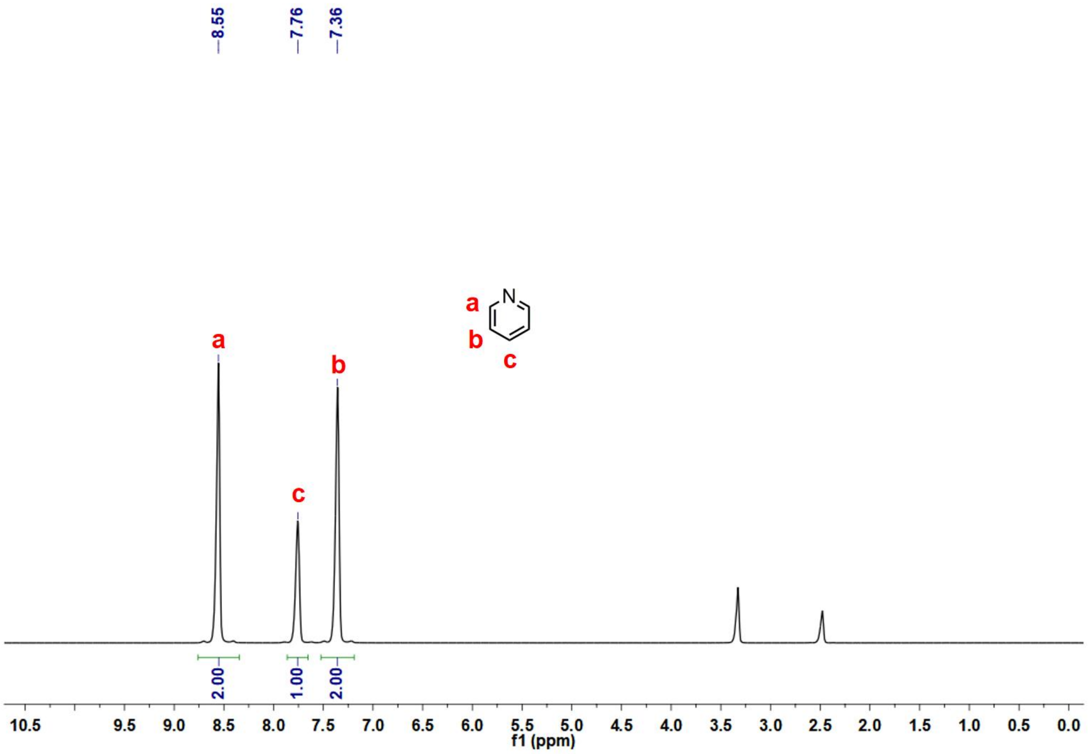

Supplementary Fig. 108 ^{1}H NMR spectrum (500 MHz, DMSO-d_6, 298 K) of **Py**.

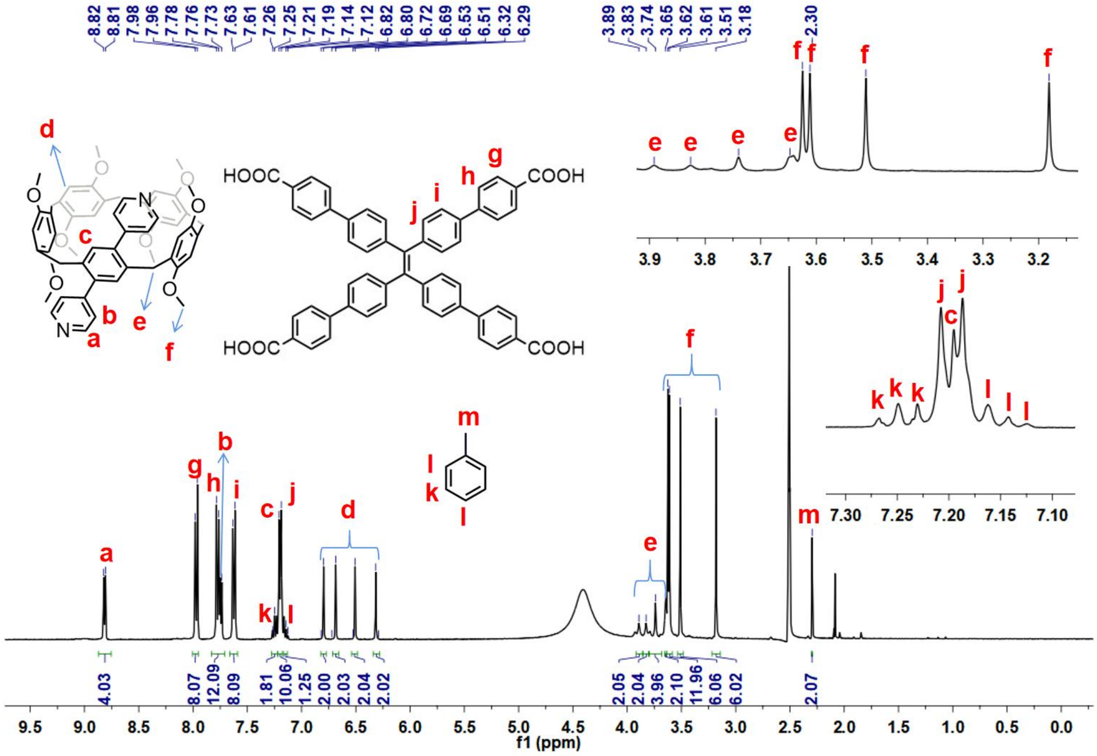

Supplementary Fig. 109 ^{1}H NMR spectrum (500 MHz, DMSO-d_6:DCl = 100:1, 298 K) of **MeP5-MOF-1** after immersing in **Tol** for two minutes.

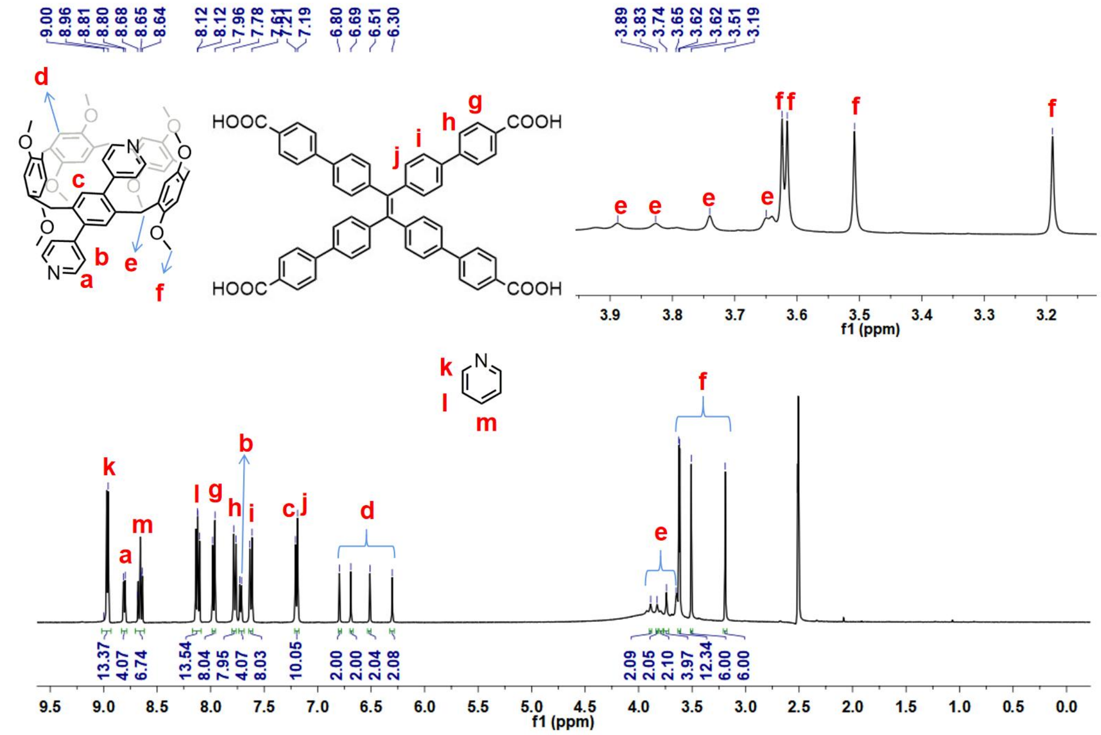

Supplementary Fig. 110 ^{1}H NMR spectrum (500 MHz, DMSO-d_6:DCl = 100:1, 298 K) of **MeP5-MOF-1** after immersing in **Py** for two minutes.

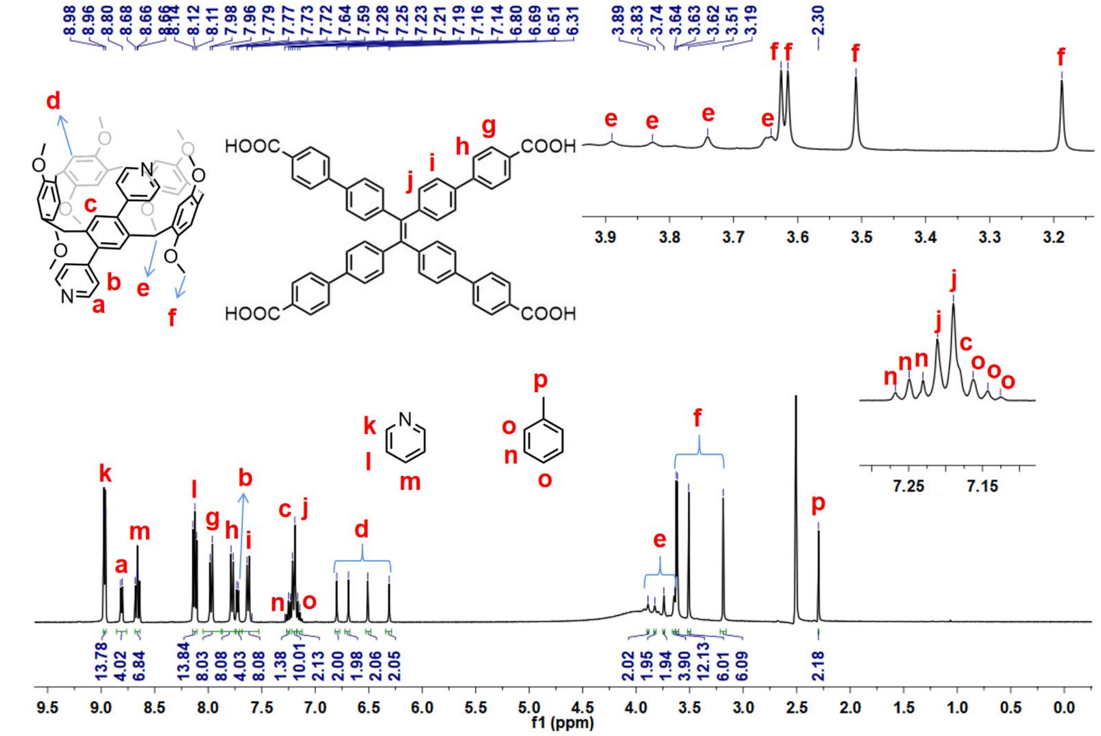

Supplementary Fig. 111 ^{1}H NMR spectrum (500 MHz, DMSO-d_6:DCl = 100:1, 298 K) of **MeP5-MOF-1** after ~20 mg of crystals were put in 100 µL of a 90:10 *v/v* **Tol**/**Py** mixture for two minutes.

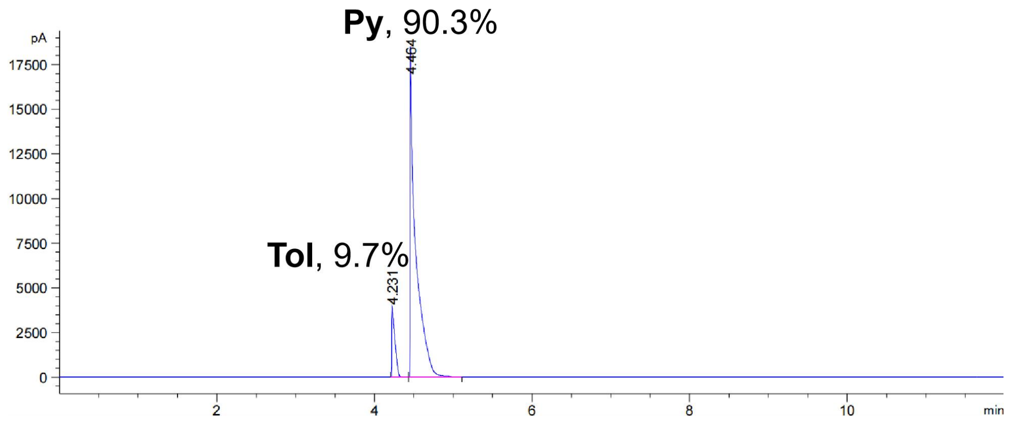

Supplementary Fig. 112 GC measurements of the relative uptake of **Tol**/**Py** in **MeP5-MOF-1** after ~20 mg of crystals were put in 100 μL of a 90:10 *v*/*v* **Tol**/**Py** mixture for two minutes.

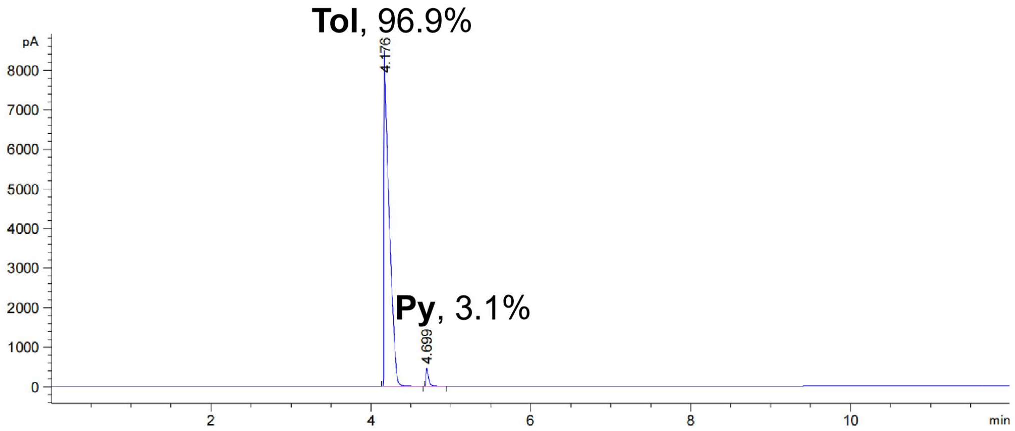

Supplementary Fig. 113 GC measurements of 100 μL of the residual 90:10 *v*:*v* (87.3:12.7 in mole percentage) **Tol**/**Py** mixture after first purification by adding ~20 mg of crystals of **MeP5-MOF-1**. The mole percentage of **Tol** increased from 87.3% to 96.9% during the course of this experiment.

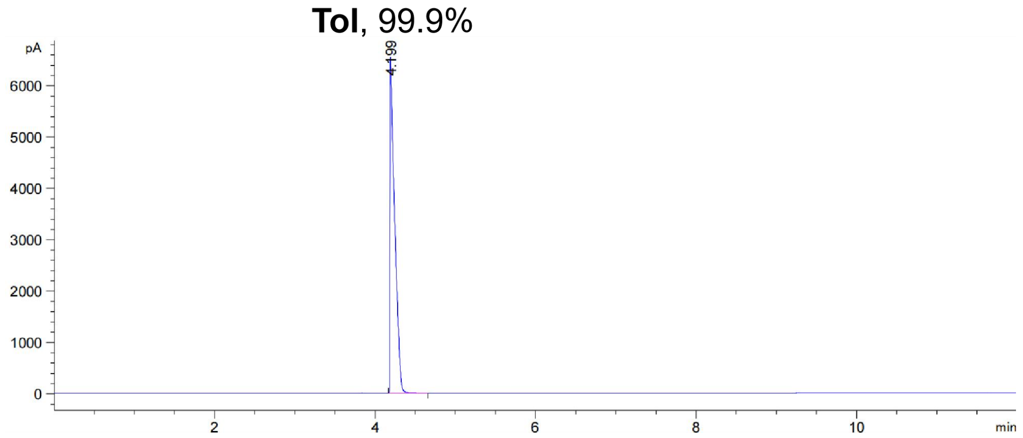

Supplementary Fig. 114 GC measurements of 100 μL of the residual 90:10 *v*:*v* **Tol**/**Py** mixture after second purification by adding another ~20 mg of fresh crystals of **MeP5-MOF-1**. The mole percentage of **Tol** increased from 96.9% to 99.9% during the course of this experiment.

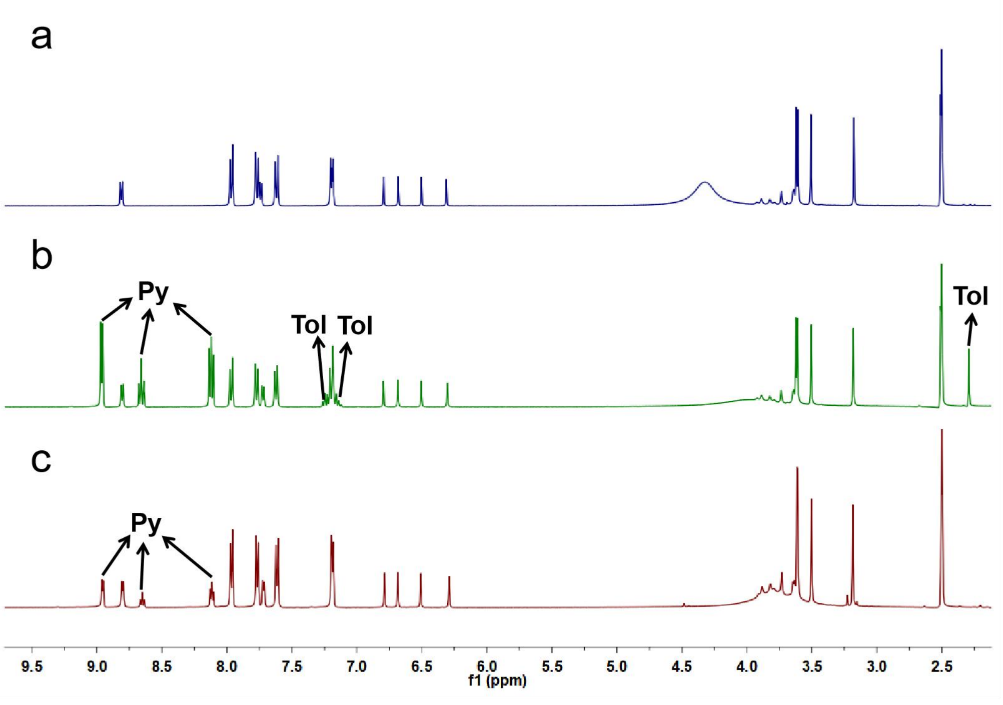

Supplementary Fig. 115 ^{1}H NMR spectra (500 MHz, DMSO-d_6:DCl = 100:1, 298 K): (a) initial **MeP5-MOF-1**; (b) initial **MeP5-MOF-1** after immersing in a 90:10 *v/v* **Tol**/**Py** mixture; (c) recycled **MeP5-MOF-1** wahsed with acetone (10 mL) five times.

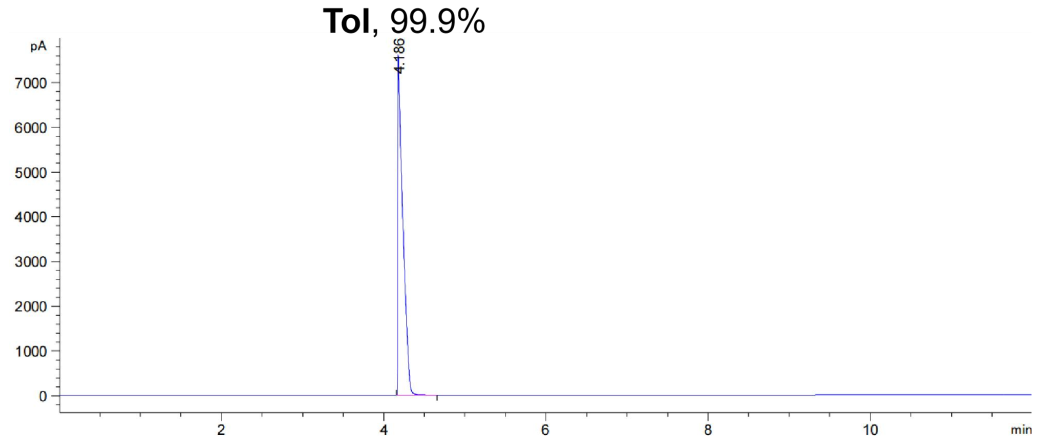

Supplementary Fig. 116 GC measurements of 100 μL of the residual 99:1 *v*:*v* (98.7:1.3 in mole percentage) **Tol** and **Py** mixture after purification by adding ~20 mg of fresh crystals of **MeP5-MOF-1**. The mole percentage of **Tol** increased from 98.7% to 99.9% during the course of this experiment.

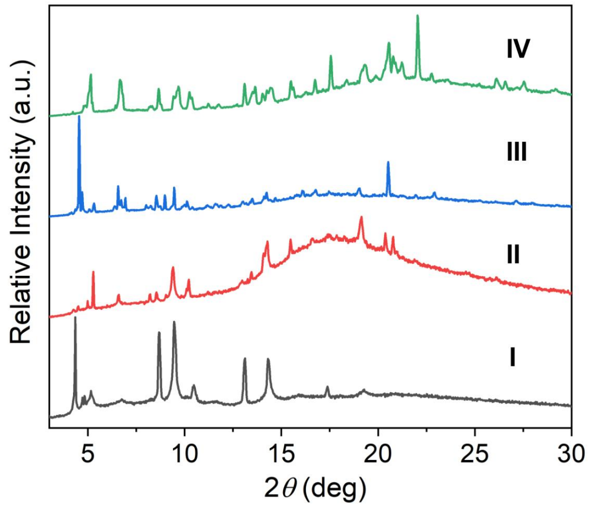

Supplementary Fig. 117 Experimental PXRD patterns of **MeP5-MOF-1**: **I**, as-synthesized samples; **II**, after uptake of **Py**; **III**, after uptake of **Tol**; **IV**, after uptake of a 90:10 *v*/*v* **Tol**/**Py** mixture. The various PXRD patterns after uptake of guests are considered reflective of the dynamics within the MOFs.

11.2. Uptake from Tol and Py by MeP5-MOF-2

Prior to the solid–liquid experiments, the mother liquor was decanted off from the crystals of the as-synthesized **MeP5-MOF-2**. The crystals were washed with DMF (10 mL) five times and subject to solvent exchange with acetone (10 mL) five times. The crystals obtained in this way were then activated at room temperature under a nitrogen stream for 12 h. This yielded ~20 mg of the **MeP5-MOF-2** adsorbent, which

was placed in a sealed 2 mL vial containing 100 µL of a 90:10 *v/v* **Tol**/**Py** mixture and allowed to stand for two minutes. The adsorbent was filtered off, and dried under air at 45 °C for one hour to remove adsorbed molecules on the crystal surfaces. The sample was then digested in DMSO-d_6/DCl (100:1 *v*/*v*) for NMR measurements. The relative uptake of **Tol** or **Py** by **MeP5-MOF-2** was measured by calculating the mole ratios of **Tol** or **Py** relative to the struts which as determined *via* integrations of the corresponding ^{1}H NMR (500 MHz, 298 K) signals. Quantitative analyses were performed by gas chromatography after heating the crystals to release the adsorbed guest molecules.

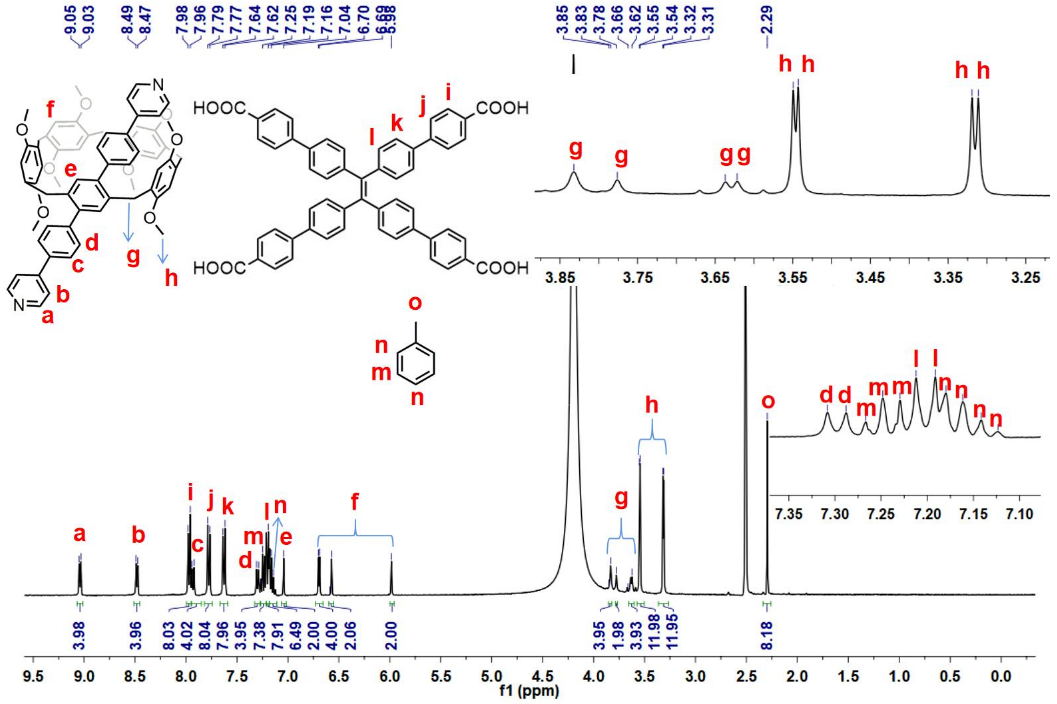

Supplementary Fig. 118 ^{1}H NMR spectrum (500 MHz, DMSO-d_6:DCl = 100:1, 298 K) of **MeP5-MOF-2** after immersing in **Tol** for two minutes.

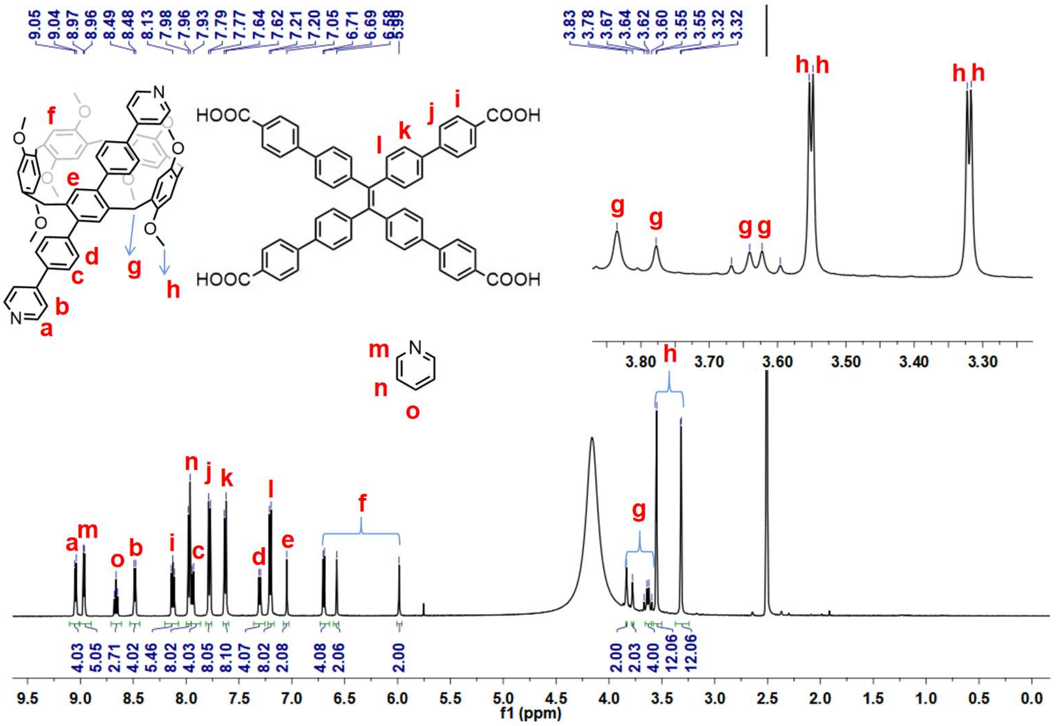

Supplementary Fig. 119 ^{1}H NMR spectrum (500 MHz, DMSO-d_6:DCl = 100:1, 298 K) of **MeP5-MOF-2** after immersing in **Py** for two minutes.

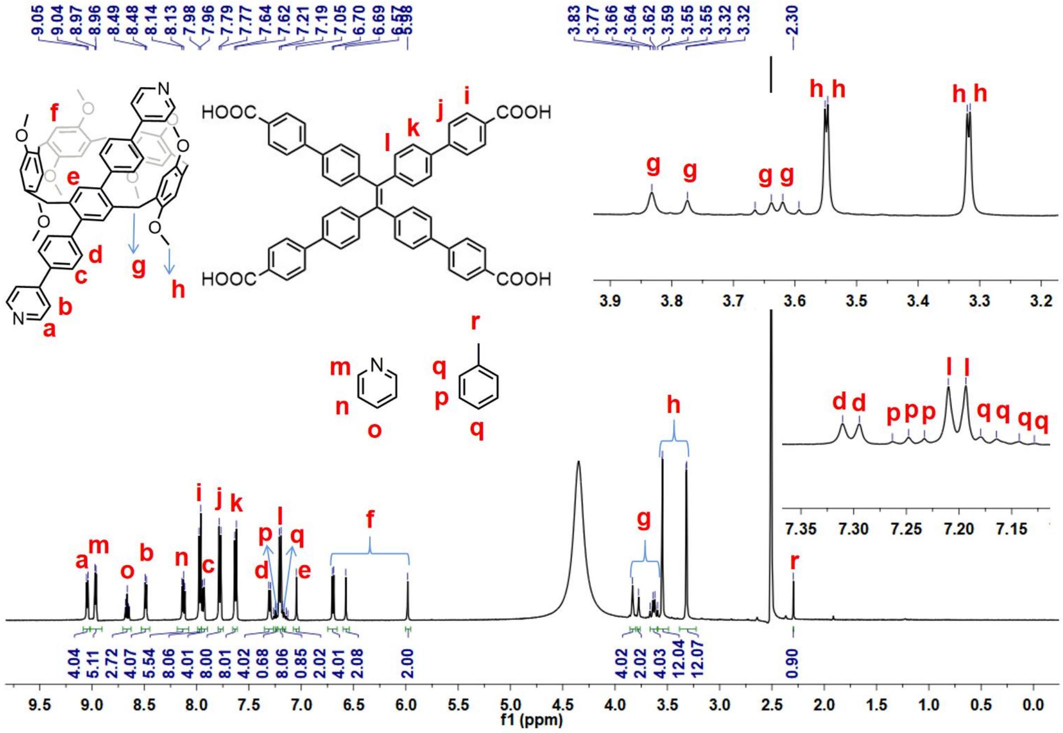

Supplementary Fig. 120 ^{1}H NMR spectrum (500 MHz, DMSO-d_6:DCl = 100:1, 298 K) of **MeP5-MOF-2** after ~20 mg of crystals were put in 100 μL of a 90:10 *v/v* **Tol**/**Py** mixture for two minutes.

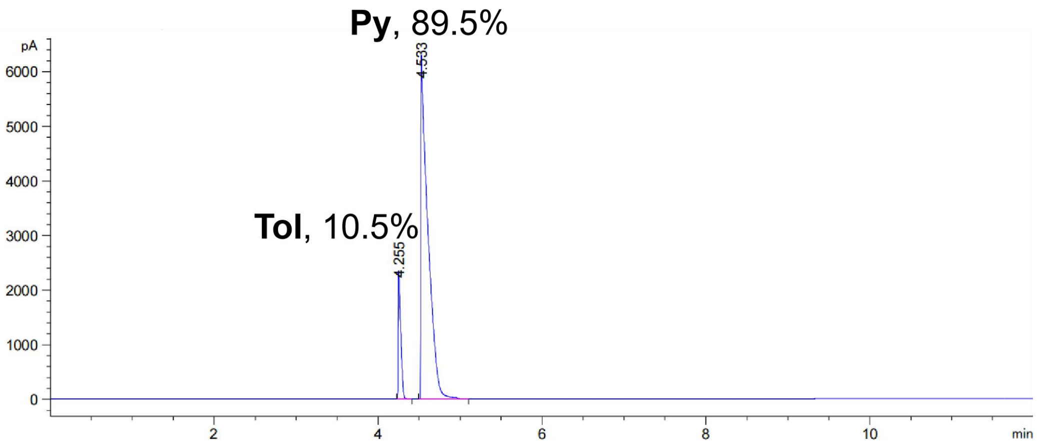

Supplementary Fig. 121 GC measurements of the relative uptake of **Tol**/**Py** in **MeP5-MOF-2** after ~20 mg of crystals were put in 100 μL of a 90:10 *v*/*v* **Tol**/**Py** mixture for two minutes.

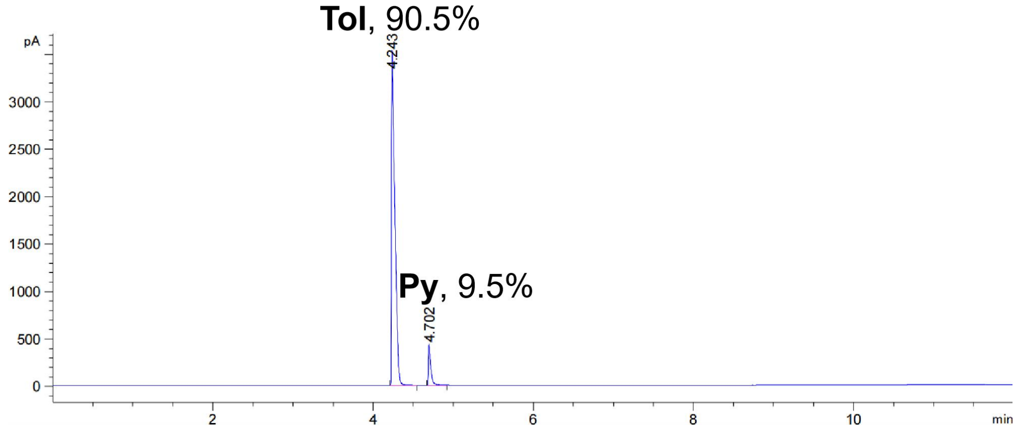

Supplementary Fig. 122 GC measurements of 100 μL of the residual 90:10 *v*:*v* (87.3:12.7 in mole percentage) **Tol**/**Py** mixture after purification by adding ~20 mg crystals of **MeP5-MOF-2**. The mole percentage of **Tol** increased from 87.3% to 90.5% during the course of this experiment.

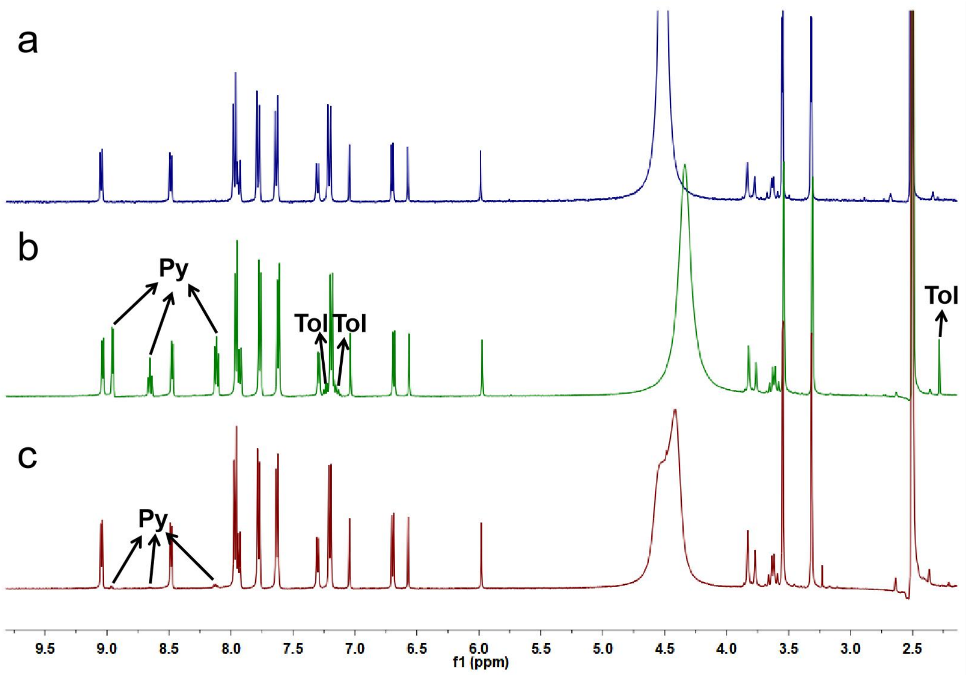

Supplementary Fig. 123 ^{1}H NMR spectra (500 MHz, DMSO-d_6:DCl = 100:1, 298 K): (a) initial **MeP5-MOF-2**; (b) initial **MeP5-MOF-2** after immersing in a 90:10 *v/v* **Tol**/**Py** mixture; (c) recycled **MeP5-MOF-2** washed with acetone (10 mL) five times.

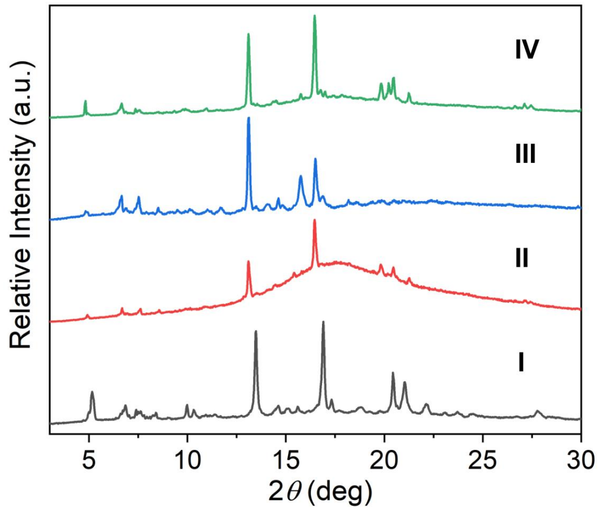

Supplementary Fig. 124 Experimental PXRD patterns of **MeP5-MOF-2**: **I**, as-synthesized samples; **II**, after uptake of **Py**; **III**, after uptake of **Tol**; **IV**, after uptake of a 90:10 *v/v* **Tol**/**Py** mixture. The various PXRD patterns after uptake of guests are considered reflective of the dynamics within the MOFs.

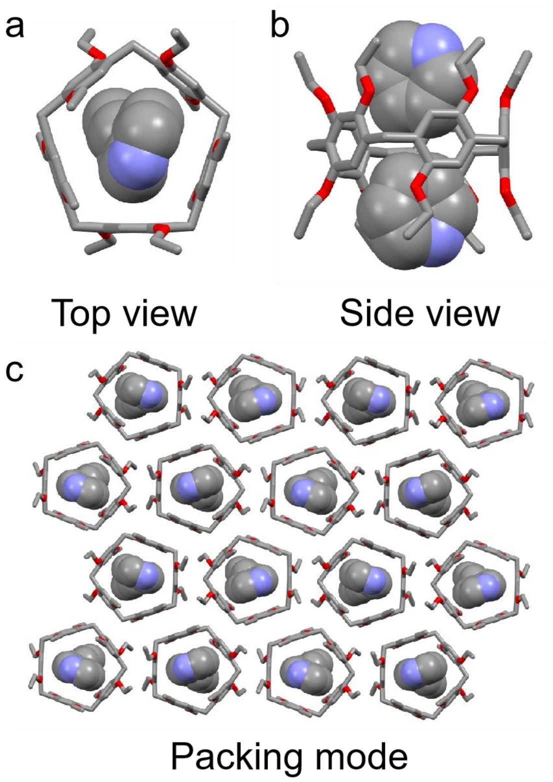

Supplementary Fig. 125 Capped-stick and spacefill representations of the single crystal structure of (**Py**)$_2$@**P5**. Carbon atoms are grey, oxygen atoms are red, and nitrogen atoms are blue. Hydrogen atoms are omitted for clarity.

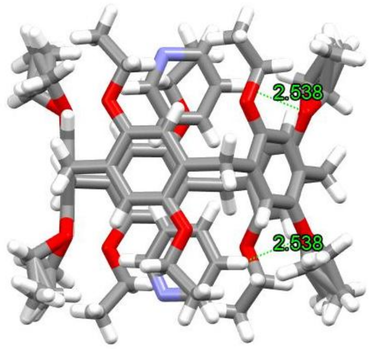

Supplementary Fig. 126 Capped-stick representation of the single crystal structure of (**Py**)$_2$@**P5**. Carbon atoms are grey, hydrogen atoms are white, oxygen atoms are red, and nitrogen atoms are blue. Hydrogen-bond parameters: [C···O] distances (Å), [H···O] distances (Å) and [C–H···O] angles (deg) of [C–H···O] hydrogen bonds, 3.42, 2.54, 154.88; 3.42, 2.54, 154.88.

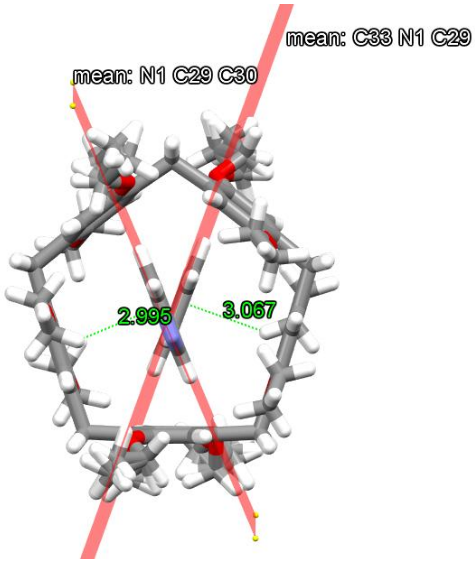

Supplementary Fig. 127 Capped-stick representation of the crystal structure of (**Py**)$_2$@**P5**. Carbon atoms are grey, hydrogen atoms are white, oxygen atoms are red, and nitrogen atoms are blue. [C–H···π] distances (Å) and angles (deg): 3.00, 158.34; 3.07, 158.76.

To predict the preferential location of pyridine molecules as guests within the frameworks, we carried out location simulations by using the sorption module of Material Studio[S9]. The Metropolis Monte Carlo method was selected for the calculation of the global minimum. The COMPASS force field was chosen for the energy calculations, and the charge equilibration method was used to calculate point atomic charges. The PXRD pattern was also predicted using the reflex module in Material Studio.

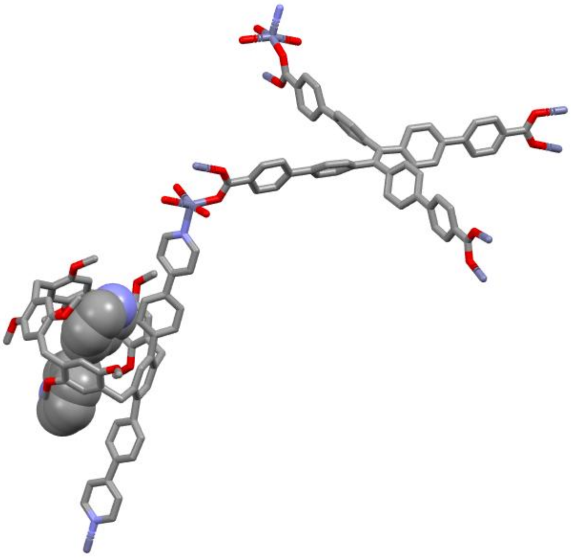

Supplementary Fig. 128 Capped-stick and spacefill representation of the calculated structure of **Py@MeP5-MOF-2**. Here, **Py** molecules are models based on the single crystal structures of **MeP5-MOF-2** and (**Py**)$_2$@**P5**. Carbon atoms are grey, oxygen atoms are red, nitrogen atoms are blue, and zinc atoms are dark blue. Hydrogen atoms are omitted for clarity.

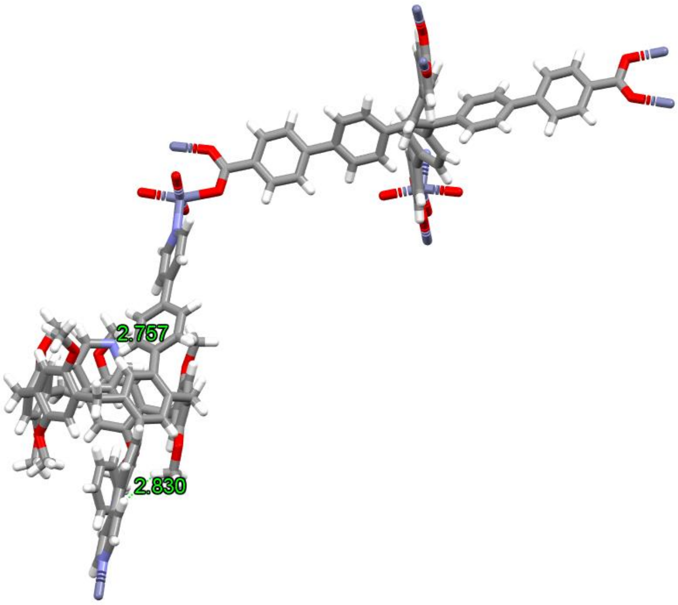

Supplementary Fig. 129 Capped-stick representation of the calculated structure of **Py@MeP5-MOF-2** obtained by "locate simulation" based on the single crystal structures of **MeP5-MOF-2** and (**Py**)$_2$@**P5**. Carbon atoms are grey, hydrogen atoms are white, oxygen atoms are red, nitrogen atoms are blue, and zinc atoms are dark blue. Hydrogen-bond parameters: [C···N] distances (Å), [H···N] distances (Å) and [C–H···N] angles (deg) of [C–H···N] hydrogen bonds, 3.60, 2.76, 144.96; 3.67, 2.83, 144.68.

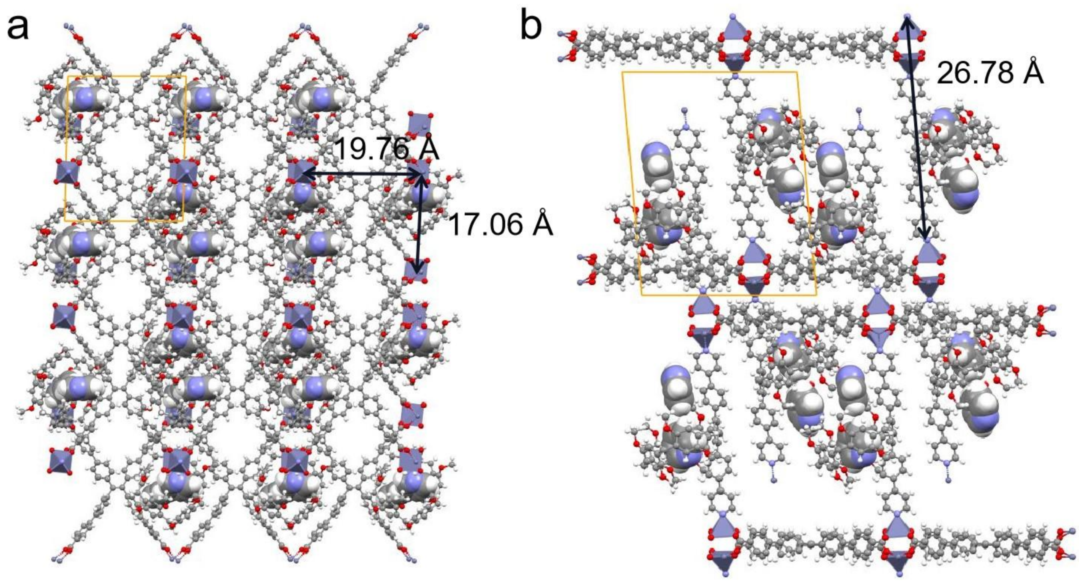

Supplementary Fig. 130 Polyhedral and spacefill representation of the calculated structure of **Py@MeP5-MOF-2** along the *a* axis (a) and the *c* axis (b). The elementary cell is marked with an orange cuboid (a = 19.76 Å, b = 17.06 Å, c = 26.78 Å). Carbon atoms are grey, hydrogen atoms are white, oxygen atoms are red, nitrogen atoms are blue, and zinc atoms are dark blue.

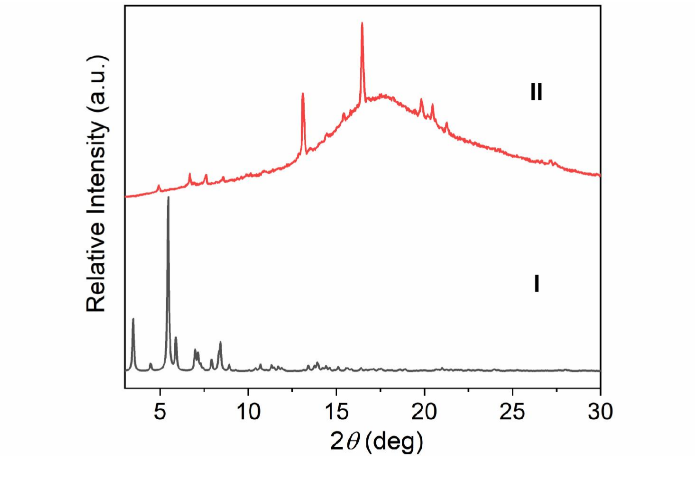

Supplementary Fig. 131 PXRD patterns of **Py@MeP5-MOF-2**: **I**, simulated from the calculated structure; **II**, from a single crystal sample. Here the mismatching of experimental and calculated PXRD patterns is considered reflective of the dynamics within **MeP5-MOF-2** after **Py** uptake.

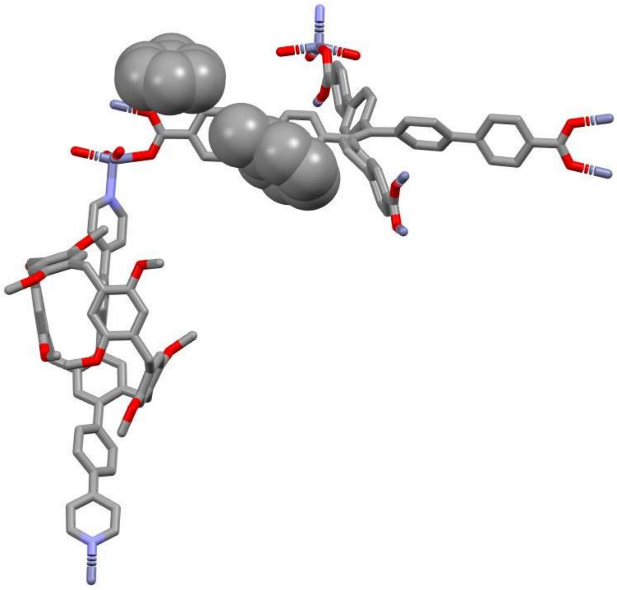

Supplementary Fig. 132 Capped-stick and spacefill representation of the single crystal structure of **Tol@MeP5-MOF-2**. The occupancy of **Tol** molecules in **MeP5-MOF-2** is 0.5. Carbon atoms are grey, oxygen atoms are red, nitrogen atoms are blue, and zinc atoms are dark blue. Hydrogen atoms are omitted for clarity.

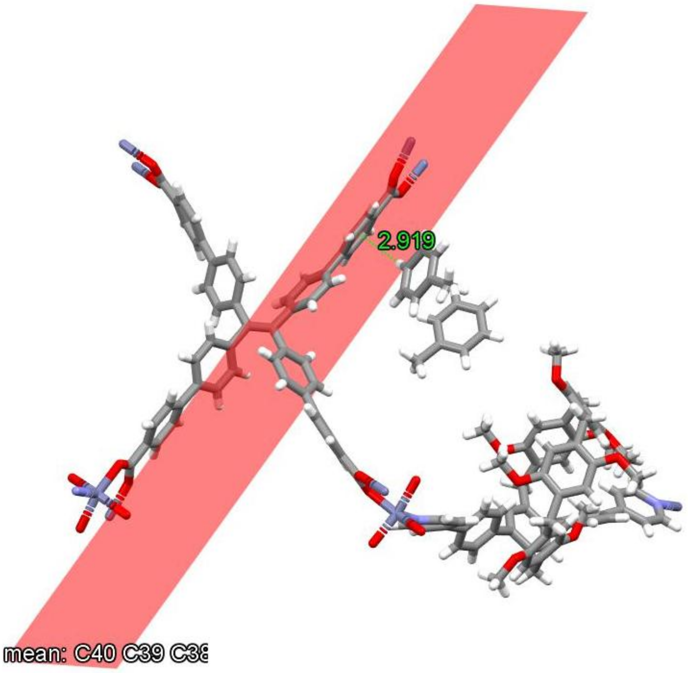

Supplementary Fig. 133 Capped-stick representation of the single crystal structure of **Tol@MeP5-MOF-2**. Carbon atoms are grey, hydrogen atoms are white, oxygen atoms are red, nitrogen atoms are blue, and zinc atoms are dark blue. [C–H···π] distance (Å) and angle (deg): 2.92, 143.38.

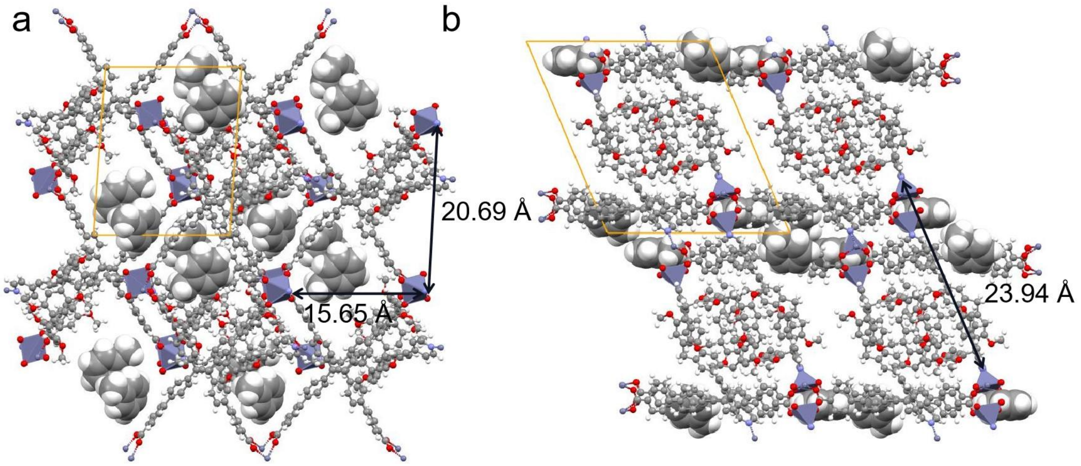

Supplementary Fig. 134 Polyhedral and spacefill representation of the single crystal structure of **Tol@MeP5-MOF-2** along the *a* axis (a) and the *c* axis (b). The elementary cell is marked with an orange cuboid (a = 20.69 Å, b = 15.65 Å, c = 23.94 Å). Carbon atoms are grey, hydrogen atoms are white, oxygen atoms are red, nitrogen atoms are blue, and zinc atoms are dark blue.

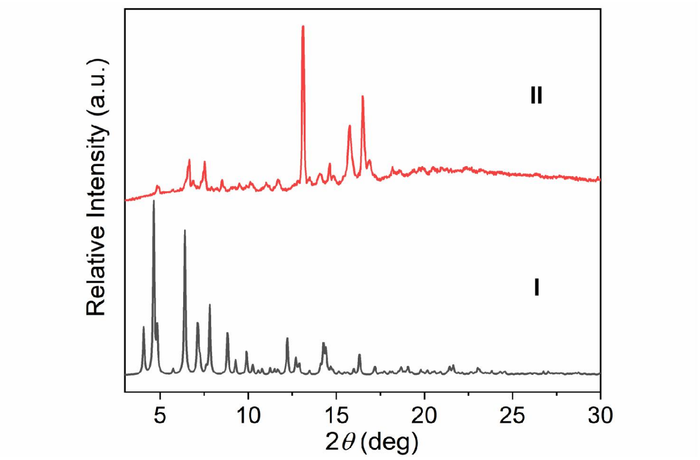

Supplementary Fig. 135 PXRD patterns of **Tol@MeP5-MOF-2**: **I**, simulated from the single crystal structure; **II**, from a single crystal sample. Here the mismatching of experimental and simulated PXRD patterns are considered reflective of the dynamics within **MeP5-MOF-2** after uptake of **Tol** molecules.

11.3. Uptake from Tol and Py by Model-MOF-1

Prior to carrying out solid–liquid experiments, the mother liquor was decanted off from the crystals of as-synthesized **MeP5-MOF-1**. The resulting crystals were washed with DMF (10 mL) five times and solvent exchanged with acetone (10 mL) five times. The crystals obtained in this way were then activated at room temperature

under a nitrogen stream for 12 h. This gave ~20 mg of the **Model-MOF-1** adsorbent, which was placed in a sealed 2 mL vial containing 100 μL of a 90:10 *v/v* **Tol**/**Py** mixture and allowed to sit for two minutes. The adsorbent was filtered off, and dried under air at 45 °C for one hour to remove adsorbed molecules on the crystal surfaces. It was then digested in DMSO-d_6/DCl (100:1 *v*/*v*) for NMR measurements. The relative uptake of **Tol** or **Py** by **Model-MOF-1** was measured by calculating the mole ratios of **Tol** or **Py** relative to the struts as determined from integrations of the corresponding ^{1}H NMR signals (500 MHz, 298 K). Quantitative analyes were performed by heating the crystals to release the adsorbed guest molecules and monitoring the volatiles by gas chromatography.

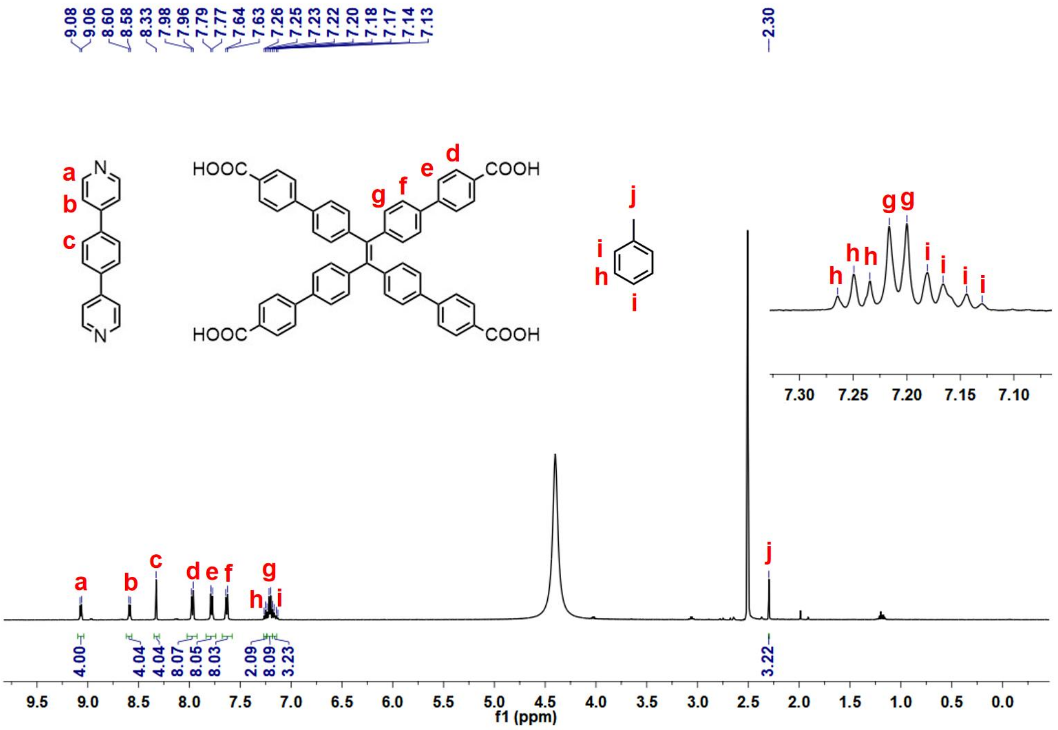

Supplementary Fig. 136 ^{1}H NMR spectrum (500 MHz, DMSO-d_6:DCl = 100:1, 298 K) of **Model-MOF-1** after immersing in **Tol** for two minutes.

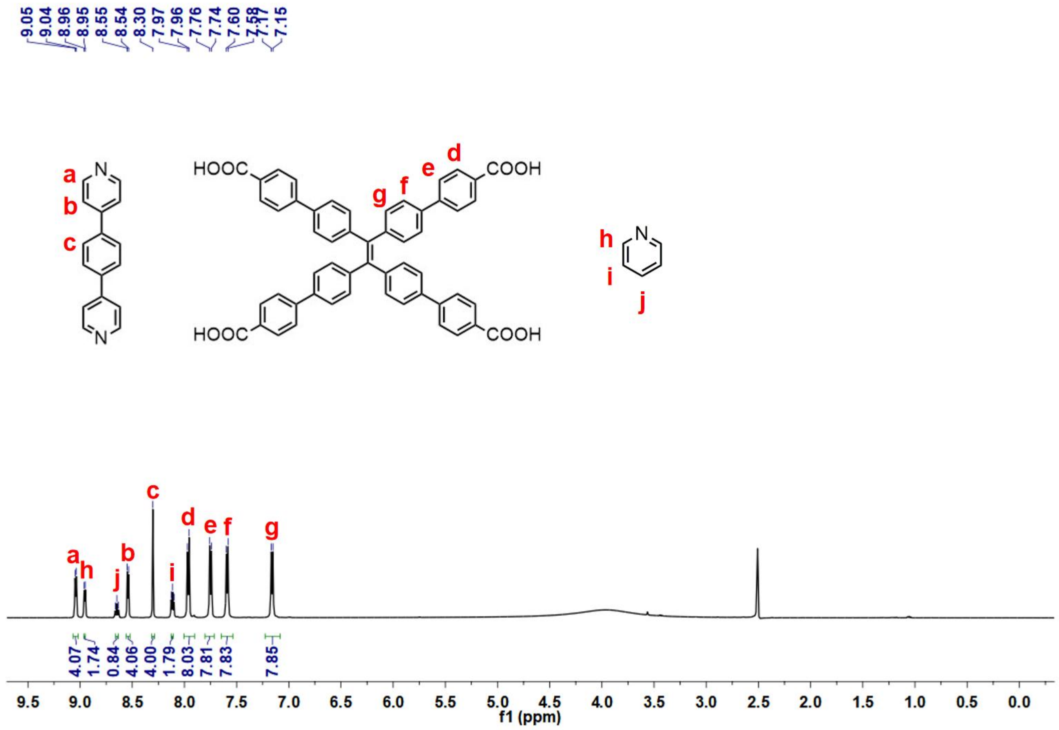

Supplementary Fig. 137 ^{1}H NMR spectrum (500 MHz, DMSO-d_6:DCl = 100:1, 298 K) of **Model-MOF-1** after immersing in **Py** for two minutes.

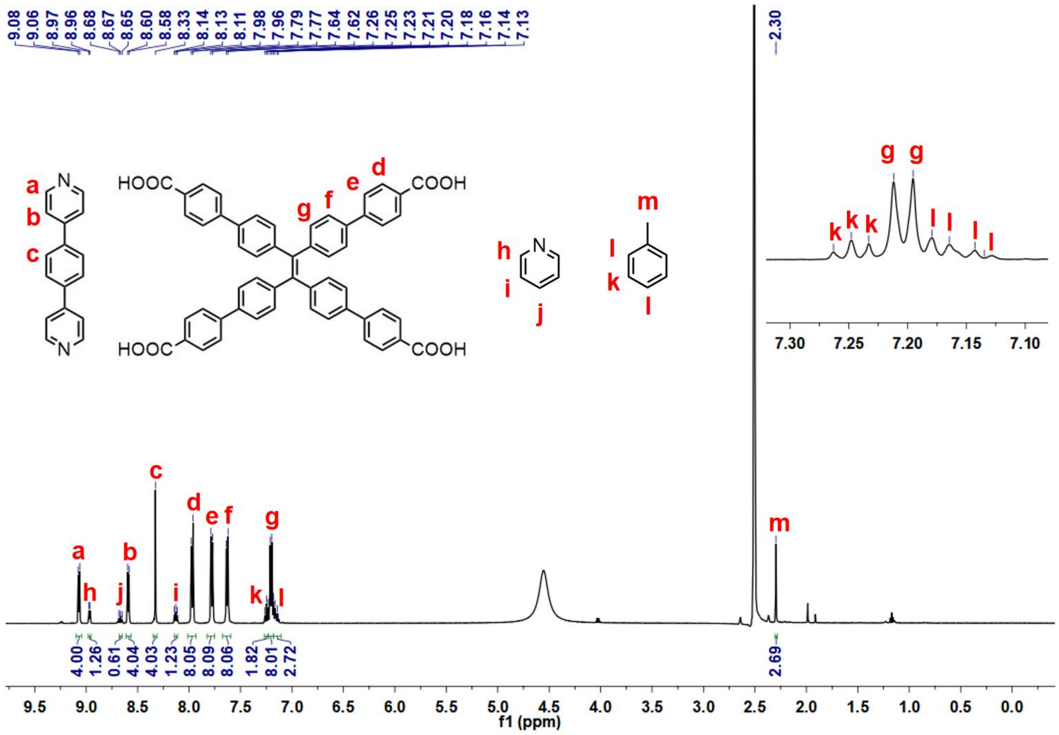

Supplementary Fig. 138 ^{1}H NMR spectrum (500 MHz, DMSO-d_6:DCl = 100:1, 298 K) of **Model-MOF-1** after ~20 mg of crystals were put in 100 μL of a 90:10 *v/v* **Tol**/**Py** mixture for two minutes.

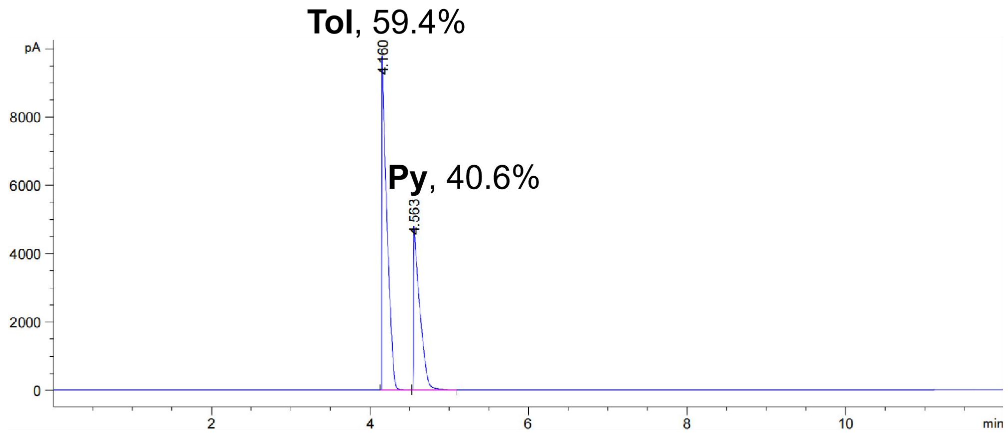

Supplementary Fig. 139 GC measurements of the relative uptake of **Tol**/**Py** in **Model-MOF-1** after ~20 mg of crystals were put in 100 μL of a 90:10 *v*/*v* **Tol**/**Py** mixture for two minutes.

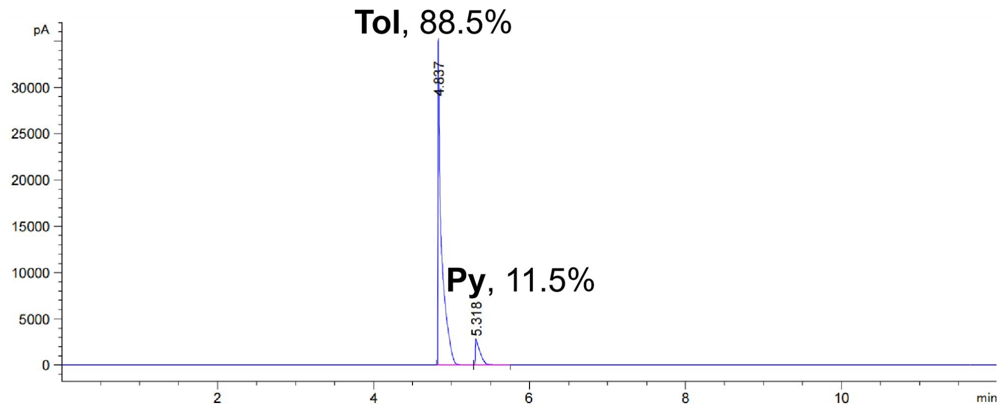

Supplementary Fig. 140 GC measurements of 100 μL of the residual 90:10 *v*:*v* (87.3:12.7 in mole percentage) **Tol**/**Py** mixture after purification by adding ~20 mg fresh crystals of **Model-MOF-1**.

11.4. Uptake from Tol and Py by MeP5

For solid–vapor mixture experiments, an open 5 mL vial containing 20.0 mg of the guest free **MeP5** adsorbent was placed in a sealed 20 mL vial containing 100 μL of a 90:10 *v*/*v* **Tol**/**Py** mixture. The adsorbent was air-dried at 45 °C for one hour to remove adsorbed molecules on the powder surfaces, The relative uptake of **Tol** or **Py** by **MeP5** was measured by calculating the mole ratios of **Tol** or **Py** to **MeP5** as determined from integrations of the corresponding ^{1}H NMR signals (500 MHz, 298 K). Quantitative analyses were performed by heating the crystals to release the adsorbed vapor molecules and monitoring the volatiles by gas chromatography.

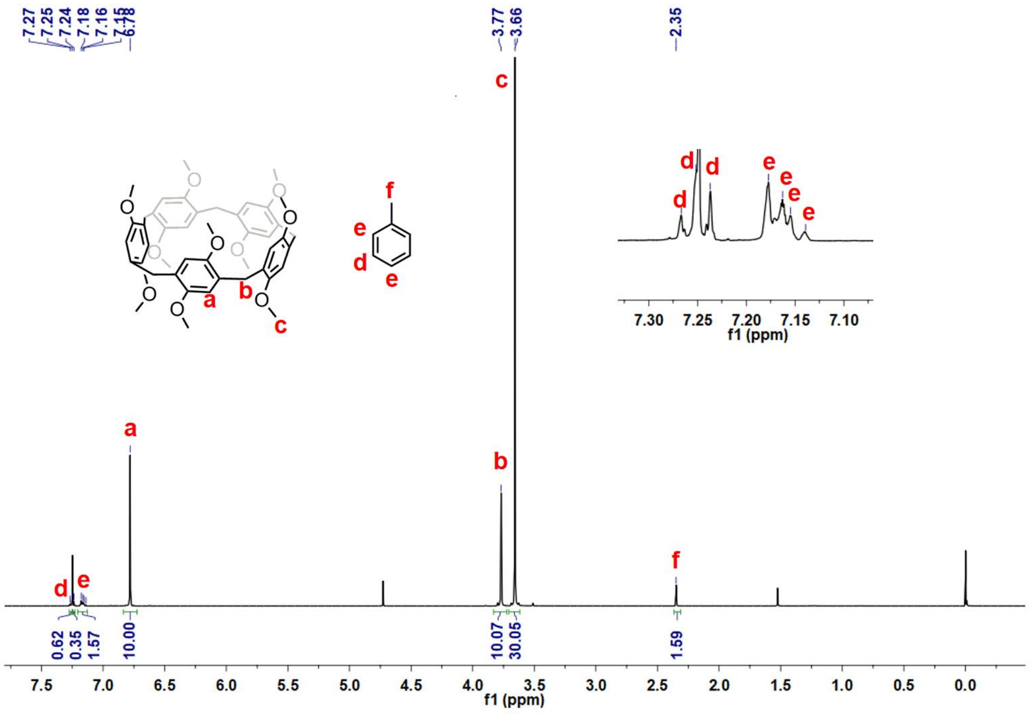

Supplementary Fig. 141 ^{1}H NMR spectrum (500 MHz, $CDCl_3$, 298 K) of **MeP5** after exposing to **Tol** vapor for 24 hours.

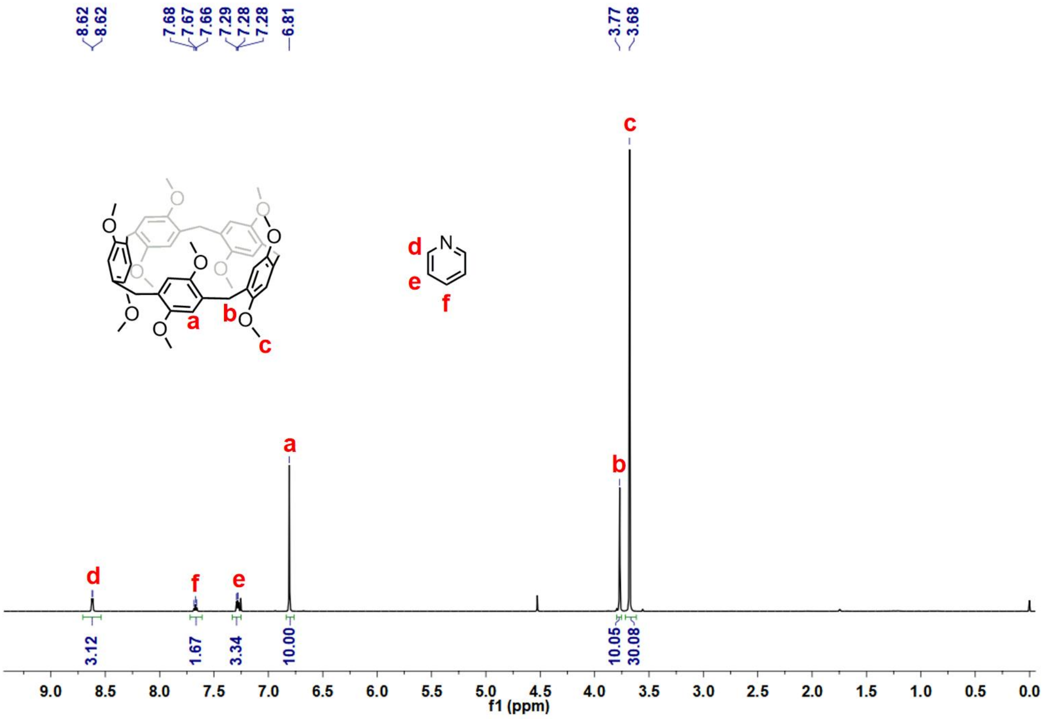

Supplementary Fig. 142 ^{1}H NMR spectrum (500 MHz, $CDCl_3$, 298 K) of **MeP5** after exposing to **Py** vapor for 24 hours.

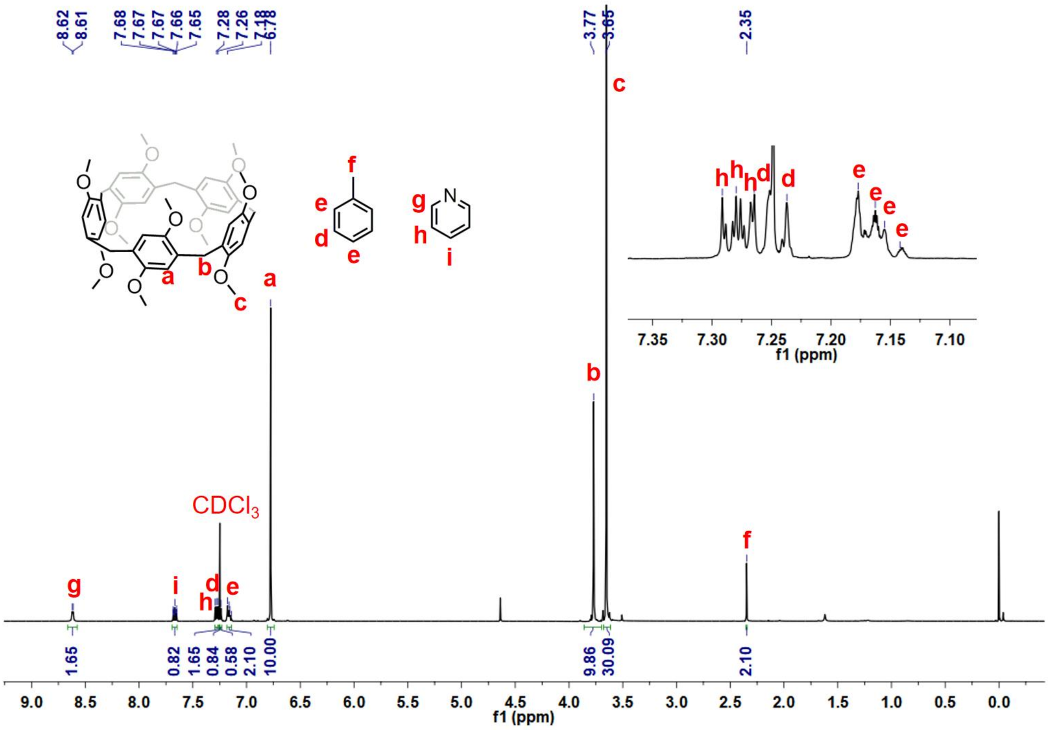

Supplementary Fig. 143 ^{1}H NMR spectrum (500 MHz, $CDCl_3$, 298 K) of **MeP5** after 20.0 mg of **MeP5** powder exposing to the vapor from a 100 μL aliquot of a 90:10 *v/v* **Tol**/**Py** mixture for 24 hours.

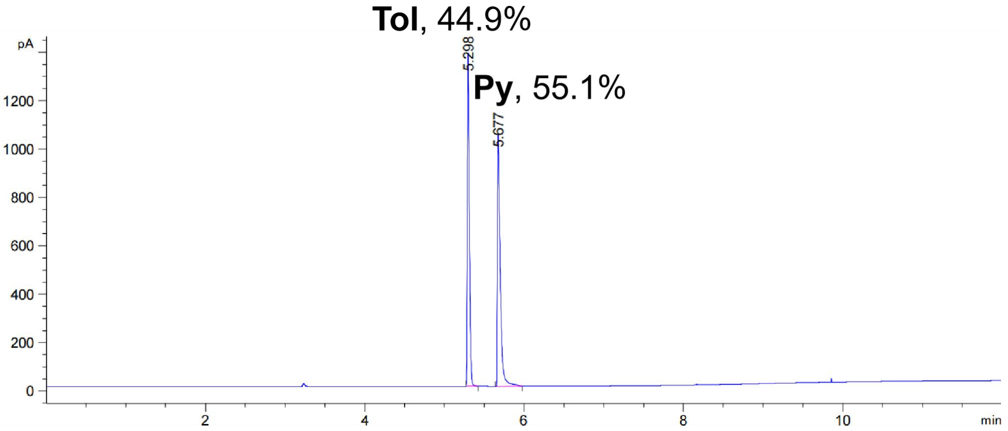

Supplementary Fig. 144 GC measurements of the relative uptake of **Tol**/**Py** in **MeP5** after 20.0 mg of **MeP5** powder exposing to the vapor from a 100 μL aliquot of a 90:10 *v/v* **Tol**/**Py** mixture for 24 hours.

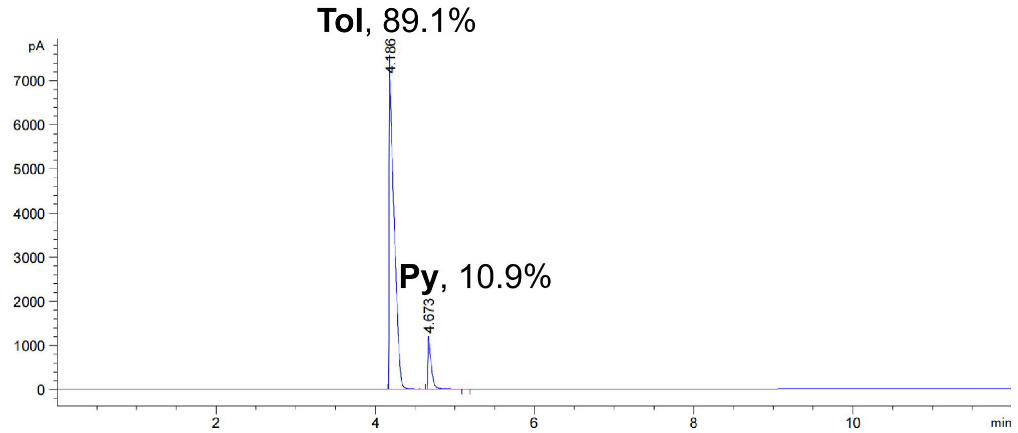

Supplementary Fig. 145 GC measurements of 100 μL of the residual 90:10 *v*:*v* (87.3:12.7 in mole ratio) **Tol**/**Py** mixture after exposing a 20.0 mg of **MeP5** powder to its vapor for 24 hours.

11.5. Uptake of Tol and Py by MeP5-MOF-3 and MeP5-MOF-4

Prior to the solid–liquid experiments, the mother liquor was decanted off from the crystals of the as-synthesized **MeP5-MOF-3** or **MeP5-MOF-4**. The crystals were washed with DMF (10 mL) five times and subject to solvent exchange with acetone (10 mL) five times. The crystals obtained in this way were then activated at room temperature under a nitrogen stream for 12 h. This yielded ~20 mg of the **MeP5-MOF-3** or **MeP5-MOF-4** adsorbent, which was placed in a sealed 2 mL vial containing 100 μL of a 90:10 *v/v* **Tol**/**Py** mixture and allowed to stand for two minutes. The adsorbents was filtered off, and dried under air at 45 °C for one hour to remove the adsorbed molecules on the crystal surfaces. Quantitative analyses were performed by gas chromatography after heating the crystals to release the adsorbed guest molecules.

Compared with **MeP5-MOF-1** and **MeP5-MOF-2**, the samples of **MeP5-MOF-3** and **MeP5-MOF-4** were obtained in lower yields and were characterized by lower crystal quality. Efforts to obtain diffraction grade single crystals always yielded polycrystalline materials or samples with cracks that made them not suitable for use in SCXRD analyses. The materials could be used for update studies. Here, the selectivities for **Py** of **MeP5-MOF-3** and **MeP5-MOF-4** in a 90:10 *v/v* **Tol**/**Py** mixture were investigated. The results revealed that both **MeP5-MOF-3** and **MeP5-MOF-4** showed selectivity for **Py** but were not as selective as **MeP5-MOF-1**

and **MeP5-MOF-2**. Based on these results, **MeP5-MOF-1** and **MeP5-MOF-2** were chosen for more in-depth separation studies.

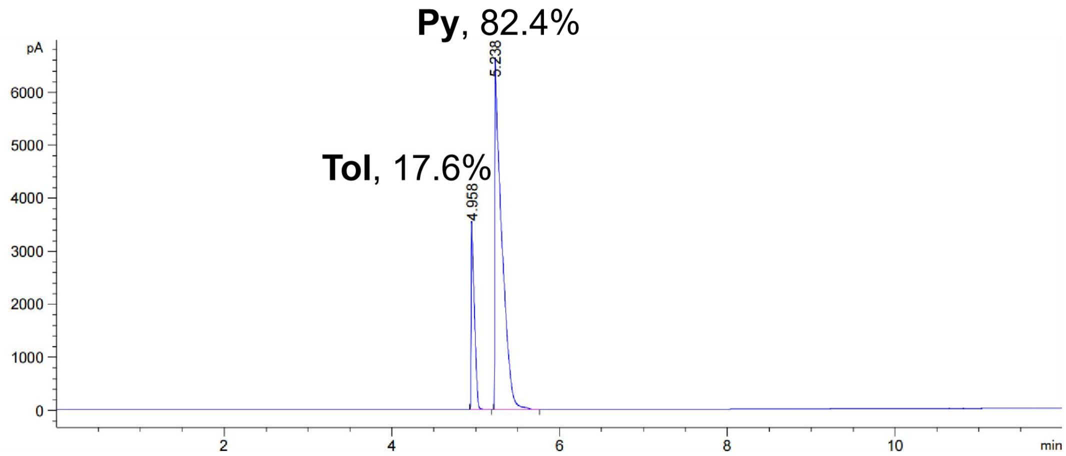

Supplementary Fig. 146 GC measurements of the relative uptake of **Tol**/**Py** in **MeP5-MOF-3** after ~20 mg of crystals were placed in 100 μL of a 90:10 *v*/*v* **Tol**/**Py** mixture and allowed to stand for two minutes.

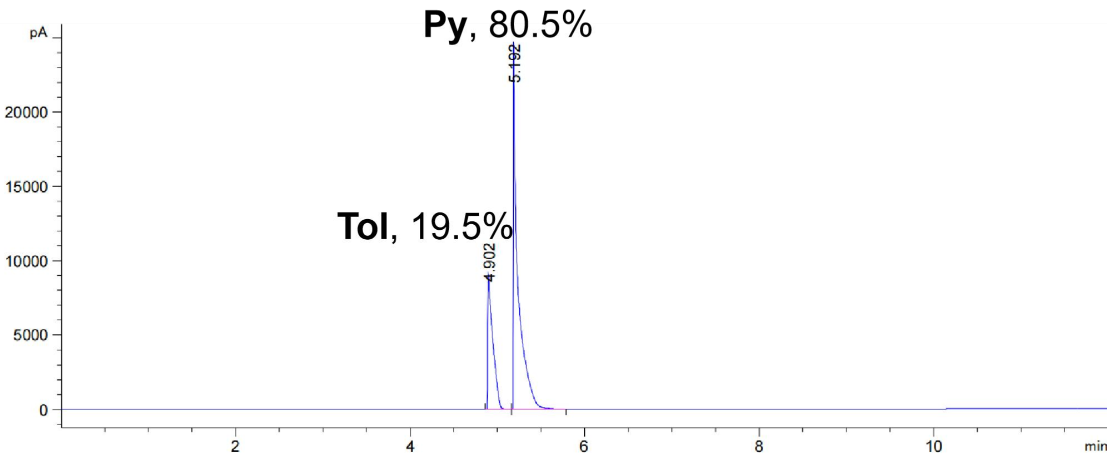

Supplementary Fig. 147 GC measurements of the relative uptake of **Tol**/**Py** in **MeP5-MOF-4** after ~20 mg of crystals were placed in 100 μL of a 90:10 *v*/*v* **Tol**/**Py** mixture and allowed to stand for two minutes.

Supplementary Table 19 Molecular composition of **MeP5-MOF-1**. Here the chemical formula and formula weight were calculated from SCXRD data.

Formula	Chemcial formula	Formula weight (g/mol)	mol%
[Zn_2(**MeP5BPy**)(**TPPE**)]	$C_{107}H_{84}N_2O_{16}Zn_2$	1784.61	100
**MeP5BPy**	$C_{53}H_{52}N_2O_8$	845.01	47.4
**TPPE**	$C_{54}H_{32}O_8$	808.84	45.3
Zn	Zn_2	65.38	7.3

Supplementary Table 20 Molecular composition of **MeP5-MOF-2**. Here the chemical formula and formula weight were calculated from SCXRD data.

Formula	Chemcial formula	Formula weight (g/mol)	mol%
[Zn_2(**MeP5BPPy**)(**TPPE**)]	$C_{119}H_{92}N_2O_{16}Zn_2$	1936.80	100
**MeP5BPPy**	$C_{65}H_{60}N_2O_8$	997.20	51.5
**TPPE**	$C_{54}H_{32}O_8$	808.84	41.8
Zn	Zn_2	65.38	6.7

Supplementary Table 21 Molecular composition of **Model-MOF-1**. Here the chemical formula and formula weight were calculated from SCXRD data.

Formula	Chemcial formula	Formula weight (g/mol)	mol%
[Zn_2(**PBPy**)(**TPPE**)]	$C_{70}H_{44}N_2O_8Zn_2$	1171.89	100
**PBPy**	$C_{16}H_{12}N_2$	232.29	19.8
**TPPE**	$C_{54}H_{32}O_8$	808.84	69.0
Zn	Zn_2	65.38	11.2

Supplementary Table 22 The amount of uptake for **Tol** and **Py**, and the selectivity of **Py** in 100 µL of the 90:10 *v*:*v* **Tol**/**Py** mixture using ~20 mg of adsorbents. Here the amount of uptake of **Tol**/**Py** to struts was calculated from the NMR spectra.

Substance	Amount of uptake of **Tol**/**Py** (mol of **Tol** or **Py**/*per* mol of struts)	Selectivity of **Py** (mol%) based on the NMR spectra	Selectivity of **Py** (mol%) based on the GC spectra
**MeP5-MOF-1**	0.7/6.9	90.8	90.3
**MeP5-MOF-2**	0.3/2.7	90.0	89.5
**Model-MOF-1**	0.9/0.6	40.0	40.6
**MeP5**	0.7/0.8	53.3	55.1

Here, the density (*ρ*) of **Tol** and **Py** are 0.872 and 0.983 g/mL, respecrtively. The relative molecular masses (*M*) of **Tol** and **Py** are 92.14 and 79.10 g/mol, respecrtively. The initial mole numbers (*n*) of **Tol** and **Py** in 100 µL of the 90:10 *v*:*v* **Tol**/**Py** mixture were calculated to be 0.852 and 0.124 mmol, respectively. The initial mole percentages (*x*) of **Tol** and **Py** are 87.3% and 12.7%, respectively. Taking **MeP5-MOF-1** as an example, the mole percentages of **Tol** in the mixture increased from 87.3% to 96.9% after the adsorbtion study, and the selectivity (*W*) of **Py** was 90.3%. The adsorbed moles of **Py** could be calculated according to the following equation (2):

Mole percentage of **Py** = (initial mole of **Py** – adsorbed mole of **Py**) ÷ (initial mole of **Tol** and **Py** – adsorbed mole of **Tol** and **Py**)

Adsorbed mole of **Py** = 0.097 mmol.

Supplementary Table 23 Moles of adsorbed **Py** in 100 µL of the 90:10 *v*:*v* **Tol**/**Py** mixture using ~20 mg adsorbents. Here the selectivity and mole percentage of **Py** after adsorbed were calculated from GC results.

Substance	Selectivity of **Py** (mol%)	Mole percentage of **Py** after adsorbed (mol%)	Adsorbed mol of **Py** (mmol)
**MeP5-MOF-1**	90.3	3.1	~0.097
**MeP5-MOF-2**	89.5	9.5	~0.035
**Model-MOF-1**	40.6	11.5	~0.016
**MeP5**	55.1	10.9	~0.022

Supplementary Table 24 Moles of adsorbed of **Py** in 100 μL of the 90:10 *v*:*v* **Tol**/**Py** mixture using ~20 mg of adsorbents. Here the calculated adsorbed mole of **Py** was obatined from the NMR spectra and the actual adsorbed mole of **Py** was calculated from GC results.

Substance	Amount of adsorbent (mmol)	Amount of uptake of **Py** (mol of **Py**/*per* mol of struts)	calculated adsorbed **Py** molecules (mmol)	actual adsorbed **Py** molecules (mmol)
**MeP5-MOF-1**	~0.011	6.9	~0.077	~0.097
**MeP5-MOF-2**	~0.010	2.7	~0.028	~0.035
**Model-MOF-1**	~0.017	0.6	~0.010	~0.016
**MeP5**	~0.027	0.8	~0.022	~0.022

The efficiency of **MeP5-MOF-1** and **MeP5-MOF-2** under bulk study conditions (~200 mg) was investigated further. It was found that **MeP5-MOF-1** and **MeP5-MOF-2** produced similar results as compared to using ~20 mg of each MOF adsorbent. In these experiments, **MeP5-MOF-1** and **MeP5-MOF-2** were washed with acetone (10 mL) five times and used in next run. These cycles were conducted three times, and the efficiency was investigated by GC and ^{1}H NMR spectral methods.

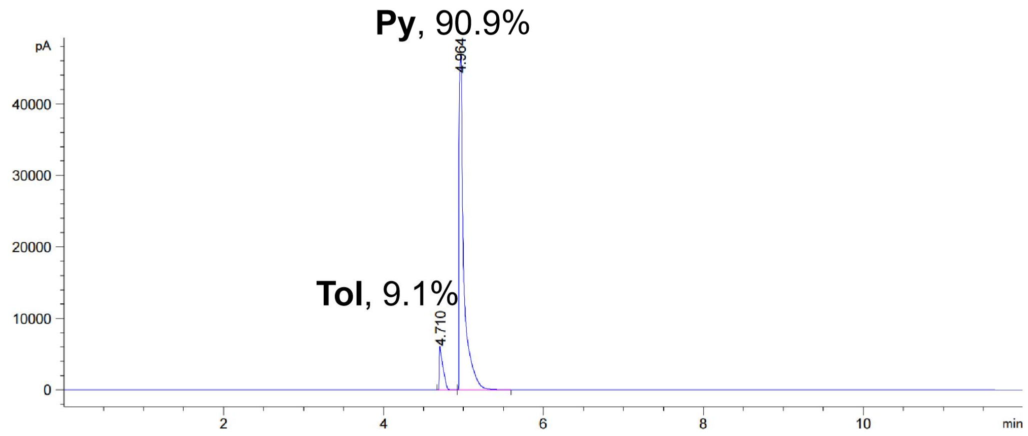

Supplementary Fig. 148 GC measurements of the relative uptake of **Tol**/**Py** in **MeP5-MOF-1** after ~200 mg of crystals were placed in 1 mL of a 90:10 *v*/*v* **Tol**/**Py** mixture and allowed to stand for two minutes.

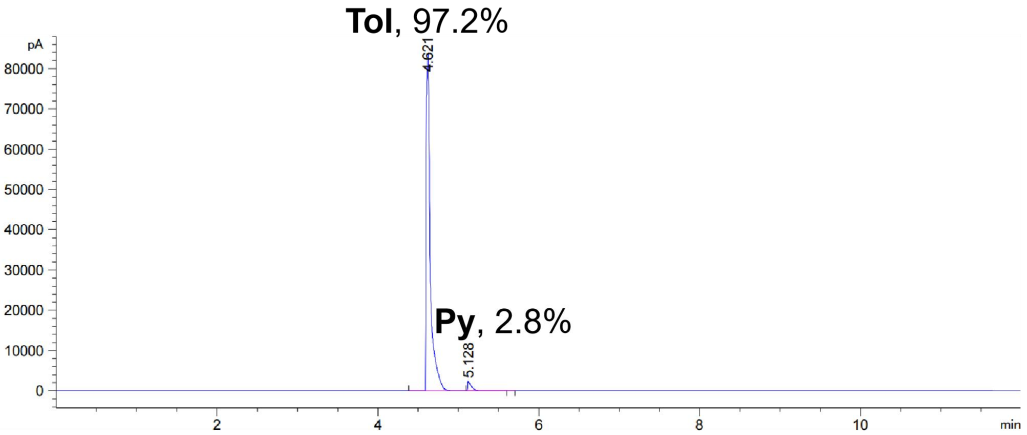

Supplementary Fig. 149 GC measurements of 1 mL of the residual 90:10 *v*:*v* (87.3:12.7 in mole percentage) **Tol**/**Py** mixture after a purification study involving adding ~200 mg crystals of **MeP5-MOF-1**. The mole percentage of **Tol** increased from 87.3% to 97.2% during the course of this experiment.

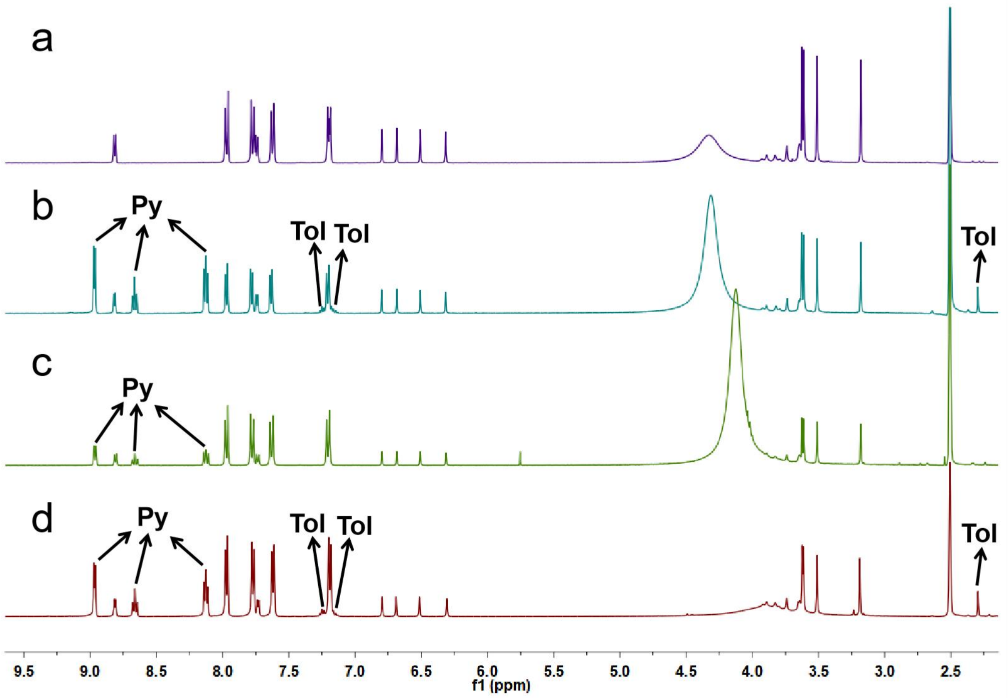

Supplementary Fig. 150 ^{1}H NMR spectra (500 MHz, DMSO-d_6:DCl = 100:1, 298 K): (a) initial **MeP5-MOF-1**; (b) initial **MeP5-MOF-1** after immersing in a 90:10 *v/v* **Tol**/**Py** mixture; (c) recycled **MeP5-MOF-1** washed with acetone (10 mL) five times. Under these conditions, about 70% guest molecules were removed; (d) recycled **MeP5-MOF-1** after immersing in a 90:10 *v/v* **Tol**/**Py** mixture.

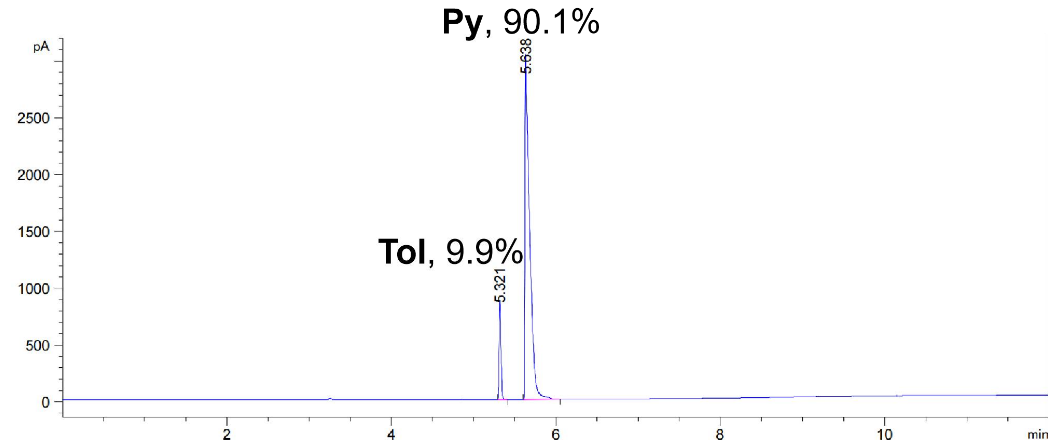

Supplementary Fig. 151 GC measurements of the relative uptake of **Tol**/**Py** in **MeP5-MOF-2** after ~200 mg of crystals were placed in 1 mL of a 90:10 *v*/*v* **Tol**/**Py** mixture and allowed to stand for two minutes.

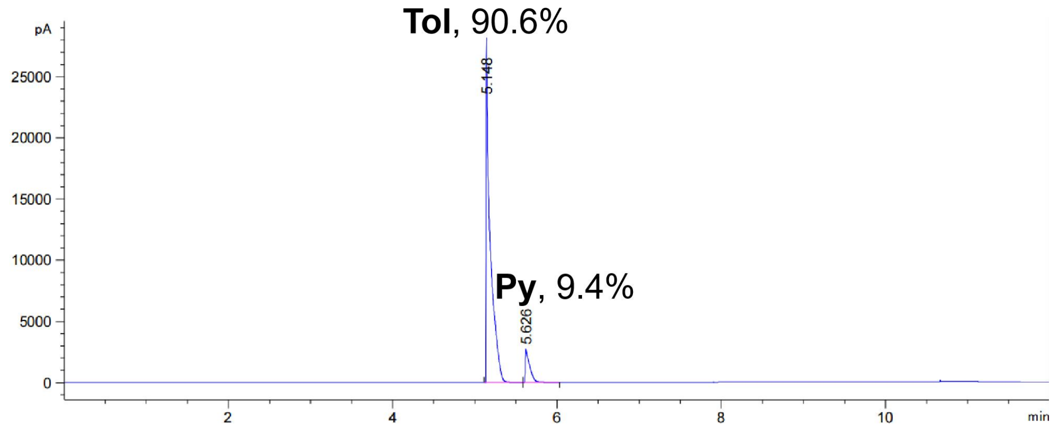

Supplementary Fig. 152 GC measurements of 1 mL of the residual 90:10 *v*:*v* (87.3:12.7 in mole percentage) **Tol**/**Py** mixture after purification studies involving adding ~200 mg crystals of **MeP5-MOF-2**. The mole percentage of **Tol** increased from 87.3% to 90.6% during the course of this experiment.

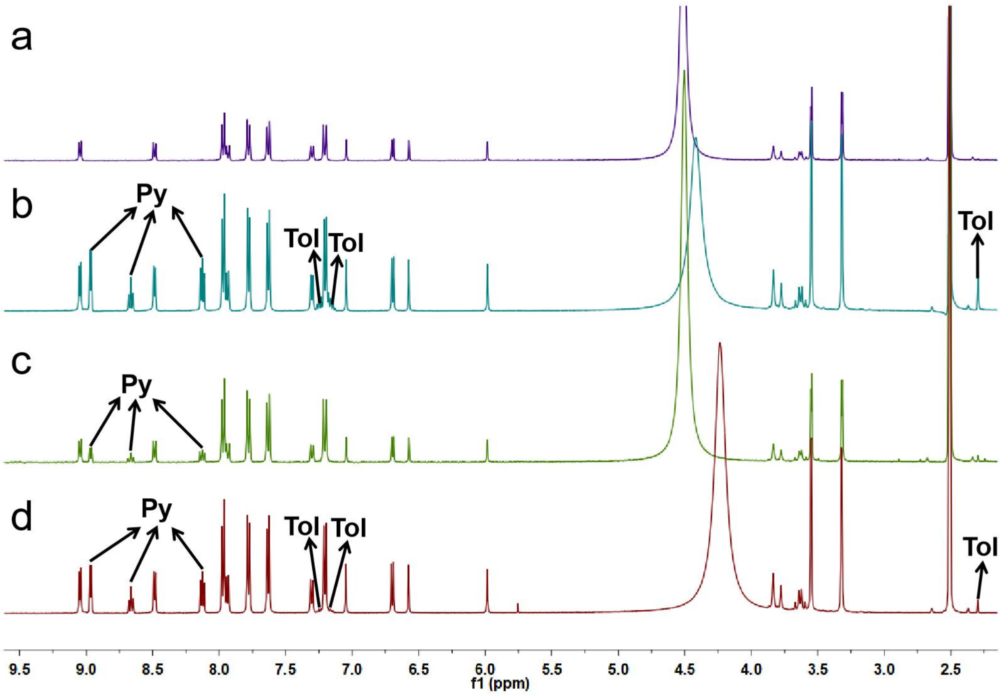

Supplementary Fig. 153 ^{1}H NMR spectra (500 MHz, DMSO-d_6:DCl = 100:1, 298 K): (a) initial **MeP5-MOF-2**; (b) initial **MeP5-MOF-2** after immersing in a 90:10 *v/v* **Tol**/**Py** mixture; (c) recycled **MeP5-MOF-2** washed with acetone (10 mL) five times; here, about 80% guest molecules were removed; (d) recycled **MeP5-MOF-2** after immersing in a 90:10 *v/v* **Tol**/**Py** mixture.

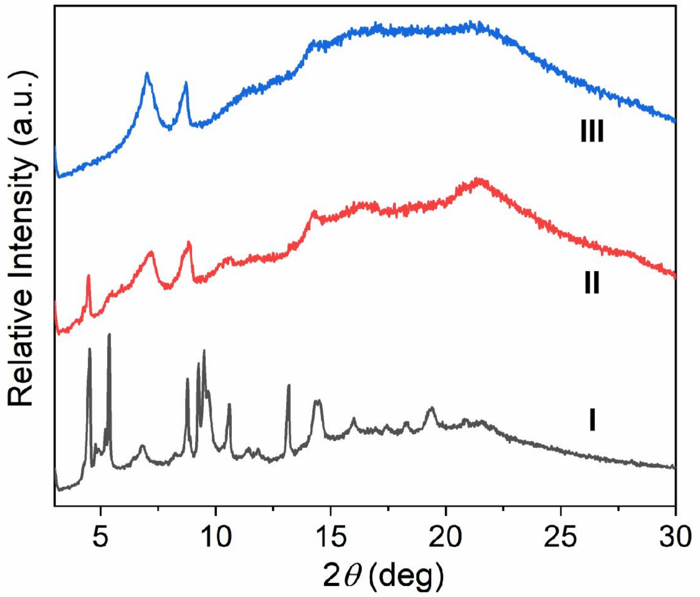

Supplementary Fig. 154 PXRD patterns of **MeP5-MOF-1**: **I**, from a single crystal sample; **II**, after the sample was immersed in a 90:10 *v/v* **Tol**/**Py** mixture and washed with acetone (10 mL) five times; **III**, after the treatments in **II** were performed three cycles.

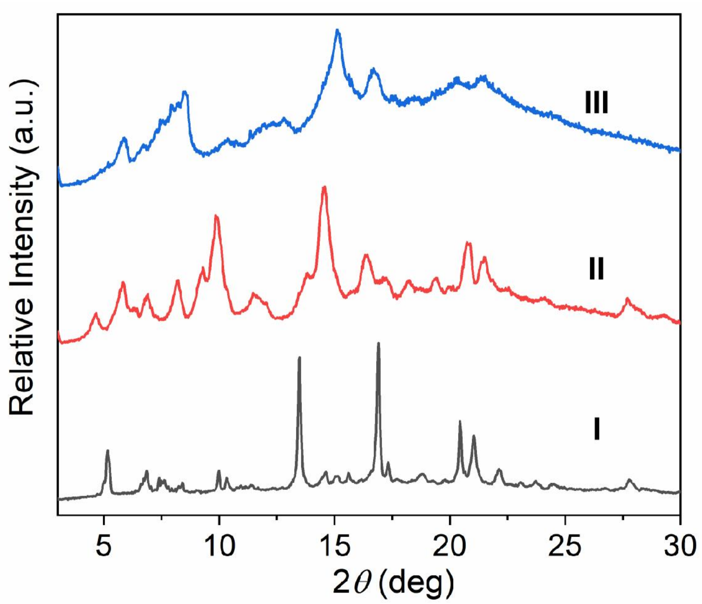

Supplementary Fig. 155 PXRD patterns of **MeP5-MOF-2**: **I**, from a single crystal sample; **II**, after the sample was immersed in a 90:10 *v/v* **Tol**/**Py** mixture and washed with acetone (10 mL) five times; **III**, after the treatments in **II** were performed three cycles.

Activation of MeP5-MOF-1 and MeP5-MOF-2 for gas isotherm

After the vials of **MeP5-MOF-1** were removed from the oven, the mother liquor was decanted and ethanol was added to the crystals. The ethanol was then decanted and replaced daily for three days. The crystals were transferred into a SCD-350M (ShiAnjia Biotechnology Company) supercritical CO_2 dryer. Excess ethanol was decanted, the temperature was lowered to 5 °C, and the chamber was filled with liquid CO_2. The sample was soaked for 12 hours total, venting for 5 min every 2 hours. The chamber was then heated to 35 °C, and the supercritical CO_2 was bled off at a rate of 10 ml/min until the chamber was at ambient pressure (ca. 12 h). The chamber was opened and the sample was quickly sealed and taken into a nitrogen atmosphere glove box for further manipulations. The dried crystals were transferred into a pre-weighed glass sample tube. The tube was sealed and quickly transferred to a dynamic vacuum setup. The sample was kept under vacuum at room temperature for 12 hours and then used for the gas adsorption measurements. Sorption of CO_2 studies were performed because these kind of MOFs typically display strong affinity for CO_2 relative to N_2[S15].

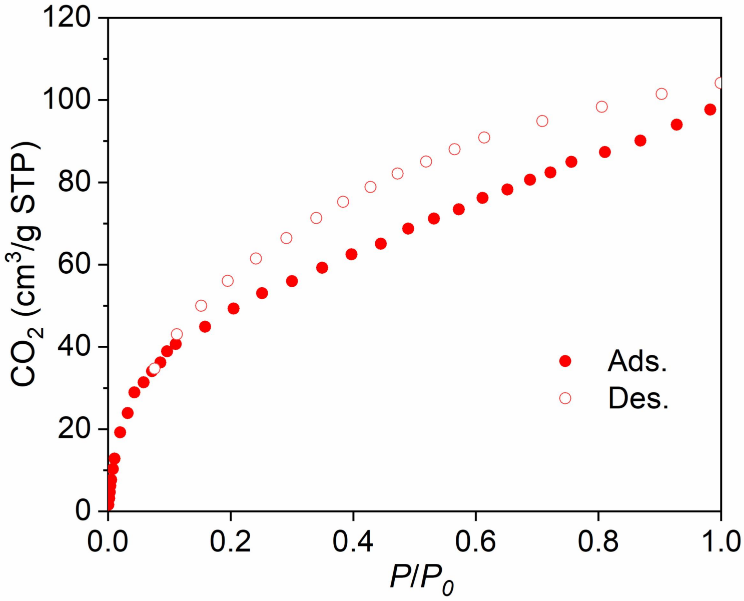

Supplementary Fig. 156 Experimental CO_2 adsorption isotherms at 195 K measuring the porosity of activated **MeP5-MOF-1**. The apparent Brunauer-Emmett-Teller (BET) surface area is calculated to be 160 m^2/g.

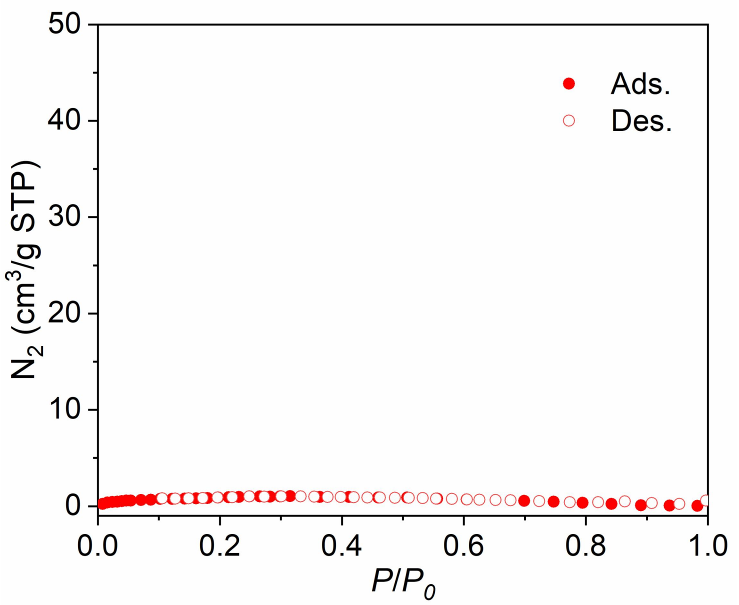

Supplementary Fig. 157 Experimental N_2 adsorption/desorption isotherms at 77 K measuring the porosity of activated **MeP5-MOF-1**. The apparent BET surface area is calculated to be 5 m^2/g, which is probably induced from the partial collapse of frameworks.

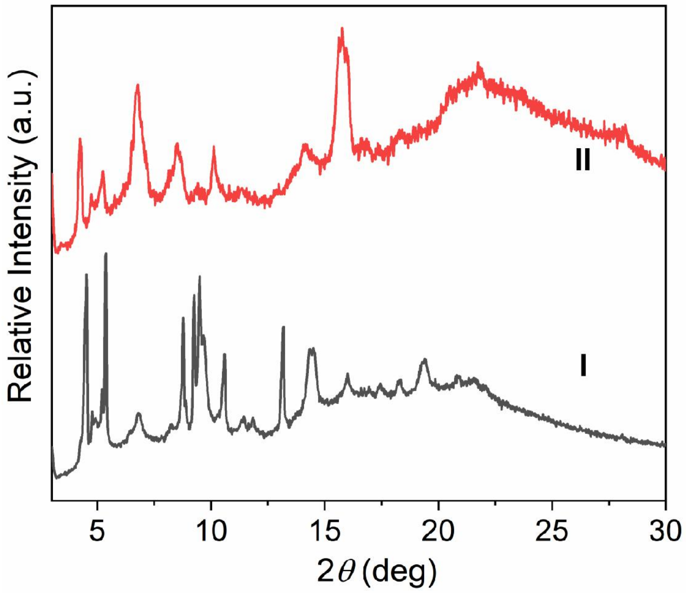

Supplementary Fig. 158 PXRD patterns of **MeP5-MOF-1**: **I**, from a single crystal sample; **II**, after the sample was activated.

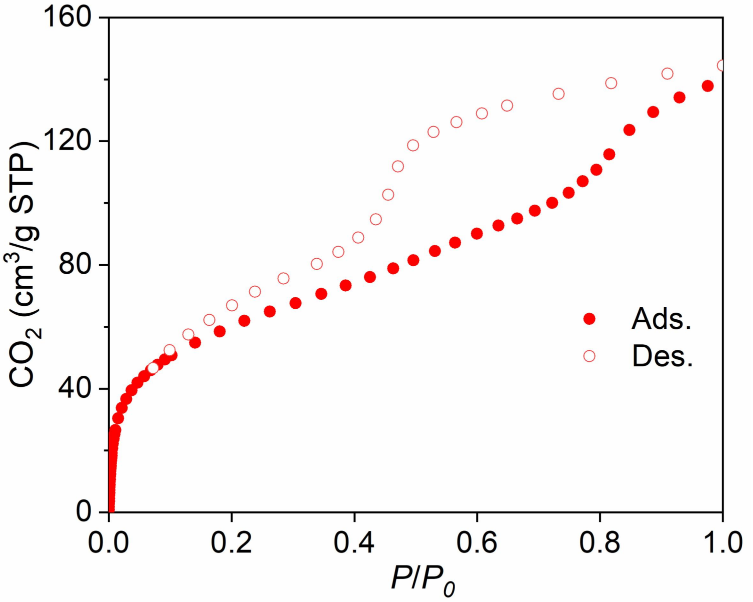

Supplementary Fig. 159 Experimental CO_2 adsorption isotherms at 195 K measuring the porosity of activated **MeP5-MOF-2**. The apparent BET surface area is calculated to be 190 m^2/g.

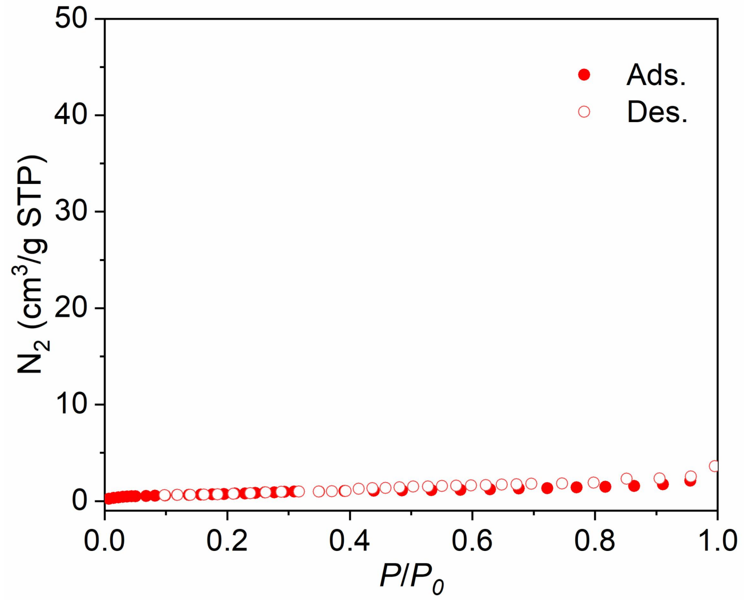

Additional Fig. 160 Experimental N_2 adsorption isotherms at 77 K measuring the porosity of activated **MeP5-MOF-2**. The apparent BET surface area is calculated to be 3 m^2/g, which is probably induced from the partial collapse of frameworks.

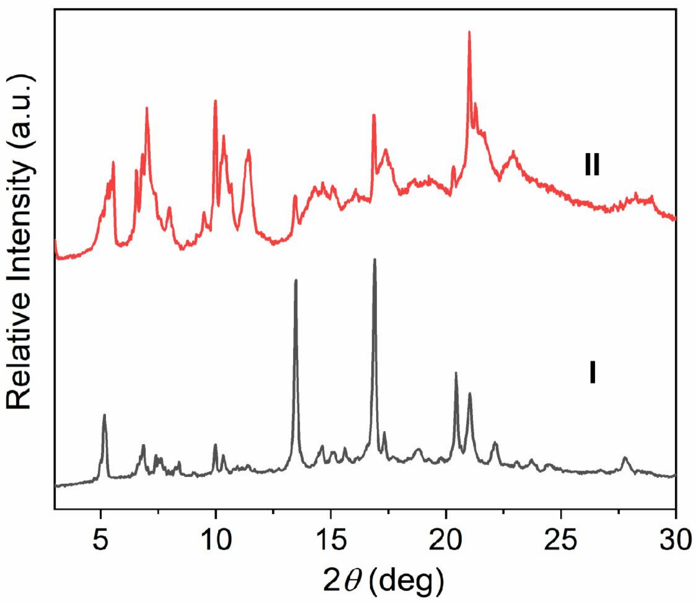

Supplementary Fig. 161 PXRD patterns of **MeP5-MOF-2**: **I**, from a single crystal sample; **II**, after the sample was activated.

TGA analyses of **MeP5-MOF-1** and **MeP5-MOF-2** were carried. The resultant TGA curves revealed that **MeP5-MOF-1** had only 3.6% weight loss before the temperature reached around 150 °C. Decomposition became noticeable at around 350 °C while **MeP5-MOF-2** had a 3.9% weight loss before the temperature reached around 150 °C and began to decompose at around 400 °C.

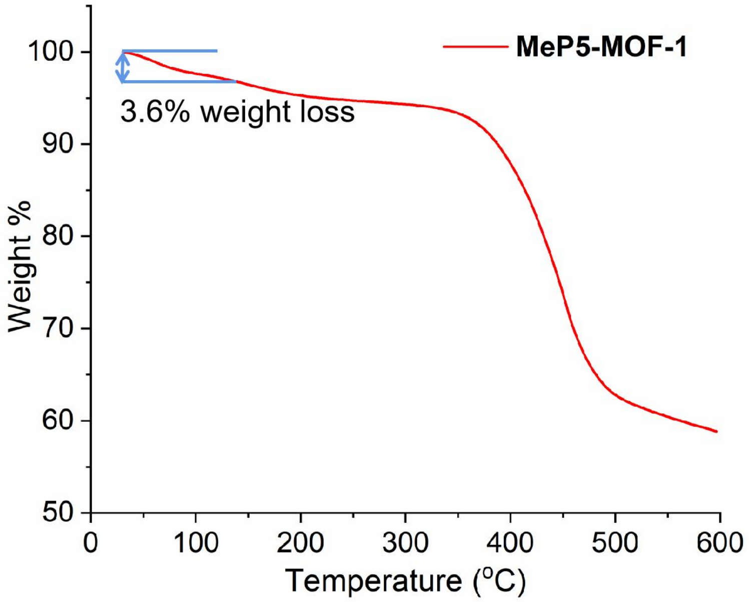

Supplementary Fig. 162 TGA curve of the decomposition of **MeP5-MOF-1**.

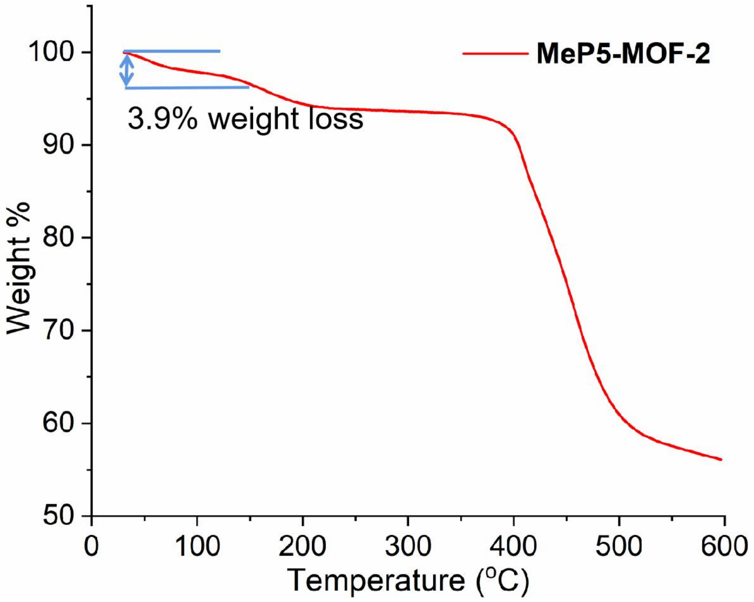

Supplementary Fig. 163 TGA curve of the decomposition of **MeP5-MOF-2**.

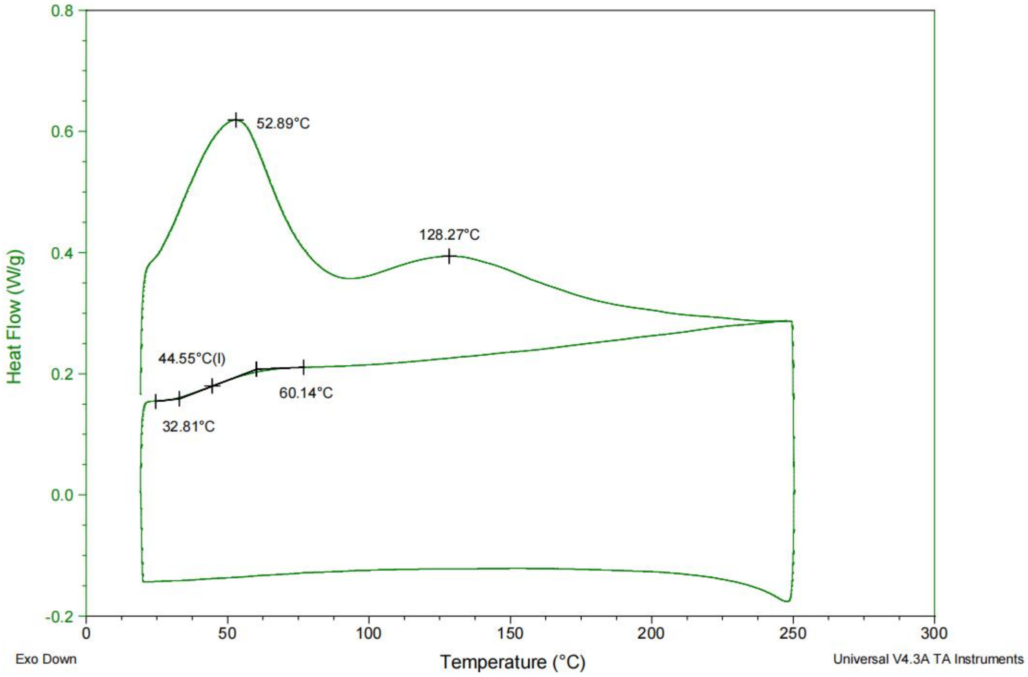

Supplementary Fig. 164 DSC analysis of **MeP5-MOF-1**. The broad peaks around 53 ℃ and 128 ℃ correspond to solvent loss.

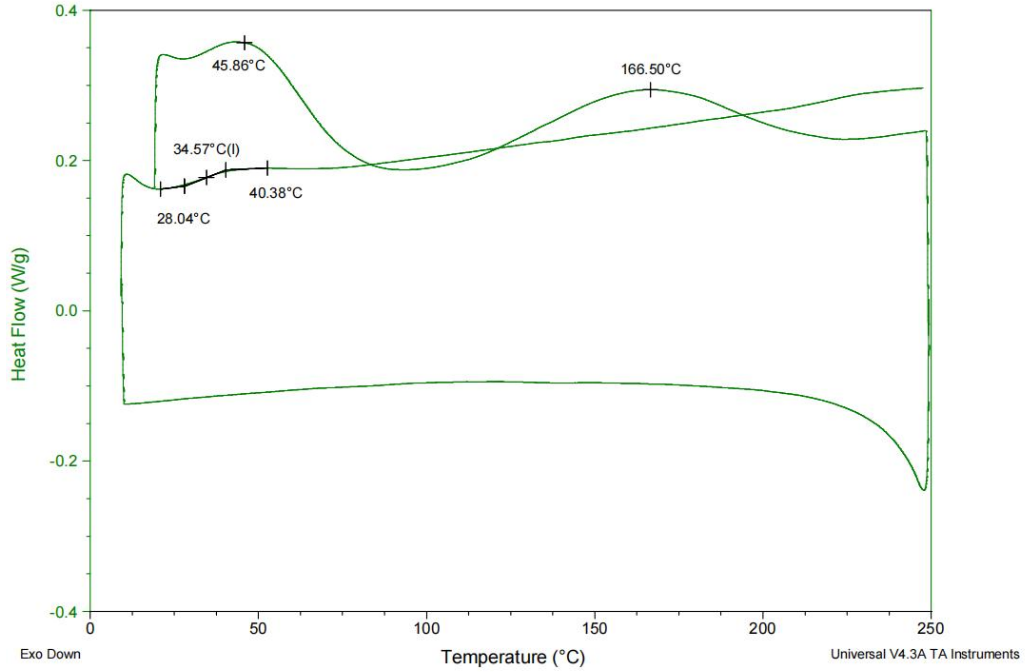

Supplementary Fig. 165 DSC analysis of **MeP5-MOF-2**. The broad peaks around 46 ℃ and 167 ℃ correspond to solvent loss.

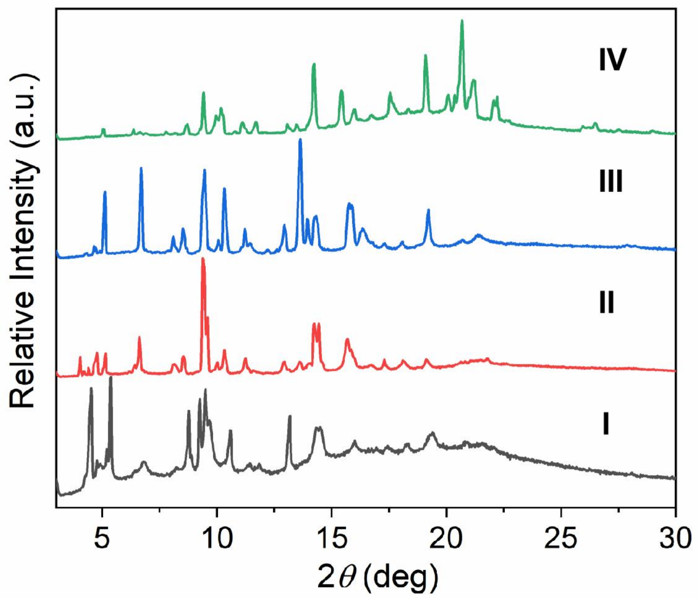

Supplementary Fig. 166 Experimental PXRD patterns of **MeP5-MOF-1**: **I**, from a single crystal sample; **II**, after immersing in acetone; **III**, after immersing in ethanol; **IV**, after immersing in tetrahydrofuran. The various PXRD patterns after uptake of guests are considered reflective of the dynamics within the MOFs.

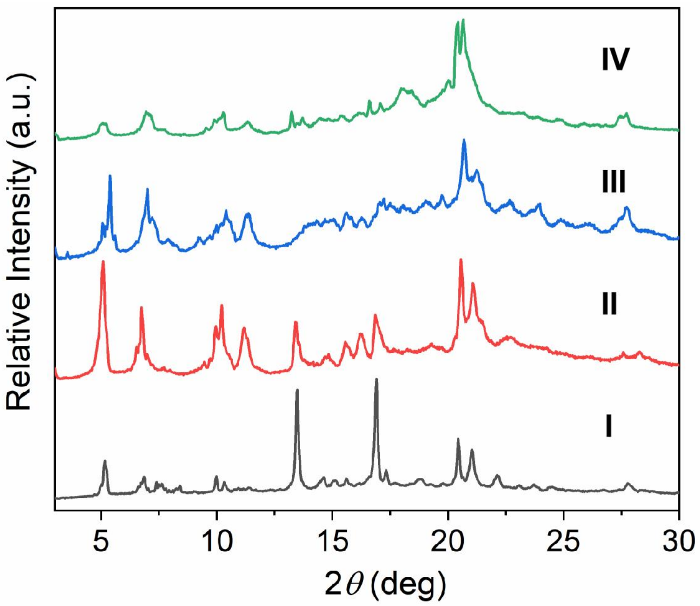

Supplementary Fig. 167 Experimental PXRD patterns of **MeP5-MOF-2**: **I**, from a single crystal sample; **II**, after immersing in acetone; **III**, after immersing in ethanol; **IV**, after immersing in tetrahydrofuran. The various PXRD patterns after uptake of guests are considered reflective of the dynamics within the MOFs.

The changes of PXRD patterns could be ascribed to the dynamics of the frameworks and pillar[5]arene units and partial structural collapse after desolvated. It can be also

assured that the (Zn···Zn)−N bonds are almost 180° in solvent while the (Zn···Zn)−N bonds become considerably bent in the desolvated state. It appears that this angular distortion occurs in order to minimize the empty space in the desolvated structure, which can lead to the observed structural transformation[S16].

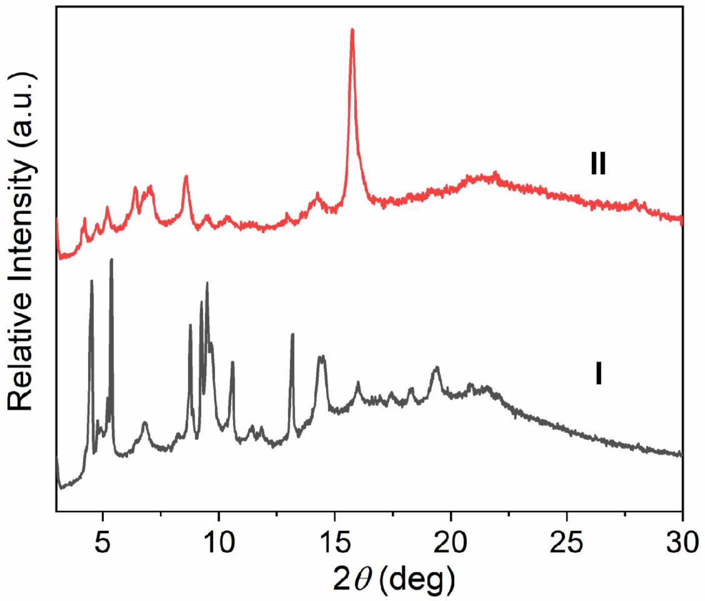

Supplementary Fig. 168 PXRD patterns of **MeP5-MOF-1**: **I**, from a single crystal sample; **II**, after the sample was desolvated under vacuum at 40 °C for 1 h.

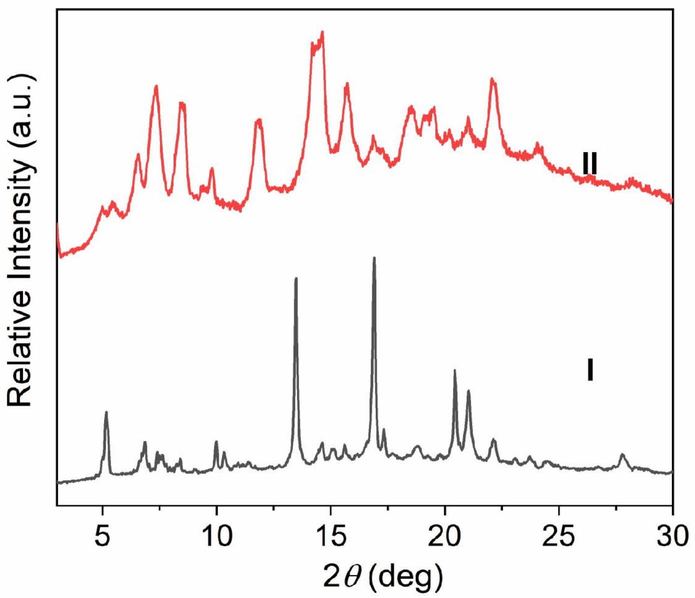

Supplementary Fig. 169 PXRD patterns of **MeP5-MOF-2**: **I**, from a single crystal sample; **II**, after the sample was desolvated under vacuum at 40 °C for 1 h.

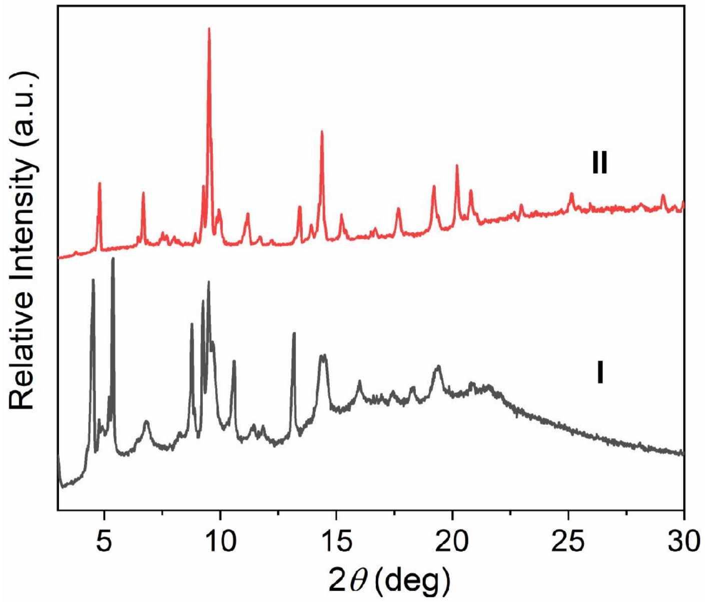

Supplementary Fig. 170 PXRD patterns of **MeP5-MOF-1**: **I**, from a single crystal sample; **II**, after the sample was immersed in deionized water at room temperature.

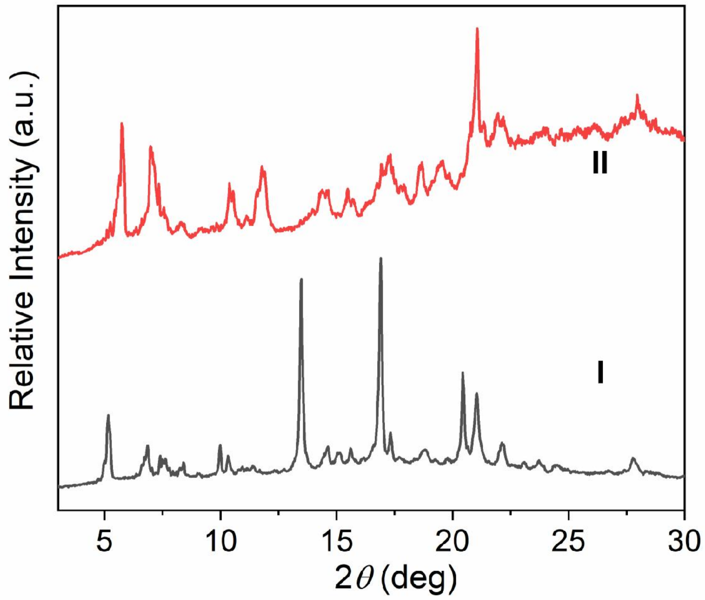

Supplementary Fig. 171 PXRD patterns of **MeP5-MOF-2**: **I**, from a single crystal sample; **II**, after the sample was immersed in deionized water at room temperature.

The structure of **MeP5-MOF-1** is non-interpenetrated and possesses open frameworks while **MeP5-MOF-2** is two-fold interpenetrated. The pore volume values of **MeP5-MOF-1** and **MeP5-MOF-2** were calculated by the equation (3):

Pore volume = Cell free volume ÷ (Cell volume × Density)

Supplementary Table 25 The pore volume of **MeP5-MOF-1** and **MeP5-MOF-2**.

Substance	Cell free volume (Å^3)	Density (g/cm^3)	Cell volume (Å^3)	Pore volume (cm^3/g)
**MeP5-MOF-1**	4895.41	0.32	6084.43	2.52
**MeP5-MOF-2**	4884.7	0.75	8589.14	0.76

Supplementary Table 26 Comparison with the previous similar published results for selective separation of **Tol** and **Py**[S17–S19].

Adsorbent	Time to point of analysis	Amount of uptake (mol of **Py**/*per* mol of host)	Ref.
**MeP5-MOF-1**	~2 min	~6.7	This work
**MeP5-MOF-2**	~2 min	~2.6	This work
Cucurbit[6]uril	~25 h	~1.0	17
Calix[4]pyrrole	~4 h	~6.0	18
Cucurbit[10]uril	~6 h	~2.0	19

12. Supplementary references

[S1] Li, Q., Zhang, W., Miljanic, O. S., Sue, C.-H., Zhao, Y.-L., Liu, L., Knobler, C. B., Stoddart, J. F., Yaghi, O. M. Docking in metal−organic frameworks. *Science* **325**, 855−859 (2009).

[S2] Lou, X.-Y., Yang, Y.-W. Pyridine- conjugated pillar[5] arene: From molecular crystals of blue luminescence to red-emissive coordination nanocrystals. *J. Am. Chem. Soc.* **143**, 11976−11981 (2021).

[S3] Zhu, H., Li, Q., Shi, B., Xing, H., Sun, Y., Lu, S., Shangguan, L., Li, P., Huang, F., Stang, P. J. Formation of planar chiral platinum triangles *via* pillar[5]arene for circularly polarized luminescence. *J. Am. Chem. Soc.* **142**, 17340−17345 (2020)

[S4] Ogoshi, T., Kanai, S., Fujinami, S., Yamagishi, T.-a., Nakamoto, Y. *para*-Bridged symmetrical pillar[5]arenes: their lewis acid catalyzed synthesis and host−guest property. *J. Am. Chem. Soc.* **130***,* 5022−5023 (2008).

[S5] Hu, X.-B., Chen, Z., Chen, L., Zhang, L., Hou, J.-L., Li, Z.-T. Pillar[*n*]arenes (*n* = 8 – 10) with two cavities: synthesis, structures, and complexing properties. *Chem. Commun.* **48**, 10999−11001 (2012).

[S6] Sheldrick, G. A short history of SHELX. *Acta Crystallogr. Sect. A: Found. Crystallogr.* **A64**, 112−122 (2008).

[S7] Sheldrick, G. Crystal structure refinement with SHELXL. *Acta Crystallogr., Sect. C: Struct. Chem*. **C71**, 3−8 (2015).

[S8] Dolomanov, O. V., Bourhis, L. J., Gildea, R. J., Howard, J. A. K., Puschmann, H. OLEX2: A complete structure solution, refinement and analysis program. *J. Appl. Cryst*. **42**, 339−341 (2009).

[S9] Materials Studio (BIOVIA 2017) , Accelrys Inc., San Diego, CA, 2017.

[S10] Jiang, Z., Duan, W., Lin, W., Yang, L., Wu, Z., Wang, J., Wang, S., Du, P., Li, Q. Cycloparaphenylene and their radicals anchored to a metal–organic framework. *Mater. Today Chem.* **25**, 100973 (2022).

[S11] Zheng, H., Fu, L., Wang, R., Jiao, J., Song, Y., Shi, C., Chen, Y., Jiang, J., Lin, C., Ma, J., Wang, L. Cation controlled rotation in anionic pillar[5] arenes and its application for fluorescence switch. *Nat. Commun.* **14**, 590 (2023).

[S12] Li, M., Liu, Y., Shao, L., Hua, B., Wang, M., Liang, H., Khashab, N. M., Sessler, J. L., Huang, F. Pillararene- Based variable stoichiometry co- crystallization: A versatile approach to diversified solid- State superstructures. *J. Am. Chem. Soc.* **145**, 667−675 (2023).

[S13] Connors, K. Binding constants, the measurement of molecular complex stability, Wiley, New York, 1987.

[S14] Lide, D. R. CRC handbook of chemistry and physics, Boca Raton, FL, pp. 203−365 (2005).

[S15] Chen, B., Ma, S., Hurtado, E. J., Lobkovsky, E. B., Zhou, H.-C. A triply interpenetrated microporous metal−organic framework for selective sorption of gas molecules. *Inorg. Chem.* **46**, 8490−8492 (2007).

[S16] Aggarwal, H., Bhatt, P. M., Bezuidenhout, C. X., Barbour, L. J. Direct evidence for single- crystal to single- crystal switching of degree of interpenetration in a metal− organic framework. *J. Am. Chem. Soc.* **136**, 3776−3779 (2014).

[S17] Li, Q., Jie, K., Huang, F. Highly selective separation of minimum-boiling azeotrope toluene/pyridine by nonporous adaptive crystals of cucurbit[6]uril. *Angew. Chem. Int. Ed.* **59**, 5355−5358 (2020).

[S18] Luo, D., Tian, J., Sessler, J. L., Chi, X. Nonporous adaptive calix[4] pyrrole crystals for polar compound separations. *J. Am. Chem. Soc*. **143**, 18849−18853 (2021).

[S19] Liu, M., Cen, R., Li, J., Li, Q., Tao, Z., Xiao, X., Isaacs, L. Double-cavity *nor-seco*-cucurbit[10]uril enables efficient and rapid separation of pyridine from

mixtures of toluene, benzene, and pyridine. *Angew. Chem. Int. Ed.* **61**, e202207209 (2022).
