# Supplementary material for: Pillararene incorporated metal–organic frameworks for supramolecular recognition and selective separation
Source: Nat Commun. 2023 Aug 15;14:4927. doi: 10.1038/s41467-023-40594-2 (PMC10427641; doi:10.1038/s41467-023-40594-2)
Supplement: Supplementary file 3 — Description of Additional Supplementary Files [file 41467_2023_40594_MOESM3_ESM.pdf]

Description of Additional Supplementary Files

File Name: Supplementary Data 1

Description: Cif and Checkcif files of all the single crystal structures in the manuscript. Single crystal X-ray diffraction data for **MeP5BPPy** (CIF), CCDC number 2211420; **MeP5-MOF-1** (CIF), CCDC number 2211421; $(DMF)_2$@**Model-MOF-1** (CIF), CCDC number 2217237; $(DMA)_2$@**MeModel-MOF-1** (CIF), CCDC number 2213797; **MeP5-MOF-2** (CIF), CCDC number 2211422; DMF@**MeP5-MOF-2** (CIF), CCDC number 2211423; $(DMF)_3$@**MeP5-MOF-2** (CIF), CCDC number 2217239; **Model-MOF-2** (CIF), CCDC number 2211425; *pS*-**MeP5-MOF-2** (CIF), CCDC number 2217240; *pR*-**MeP5-MOF-2** (CIF), CCDC number 2217241; **MeP5-MOF-3** (CIF), CCDC number 2211424; **MeP5-MOF-4** (CIF), CCDC number 2217410; **(Py)**$_2$@**P5** (CIF), CCDC number 2216441; **Tol@MeP5-MOF-2** (CIF), CCDC number 2217238; **MeP5-MOF-1-105K** (CIF), CCDC number 2267727; **MeP5-MOF-1-G** (CIF), CCDC number 2267730.
