# Supplementary material for: Pillararene incorporated metal–organic frameworks for supramolecular recognition and selective separation
Source: Nat Commun. 2023 Aug 15;14:4927. doi: 10.1038/s41467-023-40594-2 (PMC10427641; doi:10.1038/s41467-023-40594-2)

checkCIF/PLATON report

Structure factors have been supplied for datablock(s) 221014_wqangz_098762_0m

THIS REPORT IS FOR GUIDANCE ONLY. IF USED AS PART OF A REVIEW PROCEDURE FOR PUBLICATION, IT SHOULD NOT REPLACE THE EXPERTISE OF AN EXPERIENCED CRYSTALLOGRAPHIC REFEREE.

No syntax errors found. CIF dictionary Interpreting this report

Datablock: 221014_wqangz_098762_0m

Bond precision:	C-C = 0.0111 A		Wavelength=1.34139
Cell:	a=16.380(3)	b=18.015(3)	c=20.210(4)
	alpha=102.337(8)	beta=90.337(9)	gamma=115.845(8)
Temperature:	262 K		

	Calculated	Reported
Volume	5209.9(17)	5209.9(17)
Space group	P -1	P -1
Hall group	-P 1	-P 1
Moiety formula	C72 H48 N2 O10 Zn2, 2(C4 H9 N O) [+ solvent]	C72 H48 N2 O10 Zn2, 2(C4 H9 N O)
Sum formula	C80 H66 N4 O12 Zn2 [+ solvent]	C80 H66 N4 O12 Zn2
Mr	1406.15	1406.10
Dx,g cm-3	0.896	0.896
Z	2	2
Mu (mm-1)	0.604	0.604
F000	1460.0	1460.0
F000'	1452.08	
h,k,lmax	19,21,24	19,21,24
Nref	19086	18977
Tmin,Tmax	0.924,0.941	0.554,0.751
Tmin'	0.924	

Correction method= # Reported T Limits: Tmin=0.554 Tmax=0.751
AbsCorr = MULTI-SCAN

Data completeness= 0.994 Theta(max)= 53.907

R(reflections)= 0.0999(8256)   wR2(reflections)= 0.2852(18977)

S = 1.031   Npar= 917

The following ALERTS were generated. Each ALERT has the format
test-name_ALERT_alert-type_alert-level.
Click on the hyperlinks for more details of the test.

Alert level C

```
PLAT026_ALERT_3_C Ratio Observed / Unique Reflections (too) Low ..        44% Check
PLAT084_ALERT_3_C High wR2 Value (i.e. > 0.25) ...................       0.29 Report
PLAT242_ALERT_2_C Low    'MainMol' Ueq as Compared to Neighbors of         N1 Check
PLAT242_ALERT_2_C Low    'MainMol' Ueq as Compared to Neighbors of         C3 Check
PLAT242_ALERT_2_C Low    'MainMol' Ueq as Compared to Neighbors of        C47 Check
PLAT244_ALERT_4_C Low    'Solvent' Ueq as Compared to Neighbors of        C74 Check
PLAT244_ALERT_4_C Low    'Solvent' Ueq as Compared to Neighbors of         N3 Check
PLAT244_ALERT_4_C Low    'Solvent' Ueq as Compared to Neighbors of        C78 Check
PLAT250_ALERT_2_C Large U3/U1 Ratio for Average U(i,j) Tensor ....        2.1 Note
PLAT250_ALERT_2_C Large U3/U1 Ratio for Average U(i,j) Tensor ....        2.9 Note
PLAT260_ALERT_2_C Large Average Ueq of Residue Including       O11      0.276 Check
PLAT260_ALERT_2_C Large Average Ueq of Residue Including       O12      0.158 Check
PLAT309_ALERT_2_C Single Bonded Oxygen (C-O > 1.3 Ang) ..........         O11 Check
PLAT341_ALERT_3_C Low Bond Precision on  C-C Bonds ...............    0.01105 Ang.
PLAT905_ALERT_3_C Negative K value in the Analysis of Variance ...    -21.512 Report
PLAT905_ALERT_3_C Negative K value in the Analysis of Variance ...     -0.604 Report
PLAT911_ALERT_3_C Missing FCF Refl Between Thmin & STh/L=    0.600         92 Report
```

Alert level G

```
ABSMU01_ALERT_1_G  Calculation of _exptl_absorpt_correction_mu
                  not performed for this radiation type.
PLAT002_ALERT_2_G Number of Distance or Angle Restraints on AtSite         11 Note
PLAT003_ALERT_2_G Number of Uiso or Uij Restrained non-H Atoms ...         14 Report
PLAT004_ALERT_5_G Polymeric Structure Found with Maximum Dimension          3 Info
PLAT083_ALERT_2_G SHELXL Second Parameter in WGHT  Unusually Large       8.00 Why ?
PLAT172_ALERT_4_G The CIF-Embedded .res File Contains DFIX Records          4 Report
PLAT173_ALERT_4_G The CIF-Embedded .res File Contains DANG Records          7 Report
PLAT174_ALERT_4_G The CIF-Embedded .res File Contains FLAT Records          1 Report
PLAT178_ALERT_4_G The CIF-Embedded .res File Contains SIMU Records          2 Report
PLAT301_ALERT_3_G Main Residue  Disorder .............(Resd  1  )         5% Note
PLAT413_ALERT_2_G Short Inter XH3 .. XHn     H1       ..H13A     .       2.07 Ang.
                                              -x,-y,1-z  =      2_556 Check
PLAT606_ALERT_4_G Solvent Accessible VOID(S) in Structure ........          ! Info
PLAT794_ALERT_5_G Tentative Bond Valency for Zn1       (II)      .       2.10 Info
PLAT860_ALERT_3_G Number of Least-Squares Restraints ............         134 Note
PLAT912_ALERT_4_G Missing # of FCF Reflections Above STh/L=  0.600         19 Note
PLAT933_ALERT_2_G Number of HKL-OMIT Records in Embedded .res File         21 Note
PLAT978_ALERT_2_G Number C-C Bonds with Positive Residual Density.          0 Info
```

---

0 **ALERT level A** = Most likely a serious problem - resolve or explain
0 **ALERT level B** = A potentially serious problem, consider carefully
17 **ALERT level C** = Check. Ensure it is not caused by an omission or oversight
17 **ALERT level G** = General information/check it is not something unexpected

```
 1 ALERT type 1 CIF construction/syntax error, inconsistent or missing data
14 ALERT type 2 Indicator that the structure model may be wrong or deficient
 8 ALERT type 3 Indicator that the structure quality may be low
 9 ALERT type 4 Improvement, methodology, query or suggestion
 2 ALERT type 5 Informative message, check
```

It is advisable to attempt to resolve as many as possible of the alerts in all categories. Often the minor alerts point to easily fixed oversights, errors and omissions in your CIF or refinement strategy, so attention to these fine details can be worthwhile. In order to resolve some of the more serious problems it may be necessary to carry out additional measurements or structure refinements. However, the purpose of your study may justify the reported deviations and the more serious of these should normally be commented upon in the discussion or experimental section of a paper or in the "special_details" fields of the CIF. checkCIF was carefully designed to identify outliers and unusual parameters, but every test has its limitations and alerts that are not important in a particular case may appear. Conversely, the absence of alerts does not guarantee there are no aspects of the results needing attention. It is up to the individual to critically assess their own results and, if necessary, seek expert advice.

Publication of your CIF in IUCr journals

A basic structural check has been run on your CIF. These basic checks will be run on all CIFs submitted for publication in IUCr journals (*Acta Crystallographica*, *Journal of Applied Crystallography*, *Journal of Synchrotron Radiation*); however, if you intend to submit to *Acta Crystallographica Section C* or *E* or *IUCrData*, you should make sure that full publication checks are run on the final version of your CIF prior to submission.

Publication of your CIF in other journals

Please refer to the *Notes for Authors* of the relevant journal for any special instructions relating to CIF submission.

PLATON version of 12/09/2022; check.def file version of 09/08/2022

Datablock 221014_wqangz_098762_0m - ellipsoid plot

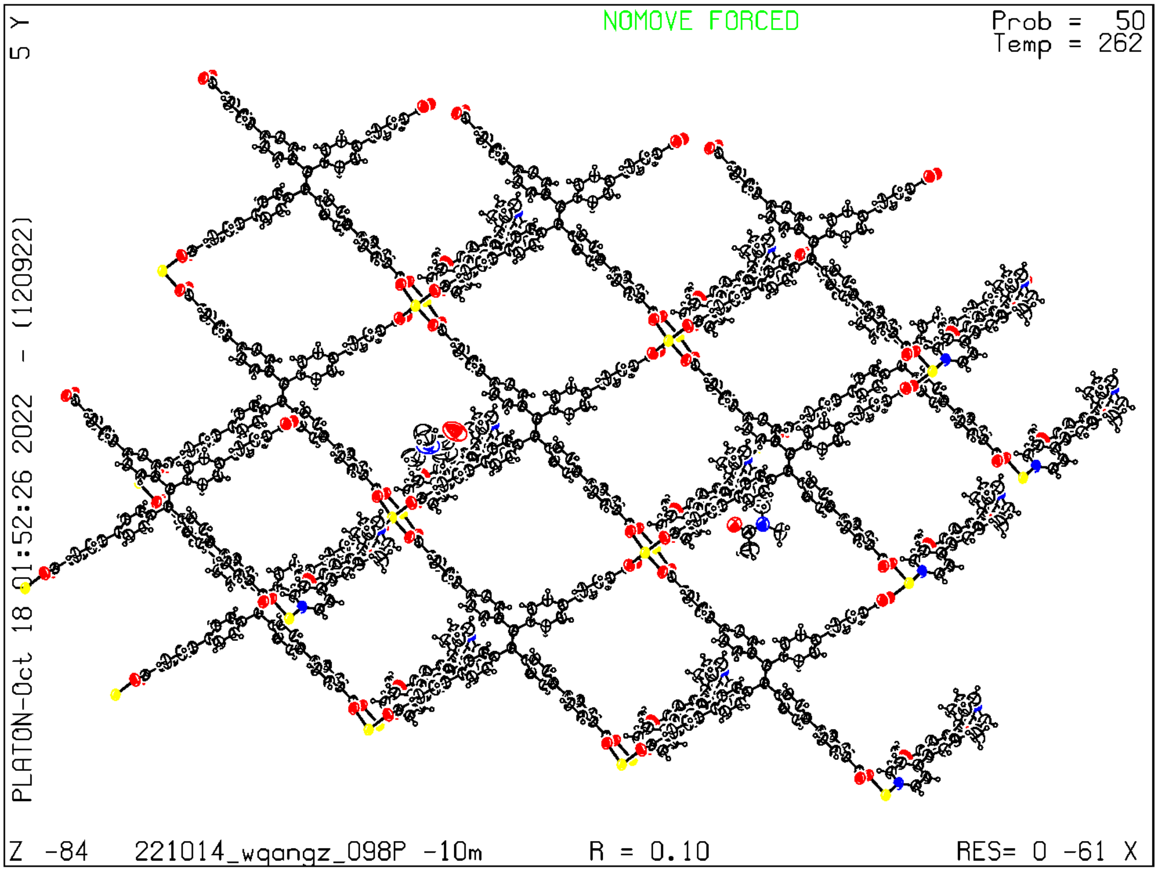

Supplement: Supplementary file 4 — Supplementary Data 1 [file 41467_2023_40594_MOESM4_ESM.zip › Supplementary Data 1/(DMA)2@MeModel-MOF-1.pdf]
