# Supplementary material for: Pillararene incorporated metal–organic frameworks for supramolecular recognition and selective separation
Source: Nat Commun. 2023 Aug 15;14:4927. doi: 10.1038/s41467-023-40594-2 (PMC10427641; doi:10.1038/s41467-023-40594-2)

checkCIF/PLATON report

Structure factors have been supplied for datablock(s) 221031_wqangz_289057_0m_5_sq

THIS REPORT IS FOR GUIDANCE ONLY. IF USED AS PART OF A REVIEW PROCEDURE FOR PUBLICATION, IT SHOULD NOT REPLACE THE EXPERTISE OF AN EXPERIENCED CRYSTALLOGRAPHIC REFEREE.

No syntax errors found. CIF dictionary Interpreting this report

Datablock: 221031_wqangz_289057_0m_5_sq

Bond precision: C-C = 0.0183 A Wavelength=1.34139

Cell:	a=16.4239(13)	b=18.2159(13)	c=20.2865(15)
	alpha=78.748(4)	beta=89.927(4)	gamma=77.576(4)
Temperature:	193 K		

	Calculated	Reported
Volume	5807.9(8)	5807.9(8)
Space group	P -1	P -1
Hall group	-P 1	-P 1
Moiety formula	C70 H44 N2 O8 Zn2, 2(C3 H7 N O) [+ solvent]	C70 H44 N2 O8 Zn2, 2(C3 H7 N O)
Sum formula	C76 H58 N4 O10 Zn2 [+ solvent]	C76 H58 N4 O10 Zn2
Mr	1318.05	1318.00
Dx,g cm-3	0.754	0.754
Z	2	2
Mu (mm-1)	0.521	0.521
F000	1364.0	1364.0
F000'	1355.83	
h,k,lmax	19,21,24	19,21,24
Nref	21271	20969
Tmin,Tmax	0.935,0.949	0.538,0.752
Tmin'	0.935	

Correction method= # Reported T Limits: Tmin=0.538 Tmax=0.752
AbsCorr = MULTI-SCAN

Data completeness= 0.986 Theta(max)= 53.906

R(reflections)= 0.1388(14067)　　wR2(reflections)= 0.3489(20969)

S = 1.130　　Npar= 834

The following ALERTS were generated. Each ALERT has the format
test-name_ALERT_alert-type_alert-level.
Click on the hyperlinks for more details of the test.

Alert level B

PLAT341_ALERT_3_B Low Bond Precision on C-C Bonds .... 0.01834 Ang.

Alert level C

PLAT029_ALERT_3_C _diffrn_measured_fraction_theta_full value Low . 0.965 Why?
PLAT082_ALERT_2_C High R1 Value .... 0.14 Report
PLAT084_ALERT_3_C High wR2 Value (i.e. > 0.25) .... 0.35 Report
PLAT241_ALERT_2_C High 'MainMol' Ueq as Compared to Neighbors of C4 Check
PLAT241_ALERT_2_C High 'MainMol' Ueq as Compared to Neighbors of C25 Check
PLAT241_ALERT_2_C High 'MainMol' Ueq as Compared to Neighbors of C39 Check
PLAT241_ALERT_2_C High 'MainMol' Ueq as Compared to Neighbors of C41 Check
PLAT242_ALERT_2_C Low 'MainMol' Ueq as Compared to Neighbors of C3 Check
PLAT242_ALERT_2_C Low 'MainMol' Ueq as Compared to Neighbors of C24 Check
PLAT242_ALERT_2_C Low 'MainMol' Ueq as Compared to Neighbors of C40 Check
PLAT244_ALERT_4_C Low 'Solvent' Ueq as Compared to Neighbors of N3 Check
PLAT244_ALERT_4_C Low 'Solvent' Ueq as Compared to Neighbors of C73 Check
PLAT260_ALERT_2_C Large Average Ueq of Residue Including O9 0.118 Check
PLAT260_ALERT_2_C Large Average Ueq of Residue Including O10 0.136 Check
PLAT317_ALERT_2_C Too many H on C in C=N Moiety in Solvent/Ion ... C71 Check
PLAT334_ALERT_2_C Small <C-C> Benzene Dist. C37 -C42 . 1.37 Ang.
PLAT334_ALERT_2_C Small <C-C> Benzene Dist. C51 -C56 . 1.37 Ang.
PLAT334_ALERT_2_C Small <C-C> Benzene Dist. C64 -C69 . 1.37 Ang.
PLAT369_ALERT_2_C Long C(sp2)-C(sp2) Bond C9 - C12 . 1.53 Ang.
PLAT369_ALERT_2_C Long C(sp2)-C(sp2) Bond C40 - C43 . 1.54 Ang.
PLAT369_ALERT_2_C Long C(sp2)-C(sp2) Bond C61 - C64 . 1.55 Ang.
PLAT906_ALERT_3_C Large K Value in the Analysis of Variance ...... 2.013 Check
PLAT911_ALERT_3_C Missing FCF Refl Between Thmin & STh/L= 0.600 732 Report

---

Alert level G

ABSMU01_ALERT_1_G Calculation of _exptl_absorpt_correction_mu
not performed for this radiation type.
PLAT003_ALERT_2_G Number of Uiso or Uij Restrained non-H Atoms ... 16 Report
PLAT004_ALERT_5_G Polymeric Structure Found with Maximum Dimension 3 Info
PLAT083_ALERT_2_G SHELXL Second Parameter in WGHT Unusually Large 50.00 Why ?
PLAT154_ALERT_1_G The s.u.'s on the Cell Angles are Equal ..(Note) 0.004 Degree
PLAT178_ALERT_4_G The CIF-Embedded .res File Contains SIMU Records 3 Report
PLAT335_ALERT_2_G Check Large C6 Ring C-C Range C6 -C11 0.22 Ang.
PLAT606_ALERT_4_G Solvent Accessible VOID(S) in Structure .... ! Info
PLAT794_ALERT_5_G Tentative Bond Valency for Zn1 (II) . 2.11 Info
PLAT794_ALERT_5_G Tentative Bond Valency for Zn2 (II) . 2.12 Info
PLAT860_ALERT_3_G Number of Least-Squares Restraints .... 84 Note
PLAT869_ALERT_4_G ALERTS Related to the Use of SQUEEZE Suppressed ! Info
PLAT870_ALERT_4_G ALERTS Related to Twinning Effects Suppressed .. ! Info
PLAT912_ALERT_4_G Missing # of FCF Reflections Above STh/L= 0.600 27 Note

```
PLAT913_ALERT_3_G Missing # of Very Strong Reflections in FCF ....          1 Note
PLAT931_ALERT_5_G CIFcalcFCF  Twin Law ( 1 0 0)        Est.d BASF       0.29 Check
PLAT933_ALERT_2_G Number of HKL-OMIT Records in Embedded .res File          1 Note
PLAT941_ALERT_3_G Average HKL Measurement Multiplicity ..........         1.0 Low
```

```
   0 ALERT level A = Most likely a serious problem - resolve or explain
   1 ALERT level B = A potentially serious problem, consider carefully
  23 ALERT level C = Check. Ensure it is not caused by an omission or oversight
  18 ALERT level G = General information/check it is not something unexpected

   2 ALERT type 1 CIF construction/syntax error, inconsistent or missing data
  21 ALERT type 2 Indicator that the structure model may be wrong or deficient
   8 ALERT type 3 Indicator that the structure quality may be low
   7 ALERT type 4 Improvement, methodology, query or suggestion
   4 ALERT type 5 Informative message, check
```

It is advisable to attempt to resolve as many as possible of the alerts in all categories. Often the minor alerts point to easily fixed oversights, errors and omissions in your CIF or refinement strategy, so attention to these fine details can be worthwhile. In order to resolve some of the more serious problems it may be necessary to carry out additional measurements or structure refinements. However, the purpose of your study may justify the reported deviations and the more serious of these should normally be commented upon in the discussion or experimental section of a paper or in the "special_details" fields of the CIF. checkCIF was carefully designed to identify outliers and unusual parameters, but every test has its limitations and alerts that are not important in a particular case may appear. Conversely, the absence of alerts does not guarantee there are no aspects of the results needing attention. It is up to the individual to critically assess their own results and, if necessary, seek expert advice.

Publication of your CIF in IUCr journals

A basic structural check has been run on your CIF. These basic checks will be run on all CIFs submitted for publication in IUCr journals (*Acta Crystallographica*, *Journal of Applied Crystallography*, *Journal of Synchrotron Radiation*); however, if you intend to submit to *Acta Crystallographica Section C* or *E* or *IUCrData*, you should make sure that full publication checks are run on the final version of your CIF prior to submission.

Publication of your CIF in other journals

Please refer to the *Notes for Authors* of the relevant journal for any special instructions relating to CIF submission.

PLATON version of 12/09/2022; check.def file version of 09/08/2022

Datablock 221031_wqangz_289057_0m_5_sq - ellipsoid plot

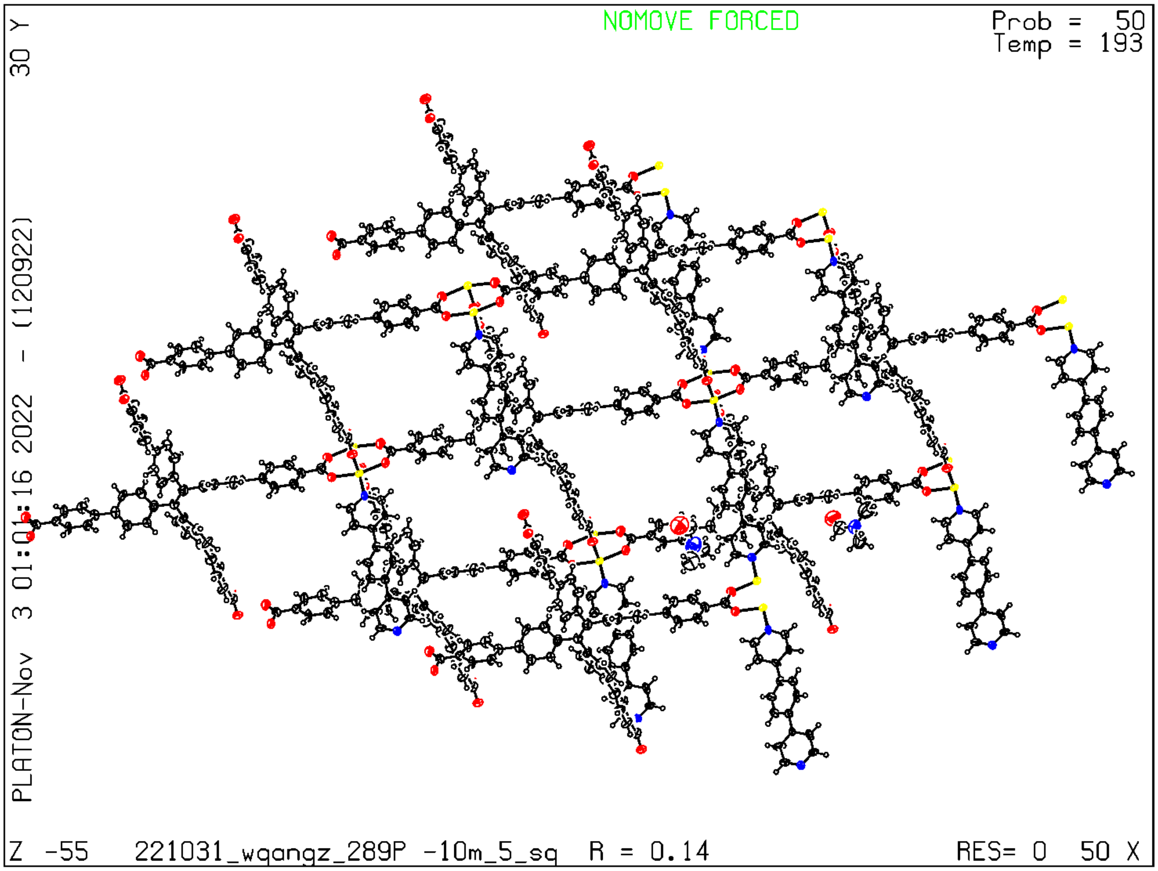

Supplement: Supplementary file 4 — Supplementary Data 1 [file 41467_2023_40594_MOESM4_ESM.zip › Supplementary Data 1/(DMF)2@Model-MOF-1.pdf]
