# Supplementary material for: Pillararene incorporated metal–organic frameworks for supramolecular recognition and selective separation
Source: Nat Commun. 2023 Aug 15;14:4927. doi: 10.1038/s41467-023-40594-2 (PMC10427641; doi:10.1038/s41467-023-40594-2)

checkCIF/PLATON report

Structure factors have been supplied for datablock(s) 221031li_lidz263361_py_0m

THIS REPORT IS FOR GUIDANCE ONLY. IF USED AS PART OF A REVIEW PROCEDURE FOR PUBLICATION, IT SHOULD NOT REPLACE THE EXPERTISE OF AN EXPERIENCED CRYSTALLOGRAPHIC REFEREE.

No syntax errors found. CIF dictionary Interpreting this report

Datablock: 221031li_lidz263361_py_0m

Bond precision:	C-C = 0.0075 A		Wavelength=1.34139
Cell:	a=17.335(3)	b=19.597(3)	c=26.871(4)
	alpha=85.269(7)	beta=78.160(7)	gamma=89.939(6)
Temperature:	193 K		

	Calculated	Reported
Volume	8903(2)	8903(2)
Space group	P -1	P -1
Hall group	-P 1	-P 1
Moiety formula	C119 H92 N2 O16 Zn2, 3(C3 H7 N O) [+ solvent]	C119 H92 N2 O16 Zn2, 3(C3 H7 N O)
Sum formula	C128 H113 N5 O19 Zn2 [+ solvent]	C128 H113 N5 O19 Zn2
Mr	2156.02	2155.97
Dx,g cm-3	0.804	0.804
Z	2	2
Mu (mm-1)	0.449	0.449
F000	2256.0	2256.0
F000'	2249.89	
h,k,lmax	20,23,32	20,23,32
Nref	32625	31475
Tmin,Tmax	0.943,0.956	0.561,0.751
Tmin'	0.943	

Correction method= # Reported T Limits: Tmin=0.561 Tmax=0.751
AbsCorr = MULTI-SCAN

Data completeness= 0.965 Theta(max)= 53.907

R(reflections)= 0.0995(20489)   wR2(reflections)= 0.3209(31475)

S = 1.168   Npar= 1400

The following ALERTS were generated. Each ALERT has the format
test-name_ALERT_alert-type_alert-level.
Click on the hyperlinks for more details of the test.

Alert level B

```
PLAT973_ALERT_2_B Check Calcd Positive Resid. Density on       Zn1       1.73 eA-3
```

Alert level C

```
PLAT029_ALERT_3_C _diffrn_measured_fraction_theta_full value Low .      0.965 Why?
PLAT084_ALERT_3_C High wR2 Value (i.e. > 0.25) ..................        0.32 Report
PLAT220_ALERT_2_C NonSolvent   Resd 1  C   Ueq(max)/Ueq(min) Range        5.7 Ratio
PLAT220_ALERT_2_C NonSolvent   Resd 1  O   Ueq(max)/Ueq(min) Range        3.2 Ratio
PLAT222_ALERT_3_C NonSolvent Resd 1  H   Uiso(max)/Uiso(min) Range        6.7 Ratio
PLAT241_ALERT_2_C High   'MainMol' Ueq as Compared to Neighbors of        C71 Check
PLAT241_ALERT_2_C High   'MainMol' Ueq as Compared to Neighbors of        C73 Check
PLAT241_ALERT_2_C High   'MainMol' Ueq as Compared to Neighbors of        C80 Check
PLAT241_ALERT_2_C High   'MainMol' Ueq as Compared to Neighbors of        C82 Check
PLAT241_ALERT_2_C High   'MainMol' Ueq as Compared to Neighbors of        C92 Check
PLAT241_ALERT_2_C High   'MainMol' Ueq as Compared to Neighbors of        C94 Check
PLAT242_ALERT_2_C Low    'MainMol' Ueq as Compared to Neighbors of        O15 Check
PLAT242_ALERT_2_C Low    'MainMol' Ueq as Compared to Neighbors of         N2 Check
PLAT242_ALERT_2_C Low    'MainMol' Ueq as Compared to Neighbors of        C78 Check
PLAT242_ALERT_2_C Low    'MainMol' Ueq as Compared to Neighbors of        C86 Check
PLAT244_ALERT_4_C Low    'Solvent' Ueq as Compared to Neighbors of         N5 Check
PLAT244_ALERT_4_C Low    'Solvent' Ueq as Compared to Neighbors of       C122 Check
PLAT244_ALERT_4_C Low    'Solvent' Ueq as Compared to Neighbors of       C125 Check
PLAT244_ALERT_4_C Low    'Solvent' Ueq as Compared to Neighbors of         N3 Check
PLAT260_ALERT_2_C Large Average Ueq of Residue Including       O17      0.148 Check
PLAT260_ALERT_2_C Large Average Ueq of Residue Including       O18      0.201 Check
PLAT260_ALERT_2_C Large Average Ueq of Residue Including       O19      0.253 Check
PLAT334_ALERT_2_C Small <C-C> Benzene Dist.   C102     -C107     .       1.37 Ang.
PLAT341_ALERT_3_C Low Bond Precision on  C-C Bonds ...............    0.00754 Ang.
PLAT412_ALERT_2_C Short Intra XH3 .. XHn     H11I     ..H112     .       1.82 Ang.
                                                   x,y,z  =      1_555 Check
PLAT412_ALERT_2_C Short Intra XH3 .. XHn     H88      ..H90C     .       1.84 Ang.
                                                   x,y,z  =      1_555 Check
PLAT412_ALERT_2_C Short Intra XH3 .. XHn     H12N     ..H12Q     .       1.85 Ang.
                                                   x,y,z  =      1_555 Check
PLAT911_ALERT_3_C Missing FCF Refl Between Thmin & STh/L=    0.600       1138 Report
PLAT918_ALERT_3_C Reflection(s) with I(obs) much Smaller I(calc) .          7 Check
PLAT973_ALERT_2_C Check Calcd Positive Resid. Density on       Zn2       1.50 eA-3
PLAT992_ALERT_5_C Repd & Actual _reflns_number_gt Values Differ by         32 Check
```

Alert level G

```
ABSMU01_ALERT_1_G  Calculation of _exptl_absorpt_correction_mu
                not performed for this radiation type.
PLAT002_ALERT_2_G Number of Distance or Angle Restraints on AtSite         15 Note
PLAT003_ALERT_2_G Number of Uiso or Uij Restrained non-H Atoms ...         15 Report
```

```
PLAT004_ALERT_5_G Polymeric Structure Found with Maximum Dimension         3 Info
PLAT072_ALERT_2_G SHELXL First  Parameter in WGHT  Unusually Large      0.20 Report
PLAT172_ALERT_4_G The CIF-Embedded .res File Contains DFIX Records        12 Report
PLAT173_ALERT_4_G The CIF-Embedded .res File Contains DANG Records         6 Report
PLAT174_ALERT_4_G The CIF-Embedded .res File Contains FLAT Records         3 Report
PLAT176_ALERT_4_G The CIF-Embedded .res File Contains SADI Records         3 Report
PLAT178_ALERT_4_G The CIF-Embedded .res File Contains SIMU Records         3 Report
PLAT187_ALERT_4_G The CIF-Embedded .res File Contains RIGU Records         3 Report
PLAT606_ALERT_4_G Solvent Accessible VOID(S) in Structure ........         ! Info
PLAT794_ALERT_5_G Tentative Bond Valency for Zn1       (II)      .      2.03 Info
PLAT794_ALERT_5_G Tentative Bond Valency for Zn2       (II)      .      2.06 Info
PLAT860_ALERT_3_G Number of Least-Squares Restraints .............       171 Note
PLAT912_ALERT_4_G Missing # of FCF Reflections Above STh/L=  0.600        14 Note
PLAT913_ALERT_3_G Missing # of Very Strong Reflections in FCF ....         1 Note
PLAT933_ALERT_2_G Number of HKL-OMIT Records in Embedded .res File        30 Note
PLAT941_ALERT_3_G Average HKL Measurement Multiplicity ...........       2.7 Low
PLAT978_ALERT_2_G Number C-C Bonds with Positive Residual Density.         0 Info
```

---

0 **ALERT level A** = Most likely a serious problem - resolve or explain
1 **ALERT level B** = A potentially serious problem, consider carefully
31 **ALERT level C** = Check. Ensure it is not caused by an omission or oversight
20 **ALERT level G** = General information/check it is not something unexpected

1 ALERT type 1 CIF construction/syntax error, inconsistent or missing data
26 ALERT type 2 Indicator that the structure model may be wrong or deficient
9 ALERT type 3 Indicator that the structure quality may be low
12 ALERT type 4 Improvement, methodology, query or suggestion
4 ALERT type 5 Informative message, check

It is advisable to attempt to resolve as many as possible of the alerts in all categories. Often the minor alerts point to easily fixed oversights, errors and omissions in your CIF or refinement strategy, so attention to these fine details can be worthwhile. In order to resolve some of the more serious problems it may be necessary to carry out additional measurements or structure refinements. However, the purpose of your study may justify the reported deviations and the more serious of these should normally be commented upon in the discussion or experimental section of a paper or in the "special_details" fields of the CIF. checkCIF was carefully designed to identify outliers and unusual parameters, but every test has its limitations and alerts that are not important in a particular case may appear. Conversely, the absence of alerts does not guarantee there are no aspects of the results needing attention. It is up to the individual to critically assess their own results and, if necessary, seek expert advice.

Publication of your CIF in IUCr journals

A basic structural check has been run on your CIF. These basic checks will be run on all CIFs submitted for publication in IUCr journals (*Acta Crystallographica*, *Journal of Applied Crystallography*, *Journal of Synchrotron Radiation*); however, if you intend to submit to *Acta Crystallographica Section C* or *E* or *IUCrData*, you should make sure that full publication checks are run on the final version of your CIF prior to submission.

Publication of your CIF in other journals

Please refer to the *Notes for Authors* of the relevant journal for any special instructions relating to CIF submission.

PLATON version of 12/09/2022; check.def file version of 09/08/2022

Datablock 221031li_lidz263361_py_0m - ellipsoid plot

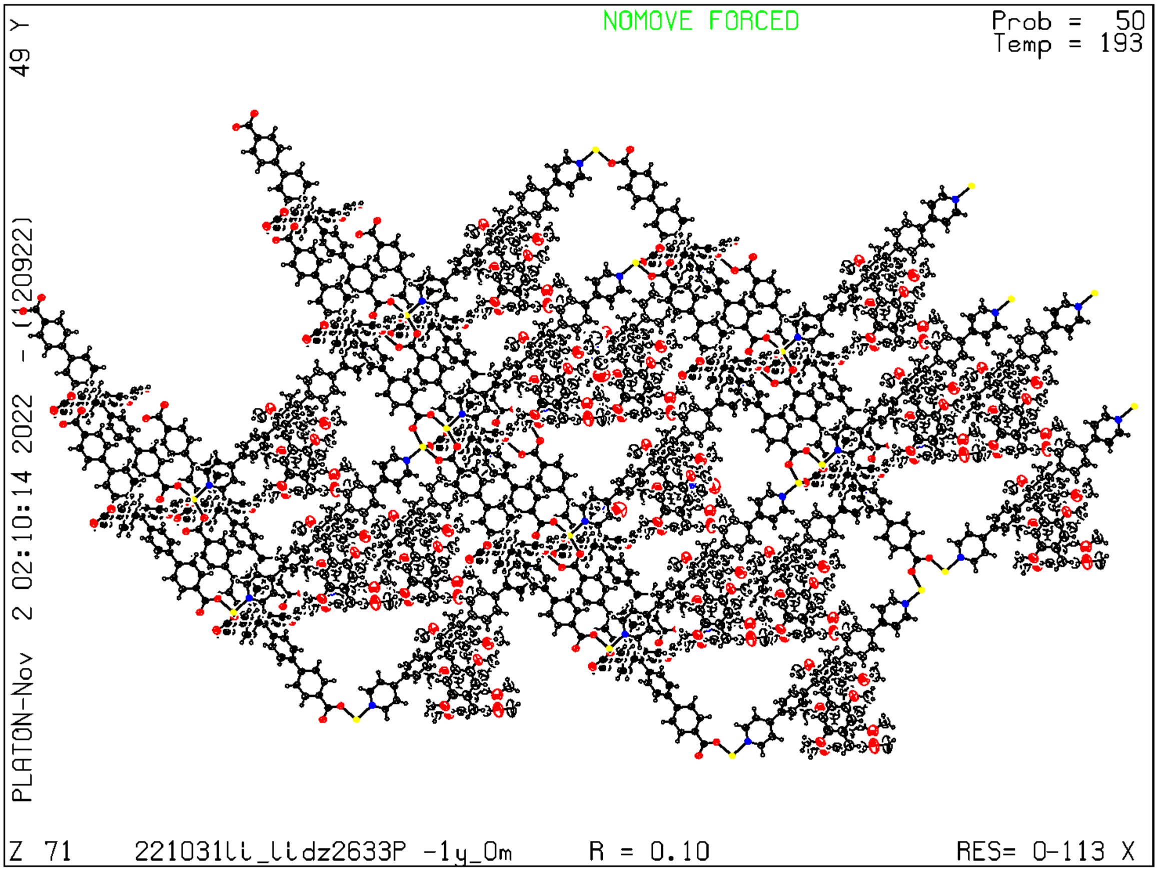

Supplement: Supplementary file 4 — Supplementary Data 1 [file 41467_2023_40594_MOESM4_ESM.zip › Supplementary Data 1/(DMF)3@MeP5-MOF-2.pdf]
