# Supplementary material for: Pillararene incorporated metal–organic frameworks for supramolecular recognition and selective separation
Source: Nat Commun. 2023 Aug 15;14:4927. doi: 10.1038/s41467-023-40594-2 (PMC10427641; doi:10.1038/s41467-023-40594-2)

checkCIF/PLATON report

Structure factors have been supplied for datablock(s) 221013li_lidz098964_0m

THIS REPORT IS FOR GUIDANCE ONLY. IF USED AS PART OF A REVIEW PROCEDURE FOR PUBLICATION, IT SHOULD NOT REPLACE THE EXPERTISE OF AN EXPERIENCED CRYSTALLOGRAPHIC REFEREE.

No syntax errors found. CIF dictionary Interpreting this report

Datablock: 221013li_lidz098964_0m

Bond precision: C-C = 0.0037 A Wavelength=1.34139

Cell:	a=12.1492(5)	b=19.8813(7)	c=24.9952(10)
	alpha=90	beta=90	gamma=90
Temperature:	193 K		

	Calculated	Reported
Volume	6037.4(4)	6037.4(4)
Space group	P c c n	P c c n
Hall group	-P 2ab 2ac	-P 2ab 2ac
Moiety formula	C55 H70 O10, 2(C5 H5 N)	C55 H70 O10, 2(C5 H5 N)
Sum formula	C65 H80 N2 O10	C65 H80 N2 O10
Mr	1049.31	1049.31
Dx,g cm-3	1.154	1.154
Z	4	4
Mu (mm-1)	0.392	0.392
F000	2256.0	2256.0
F000'	2261.02	
h,k,lmax	15,25,32	15,25,31
Nref	6845	6718
Tmin,Tmax	0.950,0.962	0.622,0.752
Tmin'	0.950	

Correction method= # Reported T Limits: Tmin=0.622 Tmax=0.752
AbsCorr = MULTI-SCAN

Data completeness= 0.981 Theta(max)= 60.175

R(reflections)= 0.0617(3935) wR2(reflections)= 0.1824(6718)

S = 1.026 Npar= 393

The following ALERTS were generated. Each ALERT has the format
test-name_ALERT_alert-type_alert-level.
Click on the hyperlinks for more details of the test.

Alert level C

```
PLAT094_ALERT_2_C Ratio of Maximum / Minimum Residual Density ....       2.27 Report
PLAT250_ALERT_2_C Large U3/U1 Ratio for Average U(i,j) Tensor ....        2.1 Note
PLAT260_ALERT_2_C Large Average Ueq of Residue Including        N1      0.131 Check
PLAT906_ALERT_3_C Large K Value in the Analysis of Variance ......      7.577 Check
PLAT911_ALERT_3_C Missing FCF Refl Between Thmin & STh/L=    0.600          4 Report
```

Alert level G

```
ABSMU01_ALERT_1_G  Calculation of _exptl_absorpt_correction_mu
                 not performed for this radiation type.
PLAT002_ALERT_2_G Number of Distance or Angle Restraints on AtSite         10 Note
PLAT003_ALERT_2_G Number of Uiso or Uij Restrained non-H Atoms ...          8 Report
PLAT172_ALERT_4_G The CIF-Embedded .res File Contains DFIX Records          2 Report
PLAT176_ALERT_4_G The CIF-Embedded .res File Contains SADI Records          4 Report
PLAT178_ALERT_4_G The CIF-Embedded .res File Contains SIMU Records          2 Report
PLAT186_ALERT_4_G The CIF-Embedded .res File Contains ISOR Records          1 Report
PLAT300_ALERT_4_G Atom Site Occupancy of H1A       Constrained at         0.5 Check
PLAT300_ALERT_4_G Atom Site Occupancy of H1B       Constrained at         0.5 Check
PLAT301_ALERT_3_G Main Residue  Disorder .............(Resd  1  )         12% Note
PLAT367_ALERT_2_G Long?  C(sp?)-C(sp?) Bond  C1       - C2       .       1.51 Ang.
PLAT367_ALERT_2_G Long?  C(sp?)-C(sp?) Bond  C1       - C2_a     .       1.51 Ang.
PLAT720_ALERT_4_G Number of Unusual/Non-Standard Labels .........          10 Note
PLAT860_ALERT_3_G Number of Least-Squares Restraints ............         104 Note
PLAT912_ALERT_4_G Missing # of FCF Reflections Above STh/L=  0.600        114 Note
PLAT913_ALERT_3_G Missing # of Very Strong Reflections in FCF ....          3 Note
PLAT978_ALERT_2_G Number C-C Bonds with Positive Residual Density.          0 Info
```

0 **ALERT level A** = Most likely a serious problem - resolve or explain
0 **ALERT level B** = A potentially serious problem, consider carefully
5 **ALERT level C** = Check. Ensure it is not caused by an omission or oversight
17 **ALERT level G** = General information/check it is not something unexpected

1 ALERT type 1 CIF construction/syntax error, inconsistent or missing data
8 ALERT type 2 Indicator that the structure model may be wrong or deficient
5 ALERT type 3 Indicator that the structure quality may be low
8 ALERT type 4 Improvement, methodology, query or suggestion
0 ALERT type 5 Informative message, check

It is advisable to attempt to resolve as many as possible of the alerts in all categories. Often the minor alerts point to easily fixed oversights, errors and omissions in your CIF or refinement strategy, so attention to these fine details can be worthwhile. In order to resolve some of the more serious problems it may be necessary to carry out additional measurements or structure refinements. However, the purpose of your study may justify the reported deviations and the more serious of these should normally be commented upon in the discussion or experimental section of a paper or in the "special_details" fields of the CIF. checkCIF was carefully designed to identify outliers and unusual parameters, but every test has its limitations and alerts that are not important in a particular case may appear. Conversely, the absence of alerts does not guarantee there are no aspects of the results needing attention. It is up to the individual to critically assess their own results and, if necessary, seek expert advice.

Publication of your CIF in IUCr journals

A basic structural check has been run on your CIF. These basic checks will be run on all CIFs submitted for publication in IUCr journals (*Acta Crystallographica*, *Journal of Applied Crystallography*, *Journal of Synchrotron Radiation*); however, if you intend to submit to *Acta Crystallographica Section C* or *E* or *IUCrData*, you should make sure that full publication checks are run on the final version of your CIF prior to submission.

Publication of your CIF in other journals

Please refer to the *Notes for Authors* of the relevant journal for any special instructions relating to CIF submission.

PLATON version of 12/09/2022; check.def file version of 09/08/2022

Datablock 221013li_lidz098964_0m - ellipsoid plot

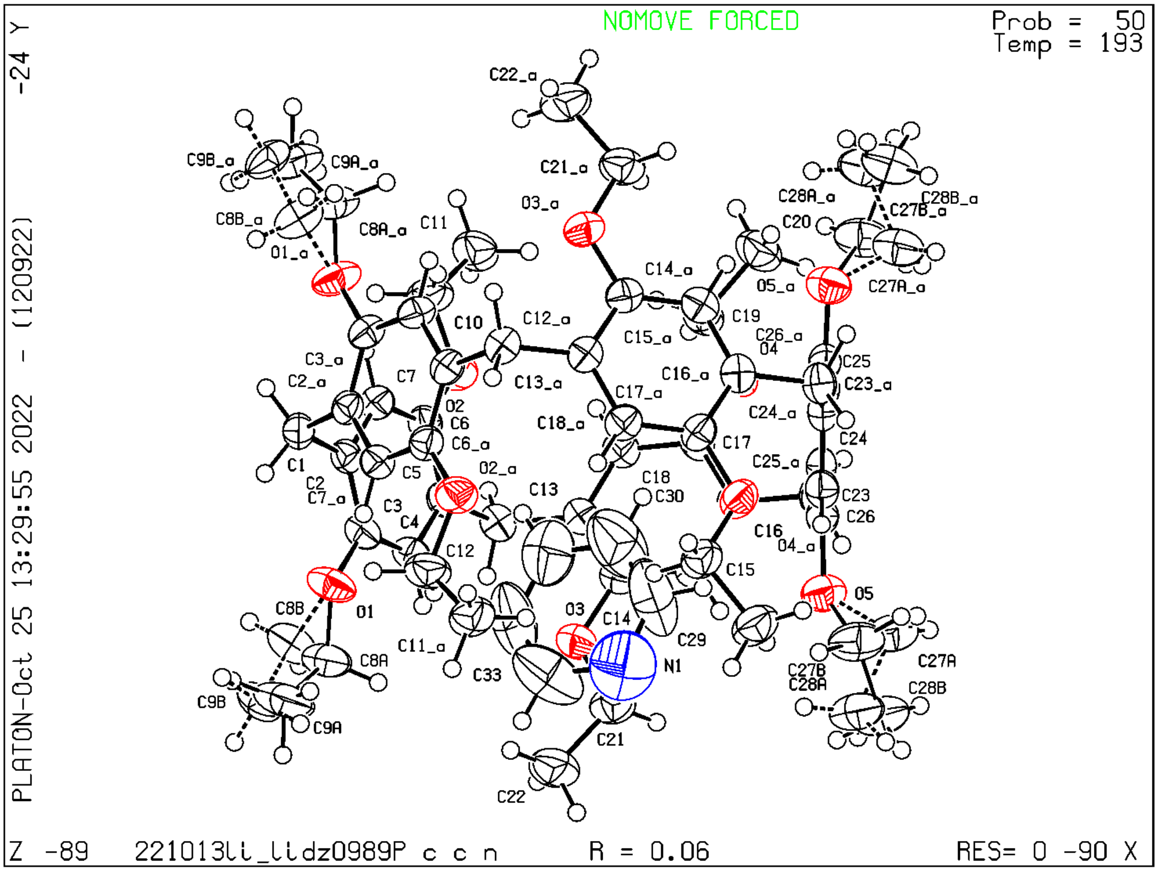

Supplement: Supplementary file 4 — Supplementary Data 1 [file 41467_2023_40594_MOESM4_ESM.zip › Supplementary Data 1/(Py)2@P5.pdf]
