# Supplementary material for: Pillararene incorporated metal–organic frameworks for supramolecular recognition and selective separation
Source: Nat Commun. 2023 Aug 15;14:4927. doi: 10.1038/s41467-023-40594-2 (PMC10427641; doi:10.1038/s41467-023-40594-2)

checkCIF/PLATON report

Structure factors have been supplied for datablock(s) a1_sq

THIS REPORT IS FOR GUIDANCE ONLY. IF USED AS PART OF A REVIEW PROCEDURE FOR PUBLICATION, IT SHOULD NOT REPLACE THE EXPERTISE OF AN EXPERIENCED CRYSTALLOGRAPHIC REFEREE.

No syntax errors found. CIF dictionary Interpreting this report

Datablock: a1_sq

Bond precision:	C-C = 0.0120 A		Wavelength=1.34139
Cell:	a=16.9571(13)	b=19.8151(14)	c=26.719(3)
	alpha=83.462(6)	beta=71.545(4)	gamma=89.884(4)
Temperature:	193 K		

	Calculated	Reported
Volume	8455.2(13)	8455.3(12)
Space group	P -1	P -1
Hall group	-P 1	-P 1
Moiety formula	C119 H92 N2 O16 Zn2, C3 H7 N O [+ solvent]	C119 H92 N2 O16 Zn2, C3 H7 N O
Sum formula	C122 H99 N3 O17 Zn2 [+ solvent]	C122 H99 N3 O17 Zn2
Mr	2009.83	2009.78
Dx,g cm-3	0.789	0.789
Z	2	2
Mu (mm-1)	0.450	0.450
F000	2096.0	2096.0
F000'	2089.51	
h,k,lmax	20,23,32	20,23,32
Nref	31000	30837
Tmin,Tmax	0.943,0.956	0.569,0.751
Tmin'	0.943	

Correction method= # Reported T Limits: Tmin=0.569 Tmax=0.751
AbsCorr = MULTI-SCAN

Data completeness= 0.995 Theta(max)= 53.906

R(reflections)= 0.1049(14171) wR2(reflections)= 0.3402(30837)

S = 0.999 Npar= 1322

The following ALERTS were generated. Each ALERT has the format
test-name_ALERT_alert-type_alert-level.
Click on the hyperlinks for more details of the test.

Alert level C

```
PLAT026_ALERT_3_C Ratio Observed / Unique Reflections (too) Low ..       46% Check
PLAT084_ALERT_3_C High wR2 Value (i.e. > 0.25) ...................      0.34 Report
PLAT220_ALERT_2_C NonSolvent   Resd 1  C   Ueq(max)/Ueq(min) Range       5.8 Ratio
PLAT220_ALERT_2_C NonSolvent   Resd 1  O   Ueq(max)/Ueq(min) Range       4.5 Ratio
PLAT222_ALERT_3_C NonSolvent Resd 1  H   Uiso(max)/Uiso(min) Range       6.9 Ratio
PLAT241_ALERT_2_C High   'MainMol' Ueq as Compared to Neighbors of       C77 Check
PLAT242_ALERT_2_C Low    'MainMol' Ueq as Compared to Neighbors of        N1 Check
PLAT242_ALERT_2_C Low    'MainMol' Ueq as Compared to Neighbors of       C54 Check
PLAT244_ALERT_4_C Low    'Solvent' Ueq as Compared to Neighbors of        N3 Check
PLAT244_ALERT_4_C Low    'Solvent' Ueq as Compared to Neighbors of      C116 Check
PLAT260_ALERT_2_C Large Average Ueq of Residue Including       Zn1     0.106 Check
PLAT260_ALERT_2_C Large Average Ueq of Residue Including       O17     0.215 Check
PLAT341_ALERT_3_C Low Bond Precision on  C-C Bonds ...............   0.01202 Ang.
PLAT412_ALERT_2_C Short Intra XH3 .. XHn     H11M      ..H69      .     1.81 Ang.
                                                      x,y,z  =      1_555 Check
PLAT905_ALERT_3_C Negative K value in the Analysis of Variance ...    -2.579 Report
PLAT911_ALERT_3_C Missing FCF Refl Between Thmin & STh/L=    0.600       164 Report
```

Alert level G

```
ABSMU01_ALERT_1_G  Calculation of _exptl_absorpt_correction_mu
                not performed for this radiation type.
PLAT002_ALERT_2_G Number of Distance or Angle Restraints on AtSite        25 Note
PLAT003_ALERT_2_G Number of Uiso or Uij Restrained non-H Atoms ...        72 Report
PLAT004_ALERT_5_G Polymeric Structure Found with Maximum Dimension         3 Info
PLAT072_ALERT_2_G SHELXL First  Parameter in WGHT  Unusually Large      0.19 Report
PLAT172_ALERT_4_G The CIF-Embedded .res File Contains DFIX Records         2 Report
PLAT173_ALERT_4_G The CIF-Embedded .res File Contains DANG Records         6 Report
PLAT176_ALERT_4_G The CIF-Embedded .res File Contains SADI Records        13 Report
PLAT178_ALERT_4_G The CIF-Embedded .res File Contains SIMU Records         4 Report
PLAT186_ALERT_4_G The CIF-Embedded .res File Contains ISOR Records         2 Report
PLAT301_ALERT_3_G Main Residue  Disorder ..............(Resd  1  )        6% Note
PLAT410_ALERT_2_G Short Intra H...H Contact  H92       ..H79B     .     2.03 Ang.
                                                      x,y,z  =      1_555 Check
PLAT606_ALERT_4_G Solvent Accessible VOID(S) in Structure ........         ! Info
PLAT720_ALERT_4_G Number of Unusual/Non-Standard Labels ..........         6 Note
PLAT794_ALERT_5_G Tentative Bond Valency for Zn1        (II)      .     2.07 Info
PLAT860_ALERT_3_G Number of Least-Squares Restraints .............       866 Note
PLAT869_ALERT_4_G ALERTS Related to the Use of SQUEEZE  Suppressed         ! Info
PLAT870_ALERT_4_G ALERTS Related to Twinning Effects Suppressed ..         ! Info
PLAT912_ALERT_4_G Missing # of FCF Reflections Above STh/L=  0.600         1 Note
PLAT913_ALERT_3_G Missing # of Very Strong Reflections in FCF ....         1 Note
PLAT931_ALERT_5_G CIFcalcFCF  Twin Law ( 2 0 1)          Est.d BASF     0.06 Check
PLAT933_ALERT_2_G Number of HKL-OMIT Records in Embedded .res File         7 Note
PLAT941_ALERT_3_G Average HKL Measurement Multiplicity ...........       2.3 Low
```

```
PLAT992_ALERT_5_G Repd & Actual _reflns_number_gt Values Differ by          5 Check
```

```
   0 ALERT level A = Most likely a serious problem - resolve or explain
   0 ALERT level B = A potentially serious problem, consider carefully
  16 ALERT level C = Check. Ensure it is not caused by an omission or oversight
  24 ALERT level G = General information/check it is not something unexpected

   1 ALERT type 1 CIF construction/syntax error, inconsistent or missing data
  13 ALERT type 2 Indicator that the structure model may be wrong or deficient
  10 ALERT type 3 Indicator that the structure quality may be low
  12 ALERT type 4 Improvement, methodology, query or suggestion
   4 ALERT type 5 Informative message, check
```

It is advisable to attempt to resolve as many as possible of the alerts in all categories. Often the minor alerts point to easily fixed oversights, errors and omissions in your CIF or refinement strategy, so attention to these fine details can be worthwhile. In order to resolve some of the more serious problems it may be necessary to carry out additional measurements or structure refinements. However, the purpose of your study may justify the reported deviations and the more serious of these should normally be commented upon in the discussion or experimental section of a paper or in the "special_details" fields of the CIF. checkCIF was carefully designed to identify outliers and unusual parameters, but every test has its limitations and alerts that are not important in a particular case may appear. Conversely, the absence of alerts does not guarantee there are no aspects of the results needing attention. It is up to the individual to critically assess their own results and, if necessary, seek expert advice.

Publication of your CIF in IUCr journals

A basic structural check has been run on your CIF. These basic checks will be run on all CIFs submitted for publication in IUCr journals (*Acta Crystallographica*, *Journal of Applied Crystallography*, *Journal of Synchrotron Radiation*); however, if you intend to submit to *Acta Crystallographica Section C* or *E* or *IUCrData*, you should make sure that full publication checks are run on the final version of your CIF prior to submission.

Publication of your CIF in other journals

Please refer to the *Notes for Authors* of the relevant journal for any special instructions relating to CIF submission.

PLATON version of 18/05/2022; check.def file version of 17/05/2022

Datablock a1_sq - ellipsoid plot

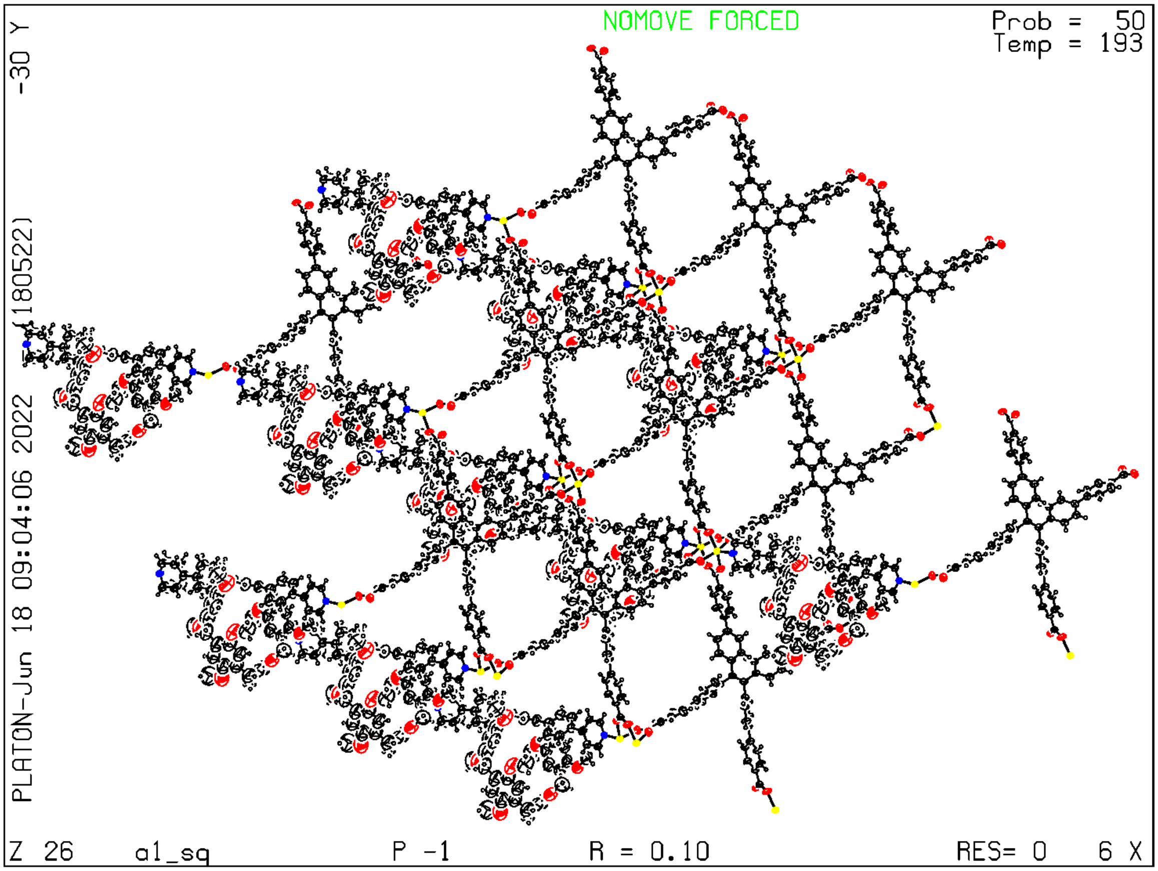

Supplement: Supplementary file 4 — Supplementary Data 1 [file 41467_2023_40594_MOESM4_ESM.zip › Supplementary Data 1/DMF@MeP5-MOF-2.pdf]
