# Supplementary material for: Pillararene incorporated metal–organic frameworks for supramolecular recognition and selective separation
Source: Nat Commun. 2023 Aug 15;14:4927. doi: 10.1038/s41467-023-40594-2 (PMC10427641; doi:10.1038/s41467-023-40594-2)

checkCIF/PLATON report

Structure factors have been supplied for datablock(s) a

THIS REPORT IS FOR GUIDANCE ONLY. IF USED AS PART OF A REVIEW PROCEDURE FOR PUBLICATION, IT SHOULD NOT REPLACE THE EXPERTISE OF AN EXPERIENCED CRYSTALLOGRAPHIC REFEREE.

No syntax errors found. CIF dictionary Interpreting this report

Datablock: a

```
Bond precision:   C-C = 0.0091 A          Wavelength=1.34139

Cell:               a=16.181(12)      b=18.24(1)      c=20.371(15)
                    alpha=90          beta=90         gamma=90
Temperature:        105 K

                 Calculated                    Reported
Volume           6012(7)                       6012(7)
Space group      P m m m                       P m m m
Hall group       -P 2 2                        -P 2 2
Moiety formula   C35 H22 N O4 Zn [+ solvent] C35 H22 N O4 Zn1
Sum formula      C35 H22 N O4 Zn [+ solvent] C35 H22 N O4 Zn
Mr               585.93                        585.90
Dx,g cm-3        0.324                         0.324
Z                2                             2
Mu (mm-1)        0.236                         0.236
F000             602.0                         602.0
F000'            597.72
h,k,lmax         19,21,24                      19,21,24
Nref             6091                          6087
Tmin,Tmax        0.970,0.977                   0.552,0.752
Tmin'            0.970

Correction method= # Reported T Limits: Tmin=0.552 Tmax=0.752
AbsCorr = MULTI-SCAN

Data completeness= 0.999           Theta(max)= 53.906

R(reflections)= 0.1298( 4790)                          wR2(reflections)=
                                                       0.3135( 6087)
S = 0.957                   Npar= 151
```

The following ALERTS were generated. Each ALERT has the format
test-name_ALERT_alert-type_alert-level.
Click on the hyperlinks for more details of the test.

Alert level C

```
PLAT082_ALERT_2_C High R1 Value ..................................       0.13 Report
PLAT084_ALERT_3_C High wR2 Value (i.e. > 0.25) ...................       0.31 Report
PLAT241_ALERT_2_C High   'MainMol' Ueq as Compared to Neighbors of        O1 Check
PLAT242_ALERT_2_C Low    'MainMol' Ueq as Compared to Neighbors of       C15 Check
PLAT242_ALERT_2_C Low    'MainMol' Ueq as Compared to Neighbors of       C16 Check
PLAT260_ALERT_2_C Large Average Ueq of Residue Including       Zn1      0.107 Check
PLAT313_ALERT_2_C Oxygen with Three Covalent Bonds (rare) ........        O1 Check
PLAT341_ALERT_3_C Low Bond Precision on  C-C Bonds ...............   0.00908 Ang.
PLAT911_ALERT_3_C Missing FCF Refl Between Thmin & STh/L=    0.600          2 Report
PLAT918_ALERT_3_C Reflection(s) with I(obs) much Smaller I(calc) .          2 Check
PLAT976_ALERT_2_C Check Calcd Resid. Dens.  1.03Ang From O1      .      -0.41 eA-3
```

Alert level G

```
ABSMU01_ALERT_1_G  Calculation of _exptl_absorpt_correction_mu
                 not performed for this radiation type.
PLAT003_ALERT_2_G Number of Uiso or Uij Restrained non-H Atoms ...          5 Report
PLAT004_ALERT_5_G Polymeric Structure Found with Maximum Dimension          3 Info
PLAT042_ALERT_1_G Calc. and Reported MoietyFormula Strings  Differ     Please Check
PLAT072_ALERT_2_G SHELXL First  Parameter in WGHT  Unusually Large       0.12 Report
PLAT083_ALERT_2_G SHELXL Second Parameter in WGHT  Unusually Large       8.00 Why ?
PLAT178_ALERT_4_G The CIF-Embedded .res File Contains SIMU Records          1 Report
PLAT186_ALERT_4_G The CIF-Embedded .res File Contains ISOR Records          2 Report
PLAT188_ALERT_3_G A Non-default SIMU Restraint Value has been used     0.0050 Report
PLAT232_ALERT_2_G Hirshfeld Test Diff (M-X)  Zn1      --N1       .       31.3 s.u.
PLAT232_ALERT_2_G Hirshfeld Test Diff (M-X)  Zn2      --O1       .       16.5 s.u.
PLAT232_ALERT_2_G Hirshfeld Test Diff (M-X)  Zn2      --N1       .       17.5 s.u.
PLAT232_ALERT_2_G Hirshfeld Test Diff (M-X)  Zn3      --O1       .       11.3 s.u.
PLAT232_ALERT_2_G Hirshfeld Test Diff (M-X)  Zn3      --N1       .       14.0 s.u.
PLAT232_ALERT_2_G Hirshfeld Test Diff (M-X)  Zn4      --O1       .       10.3 s.u.
PLAT300_ALERT_4_G Atom Site Occupancy of Zn1        Constrained at       0.56 Check
PLAT300_ALERT_4_G Atom Site Occupancy of Zn2        Constrained at        0.2 Check
PLAT300_ALERT_4_G Atom Site Occupancy of Zn3        Constrained at        0.2 Check
PLAT300_ALERT_4_G Atom Site Occupancy of Zn4        Constrained at       0.04 Check
PLAT300_ALERT_4_G Atom Site Occupancy of C7         Constrained at        0.5 Check
PLAT300_ALERT_4_G Atom Site Occupancy of C8         Constrained at        0.5 Check
PLAT300_ALERT_4_G Atom Site Occupancy of C10        Constrained at        0.5 Check
PLAT300_ALERT_4_G Atom Site Occupancy of C11        Constrained at        0.5 Check
PLAT300_ALERT_4_G Atom Site Occupancy of C13        Constrained at        0.5 Check
PLAT300_ALERT_4_G Atom Site Occupancy of C14        Constrained at        0.5 Check
PLAT300_ALERT_4_G Atom Site Occupancy of H7         Constrained at        0.5 Check
PLAT300_ALERT_4_G Atom Site Occupancy of H8         Constrained at        0.5 Check
PLAT300_ALERT_4_G Atom Site Occupancy of H10        Constrained at        0.5 Check
PLAT300_ALERT_4_G Atom Site Occupancy of H11        Constrained at        0.5 Check
PLAT300_ALERT_4_G Atom Site Occupancy of H13        Constrained at        0.5 Check
PLAT300_ALERT_4_G Atom Site Occupancy of H14        Constrained at        0.5 Check
PLAT301_ALERT_3_G Main Residue  Disorder ..............(Resd  1  )        32% Note
PLAT606_ALERT_4_G Solvent Accessible VOID(S) in Structure ........          ! Info
PLAT773_ALERT_2_G Check long C-C Bond in CIF: C7       --C10             1.89 Ang.
PLAT773_ALERT_2_G Check long C-C Bond in CIF: C13      --C14             1.94 Ang.
```

```
PLAT773_ALERT_2_G Check long C-C Bond in CIF: C11      --C8             1.99 Ang.
PLAT773_ALERT_2_G Check long C-C Bond in CIF: C8       --C8             2.03 Ang.
PLAT779_ALERT_4_G Suspect or Irrelevant (Bond) Angle(s) in CIF ...      0.00 Deg.
              C1   -O1   -C1      8_565   1_555   1_555 ......     #   18 Check
PLAT779_ALERT_4_G Suspect or Irrelevant (Bond) Angle(s) in CIF ...      0.00 Deg.
              C1   -C2   -C1      1_555   1_555   8_565 ......     #   43 Check
PLAT779_ALERT_4_G Suspect or Irrelevant (Bond) Angle(s) in CIF ...      0.00 Deg.
              C1   -C1   -O1      8_565   1_555   1_555 ......     #   55 Check
PLAT779_ALERT_4_G Suspect or Irrelevant (Bond) Angle(s) in CIF ...      0.00 Deg.
              C1   -C1   -O1      8_565   1_555   8_565 ......     #   56 Check
PLAT779_ALERT_4_G Suspect or Irrelevant (Bond) Angle(s) in CIF ...      0.00 Deg.
              C1   -C1   -C2      8_565   1_555   1_555 ......     #   57 Check
PLAT779_ALERT_4_G Suspect or Irrelevant (Bond) Angle(s) in CIF ...      0.00 Deg.
              C1   -C1   -ZN4     8_565   1_555   5_667 ......     #   58 Check
PLAT779_ALERT_4_G Suspect or Irrelevant (Bond) Angle(s) in CIF ...     31.20 Deg.
              N1   -C13  -ZN3     1_555   1_555   1_555 ......     #  100 Check
PLAT779_ALERT_4_G Suspect or Irrelevant (Bond) Angle(s) in CIF ...     42.70 Deg.
              C7   -C11  -C8      8_565   1_555   8_565 ......     #  112 Check
PLAT779_ALERT_4_G Suspect or Irrelevant (Bond) Angle(s) in CIF ...     44.60 Deg.
              C10  -C11  -C8      1_555   1_555   8_565 ......     #  114 Check
PLAT779_ALERT_4_G Suspect or Irrelevant (Bond) Angle(s) in CIF ...     43.80 Deg.
              C9   -C8   -C8      1_555   1_555   8_565 ......     #  122 Check
PLAT811_ALERT_5_G No ADDSYM Analysis: Too Many Excluded Atoms ....         ! Info
PLAT860_ALERT_3_G Number of Least-Squares Restraints ............         66 Note
PLAT868_ALERT_4_G ALERTS Due to the Use of _smtbx_masks Suppressed         ! Info
PLAT910_ALERT_3_G Missing # of FCF Reflection(s) Below Theta(Min).         2 Note
PLAT913_ALERT_3_G Missing # of Very Strong Reflections in FCF ....         1 Note
PLAT933_ALERT_2_G Number of HKL-OMIT Records in Embedded .res File         1 Note
PLAT978_ALERT_2_G Number C-C Bonds with Positive Residual Density.         0 Info
```

---

0 **ALERT level A** = Most likely a serious problem - resolve or explain
0 **ALERT level B** = A potentially serious problem, consider carefully
11 **ALERT level C** = Check. Ensure it is not caused by an omission or oversight
54 **ALERT level G** = General information/check it is not something unexpected

2 ALERT type 1 CIF construction/syntax error, inconsistent or missing data
22 ALERT type 2 Indicator that the structure model may be wrong or deficient
9 ALERT type 3 Indicator that the structure quality may be low
30 ALERT type 4 Improvement, methodology, query or suggestion
2 ALERT type 5 Informative message, check

It is advisable to attempt to resolve as many as possible of the alerts in all categories. Often the minor alerts point to easily fixed oversights, errors and omissions in your CIF or refinement strategy, so attention to these fine details can be worthwhile. In order to resolve some of the more serious problems it may be necessary to carry out additional measurements or structure refinements. However, the purpose of your study may justify the reported deviations and the more serious of these should normally be commented upon in the discussion or experimental section of a paper or in the "special_details" fields of the CIF. checkCIF was carefully designed to identify outliers and unusual parameters, but every test has its limitations and alerts that are not important in a particular case may appear. Conversely, the absence of alerts does not guarantee there are no aspects of the results needing attention. It is up to the individual to critically assess their own results and, if necessary, seek expert advice.

Publication of your CIF in IUCr journals

A basic structural check has been run on your CIF. These basic checks will be run on all CIFs submitted for publication in IUCr journals (*Acta Crystallographica*, *Journal of Applied Crystallography*, *Journal of Synchrotron Radiation*); however, if you intend to submit to *Acta Crystallographica Section C* or *E* or *IUCrData*, you should make sure that full publication checks are run on the final version of your CIF prior to submission.

Publication of your CIF in other journals

Please refer to the *Notes for Authors* of the relevant journal for any special instructions relating to CIF submission.

PLATON version of 10/05/2023; check.def file version of 10/05/2023

Datablock a - ellipsoid plot

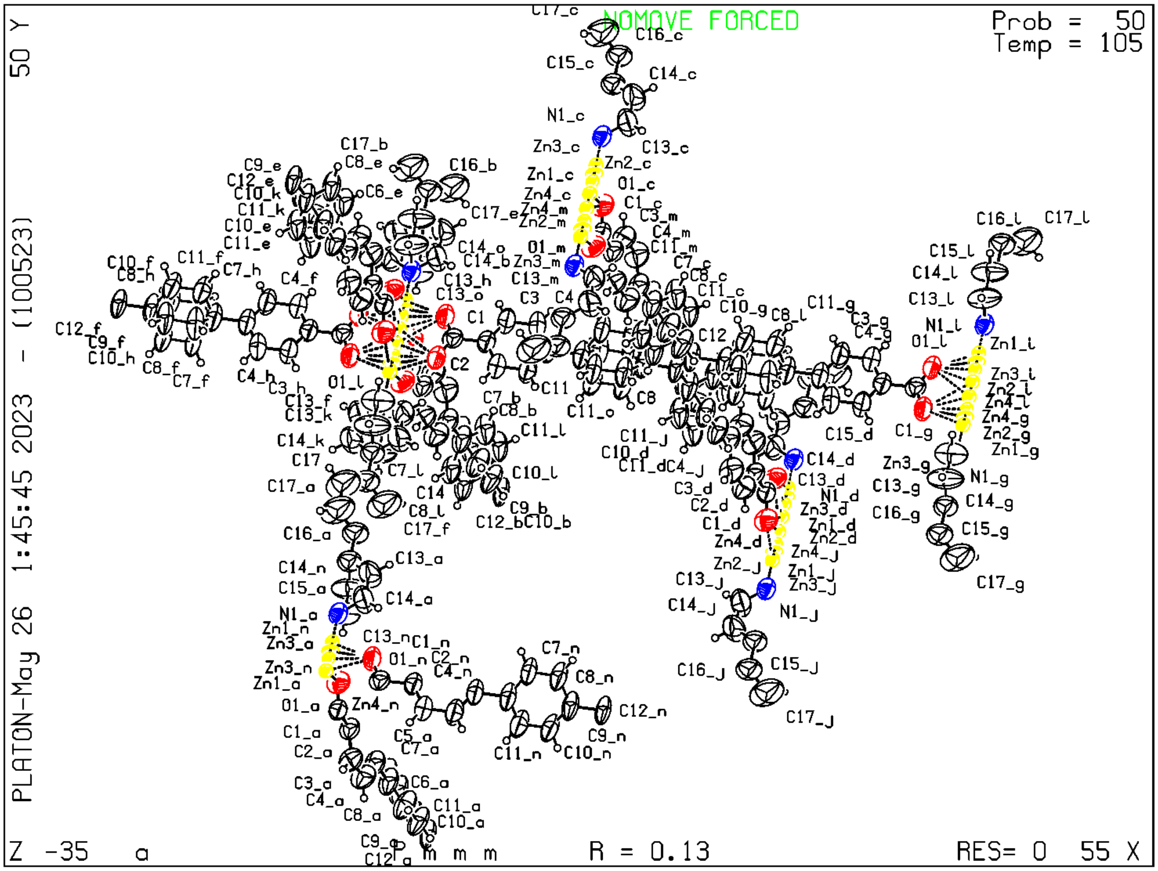

Supplement: Supplementary file 4 — Supplementary Data 1 [file 41467_2023_40594_MOESM4_ESM.zip › Supplementary Data 1/MeP5-MOF-1-105K.pdf]
