# Supplementary material for: Pillararene incorporated metal–organic frameworks for supramolecular recognition and selective separation
Source: Nat Commun. 2023 Aug 15;14:4927. doi: 10.1038/s41467-023-40594-2 (PMC10427641; doi:10.1038/s41467-023-40594-2)

checkCIF/PLATON report

Structure factors have been supplied for datablock(s) a

THIS REPORT IS FOR GUIDANCE ONLY. IF USED AS PART OF A REVIEW PROCEDURE FOR PUBLICATION, IT SHOULD NOT REPLACE THE EXPERTISE OF AN EXPERIENCED CRYSTALLOGRAPHIC REFEREE.

No syntax errors found. CIF dictionary Interpreting this report

Datablock: a

```
Bond precision:   C-C = 0.0113 A          Wavelength=1.54178

Cell:               a=16.143(3)      b=18.713(5)      c=20.483(3)
                    alpha=90         beta=90          gamma=90
Temperature:        193 K

                Calculated                    Reported
Volume          6188(2)                       6187(2)
Space group     P m m m                       P m m m
Hall group      -P 2 2                        -P 2 2
Moiety formula  C35 H22 N O4 Zn [+ solvent] C35 H22 N O4 Zn
Sum formula     C35 H22 N O4 Zn [+ solvent] C35 H22 N O4 Zn
Mr              585.93                        585.90
Dx,g cm-3       0.315                         0.314
Z               2                             2
Mu (mm-1)       0.350                         0.350
F000            602.0                         602.0
F000'           600.53
h,k,lmax        19,22,24                      19,22,24
Nref            6267                          6097
Tmin,Tmax       0.956,0.966                   0.356,0.754
Tmin'           0.956

Correction method= # Reported T Limits: Tmin=0.356 Tmax=0.754
AbsCorr = MULTI-SCAN

Data completeness= 0.973          Theta(max)= 68.236

R(reflections)= 0.1476( 2568)                      wR2(reflections)=
                                                   0.3522( 6097)
S = 1.037                 Npar= 145
```

The following ALERTS were generated. Each ALERT has the format
test-name_ALERT_alert-type_alert-level.
Click on the hyperlinks for more details of the test.

Alert level C

```
RINTA01_ALERT_3_C  The value of Rint is greater than 0.12
            Rint given   0.178
PLAT020_ALERT_3_C The Value of Rint is Greater Than 0.12 .........      0.178 Report
PLAT026_ALERT_3_C Ratio Observed / Unique Reflections (too) Low ..        42% Check
PLAT029_ALERT_3_C _diffrn_measured_fraction_theta_full value Low .      0.975 Why?
PLAT082_ALERT_2_C High R1 Value ..................................       0.15 Report
PLAT084_ALERT_3_C High wR2 Value (i.e. > 0.25) ...................       0.35 Report
PLAT213_ALERT_2_C Atom C10             has ADP max/min Ratio .....        3.3 prolat
PLAT234_ALERT_4_C Large Hirshfeld Difference N1       --C11      .       0.18 Ang.
PLAT234_ALERT_4_C Large Hirshfeld Difference C1       --C2       .       0.25 Ang.
PLAT234_ALERT_4_C Large Hirshfeld Difference C2       --C3       .       0.21 Ang.
PLAT234_ALERT_4_C Large Hirshfeld Difference C4       --C5       .       0.18 Ang.
PLAT234_ALERT_4_C Large Hirshfeld Difference C12      --C13      .       0.19 Ang.
PLAT241_ALERT_2_C High   'MainMol' Ueq as Compared to Neighbors of         O1 Check
PLAT241_ALERT_2_C High   'MainMol' Ueq as Compared to Neighbors of         C4 Check
PLAT241_ALERT_2_C High   'MainMol' Ueq as Compared to Neighbors of         C6 Check
PLAT242_ALERT_2_C Low    'MainMol' Ueq as Compared to Neighbors of        Zn1 Check
PLAT242_ALERT_2_C Low    'MainMol' Ueq as Compared to Neighbors of         C5 Check
PLAT250_ALERT_2_C Large U3/U1 Ratio for Average U(i,j) Tensor ....        3.3 Note
PLAT260_ALERT_2_C Large Average Ueq of Residue Including       Zn1      0.156 Check
PLAT341_ALERT_3_C Low Bond Precision on  C-C Bonds ...............    0.01133 Ang.
PLAT905_ALERT_3_C Negative K value in the Analysis of Variance ...    -62.578 Report
PLAT905_ALERT_3_C Negative K value in the Analysis of Variance ...     -3.356 Report
PLAT911_ALERT_3_C Missing FCF Refl Between Thmin & STh/L=    0.600        157 Report
PLAT976_ALERT_2_C Check Calcd Resid. Dens.  0.52Ang From C15A    .      -0.55 eA-3
```

Alert level G

```
PLAT002_ALERT_2_G Number of Distance or Angle Restraints on AtSite          9 Note
PLAT003_ALERT_2_G Number of Uiso or Uij Restrained non-H Atoms ...         21 Report
PLAT004_ALERT_5_G Polymeric Structure Found with Maximum Dimension          3 Info
PLAT083_ALERT_2_G SHELXL Second Parameter in WGHT  Unusually Large      10.00 Why ?
PLAT172_ALERT_4_G The CIF-Embedded .res File Contains DFIX Records          9 Report
PLAT173_ALERT_4_G The CIF-Embedded .res File Contains DANG Records          1 Report
PLAT174_ALERT_4_G The CIF-Embedded .res File Contains FLAT Records          1 Report
PLAT178_ALERT_4_G The CIF-Embedded .res File Contains SIMU Records          1 Report
PLAT186_ALERT_4_G The CIF-Embedded .res File Contains ISOR Records          1 Report
PLAT230_ALERT_2_G Hirshfeld Test Diff for    C6       --C7       .        6.5 s.u.
PLAT230_ALERT_2_G Hirshfeld Test Diff for    C6       --C7A      .        6.1 s.u.
PLAT232_ALERT_2_G Hirshfeld Test Diff (M-X)  Zn1      --O1       .        8.8 s.u.
PLAT232_ALERT_2_G Hirshfeld Test Diff (M-X)  Zn1      --N1       .       10.0 s.u.
PLAT300_ALERT_4_G Atom Site Occupancy of C11        Constrained at        0.5 Check
PLAT300_ALERT_4_G Atom Site Occupancy of C12        Constrained at        0.5 Check
PLAT300_ALERT_4_G Atom Site Occupancy of C15        Constrained at        0.5 Check
PLAT300_ALERT_4_G Atom Site Occupancy of C15A       Constrained at        0.5 Check
PLAT300_ALERT_4_G Atom Site Occupancy of H11        Constrained at        0.5 Check
PLAT300_ALERT_4_G Atom Site Occupancy of H12        Constrained at        0.5 Check
PLAT300_ALERT_4_G Atom Site Occupancy of H15        Constrained at        0.5 Check
PLAT300_ALERT_4_G Atom Site Occupancy of H15A       Constrained at        0.5 Check
PLAT301_ALERT_3_G Main Residue  Disorder .............(Resd  1  )         29% Note
```

```
PLAT367_ALERT_2_G Long?  C(sp?)-C(sp?) Bond  C5       - C6       .       1.53 Ang.
PLAT367_ALERT_2_G Long?  C(sp?)-C(sp?) Bond  C13      - C14      .       1.51 Ang.
PLAT606_ALERT_4_G Solvent Accessible VOID(S) in Structure ........          ! Info
PLAT773_ALERT_2_G Check long C-C Bond in CIF: C11      --C12             1.98 Ang.
PLAT779_ALERT_4_G Suspect or Irrelevant (Bond) Angle(s) in CIF ...      44.00 Deg.
              C12  -C11  -C12     1_555   1_555   8_655 ......     #   82 Check
PLAT860_ALERT_3_G Number of Least-Squares Restraints ............        181 Note
PLAT910_ALERT_3_G Missing # of FCF Reflection(s) Below Theta(Min).          1 Note
PLAT912_ALERT_4_G Missing # of FCF Reflections Above STh/L=  0.600         12 Note
PLAT913_ALERT_3_G Missing # of Very Strong Reflections in FCF ....          2 Note
PLAT933_ALERT_2_G Number of HKL-OMIT Records in Embedded .res File          8 Note
PLAT978_ALERT_2_G Number C-C Bonds with Positive Residual Density.          0 Info
```

```
   0 ALERT level A = Most likely a serious problem - resolve or explain
   0 ALERT level B = A potentially serious problem, consider carefully
  24 ALERT level C = Check. Ensure it is not caused by an omission or oversight
  33 ALERT level G = General information/check it is not something unexpected

   0 ALERT type 1 CIF construction/syntax error, inconsistent or missing data
  22 ALERT type 2 Indicator that the structure model may be wrong or deficient
  13 ALERT type 3 Indicator that the structure quality may be low
  21 ALERT type 4 Improvement, methodology, query or suggestion
   1 ALERT type 5 Informative message, check
```

It is advisable to attempt to resolve as many as possible of the alerts in all categories. Often the minor alerts point to easily fixed oversights, errors and omissions in your CIF or refinement strategy, so attention to these fine details can be worthwhile. In order to resolve some of the more serious problems it may be necessary to carry out additional measurements or structure refinements. However, the purpose of your study may justify the reported deviations and the more serious of these should normally be commented upon in the discussion or experimental section of a paper or in the "special_details" fields of the CIF. checkCIF was carefully designed to identify outliers and unusual parameters, but every test has its limitations and alerts that are not important in a particular case may appear. Conversely, the absence of alerts does not guarantee there are no aspects of the results needing attention. It is up to the individual to critically assess their own results and, if necessary, seek expert advice.

Publication of your CIF in IUCr journals

A basic structural check has been run on your CIF. These basic checks will be run on all CIFs submitted for publication in IUCr journals (*Acta Crystallographica*, *Journal of Applied Crystallography*, *Journal of Synchrotron Radiation*); however, if you intend to submit to *Acta Crystallographica Section C* or *E* or *IUCrData*, you should make sure that full publication checks are run on the final version of your CIF prior to submission.

Publication of your CIF in other journals

Please refer to the *Notes for Authors* of the relevant journal for any special instructions relating to CIF submission.

PLATON version of 18/05/2022; check.def file version of 17/05/2022

Datablock a - ellipsoid plot

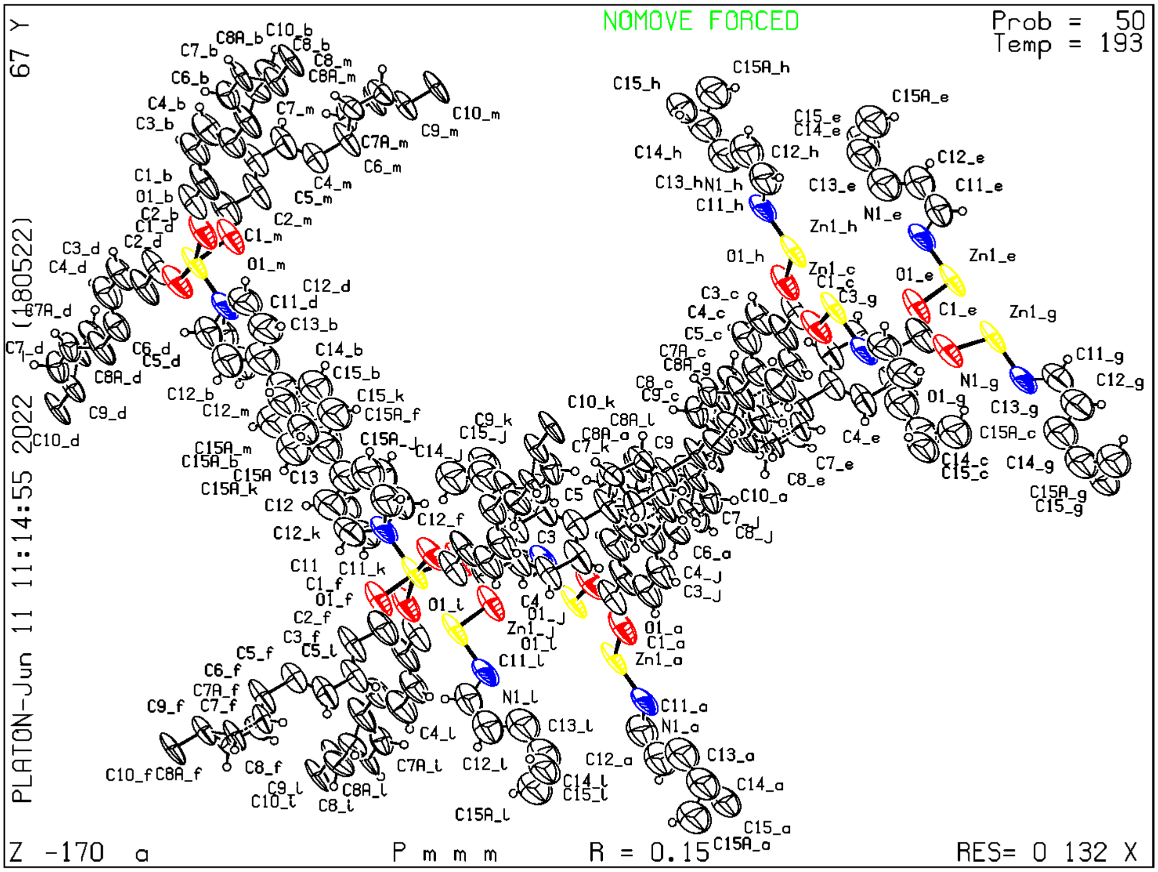

Supplement: Supplementary file 4 — Supplementary Data 1 [file 41467_2023_40594_MOESM4_ESM.zip › Supplementary Data 1/MeP5-MOF-1-G.pdf]
