# Supplementary material for: Pillararene incorporated metal–organic frameworks for supramolecular recognition and selective separation
Source: Nat Commun. 2023 Aug 15;14:4927. doi: 10.1038/s41467-023-40594-2 (PMC10427641; doi:10.1038/s41467-023-40594-2)

checkCIF/PLATON report

You have not supplied any structure factors. As a result the full set of tests cannot be run.

THIS REPORT IS FOR GUIDANCE ONLY. IF USED AS PART OF A REVIEW PROCEDURE FOR PUBLICATION, IT SHOULD NOT REPLACE THE EXPERTISE OF AN EXPERIENCED CRYSTALLOGRAPHIC REFEREE.

No syntax errors found. CIF dictionary Interpreting this report

Datablock: a

```
Bond precision:   C-C = 0.0047 A          Wavelength=0.71073

Cell:               a=16.449(3)      b=18.309(3)      c=20.203(4)
                    alpha=90         beta=90          gamma=90
Temperature:        193 K

                 Calculated                     Reported
Volume           6084.4(19)                     6084.6(17)
Space group      P m m m                        P m m m
Hall group       -P 2 2                         -P 2 2
Moiety formula   C35 H22 N O4 Zn [+ solvent] C70 H44 N2 O8 Zn2
Sum formula      C35 H22 N O4 Zn [+ solvent] C70 H44 N2 O8 Zn2
Mr               585.93                         1171.81
Dx,g cm-3        0.320                          0.320
Z                2                              1
Mu (mm-1)        0.211                          0.211
F000             602.0                          602.0
F000'            602.80
h,k,lmax         20,22,25                       20,22,25
Nref             6893                           6854
Tmin,Tmax        0.973,0.979                    0.563,0.745
Tmin'            0.973

Correction method= # Reported T Limits: Tmin=0.563 Tmax=0.745
AbsCorr = MULTI-SCAN

Data completeness= 0.994           Theta(max)= 26.427

R(reflections)= 0.0688( 4381)                       wR2(reflections)=
                                                    0.1911( 6854)
S = 1.012                  Npar= 145
```

The following ALERTS were generated. Each ALERT has the format
test-name_ALERT_alert-type_alert-level.
Click on the hyperlinks for more details of the test.

Alert level C

```
PLAT230_ALERT_2_C Hirshfeld Test Diff for    O1       --C1       .        5.5 s.u.
PLAT241_ALERT_2_C High   'MainMol' Ueq as Compared to Neighbors of         O1 Check
PLAT242_ALERT_2_C Low    'MainMol' Ueq as Compared to Neighbors of        Zn1 Check
PLAT242_ALERT_2_C Low    'MainMol' Ueq as Compared to Neighbors of        C13 Check
```

Alert level G

```
PLAT002_ALERT_2_G Number of Distance or Angle Restraints on AtSite          7 Note
PLAT003_ALERT_2_G Number of Uiso or Uij Restrained non-H Atoms ...          7 Report
PLAT004_ALERT_5_G Polymeric Structure Found with Maximum Dimension          3 Info
PLAT042_ALERT_1_G Calc. and Reported MoietyFormula Strings  Differ     Please Check
PLAT045_ALERT_1_G Calculated and Reported Z Differ by a Factor ...          2 Check
PLAT072_ALERT_2_G SHELXL First  Parameter in WGHT  Unusually Large       0.10 Report
PLAT152_ALERT_1_G The Supplied and Calc. Volume s.u. Differ by ...          2 Units
PLAT172_ALERT_4_G The CIF-Embedded .res File Contains DFIX Records          7 Report
PLAT173_ALERT_4_G The CIF-Embedded .res File Contains DANG Records          1 Report
PLAT174_ALERT_4_G The CIF-Embedded .res File Contains FLAT Records          1 Report
PLAT178_ALERT_4_G The CIF-Embedded .res File Contains SIMU Records          1 Report
PLAT188_ALERT_3_G A Non-default SIMU Restraint Value has been used     0.0100 Report
PLAT232_ALERT_2_G Hirshfeld Test Diff (M-X)  Zn1      --O1       .        9.3 s.u.
PLAT300_ALERT_4_G Atom Site Occupancy of C11        Constrained at        0.5 Check
PLAT300_ALERT_4_G Atom Site Occupancy of C12        Constrained at        0.5 Check
PLAT300_ALERT_4_G Atom Site Occupancy of C15        Constrained at        0.5 Check
PLAT300_ALERT_4_G Atom Site Occupancy of C15A       Constrained at        0.5 Check
PLAT300_ALERT_4_G Atom Site Occupancy of H11        Constrained at        0.5 Check
PLAT300_ALERT_4_G Atom Site Occupancy of H12        Constrained at        0.5 Check
PLAT300_ALERT_4_G Atom Site Occupancy of H15        Constrained at        0.5 Check
PLAT300_ALERT_4_G Atom Site Occupancy of H15A       Constrained at        0.5 Check
PLAT301_ALERT_3_G Main Residue  Disorder ..............(Resd  1  )        29% Note
PLAT606_ALERT_4_G Solvent Accessible VOID(S) in Structure ........          ! Info
PLAT860_ALERT_3_G Number of Least-Squares Restraints ............          53 Note
PLAT933_ALERT_2_G Number of HKL-OMIT Records in Embedded .res File          5 Note
PLAT941_ALERT_3_G Average HKL Measurement Multiplicity ...........        3.5 Low
```

0 **ALERT level A** = Most likely a serious problem - resolve or explain
0 **ALERT level B** = A potentially serious problem, consider carefully
4 **ALERT level C** = Check. Ensure it is not caused by an omission or oversight
26 **ALERT level G** = General information/check it is not something unexpected

3 ALERT type 1 CIF construction/syntax error, inconsistent or missing data
9 ALERT type 2 Indicator that the structure model may be wrong or deficient
4 ALERT type 3 Indicator that the structure quality may be low
13 ALERT type 4 Improvement, methodology, query or suggestion
1 ALERT type 5 Informative message, check

It is advisable to attempt to resolve as many as possible of the alerts in all categories. Often the minor alerts point to easily fixed oversights, errors and omissions in your CIF or refinement strategy, so attention to these fine details can be worthwhile. In order to resolve some of the more serious problems it may be necessary to carry out additional measurements or structure refinements. However, the purpose of your study may justify the reported deviations and the more serious of these should normally be commented upon in the discussion or experimental section of a paper or in the "special_details" fields of the CIF. checkCIF was carefully designed to identify outliers and unusual parameters, but every test has its limitations and alerts that are not important in a particular case may appear. Conversely, the absence of alerts does not guarantee there are no aspects of the results needing attention. It is up to the individual to critically assess their own results and, if necessary, seek expert advice.

Publication of your CIF in IUCr journals

A basic structural check has been run on your CIF. These basic checks will be run on all CIFs submitted for publication in IUCr journals (*Acta Crystallographica*, *Journal of Applied Crystallography*, *Journal of Synchrotron Radiation*); however, if you intend to submit to *Acta Crystallographica Section C* or *E* or *IUCrData*, you should make sure that full publication checks are run on the final version of your CIF prior to submission.

Publication of your CIF in other journals

Please refer to the *Notes for Authors* of the relevant journal for any special instructions relating to CIF submission.

PLATON version of 28/11/2022; check.def file version of 28/11/2022

Datablock a - ellipsoid plot

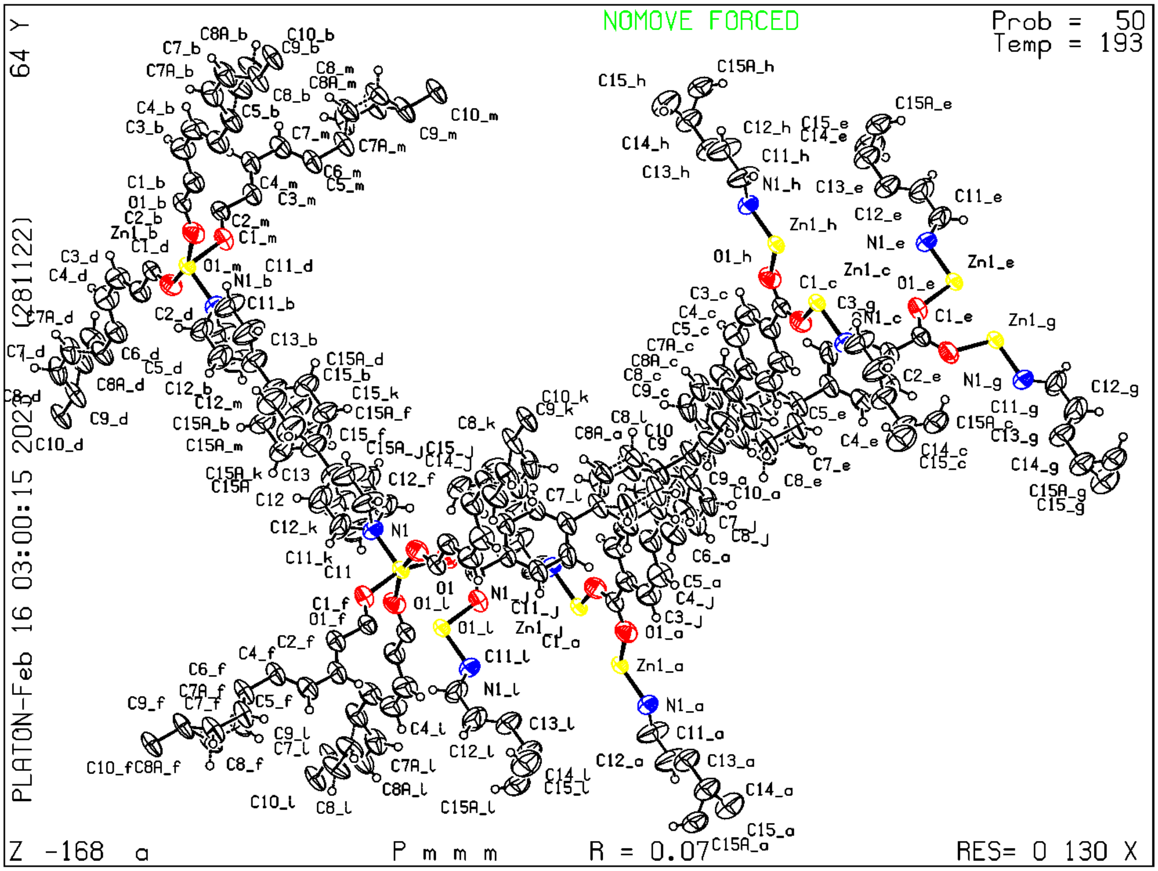

Supplement: Supplementary file 4 — Supplementary Data 1 [file 41467_2023_40594_MOESM4_ESM.zip › Supplementary Data 1/MeP5-MOF-1.pdf]
