# Supplementary material for: Pillararene incorporated metal–organic frameworks for supramolecular recognition and selective separation
Source: Nat Commun. 2023 Aug 15;14:4927. doi: 10.1038/s41467-023-40594-2 (PMC10427641; doi:10.1038/s41467-023-40594-2)

checkCIF/PLATON report

Structure factors have been supplied for datablock(s) 221031_wqangz_267677_0m

THIS REPORT IS FOR GUIDANCE ONLY. IF USED AS PART OF A REVIEW PROCEDURE FOR PUBLICATION, IT SHOULD NOT REPLACE THE EXPERTISE OF AN EXPERIENCED CRYSTALLOGRAPHIC REFEREE.

No syntax errors found. CIF dictionary Interpreting this report

Datablock: 221031_wqangz_267677_0m

Bond precision: C-C = 0.0098 A Wavelength=1.34139

Cell:	a=17.0634(12)	b=19.7634(14)	c=26.7805(19)
	alpha=85.077(3)	beta=72.719(3)	gamma=89.875(4)
Temperature:	193 K		

	Calculated	Reported
Volume	8589.1(11)	8589.1(11)
Space group	P -1	P -1
Hall group	-P 1	-P 1
Moiety formula	C119 H92 N2 O16 Zn2 [+ solvent]	C119 H92 N2 O16 Zn2
Sum formula	C119 H92 N2 O16 Zn2 [+ solvent]	C119 H92 N2 O16 Zn2
Mr	1936.73	1936.68
Dx,g cm-3	0.749	0.749
Z	2	2
Mu (mm-1)	0.432	0.432
F000	2016.0	2016.0
F000'	2009.32	
h,k,lmax	22,25,34	22,25,34
Nref	39348	38737
Tmin,Tmax	0.945,0.958	0.645,0.752
Tmin'	0.945	

Correction method= # Reported T Limits: Tmin=0.645 Tmax=0.752
AbsCorr = MULTI-SCAN

Data completeness= 0.984 Theta(max)= 60.542

R(reflections)= 0.1181(29728)   wR2(reflections)= 0.3200(38737)

S = 1.128   Npar= 1211

The following ALERTS were generated. Each ALERT has the format
test-name_ALERT_alert-type_alert-level.
Click on the hyperlinks for more details of the test.

Alert level B

```
PLAT220_ALERT_2_B NonSolvent   Resd 1   C   Ueq(max)/Ueq(min) Range        7.2 Ratio
```

Alert level C

```
PLAT082_ALERT_2_C High R1 Value ................................       0.12 Report
PLAT084_ALERT_3_C High wR2 Value (i.e. > 0.25) ..................       0.32 Report
PLAT213_ALERT_2_C Atom C58             has ADP max/min Ratio .....        3.1 prolat
PLAT220_ALERT_2_C NonSolvent   Resd 1   O   Ueq(max)/Ueq(min) Range        5.3 Ratio
PLAT222_ALERT_3_C NonSolvent Resd 1   H   Uiso(max)/Uiso(min) Range        8.1 Ratio
PLAT241_ALERT_2_C High   'MainMol' Ueq as Compared to Neighbors of         O7 Check
PLAT241_ALERT_2_C High   'MainMol' Ueq as Compared to Neighbors of        C56 Check
PLAT241_ALERT_2_C High   'MainMol' Ueq as Compared to Neighbors of        C58 Check
PLAT241_ALERT_2_C High   'MainMol' Ueq as Compared to Neighbors of        C59 Check
PLAT241_ALERT_2_C High   'MainMol' Ueq as Compared to Neighbors of        C62 Check
PLAT241_ALERT_2_C High   'MainMol' Ueq as Compared to Neighbors of        C65 Check
PLAT242_ALERT_2_C Low    'MainMol' Ueq as Compared to Neighbors of        Zn2 Check
PLAT242_ALERT_2_C Low    'MainMol' Ueq as Compared to Neighbors of         N1 Check
PLAT242_ALERT_2_C Low    'MainMol' Ueq as Compared to Neighbors of        C57 Check
PLAT242_ALERT_2_C Low    'MainMol' Ueq as Compared to Neighbors of        C60 Check
PLAT242_ALERT_2_C Low    'MainMol' Ueq as Compared to Neighbors of        C63 Check
PLAT242_ALERT_2_C Low    'MainMol' Ueq as Compared to Neighbors of       C109 Check
PLAT242_ALERT_2_C Low    'MainMol' Ueq as Compared to Neighbors of       C112 Check
PLAT260_ALERT_2_C Large Average Ueq of Residue Including       Zn1      0.105 Check
PLAT341_ALERT_3_C Low Bond Precision on  C-C Bonds ...............    0.00977 Ang.
PLAT412_ALERT_2_C Short Intra XH3 .. XHn     H96       ..H97B     .       1.86 Ang.
                                                       x,y,z  =      1_555 Check
PLAT906_ALERT_3_C Large K Value in the Analysis of Variance ......      3.605 Check
PLAT906_ALERT_3_C Large K Value in the Analysis of Variance ......      2.099 Check
PLAT911_ALERT_3_C Missing FCF Refl Between Thmin & STh/L=    0.600         33 Report
PLAT918_ALERT_3_C Reflection(s) with I(obs) much Smaller I(calc) .         13 Check
PLAT972_ALERT_2_C Check Calcd Resid. Dens.  0.98Ang From C85             -1.64 eA-3
PLAT976_ALERT_2_C Check Calcd Resid. Dens.  0.66Ang From O11      .      -0.57 eA-3
PLAT977_ALERT_2_C Check Negative Difference Density on H22        .      -0.33 eA-3
PLAT977_ALERT_2_C Check Negative Difference Density on H30        .      -0.35 eA-3
PLAT977_ALERT_2_C Check Negative Difference Density on H49        .      -0.31 eA-3
PLAT977_ALERT_2_C Check Negative Difference Density on H53        .      -0.37 eA-3
PLAT977_ALERT_2_C Check Negative Difference Density on H55        .      -0.38 eA-3
PLAT977_ALERT_2_C Check Negative Difference Density on H96        .      -0.35 eA-3
```

Alert level G

```
ABSMU01_ALERT_1_G  Calculation of _exptl_absorpt_correction_mu
                not performed for this radiation type.
PLAT002_ALERT_2_G Number of Distance or Angle Restraints on AtSite         21 Note
PLAT003_ALERT_2_G Number of Uiso or Uij Restrained non-H Atoms ...         51 Report
```

```
PLAT004_ALERT_5_G Polymeric Structure Found with Maximum Dimension          3 Info
PLAT072_ALERT_2_G SHELXL First  Parameter in WGHT  Unusually Large       0.12 Report
PLAT083_ALERT_2_G SHELXL Second Parameter in WGHT  Unusually Large      20.00 Why ?
PLAT172_ALERT_4_G The CIF-Embedded .res File Contains DFIX Records          4 Report
PLAT176_ALERT_4_G The CIF-Embedded .res File Contains SADI Records          1 Report
PLAT178_ALERT_4_G The CIF-Embedded .res File Contains SIMU Records          1 Report
PLAT335_ALERT_2_G Check Large C6 Ring C-C Range C109     -C114           0.16 Ang.
PLAT398_ALERT_2_G Deviating  C-O-C   Angle From 120 for O10      .      134.8 Degree
PLAT398_ALERT_2_G Deviating  C-O-C   Angle From 120 for O14      .      108.6 Degree
PLAT432_ALERT_2_G Short Inter X...Y Contact  C37      ..C117     .       3.14 Ang.
                                                 -x,1-y,1-z  =      2_566 Check
PLAT606_ALERT_4_G Solvent Accessible VOID(S) in Structure ........          ! Info
PLAT794_ALERT_5_G Tentative Bond Valency for Zn1       (II)      .       2.02 Info
PLAT794_ALERT_5_G Tentative Bond Valency for Zn2       (II)      .       2.09 Info
PLAT860_ALERT_3_G Number of Least-Squares Restraints .............        368 Note
PLAT912_ALERT_4_G Missing # of FCF Reflections Above STh/L=  0.600        579 Note
PLAT913_ALERT_3_G Missing # of Very Strong Reflections in FCF ....          2 Note
PLAT933_ALERT_2_G Number of HKL-OMIT Records in Embedded .res File         14 Note
PLAT978_ALERT_2_G Number C-C Bonds with Positive Residual Density.          0 Info
PLAT992_ALERT_5_G Repd & Actual _reflns_number_gt Values Differ by          3 Check
```

```
  0 ALERT level A = Most likely a serious problem - resolve or explain
  1 ALERT level B = A potentially serious problem, consider carefully
 33 ALERT level C = Check. Ensure it is not caused by an omission or oversight
 22 ALERT level G = General information/check it is not something unexpected

  1 ALERT type 1 CIF construction/syntax error, inconsistent or missing data
 37 ALERT type 2 Indicator that the structure model may be wrong or deficient
  9 ALERT type 3 Indicator that the structure quality may be low
  5 ALERT type 4 Improvement, methodology, query or suggestion
  4 ALERT type 5 Informative message, check
```

It is advisable to attempt to resolve as many as possible of the alerts in all categories. Often the minor alerts point to easily fixed oversights, errors and omissions in your CIF or refinement strategy, so attention to these fine details can be worthwhile. In order to resolve some of the more serious problems it may be necessary to carry out additional measurements or structure refinements. However, the purpose of your study may justify the reported deviations and the more serious of these should normally be commented upon in the discussion or experimental section of a paper or in the "special_details" fields of the CIF. checkCIF was carefully designed to identify outliers and unusual parameters, but every test has its limitations and alerts that are not important in a particular case may appear. Conversely, the absence of alerts does not guarantee there are no aspects of the results needing attention. It is up to the individual to critically assess their own results and, if necessary, seek expert advice.

Publication of your CIF in IUCr journals

A basic structural check has been run on your CIF. These basic checks will be run on all CIFs submitted for publication in IUCr journals (*Acta Crystallographica*, *Journal of Applied Crystallography*, *Journal of Synchrotron Radiation*); however, if you intend to submit to *Acta Crystallographica Section C* or *E* or *IUCrData*, you should make sure that full publication checks are run on the final version of your CIF prior to submission.

Publication of your CIF in other journals

Please refer to the *Notes for Authors* of the relevant journal for any special instructions relating to CIF submission.

PLATON version of 12/09/2022; check.def file version of 09/08/2022

Datablock 221031_wqangz_267677_0m - ellipsoid plot

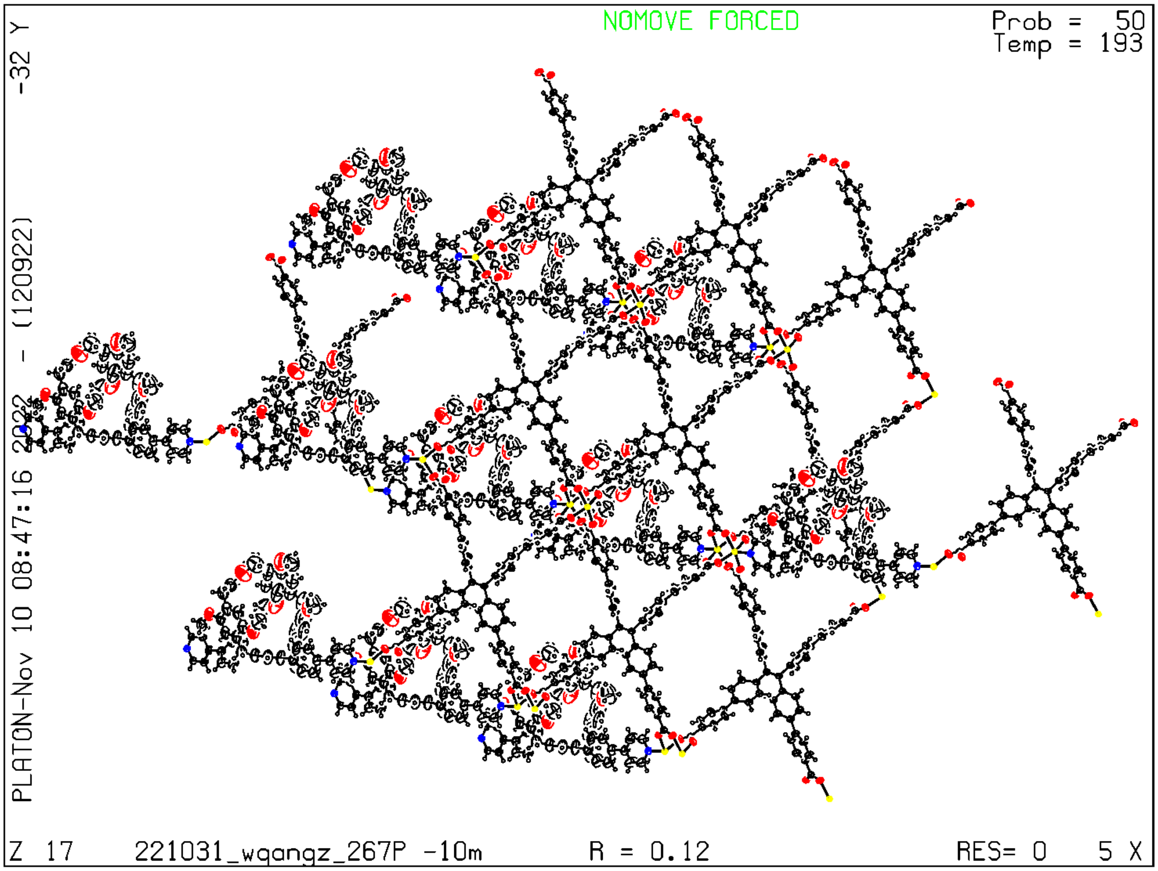

Supplement: Supplementary file 4 — Supplementary Data 1 [file 41467_2023_40594_MOESM4_ESM.zip › Supplementary Data 1/MeP5-MOF-2.pdf]
