# Supplementary material for: Pillararene incorporated metal–organic frameworks for supramolecular recognition and selective separation
Source: Nat Commun. 2023 Aug 15;14:4927. doi: 10.1038/s41467-023-40594-2 (PMC10427641; doi:10.1038/s41467-023-40594-2)

checkCIF/PLATON report

Structure factors have been supplied for datablock(s) a

THIS REPORT IS FOR GUIDANCE ONLY. IF USED AS PART OF A REVIEW PROCEDURE FOR PUBLICATION, IT SHOULD NOT REPLACE THE EXPERTISE OF AN EXPERIENCED CRYSTALLOGRAPHIC REFEREE.

No syntax errors found. CIF dictionary Interpreting this report

Datablock: a

```
Bond precision:   C-C = 0.0088 A          Wavelength=1.34139

Cell:             a=13.1993(9)     b=11.4758(7)       c=26.8418(19)
                  alpha=90         beta=95.824(4)     gamma=90
Temperature:      193 K

                 Calculated                    Reported
Volume           4044.8(5)                     4044.8(5)
Space group      P 2/m                         P 1 2/m 1
Hall group       -P 2y                         -P 2y
Moiety formula   C29 H18 N O4 Zn [+ solvent]   C58 H36 N2 O8 Zn2
Sum formula      C29 H18 N O4 Zn [+ solvent]   C58 H36 N2 O8 Zn2
Mr               509.83                        1019.63
Dx,g cm-3        0.419                         0.419
Z                2                             1
Mu (mm-1)        0.333                         0.333
F000             522.0                         522.0
F000'            517.57
h,k,lmax         15,13,32                      15,13,32
Nref             7797                          7649
Tmin,Tmax        0.958,0.967                   0.443,0.752
Tmin'            0.958

Correction method= # Reported T Limits: Tmin=0.443 Tmax=0.752
AbsCorr = MULTI-SCAN

Data completeness= 0.981           Theta(max)= 53.902

R(reflections)= 0.1498( 4824)                         wR2(reflections)=
                                                      0.3435( 7649)
S = 1.143                    Npar= 339
```

The following ALERTS were generated. Each ALERT has the format
test-name_ALERT_alert-type_alert-level.
Click on the hyperlinks for more details of the test.

Alert level B

```
PLAT971_ALERT_2_B Check Calcd Resid. Dens.  0.91Ang From N2           2.60 eA-3
```

Alert level C

```
PLAT082_ALERT_2_C High R1 Value ..................................       0.15 Report
PLAT084_ALERT_3_C High wR2 Value (i.e. > 0.25) ...................       0.34 Report
PLAT241_ALERT_2_C High   'MainMol' Ueq as Compared to Neighbors of         O1 Check
PLAT242_ALERT_2_C Low    'MainMol' Ueq as Compared to Neighbors of        Zn1 Check
PLAT242_ALERT_2_C Low    'MainMol' Ueq as Compared to Neighbors of         C9 Check
PLAT334_ALERT_2_C Small <C-C> Benzene Dist.   C7       -C18      .       1.37 Ang.
PLAT341_ALERT_3_C Low Bond Precision on  C-C Bonds ...............    0.00875 Ang.
PLAT369_ALERT_2_C Long   C(sp2)-C(sp2) Bond  C7       - C9       .       1.53 Ang.
PLAT906_ALERT_3_C Large K Value in the Analysis of Variance ......      2.515 Check
PLAT911_ALERT_3_C Missing FCF Refl Between Thmin & STh/L=    0.600        146 Report
PLAT971_ALERT_2_C Check Calcd Resid. Dens.  1.05Ang From Zn1             2.40 eA-3
```

Alert level G

```
ABSMU01_ALERT_1_G  Calculation of _exptl_absorpt_correction_mu
                not performed for this radiation type.
PLAT002_ALERT_2_G Number of Distance or Angle Restraints on AtSite         18 Note
PLAT003_ALERT_2_G Number of Uiso or Uij Restrained non-H Atoms ...         40 Report
PLAT004_ALERT_5_G Polymeric Structure Found with Maximum Dimension          3 Info
PLAT042_ALERT_1_G Calc. and Reported MoietyFormula Strings  Differ     Please Check
PLAT045_ALERT_1_G Calculated and Reported Z Differ by a Factor ...          2 Check
PLAT083_ALERT_2_G SHELXL Second Parameter in WGHT  Unusually Large      15.00 Why ?
PLAT172_ALERT_4_G The CIF-Embedded .res File Contains DFIX Records          1 Report
PLAT174_ALERT_4_G The CIF-Embedded .res File Contains FLAT Records          1 Report
PLAT176_ALERT_4_G The CIF-Embedded .res File Contains SADI Records          5 Report
PLAT178_ALERT_4_G The CIF-Embedded .res File Contains SIMU Records          2 Report
PLAT186_ALERT_4_G The CIF-Embedded .res File Contains ISOR Records          1 Report
PLAT300_ALERT_4_G Atom Site Occupancy of C16        Constrained at        0.5 Check
PLAT300_ALERT_4_G Atom Site Occupancy of C17        Constrained at        0.5 Check
PLAT300_ALERT_4_G Atom Site Occupancy of C18        Constrained at        0.5 Check
PLAT300_ALERT_4_G Atom Site Occupancy of C19        Constrained at        0.5 Check
PLAT300_ALERT_4_G Atom Site Occupancy of C20        Constrained at        0.5 Check
PLAT300_ALERT_4_G Atom Site Occupancy of C21        Constrained at        0.5 Check
PLAT300_ALERT_4_G Atom Site Occupancy of C22        Constrained at        0.5 Check
PLAT300_ALERT_4_G Atom Site Occupancy of C23        Constrained at        0.5 Check
PLAT300_ALERT_4_G Atom Site Occupancy of N1         Constrained at       0.25 Check
PLAT300_ALERT_4_G Atom Site Occupancy of N2         Constrained at       0.25 Check
PLAT300_ALERT_4_G Atom Site Occupancy of C1         Constrained at       0.25 Check
PLAT300_ALERT_4_G Atom Site Occupancy of C2         Constrained at       0.25 Check
PLAT300_ALERT_4_G Atom Site Occupancy of C3         Constrained at       0.25 Check
PLAT300_ALERT_4_G Atom Site Occupancy of C4         Constrained at       0.25 Check
PLAT300_ALERT_4_G Atom Site Occupancy of C5         Constrained at       0.25 Check
PLAT300_ALERT_4_G Atom Site Occupancy of C10        Constrained at       0.25 Check
PLAT300_ALERT_4_G Atom Site Occupancy of C11        Constrained at       0.25 Check
PLAT300_ALERT_4_G Atom Site Occupancy of C12        Constrained at       0.25 Check
```

```
PLAT300_ALERT_4_G Atom Site Occupancy of C13        Constrained at       0.25 Check
PLAT300_ALERT_4_G Atom Site Occupancy of C14        Constrained at       0.25 Check
PLAT300_ALERT_4_G Atom Site Occupancy of C15        Constrained at       0.25 Check
PLAT300_ALERT_4_G Atom Site Occupancy of C24        Constrained at       0.25 Check
PLAT300_ALERT_4_G Atom Site Occupancy of C25        Constrained at       0.25 Check
PLAT300_ALERT_4_G Atom Site Occupancy of C26        Constrained at       0.25 Check
PLAT300_ALERT_4_G Atom Site Occupancy of C27        Constrained at       0.25 Check
PLAT300_ALERT_4_G Atom Site Occupancy of C28        Constrained at       0.25 Check
PLAT300_ALERT_4_G Atom Site Occupancy of C29        Constrained at       0.25 Check
PLAT300_ALERT_4_G Atom Site Occupancy of C30        Constrained at       0.25 Check
PLAT300_ALERT_4_G Atom Site Occupancy of C31        Constrained at       0.25 Check
PLAT300_ALERT_4_G Atom Site Occupancy of C32        Constrained at       0.25 Check
PLAT300_ALERT_4_G Atom Site Occupancy of C33        Constrained at       0.25 Check
PLAT300_ALERT_4_G Atom Site Occupancy of C34        Constrained at       0.25 Check
PLAT300_ALERT_4_G Atom Site Occupancy of C35        Constrained at       0.25 Check
PLAT300_ALERT_4_G Atom Site Occupancy of C36        Constrained at       0.25 Check
PLAT300_ALERT_4_G Atom Site Occupancy of C37        Constrained at       0.25 Check
PLAT300_ALERT_4_G Atom Site Occupancy of C38        Constrained at       0.25 Check
PLAT300_ALERT_4_G Atom Site Occupancy of C39        Constrained at       0.25 Check
PLAT300_ALERT_4_G Atom Site Occupancy of C40        Constrained at       0.25 Check
PLAT300_ALERT_4_G Atom Site Occupancy of H16        Constrained at        0.5 Check
PLAT300_ALERT_4_G Atom Site Occupancy of H17        Constrained at        0.5 Check
PLAT300_ALERT_4_G Atom Site Occupancy of H18        Constrained at        0.5 Check
PLAT300_ALERT_4_G Atom Site Occupancy of H19        Constrained at        0.5 Check
PLAT300_ALERT_4_G Atom Site Occupancy of H20        Constrained at        0.5 Check
PLAT300_ALERT_4_G Atom Site Occupancy of H21        Constrained at        0.5 Check
PLAT300_ALERT_4_G Atom Site Occupancy of H22        Constrained at        0.5 Check
PLAT300_ALERT_4_G Atom Site Occupancy of H23        Constrained at        0.5 Check
PLAT300_ALERT_4_G Atom Site Occupancy of H1         Constrained at       0.25 Check
PLAT300_ALERT_4_G Atom Site Occupancy of H2         Constrained at       0.25 Check
PLAT300_ALERT_4_G Atom Site Occupancy of H4         Constrained at       0.25 Check
PLAT300_ALERT_4_G Atom Site Occupancy of H5         Constrained at       0.25 Check
PLAT300_ALERT_4_G Atom Site Occupancy of H10        Constrained at       0.25 Check
PLAT300_ALERT_4_G Atom Site Occupancy of H11        Constrained at       0.25 Check
PLAT300_ALERT_4_G Atom Site Occupancy of H13        Constrained at       0.25 Check
PLAT300_ALERT_4_G Atom Site Occupancy of H14        Constrained at       0.25 Check
PLAT300_ALERT_4_G Atom Site Occupancy of H25        Constrained at       0.25 Check
PLAT300_ALERT_4_G Atom Site Occupancy of H26        Constrained at       0.25 Check
PLAT300_ALERT_4_G Atom Site Occupancy of H28        Constrained at       0.25 Check
PLAT300_ALERT_4_G Atom Site Occupancy of H29        Constrained at       0.25 Check
PLAT300_ALERT_4_G Atom Site Occupancy of H30        Constrained at       0.25 Check
PLAT300_ALERT_4_G Atom Site Occupancy of H32        Constrained at       0.25 Check
PLAT300_ALERT_4_G Atom Site Occupancy of H33        Constrained at       0.25 Check
PLAT300_ALERT_4_G Atom Site Occupancy of H35        Constrained at       0.25 Check
PLAT300_ALERT_4_G Atom Site Occupancy of H36        Constrained at       0.25 Check
PLAT300_ALERT_4_G Atom Site Occupancy of H37        Constrained at       0.25 Check
PLAT300_ALERT_4_G Atom Site Occupancy of H38        Constrained at       0.25 Check
PLAT300_ALERT_4_G Atom Site Occupancy of H40        Constrained at       0.25 Check
PLAT301_ALERT_3_G Main Residue  Disorder .............(Resd  1  )        64% Note
PLAT606_ALERT_4_G Solvent Accessible VOID(S) in Structure ........         ! Info
PLAT789_ALERT_4_G Atoms with Negative _atom_site_disorder_group  #        50 Check
PLAT811_ALERT_5_G No ADDSYM Analysis: Too Many Excluded Atoms ....         ! Info
PLAT860_ALERT_3_G Number of Least-Squares Restraints ............        514 Note
PLAT912_ALERT_4_G Missing # of FCF Reflections Above STh/L=  0.600         2 Note
PLAT913_ALERT_3_G Missing # of Very Strong Reflections in FCF ....         1 Note
PLAT933_ALERT_2_G Number of HKL-OMIT Records in Embedded .res File         1 Note
PLAT941_ALERT_3_G Average HKL Measurement Multiplicity ..........        2.9 Low
```

```
PLAT978_ALERT_2_G Number C-C Bonds with Positive Residual Density.          3 Info
```

```
   0 ALERT level A = Most likely a serious problem - resolve or explain
   1 ALERT level B = A potentially serious problem, consider carefully
  11 ALERT level C = Check. Ensure it is not caused by an omission or oversight
  88 ALERT level G = General information/check it is not something unexpected

   3 ALERT type 1 CIF construction/syntax error, inconsistent or missing data
  13 ALERT type 2 Indicator that the structure model may be wrong or deficient
   8 ALERT type 3 Indicator that the structure quality may be low
  74 ALERT type 4 Improvement, methodology, query or suggestion
   2 ALERT type 5 Informative message, check
```

It is advisable to attempt to resolve as many as possible of the alerts in all categories. Often the minor alerts point to easily fixed oversights, errors and omissions in your CIF or refinement strategy, so attention to these fine details can be worthwhile. In order to resolve some of the more serious problems it may be necessary to carry out additional measurements or structure refinements. However, the purpose of your study may justify the reported deviations and the more serious of these should normally be commented upon in the discussion or experimental section of a paper or in the "special_details" fields of the CIF. checkCIF was carefully designed to identify outliers and unusual parameters, but every test has its limitations and alerts that are not important in a particular case may appear. Conversely, the absence of alerts does not guarantee there are no aspects of the results needing attention. It is up to the individual to critically assess their own results and, if necessary, seek expert advice.

Publication of your CIF in IUCr journals

A basic structural check has been run on your CIF. These basic checks will be run on all CIFs submitted for publication in IUCr journals (*Acta Crystallographica*, *Journal of Applied Crystallography*, *Journal of Synchrotron Radiation*); however, if you intend to submit to *Acta Crystallographica Section C* or *E* or *IUCrData*, you should make sure that full publication checks are run on the final version of your CIF prior to submission.

Publication of your CIF in other journals

Please refer to the *Notes for Authors* of the relevant journal for any special instructions relating to CIF submission.

PLATON version of 12/09/2022; check.def file version of 09/08/2022

Datablock a - ellipsoid plot

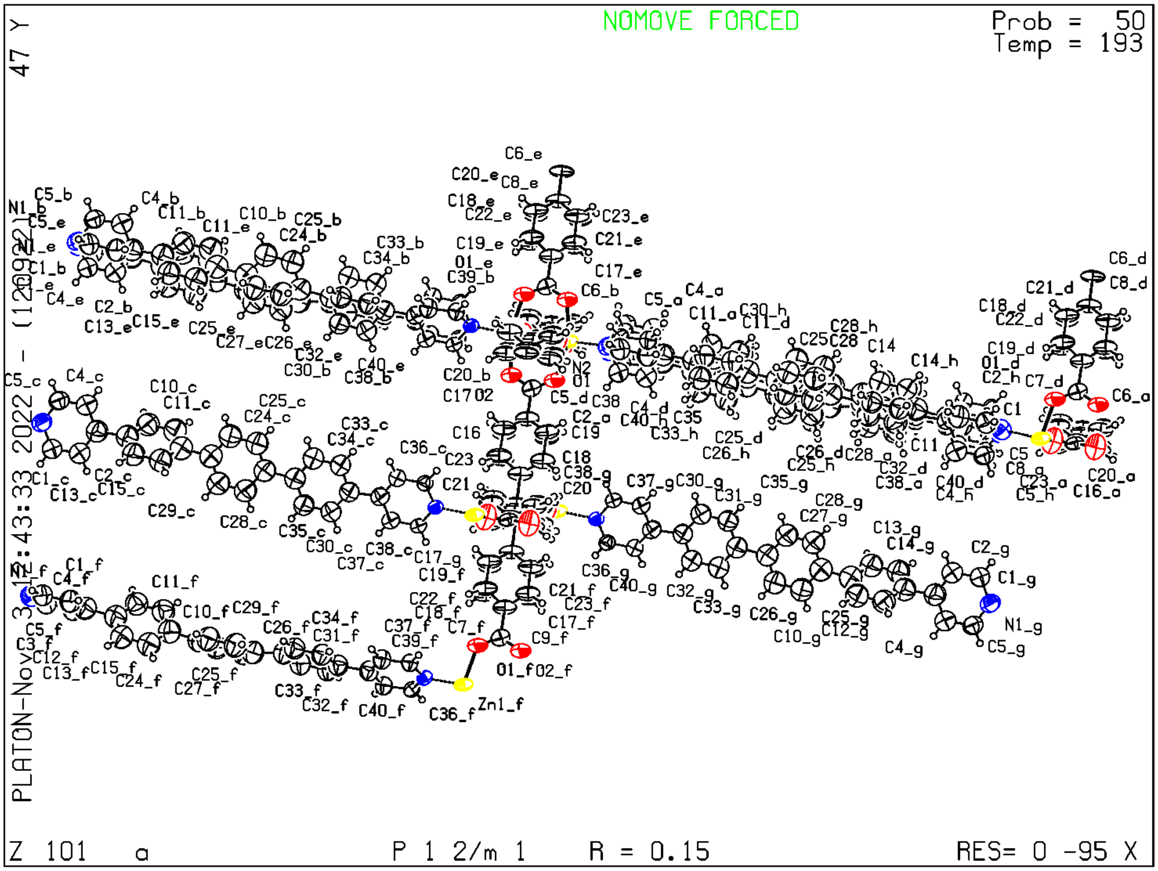

Supplement: Supplementary file 4 — Supplementary Data 1 [file 41467_2023_40594_MOESM4_ESM.zip › Supplementary Data 1/MeP5-MOF-4.pdf]
