# Supplementary material for: Pillararene incorporated metal–organic frameworks for supramolecular recognition and selective separation
Source: Nat Commun. 2023 Aug 15;14:4927. doi: 10.1038/s41467-023-40594-2 (PMC10427641; doi:10.1038/s41467-023-40594-2)

checkCIF/PLATON report

Structure factors have been supplied for datablock(s) a

THIS REPORT IS FOR GUIDANCE ONLY. IF USED AS PART OF A REVIEW PROCEDURE FOR PUBLICATION, IT SHOULD NOT REPLACE THE EXPERTISE OF AN EXPERIENCED CRYSTALLOGRAPHIC REFEREE.

No syntax errors found. CIF dictionary Interpreting this report

Datablock: a

```
Bond precision:   C-C = 0.0042 A          Wavelength=0.71073

Cell:             a=23.0941(8)     b=17.3444(6)        c=13.6476(4)
                  alpha=90         beta=106.331(1)     gamma=90
Temperature:      193 K

                 Calculated                   Reported
Volume           5246.0(3)                    5246.0(3)
Space group      P 21/c                       P 1 21/c 1
Hall group       -P 2ybc                      -P 2ybc
Moiety formula   C65 H61 N2 O8                C65 H61 N2 O8
Sum formula      C65 H61 N2 O8                C65 H61 N2 O8
Mr               998.16                       998.15
Dx,g cm-3        1.264                        1.264
Z                4                            4
Mu (mm-1)        0.083                        0.083
F000             2116.0                       2116.0
F000'            2116.96
h,k,lmax         28,21,17                     28,21,17
Nref             10756                        10737
Tmin,Tmax        0.989,0.992                  0.688,0.745
Tmin'            0.989

Correction method= # Reported T Limits: Tmin=0.688 Tmax=0.745
AbsCorr = MULTI-SCAN

Data completeness= 0.998           Theta(max)= 26.392

R(reflections)= 0.0696( 6816)                              wR2(reflections)=
                                                           0.1541( 10737)
S = 1.071                   Npar= 764
```

The following ALERTS were generated. Each ALERT has the format
test-name_ALERT_alert-type_alert-level.
Click on the hyperlinks for more details of the test.

Alert level C

```
PLAT220_ALERT_2_C NonSolvent   Resd 1  C   Ueq(max)/Ueq(min) Range        3.7 Ratio
PLAT222_ALERT_3_C NonSolvent Resd 1  H   Uiso(max)/Uiso(min) Range        4.2 Ratio
PLAT241_ALERT_2_C High   'MainMol' Ueq as Compared to Neighbors of        C33 Check
PLAT241_ALERT_2_C High   'MainMol' Ueq as Compared to Neighbors of        C42 Check
PLAT340_ALERT_3_C Low Bond Precision on  C-C Bonds ...............    0.00422 Ang.
PLAT420_ALERT_2_C D-H Bond Without Acceptor  N2       --H2A      .     Please Check
PLAT906_ALERT_3_C Large K Value in the Analysis of Variance ......     12.354 Check
PLAT906_ALERT_3_C Large K Value in the Analysis of Variance ......      2.696 Check
PLAT911_ALERT_3_C Missing FCF Refl Between Thmin & STh/L=    0.600          6 Report
PLAT977_ALERT_2_C Check Negative Difference Density on H2A       .      -0.35 eA-3
```

Alert level G

```
PLAT002_ALERT_2_G Number of Distance or Angle Restraints on AtSite         18 Note
PLAT003_ALERT_2_G Number of Uiso or Uij Restrained non-H Atoms ...          6 Report
PLAT007_ALERT_5_G Number of Unrefined Donor-H Atoms ..............          1 Report
PLAT083_ALERT_2_G SHELXL Second Parameter in WGHT  Unusually Large       5.30 Why ?
PLAT176_ALERT_4_G The CIF-Embedded .res File Contains SADI Records          6 Report
PLAT178_ALERT_4_G The CIF-Embedded .res File Contains SIMU Records          3 Report
PLAT186_ALERT_4_G The CIF-Embedded .res File Contains ISOR Records          1 Report
PLAT301_ALERT_3_G Main Residue  Disorder ..............(Resd  1  )        11% Note
PLAT412_ALERT_2_G Short Intra XH3 .. XHn     H32C     ..H50D     .       2.14 Ang.
                                                     x,y,z  =      1_555 Check
PLAT413_ALERT_2_G Short Inter XH3 .. XHn     H33B     ..H41E     .       2.12 Ang.
                                             x,1/2-y,-1/2+z  =     4_565 Check
PLAT415_ALERT_2_G Short Inter D-H..H-X       H2A      ..H49C     .       1.76 Ang.
                                                  x,y,-1+z  =      1_554 Check
PLAT415_ALERT_2_G Short Inter D-H..H-X       H2A      ..H49E     .       1.84 Ang.
                                                  x,y,-1+z  =      1_554 Check
PLAT860_ALERT_3_G Number of Least-Squares Restraints .............         36 Note
PLAT910_ALERT_3_G Missing # of FCF Reflection(s) Below Theta(Min).          2 Note
PLAT912_ALERT_4_G Missing # of FCF Reflections Above STh/L=  0.600         12 Note
PLAT913_ALERT_3_G Missing # of Very Strong Reflections in FCF ....          2 Note
PLAT933_ALERT_2_G Number of HKL-OMIT Records in Embedded .res File          1 Note
PLAT941_ALERT_3_G Average HKL Measurement Multiplicity ...........        4.1 Low
PLAT978_ALERT_2_G Number C-C Bonds with Positive Residual Density.          4 Info
```

0 **ALERT level A** = Most likely a serious problem - resolve or explain
0 **ALERT level B** = A potentially serious problem, consider carefully
10 **ALERT level C** = Check. Ensure it is not caused by an omission or oversight
19 **ALERT level G** = General information/check it is not something unexpected

0 ALERT type 1 CIF construction/syntax error, inconsistent or missing data
14 ALERT type 2 Indicator that the structure model may be wrong or deficient
10 ALERT type 3 Indicator that the structure quality may be low
4 ALERT type 4 Improvement, methodology, query or suggestion
1 ALERT type 5 Informative message, check

It is advisable to attempt to resolve as many as possible of the alerts in all categories. Often the minor alerts point to easily fixed oversights, errors and omissions in your CIF or refinement strategy, so attention to these fine details can be worthwhile. In order to resolve some of the more serious problems it may be necessary to carry out additional measurements or structure refinements. However, the purpose of your study may justify the reported deviations and the more serious of these should normally be commented upon in the discussion or experimental section of a paper or in the "special_details" fields of the CIF. checkCIF was carefully designed to identify outliers and unusual parameters, but every test has its limitations and alerts that are not important in a particular case may appear. Conversely, the absence of alerts does not guarantee there are no aspects of the results needing attention. It is up to the individual to critically assess their own results and, if necessary, seek expert advice.

Publication of your CIF in IUCr journals

A basic structural check has been run on your CIF. These basic checks will be run on all CIFs submitted for publication in IUCr journals (*Acta Crystallographica*, *Journal of Applied Crystallography*, *Journal of Synchrotron Radiation*); however, if you intend to submit to *Acta Crystallographica Section C* or *E* or *IUCrData*, you should make sure that full publication checks are run on the final version of your CIF prior to submission.

Publication of your CIF in other journals

Please refer to the *Notes for Authors* of the relevant journal for any special instructions relating to CIF submission.

PLATON version of 18/05/2022; check.def file version of 17/05/2022

Datablock a - ellipsoid plot

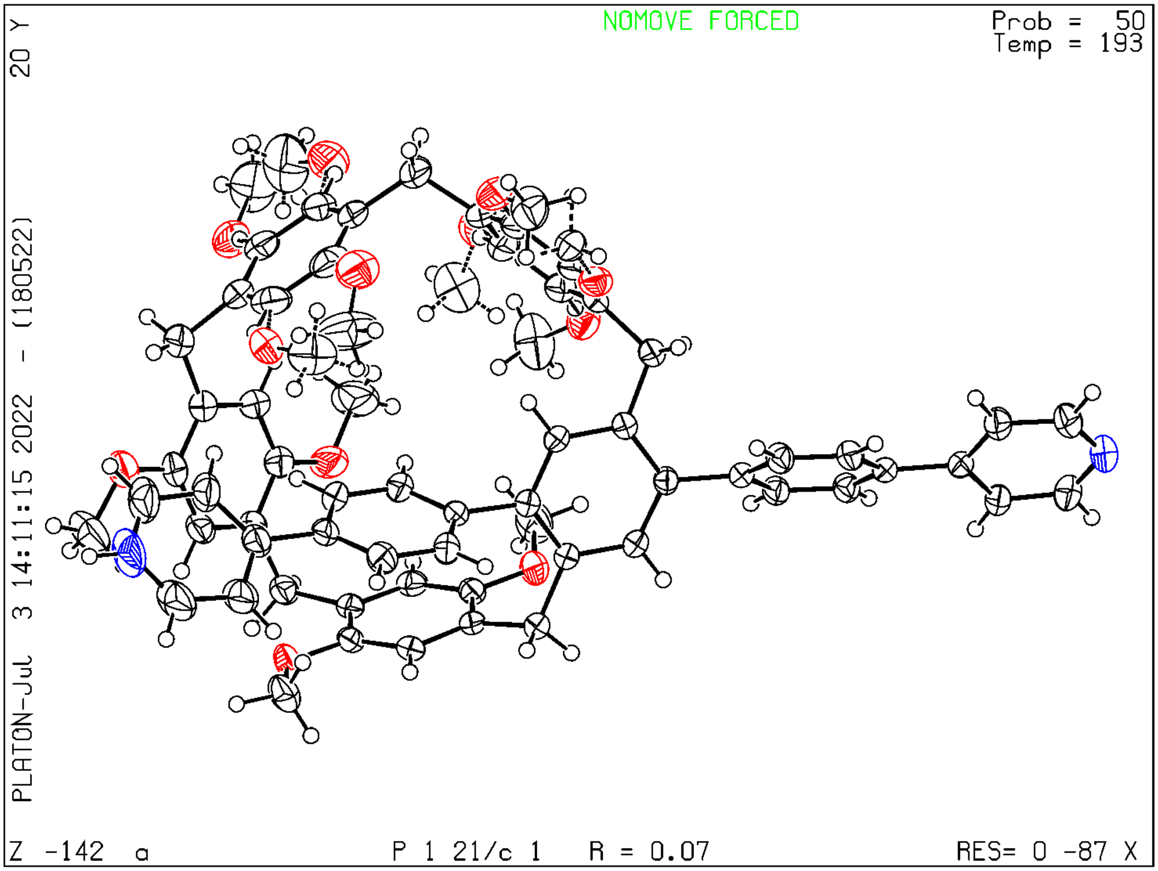

Supplement: Supplementary file 4 — Supplementary Data 1 [file 41467_2023_40594_MOESM4_ESM.zip › Supplementary Data 1/MeP5BPPy.pdf]
