# Supplementary material for: Pillararene incorporated metal–organic frameworks for supramolecular recognition and selective separation
Source: Nat Commun. 2023 Aug 15;14:4927. doi: 10.1038/s41467-023-40594-2 (PMC10427641; doi:10.1038/s41467-023-40594-2)

checkCIF/PLATON report

Structure factors have been supplied for datablock(s) a1

THIS REPORT IS FOR GUIDANCE ONLY. IF USED AS PART OF A REVIEW PROCEDURE FOR PUBLICATION, IT SHOULD NOT REPLACE THE EXPERTISE OF AN EXPERIENCED CRYSTALLOGRAPHIC REFEREE.

No syntax errors found. CIF dictionary Interpreting this report

Datablock: a1

```
Bond precision:   C-C = 0.0127 A          Wavelength=1.34139

Cell:              a=20.2233(14)      b=51.597(4)     c=16.3953(11)
                   alpha=90           beta=90         gamma=90
Temperature:       193 K

                 Calculated                        Reported
Volume           17108(2)                          17108(2)
Space group      P 21 21 2                         P 21 21 2
Hall group       P 2 2ab                           P 2 2ab
Moiety formula   C54 H36 O10 Zn2 [+ solvent]       C54 H36 O10 Zn2
Sum formula      C54 H36 O10 Zn2 [+ solvent]       C54 H36 O10 Zn2
Mr               975.61                            975.57
Dx,g cm-3        0.379                             0.379
Z                4                                 4
Mu (mm-1)        0.314                             0.314
F000             2000.0                            2000.0
F000'            1982.23
h,k,lmax         24,61,19                          23,61,19
Nref             30239[ 16333]                     28610
Tmin,Tmax        0.960,0.969                       0.510,0.751
Tmin'            0.960

Correction method= # Reported T Limits: Tmin=0.510 Tmax=0.751
AbsCorr = MULTI-SCAN

Data completeness= 1.75/0.95        Theta(max)= 52.981

R(reflections)= 0.1288( 9273)                      wR2(reflections)=
                                                   0.3454( 28610)
S = 1.046                  Npar= 502
```

The following ALERTS were generated. Each ALERT has the format
test-name_ALERT_alert-type_alert-level.
Click on the hyperlinks for more details of the test.

Alert level B

PLAT026_ALERT_3_B Ratio Observed / Unique Reflections (too) Low .. 32% Check
PLAT242_ALERT_2_B Low 'MainMol' Ueq as Compared to Neighbors of Zn2 Check
PLAT420_ALERT_2_B D-H Bond Without Acceptor O9 --H9A . Please Check
PLAT420_ALERT_2_B D-H Bond Without Acceptor O9 --H9B . Please Check
PLAT420_ALERT_2_B D-H Bond Without Acceptor O10 --H10A . Please Check
PLAT420_ALERT_2_B D-H Bond Without Acceptor O10 --H10B . Please Check

Alert level C

RINTA01_ALERT_3_C The value of Rint is greater than 0.12
Rint given 0.151
STRVA01_ALERT_4_C Flack test results are ambiguous.
From the CIF: _refine_ls_abs_structure_Flack 0.420
From the CIF: _refine_ls_abs_structure_Flack_su 0.090
PLAT020_ALERT_3_C The Value of Rint is Greater Than 0.12 .... 0.151 Report
PLAT082_ALERT_2_C High R1 Value .... 0.13 Report
PLAT084_ALERT_3_C High wR2 Value (i.e. > 0.25) .... 0.35 Report
PLAT241_ALERT_2_C High 'MainMol' Ueq as Compared to Neighbors of C9 Check
PLAT241_ALERT_2_C High 'MainMol' Ueq as Compared to Neighbors of C12 Check
PLAT241_ALERT_2_C High 'MainMol' Ueq as Compared to Neighbors of C53 Check
PLAT242_ALERT_2_C Low 'MainMol' Ueq as Compared to Neighbors of Zn1 Check
PLAT250_ALERT_2_C Large U3/U1 Ratio for Average U(i,j) Tensor .... 2.1 Note
PLAT260_ALERT_2_C Large Average Ueq of Residue Including Zn1 0.107 Check
PLAT341_ALERT_3_C Low Bond Precision on C-C Bonds .... 0.01267 Ang.
PLAT369_ALERT_2_C Long C(sp2)-C(sp2) Bond C1 - C2 . 1.56 Ang.
PLAT369_ALERT_2_C Long C(sp2)-C(sp2) Bond C5 - C8 . 1.53 Ang.
PLAT369_ALERT_2_C Long C(sp2)-C(sp2) Bond C19 - C22 . 1.55 Ang.
PLAT369_ALERT_2_C Long C(sp2)-C(sp2) Bond C25 - C28 . 1.53 Ang.
PLAT369_ALERT_2_C Long C(sp2)-C(sp2) Bond C32 - C35 . 1.56 Ang.
PLAT369_ALERT_2_C Long C(sp2)-C(sp2) Bond C45 - C48 . 1.54 Ang.
PLAT905_ALERT_3_C Negative K value in the Analysis of Variance ... -20.048 Report
PLAT905_ALERT_3_C Negative K value in the Analysis of Variance ... -2.326 Report
PLAT911_ALERT_3_C Missing FCF Refl Between Thmin & STh/L= 0.595 266 Report
PLAT977_ALERT_2_C Check Negative Difference Density on H4 . -0.32 eA-3
PLAT977_ALERT_2_C Check Negative Difference Density on H10 . -0.38 eA-3
PLAT977_ALERT_2_C Check Negative Difference Density on H23 . -0.36 eA-3
PLAT977_ALERT_2_C Check Negative Difference Density on H24 . -0.34 eA-3
PLAT977_ALERT_2_C Check Negative Difference Density on H27 . -0.37 eA-3
PLAT977_ALERT_2_C Check Negative Difference Density on H34 . -0.41 eA-3
PLAT977_ALERT_2_C Check Negative Difference Density on H47 . -0.34 eA-3

Alert level G

ABSMU01_ALERT_1_G Calculation of _exptl_absorpt_correction_mu
not performed for this radiation type.
PLAT002_ALERT_2_G Number of Distance or Angle Restraints on AtSite 32 Note
PLAT003_ALERT_2_G Number of Uiso or Uij Restrained non-H Atoms ... 66 Report
PLAT004_ALERT_5_G Polymeric Structure Found with Maximum Dimension 2 Info
PLAT007_ALERT_5_G Number of Unrefined Donor-H Atoms .... 4 Report

```
PLAT033_ALERT_4_G Flack x Value Deviates > 3.0 * sigma from Zero .      0.420 Note
PLAT072_ALERT_2_G SHELXL First  Parameter in WGHT  Unusually Large       0.12 Report
PLAT172_ALERT_4_G The CIF-Embedded .res File Contains DFIX Records         10 Report
PLAT173_ALERT_4_G The CIF-Embedded .res File Contains DANG Records          4 Report
PLAT176_ALERT_4_G The CIF-Embedded .res File Contains SADI Records          2 Report
PLAT178_ALERT_4_G The CIF-Embedded .res File Contains SIMU Records          1 Report
PLAT186_ALERT_4_G The CIF-Embedded .res File Contains ISOR Records          1 Report
PLAT606_ALERT_4_G Solvent Accessible VOID(S) in Structure ........          ! Info
PLAT794_ALERT_5_G Tentative Bond Valency for Zn1       (II)      .       2.62 Info
PLAT794_ALERT_5_G Tentative Bond Valency for Zn2       (II)      .       1.86 Info
PLAT860_ALERT_3_G Number of Least-Squares Restraints ............         848 Note
PLAT910_ALERT_3_G Missing # of FCF Reflection(s) Below Theta(Min).          1 Note
PLAT913_ALERT_3_G Missing # of Very Strong Reflections in FCF ....          1 Note
PLAT933_ALERT_2_G Number of HKL-OMIT Records in Embedded .res File          4 Note
PLAT941_ALERT_3_G Average HKL Measurement Multiplicity ..........         3.8 Low
PLAT978_ALERT_2_G Number C-C Bonds with Positive Residual Density.          0 Info
```

---

 0 **ALERT level A** = Most likely a serious problem - resolve or explain
 6 **ALERT level B** = A potentially serious problem, consider carefully
28 **ALERT level C** = Check. Ensure it is not caused by an omission or oversight
21 **ALERT level G** = General information/check it is not something unexpected

 1 ALERT type 1 CIF construction/syntax error, inconsistent or missing data
30 ALERT type 2 Indicator that the structure model may be wrong or deficient
12 ALERT type 3 Indicator that the structure quality may be low
 8 ALERT type 4 Improvement, methodology, query or suggestion
 4 ALERT type 5 Informative message, check

It is advisable to attempt to resolve as many as possible of the alerts in all categories. Often the minor alerts point to easily fixed oversights, errors and omissions in your CIF or refinement strategy, so attention to these fine details can be worthwhile. In order to resolve some of the more serious problems it may be necessary to carry out additional measurements or structure refinements. However, the purpose of your study may justify the reported deviations and the more serious of these should normally be commented upon in the discussion or experimental section of a paper or in the "special_details" fields of the CIF. checkCIF was carefully designed to identify outliers and unusual parameters, but every test has its limitations and alerts that are not important in a particular case may appear. Conversely, the absence of alerts does not guarantee there are no aspects of the results needing attention. It is up to the individual to critically assess their own results and, if necessary, seek expert advice.

Publication of your CIF in IUCr journals

A basic structural check has been run on your CIF. These basic checks will be run on all CIFs submitted for publication in IUCr journals (*Acta Crystallographica*, *Journal of Applied Crystallography*, *Journal of Synchrotron Radiation*); however, if you intend to submit to *Acta Crystallographica Section C* or *E* or *IUCrData*, you should make sure that full publication checks are run on the final version of your CIF prior to submission.

Publication of your CIF in other journals

Please refer to the *Notes for Authors* of the relevant journal for any special instructions relating to CIF submission.

PLATON version of 12/09/2022; check.def file version of 09/08/2022

Datablock a1 - ellipsoid plot

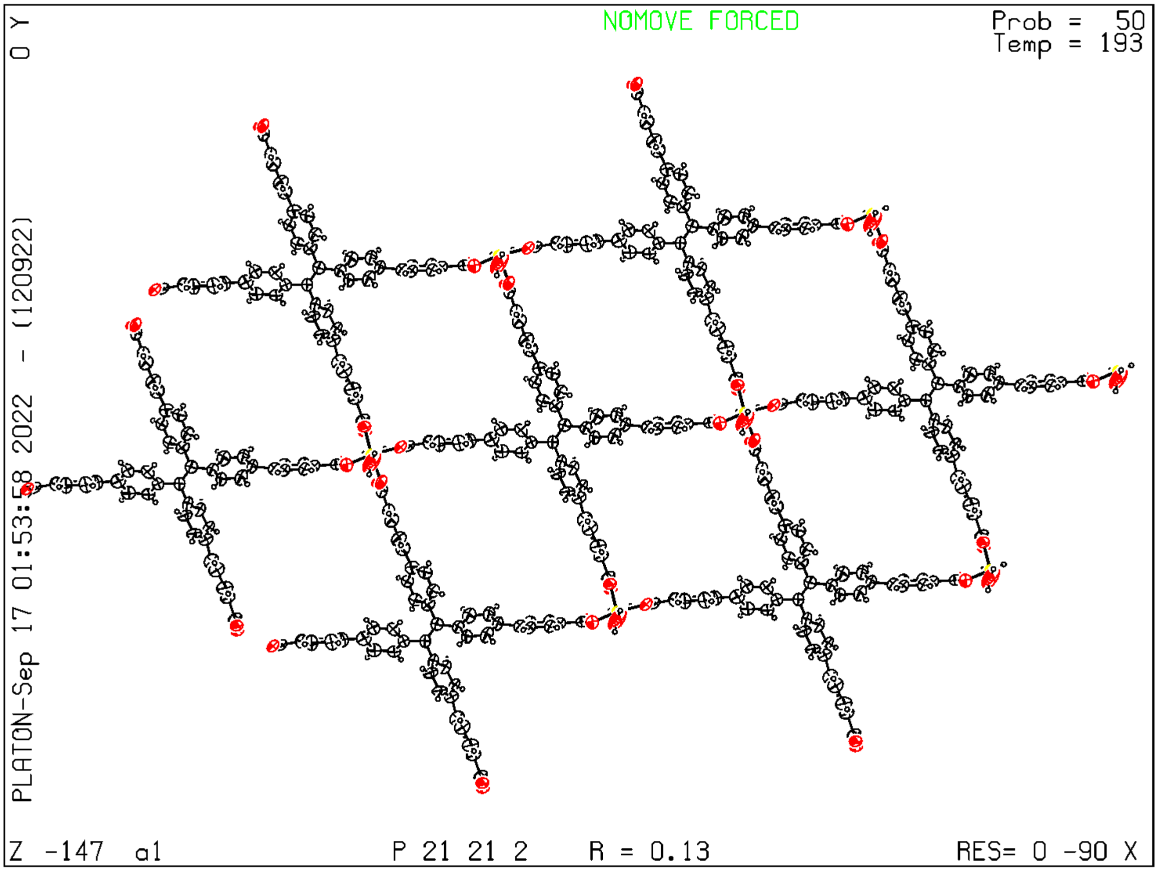

Supplement: Supplementary file 4 — Supplementary Data 1 [file 41467_2023_40594_MOESM4_ESM.zip › Supplementary Data 1/Model-MOF-2.pdf]
