# Supplementary material for: Pillararene incorporated metal–organic frameworks for supramolecular recognition and selective separation
Source: Nat Commun. 2023 Aug 15;14:4927. doi: 10.1038/s41467-023-40594-2 (PMC10427641; doi:10.1038/s41467-023-40594-2)

checkCIF/PLATON report

Structure factors have been supplied for datablock(s) a

THIS REPORT IS FOR GUIDANCE ONLY. IF USED AS PART OF A REVIEW PROCEDURE FOR PUBLICATION, IT SHOULD NOT REPLACE THE EXPERTISE OF AN EXPERIENCED CRYSTALLOGRAPHIC REFEREE.

No syntax errors found.   CIF dictionary   Interpreting this report

Datablock: a

Bond precision: C-C = 0.0078 A   Wavelength=1.34139

Cell:	a=20.313(2)	b=50.970(6)	c=16.226(2)
	alpha=90	beta=90	gamma=90
Temperature:	193 K		

	Calculated	Reported
Volume	16800(3)	16800(4)
Space group	P 21 21 2	P 21 21 2
Hall group	P 2 2ab	P 2 2ab
Moiety formula	C119 H92 N2 O16 Zn2 [+ solvent]	C119 H92 N2 O16 Zn2
Sum formula	C119 H92 N2 O16 Zn2 [+ solvent]	C119 H92 N2 O16 Zn2
Mr	1936.73	1936.68
Dx,g cm-3	0.766	0.766
Z	4	4
Mu (mm-1)	0.441	0.441
F000	4032.0	4032.0
F000’	4018.64	
h,k,lmax	25,63,20	25,62,20
Nref	34601[18630]	32229
Tmin,Tmax	0.944,0.957	0.565,0.751
Tmin’	0.944	

Correction method= # Reported T Limits: Tmin=0.565 Tmax=0.751
AbsCorr = MULTI-SCAN

Data completeness= 1.73/0.93   Theta(max)= 57.147

R(reflections)= 0.0540(19206)

wR2(reflections)= 0.1359(32229)

S = 0.911   Npar= 1288

The following ALERTS were generated. Each ALERT has the format
test-name_ALERT_alert-type_alert-level.
Click on the hyperlinks for more details of the test.

Alert level C

PLAT029_ALERT_3_C _diffrn_measured_fraction_theta_full value Low . 0.969 Why?
PLAT213_ALERT_2_C Atom C14 has ADP max/min Ratio ..... 3.3 prolat
PLAT213_ALERT_2_C Atom C118 has ADP max/min Ratio ..... 3.6 prolat
PLAT220_ALERT_2_C NonSolvent Resd 1 C Ueq(max)/Ueq(min) Range 5.6 Ratio
PLAT220_ALERT_2_C NonSolvent Resd 1 O Ueq(max)/Ueq(min) Range 3.5 Ratio
PLAT222_ALERT_3_C NonSolvent Resd 1 H Uiso(max)/Uiso(min) Range 7.1 Ratio
PLAT241_ALERT_2_C High 'MainMol' Ueq as Compared to Neighbors of O2 Check
PLAT241_ALERT_2_C High 'MainMol' Ueq as Compared to Neighbors of C18 Check
PLAT241_ALERT_2_C High 'MainMol' Ueq as Compared to Neighbors of C20 Check
PLAT241_ALERT_2_C High 'MainMol' Ueq as Compared to Neighbors of C62 Check
PLAT241_ALERT_2_C High 'MainMol' Ueq as Compared to Neighbors of C79 Check
PLAT241_ALERT_2_C High 'MainMol' Ueq as Compared to Neighbors of C80 Check
PLAT241_ALERT_2_C High 'MainMol' Ueq as Compared to Neighbors of C81 Check
PLAT242_ALERT_2_C Low 'MainMol' Ueq as Compared to Neighbors of Zn2 Check
PLAT242_ALERT_2_C Low 'MainMol' Ueq as Compared to Neighbors of N1 Check
PLAT242_ALERT_2_C Low 'MainMol' Ueq as Compared to Neighbors of C19 Check
PLAT242_ALERT_2_C Low 'MainMol' Ueq as Compared to Neighbors of C78 Check
PLAT334_ALERT_2_C Small <C-C> Benzene Dist. C60 -C65 . 1.37 Ang.
PLAT341_ALERT_3_C Low Bond Precision on C-C Bonds .... 0.00781 Ang.
PLAT362_ALERT_2_C Short C(sp3)-C(sp2) Bond C92 - C93 . 1.38 Ang.
PLAT369_ALERT_2_C Long C(sp2)-C(sp2) Bond C57 - C60 . 1.53 Ang.
PLAT369_ALERT_2_C Long C(sp2)-C(sp2) Bond C75 - C78 . 1.53 Ang.
PLAT412_ALERT_2_C Short Intra XH3 .. XHn H86 ..H91C . 1.85 Ang.
x,y,z = 1_555 Check
PLAT905_ALERT_3_C Negative K value in the Analysis of Variance ... -1.361 Report
PLAT911_ALERT_3_C Missing FCF Refl Between Thmin & STh/L= 0.600 510 Report

---

Alert level G

ABSMU01_ALERT_1_G Calculation of _exptl_absorpt_correction_mu
not performed for this radiation type.
PLAT002_ALERT_2_G Number of Distance or Angle Restraints on AtSite 24 Note
PLAT003_ALERT_2_G Number of Uiso or Uij Restrained non-H Atoms ... 62 Report
PLAT004_ALERT_5_G Polymeric Structure Found with Maximum Dimension 3 Info
PLAT033_ALERT_4_G Flack x Value Deviates > 3.0 * sigma from Zero . 0.280 Note
PLAT173_ALERT_4_G The CIF-Embedded .res File Contains DANG Records 1 Report
PLAT176_ALERT_4_G The CIF-Embedded .res File Contains SADI Records 6 Report
PLAT178_ALERT_4_G The CIF-Embedded .res File Contains SIMU Records 1 Report
PLAT301_ALERT_3_G Main Residue Disorder ....(Resd 1) 6% Note
PLAT335_ALERT_2_G Check Large C6 Ring C-C Range C35 -C40 0.18 Ang.
PLAT335_ALERT_2_G Check Large C6 Ring C-C Range C66 -C71 0.15 Ang.
PLAT335_ALERT_2_G Check Large C6 Ring C-C Range C84 -C89 0.16 Ang.
PLAT398_ALERT_2_G Deviating C-O-C Angle From 120 for O9 . 102.8 Degree
PLAT412_ALERT_2_G Short Intra XH3 .. XHn H10D ..H1AB . 2.10 Ang.
x,y,z = 1_555 Check

```
PLAT412_ALERT_2_G Short Intra XH3 .. XHn     H10D     ..H1AC     .       2.11 Ang.
                                                  x,y,z  =     1_555 Check
PLAT606_ALERT_4_G Solvent Accessible VOID(S) in Structure .......          ! Info
PLAT720_ALERT_4_G Number of Unusual/Non-Standard Labels .........          6 Note
PLAT794_ALERT_5_G Tentative Bond Valency for Zn1       (II)      .       2.08 Info
PLAT860_ALERT_3_G Number of Least-Squares Restraints ............        420 Note
PLAT910_ALERT_3_G Missing # of FCF Reflection(s) Below Theta(Min).          4 Note
PLAT912_ALERT_4_G Missing # of FCF Reflections Above STh/L=  0.600        268 Note
PLAT913_ALERT_3_G Missing # of Very Strong Reflections in FCF ....          3 Note
PLAT916_ALERT_2_G Hooft y and Flack x Parameter Values Differ by .       0.21 Check
PLAT933_ALERT_2_G Number of HKL-OMIT Records in Embedded .res File          2 Note
PLAT978_ALERT_2_G Number C-C Bonds with Positive Residual Density.          0 Info
```

```
   0 ALERT level A = Most likely a serious problem - resolve or explain
   0 ALERT level B = A potentially serious problem, consider carefully
  25 ALERT level C = Check. Ensure it is not caused by an omission or oversight
  25 ALERT level G = General information/check it is not something unexpected

   1 ALERT type 1 CIF construction/syntax error, inconsistent or missing data
  31 ALERT type 2 Indicator that the structure model may be wrong or deficient
   9 ALERT type 3 Indicator that the structure quality may be low
   7 ALERT type 4 Improvement, methodology, query or suggestion
   2 ALERT type 5 Informative message, check
```

It is advisable to attempt to resolve as many as possible of the alerts in all categories. Often the minor alerts point to easily fixed oversights, errors and omissions in your CIF or refinement strategy, so attention to these fine details can be worthwhile. In order to resolve some of the more serious problems it may be necessary to carry out additional measurements or structure refinements. However, the purpose of your study may justify the reported deviations and the more serious of these should normally be commented upon in the discussion or experimental section of a paper or in the "special_details" fields of the CIF. checkCIF was carefully designed to identify outliers and unusual parameters, but every test has its limitations and alerts that are not important in a particular case may appear. Conversely, the absence of alerts does not guarantee there are no aspects of the results needing attention. It is up to the individual to critically assess their own results and, if necessary, seek expert advice.

Publication of your CIF in IUCr journals

A basic structural check has been run on your CIF. These basic checks will be run on all CIFs submitted for publication in IUCr journals (*Acta Crystallographica*, *Journal of Applied Crystallography*, *Journal of Synchrotron Radiation*); however, if you intend to submit to *Acta Crystallographica Section C* or *E* or *IUCrData*, you should make sure that full publication checks are run on the final version of your CIF prior to submission.

Publication of your CIF in other journals

Please refer to the *Notes for Authors* of the relevant journal for any special instructions relating to CIF submission.

PLATON version of 12/09/2022; check.def file version of 09/08/2022

Datablock a - ellipsoid plot

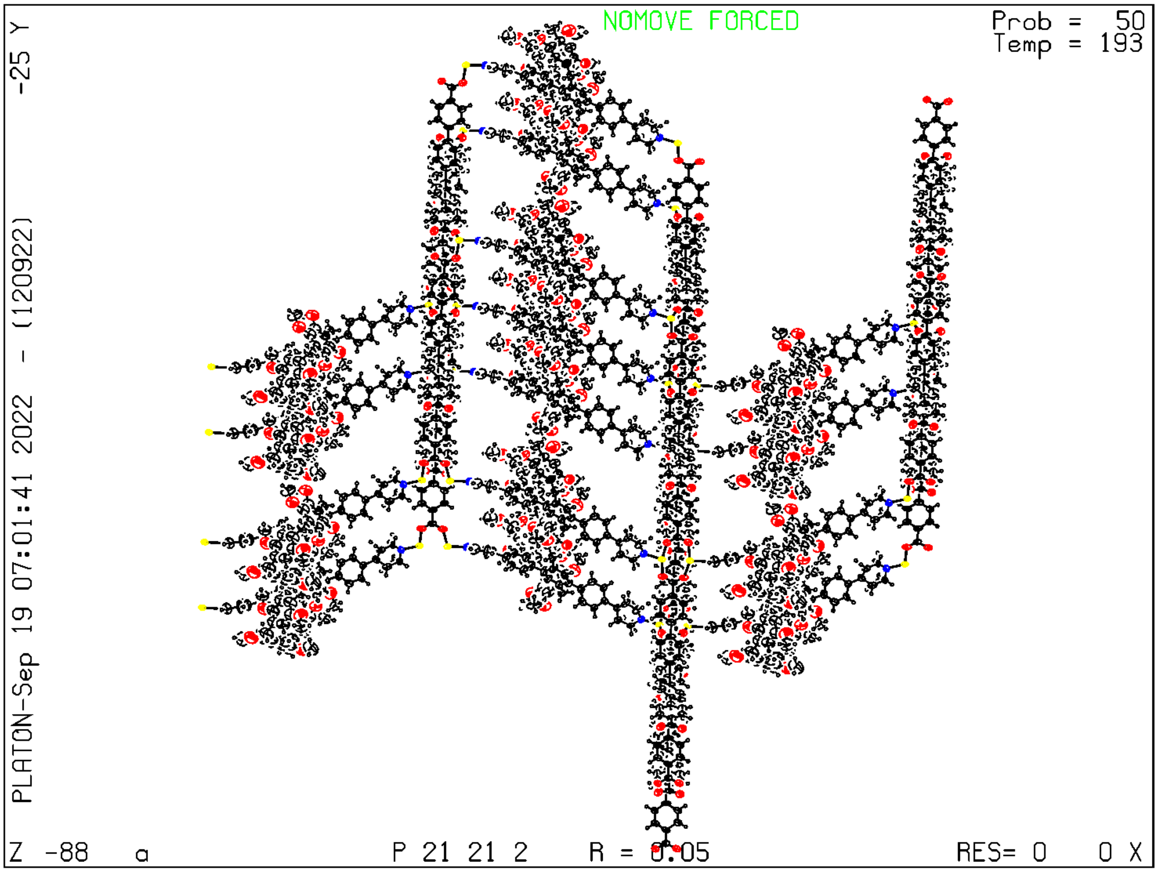

Supplement: Supplementary file 4 — Supplementary Data 1 [file 41467_2023_40594_MOESM4_ESM.zip › Supplementary Data 1/pS-MeP5-MOF-2.pdf]
