# Supplementary material for: Pillararene incorporated metal–organic frameworks for supramolecular recognition and selective separation
Source: Nat Commun. 2023 Aug 15;14:4927. doi: 10.1038/s41467-023-40594-2 (PMC10427641; doi:10.1038/s41467-023-40594-2)

checkCIF/PLATON report

Structure factors have been supplied for datablock(s) 221031li_lidz263361_tol_0m

THIS REPORT IS FOR GUIDANCE ONLY. IF USED AS PART OF A REVIEW PROCEDURE FOR PUBLICATION, IT SHOULD NOT REPLACE THE EXPERTISE OF AN EXPERIENCED CRYSTALLOGRAPHIC REFEREE.

No syntax errors found. CIF dictionary Interpreting this report

Datablock: 221031li_lidz263361_tol_0m

Bond precision: C-C = 0.0117 A Wavelength=0.71073

Cell:	a=15.652(2)	b=20.687(3)	c=23.936(3)
	alpha=67.358(4)	beta=80.274(4)	gamma=89.931(4)
Temperature:	193 K		

	Calculated	Reported
Volume	7032.8(16)	7032.3(16)
Space group	P -1	P -1
Hall group	-P 1	-P 1
Moiety formula	C119 H92 N2 O16 Zn2, 1.5(C3.50 H4) [+ solvent]	C119 H92 N2 O16 Zn2, C7 H8
Sum formula	C126 H100 N2 O16 Zn2 [+ solvent]	C126 H100 N2 O16 Zn2
Mr	2028.87	2028.81
Dx,g cm-3	0.958	0.958
Z	2	2
Mu (mm-1)	0.392	0.392
F000	2116.0	2116.0
F000'	2118.03	
h,k,lmax	18,24,28	18,24,28
Nref	25744	25536
Tmin,Tmax	0.950,0.962	0.575,0.745
Tmin'	0.950	

Correction method= # Reported T Limits: Tmin=0.575 Tmax=0.745
AbsCorr = MULTI-SCAN

Data completeness= 0.992 Theta(max)= 25.350

R(reflections)= 0.0983(13017)

wR2(reflections)= 0.3162(25536)

S = 0.982 Npar= 1365

The following ALERTS were generated. Each ALERT has the format
test-name_ALERT_alert-type_alert-level.
Click on the hyperlinks for more details of the test.

Alert level B

```
PLAT910_ALERT_3_B Missing # of FCF Reflection(s) Below Theta(Min).        12 Note
```

Alert level C

```
PLAT084_ALERT_3_C High wR2 Value (i.e. > 0.25) ...................      0.32 Report
PLAT094_ALERT_2_C Ratio of Maximum / Minimum Residual Density ....      2.02 Report
PLAT220_ALERT_2_C NonSolvent   Resd 1  C   Ueq(max)/Ueq(min) Range       5.1 Ratio
PLAT222_ALERT_3_C NonSolvent Resd 1  H   Uiso(max)/Uiso(min) Range       5.9 Ratio
PLAT241_ALERT_2_C High   'MainMol' Ueq as Compared to Neighbors of       O12 Check
PLAT241_ALERT_2_C High   'MainMol' Ueq as Compared to Neighbors of       C55 Check
PLAT241_ALERT_2_C High   'MainMol' Ueq as Compared to Neighbors of       C58 Check
PLAT241_ALERT_2_C High   'MainMol' Ueq as Compared to Neighbors of       C59 Check
PLAT241_ALERT_2_C High   'MainMol' Ueq as Compared to Neighbors of       C64 Check
PLAT242_ALERT_2_C Low    'MainMol' Ueq as Compared to Neighbors of       O10 Check
PLAT242_ALERT_2_C Low    'MainMol' Ueq as Compared to Neighbors of       O11 Check
PLAT242_ALERT_2_C Low    'MainMol' Ueq as Compared to Neighbors of       O16 Check
PLAT242_ALERT_2_C Low    'MainMol' Ueq as Compared to Neighbors of        N1 Check
PLAT242_ALERT_2_C Low    'MainMol' Ueq as Compared to Neighbors of       C63 Check
PLAT250_ALERT_2_C Large U3/U1 Ratio for Average U(i,j) Tensor ....       3.2 Note
PLAT250_ALERT_2_C Large U3/U1 Ratio for Average U(i,j) Tensor ....       2.2 Note
PLAT260_ALERT_2_C Large Average Ueq of Residue Including      C03P     0.147 Check
PLAT260_ALERT_2_C Large Average Ueq of Residue Including      C03U     0.105 Check
PLAT341_ALERT_3_C Low Bond Precision on  C-C Bonds ...............    0.0117 Ang.
PLAT412_ALERT_2_C Short Intra XH3 .. XHn     H87      ..H88C     .      1.87 Ang.
                                                       x,y,z  =      1_555 Check
PLAT905_ALERT_3_C Negative K value in the Analysis of Variance ...    -4.872 Report
PLAT911_ALERT_3_C Missing FCF Refl Between Thmin & STh/L=    0.600       193 Report
PLAT918_ALERT_3_C Reflection(s) with I(obs) much Smaller I(calc) .         4 Check
PLAT973_ALERT_2_C Check Calcd Positive Resid. Density on       Zn1      1.30 eA-3
PLAT977_ALERT_2_C Check Negative Difference Density on H93       .     -0.33 eA-3
```

Alert level G

```
PLAT002_ALERT_2_G Number of Distance or Angle Restraints on AtSite         4 Note
PLAT003_ALERT_2_G Number of Uiso or Uij Restrained non-H Atoms ...        71 Report
PLAT004_ALERT_5_G Polymeric Structure Found with Maximum Dimension         3 Info
PLAT042_ALERT_1_G Calc. and Reported MoietyFormula Strings  Differ    Please Check
PLAT072_ALERT_2_G SHELXL First  Parameter in WGHT  Unusually Large      0.20 Report
PLAT154_ALERT_1_G The s.u.'s on the Cell Angles are Equal ..(Note)     0.004 Degree
PLAT172_ALERT_4_G The CIF-Embedded .res File Contains DFIX Records         2 Report
PLAT178_ALERT_4_G The CIF-Embedded .res File Contains SIMU Records         4 Report
PLAT300_ALERT_4_G Atom Site Occupancy of C03P       Constrained at       0.5 Check
PLAT300_ALERT_4_G Atom Site Occupancy of C03W       Constrained at       0.5 Check
PLAT300_ALERT_4_G Atom Site Occupancy of C121       Constrained at       0.5 Check
PLAT300_ALERT_4_G Atom Site Occupancy of C123       Constrained at       0.5 Check
```

```
PLAT300_ALERT_4_G Atom Site Occupancy of C125      Constrained at        0.5 Check
PLAT300_ALERT_4_G Atom Site Occupancy of C126      Constrained at        0.5 Check
PLAT300_ALERT_4_G Atom Site Occupancy of C128      Constrained at        0.5 Check
PLAT300_ALERT_4_G Atom Site Occupancy of H03A      Constrained at        0.5 Check
PLAT300_ALERT_4_G Atom Site Occupancy of H03B      Constrained at        0.5 Check
PLAT300_ALERT_4_G Atom Site Occupancy of H03C      Constrained at        0.5 Check
PLAT300_ALERT_4_G Atom Site Occupancy of H121      Constrained at        0.5 Check
PLAT300_ALERT_4_G Atom Site Occupancy of H123      Constrained at        0.5 Check
PLAT300_ALERT_4_G Atom Site Occupancy of H125      Constrained at        0.5 Check
PLAT300_ALERT_4_G Atom Site Occupancy of H126      Constrained at        0.5 Check
PLAT300_ALERT_4_G Atom Site Occupancy of H128      Constrained at        0.5 Check
PLAT300_ALERT_4_G Atom Site Occupancy of C03U      Constrained at        0.5 Check
PLAT300_ALERT_4_G Atom Site Occupancy of C03V      Constrained at        0.5 Check
PLAT300_ALERT_4_G Atom Site Occupancy of C03Z      Constrained at        0.5 Check
PLAT300_ALERT_4_G Atom Site Occupancy of C040      Constrained at        0.5 Check
PLAT300_ALERT_4_G Atom Site Occupancy of C120      Constrained at        0.5 Check
PLAT300_ALERT_4_G Atom Site Occupancy of C122      Constrained at        0.5 Check
PLAT300_ALERT_4_G Atom Site Occupancy of C124      Constrained at        0.5 Check
PLAT300_ALERT_4_G Atom Site Occupancy of H03U      Constrained at        0.5 Check
PLAT300_ALERT_4_G Atom Site Occupancy of H03V      Constrained at        0.5 Check
PLAT300_ALERT_4_G Atom Site Occupancy of H03Z      Constrained at        0.5 Check
PLAT300_ALERT_4_G Atom Site Occupancy of H040      Constrained at        0.5 Check
PLAT300_ALERT_4_G Atom Site Occupancy of H12A      Constrained at        0.5 Check
PLAT300_ALERT_4_G Atom Site Occupancy of H12B      Constrained at        0.5 Check
PLAT300_ALERT_4_G Atom Site Occupancy of H12C      Constrained at        0.5 Check
PLAT300_ALERT_4_G Atom Site Occupancy of H122      Constrained at        0.5 Check
PLAT302_ALERT_4_G Anion/Solvent/Minor-Residue Disorder (Resd  2  )       100% Note
PLAT302_ALERT_4_G Anion/Solvent/Minor-Residue Disorder (Resd  3  )       100% Note
PLAT606_ALERT_4_G Solvent Accessible VOID(S) in Structure ........          ! Info
PLAT720_ALERT_4_G Number of Unusual/Non-Standard Labels ..........         13 Note
PLAT794_ALERT_5_G Tentative Bond Valency for Zn1       (II)      .       2.03 Info
PLAT794_ALERT_5_G Tentative Bond Valency for Zn2       (II)      .       1.99 Info
PLAT804_ALERT_5_G Number of ARU-Code Packing Problem(s) in PLATON          24 Info
PLAT860_ALERT_3_G Number of Least-Squares Restraints ............         458 Note
PLAT912_ALERT_4_G Missing # of FCF Reflections Above STh/L=  0.600          3 Note
PLAT913_ALERT_3_G Missing # of Very Strong Reflections in FCF ....          2 Note
PLAT933_ALERT_2_G Number of HKL-OMIT Records in Embedded .res File         12 Note
PLAT941_ALERT_3_G Average HKL Measurement Multiplicity ...........        1.9 Low
PLAT978_ALERT_2_G Number C-C Bonds with Positive Residual Density.          1 Info
PLAT992_ALERT_5_G Repd & Actual _reflns_number_gt Values Differ by          2 Check
```

```
   0 ALERT level A = Most likely a serious problem - resolve or explain
   1 ALERT level B = A potentially serious problem, consider carefully
  25 ALERT level C = Check. Ensure it is not caused by an omission or oversight
  52 ALERT level G = General information/check it is not something unexpected

   2 ALERT type 1 CIF construction/syntax error, inconsistent or missing data
  24 ALERT type 2 Indicator that the structure model may be wrong or deficient
  10 ALERT type 3 Indicator that the structure quality may be low
  37 ALERT type 4 Improvement, methodology, query or suggestion
   5 ALERT type 5 Informative message, check
```

It is advisable to attempt to resolve as many as possible of the alerts in all categories. Often the minor alerts point to easily fixed oversights, errors and omissions in your CIF or refinement strategy, so attention to these fine details can be worthwhile. In order to resolve some of the more serious problems it may be necessary to carry out additional measurements or structure refinements. However, the purpose of your study may justify the reported deviations and the more serious of these should normally be commented upon in the discussion or experimental section of a paper or in the "special_details" fields of the CIF. checkCIF was carefully designed to identify outliers and unusual parameters, but every test has its limitations and alerts that are not important in a particular case may appear. Conversely, the absence of alerts does not guarantee there are no aspects of the results needing attention. It is up to the individual to critically assess their own results and, if necessary, seek expert advice.

Publication of your CIF in IUCr journals

A basic structural check has been run on your CIF. These basic checks will be run on all CIFs submitted for publication in IUCr journals (*Acta Crystallographica*, *Journal of Applied Crystallography*, *Journal of Synchrotron Radiation*); however, if you intend to submit to *Acta Crystallographica Section C* or *E* or *IUCrData*, you should make sure that full publication checks are run on the final version of your CIF prior to submission.

Publication of your CIF in other journals

Please refer to the *Notes for Authors* of the relevant journal for any special instructions relating to CIF submission.

PLATON version of 12/09/2022; check.def file version of 09/08/2022

Datablock 221031li_lidz263361_tol_0m - ellipsoid plot

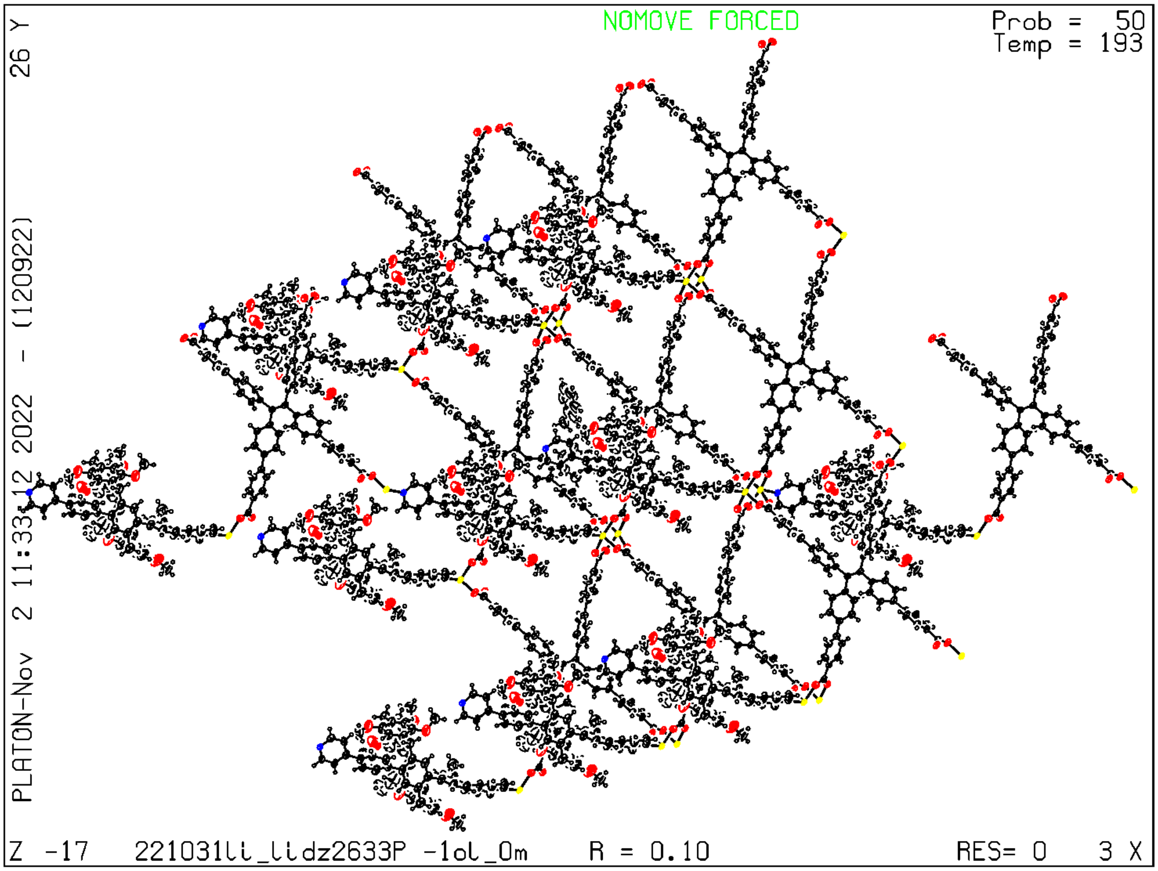

Supplement: Supplementary file 4 — Supplementary Data 1 [file 41467_2023_40594_MOESM4_ESM.zip › Supplementary Data 1/Tol@MeP5-MOF-2.pdf]
