# Supplementary material for: Pillararene incorporated metal–organic frameworks for supramolecular recognition and selective separation
Source: Nat Commun. 2023 Aug 15;14:4927. doi: 10.1038/s41467-023-40594-2 (PMC10427641; doi:10.1038/s41467-023-40594-2)

checkCIF/PLATON report

Structure factors have been supplied for datablock(s) a

THIS REPORT IS FOR GUIDANCE ONLY. IF USED AS PART OF A REVIEW PROCEDURE FOR PUBLICATION, IT SHOULD NOT REPLACE THE EXPERTISE OF AN EXPERIENCED CRYSTALLOGRAPHIC REFEREE.

No syntax errors found. CIF dictionary Interpreting this report

Datablock: a

```
Bond precision:   C-C = 0.0104 A          Wavelength=1.34139

Cell:              a=20.4547(12)      b=50.956(3)      c=16.1010(9)
                   alpha=90           beta=90          gamma=90
Temperature:       193 K
```

	Calculated	Reported
Volume	16781.9(17)	16782.0(17)
Space group	P 21 21 2	P 21 21 2
Hall group	P 2 2ab	P 2 2ab
Moiety formula	C119 H92 N2 O16 Zn2 [+ solvent]	C119 H92 N2 O16 Zn2
Sum formula	C119 H92 N2 O16 Zn2 [+ solvent]	C119 H92 N2 O16 Zn2
Mr	1936.73	1936.68
Dx,g cm-3	0.767	0.767
Z	4	4
Mu (mm-1)	0.442	0.442
F000	4032.0	4032.0
F000'	4018.64	
h,k,lmax	26,66,20	26,65,20
Nref	38210[20524]	34787
Tmin,Tmax	0.944,0.957	0.545,0.752
Tmin'	0.944	

```
Correction method= # Reported T Limits: Tmin=0.545 Tmax=0.752
AbsCorr = MULTI-SCAN

Data completeness= 1.69/0.91        Theta(max)= 60.324
```

R(reflections)= 0.0681(21355) wR2(reflections)= 0.2038(34787)

S = 0.999 Npar= 1198

The following ALERTS were generated. Each ALERT has the format
test-name_ALERT_alert-type_alert-level.
Click on the hyperlinks for more details of the test.

Alert level B

```
PLAT220_ALERT_2_B NonSolvent   Resd 1   C   Ueq(max)/Ueq(min) Range        9.8 Ratio
```

Author Response: This Level B alert arises because the macrocyclic moiety shows more motion even though the data were collected at 193 K.

```
PLAT220_ALERT_2_B NonSolvent   Resd 1   O   Ueq(max)/Ueq(min) Range        6.6 Ratio
```

Author Response: This Level B alert arises because the macrocyclic moiety shows more motion even though the data were collected at 193 K.

Alert level C

```
PLAT213_ALERT_2_C Atom C31             has ADP max/min Ratio .....        3.7 prolat
PLAT222_ALERT_3_C NonSolvent Resd 1  H   Uiso(max)/Uiso(min) Range       10.0 Ratio
PLAT241_ALERT_2_C High   'MainMol' Ueq as Compared to Neighbors of         C6 Check
PLAT241_ALERT_2_C High   'MainMol' Ueq as Compared to Neighbors of        C13 Check
PLAT241_ALERT_2_C High   'MainMol' Ueq as Compared to Neighbors of        C47 Check
PLAT241_ALERT_2_C High   'MainMol' Ueq as Compared to Neighbors of        C49 Check
PLAT241_ALERT_2_C High   'MainMol' Ueq as Compared to Neighbors of        C55 Check
PLAT241_ALERT_2_C High   'MainMol' Ueq as Compared to Neighbors of        C59 Check
PLAT242_ALERT_2_C Low    'MainMol' Ueq as Compared to Neighbors of        Zn1 Check
PLAT242_ALERT_2_C Low    'MainMol' Ueq as Compared to Neighbors of        O14 Check
PLAT242_ALERT_2_C Low    'MainMol' Ueq as Compared to Neighbors of         N1 Check
PLAT242_ALERT_2_C Low    'MainMol' Ueq as Compared to Neighbors of         C8 Check
PLAT242_ALERT_2_C Low    'MainMol' Ueq as Compared to Neighbors of        C63 Check
PLAT242_ALERT_2_C Low    'MainMol' Ueq as Compared to Neighbors of       C109 Check
PLAT260_ALERT_2_C Large Average Ueq of Residue Including       Zn1      0.147 Check
PLAT341_ALERT_3_C Low Bond Precision on  C-C Bonds ..............    0.01036 Ang.
PLAT369_ALERT_2_C Long   C(sp2)-C(sp2) Bond  C57      - C60      .       1.54 Ang.
PLAT369_ALERT_2_C Long   C(sp2)-C(sp2) Bond  C63      - C66      .       1.53 Ang.
PLAT412_ALERT_2_C Short Intra XH3 .. XHn     H83      ..H89B     .       1.87 Ang.
                                             x,y,z  =      1_555 Check
PLAT733_ALERT_1_C Torsion Calc  -179.8(10), Rep  179.90(18) ......       5.56 s.u.-R
              C95 -C94 -C93 -O14   1_555  1_555  1_555  1_555      #  320 Check
PLAT905_ALERT_3_C Negative K value in the Analysis of Variance ...     -2.442 Report
PLAT911_ALERT_3_C Missing FCF Refl Between Thmin & STh/L=    0.600         28 Report
```

Alert level G

```
ABSMU01_ALERT_1_G  Calculation of _exptl_absorpt_correction_mu
```

```
                not performed for this radiation type.
PLAT002_ALERT_2_G Number of Distance or Angle Restraints on AtSite         46 Note
PLAT003_ALERT_2_G Number of Uiso or Uij Restrained non-H Atoms ...         77 Report
PLAT004_ALERT_5_G Polymeric Structure Found with Maximum Dimension          3 Info
PLAT033_ALERT_4_G Flack x Value Deviates > 3.0 * sigma from Zero .      0.260 Note
PLAT072_ALERT_2_G SHELXL First  Parameter in WGHT  Unusually Large       0.12 Report
PLAT172_ALERT_4_G The CIF-Embedded .res File Contains DFIX Records          5 Report
PLAT173_ALERT_4_G The CIF-Embedded .res File Contains DANG Records          8 Report
PLAT174_ALERT_4_G The CIF-Embedded .res File Contains FLAT Records          1 Report
PLAT176_ALERT_4_G The CIF-Embedded .res File Contains SADI Records          2 Report
PLAT178_ALERT_4_G The CIF-Embedded .res File Contains SIMU Records          5 Report
PLAT301_ALERT_3_G Main Residue  Disorder .............(Resd  1  )          3% Note
PLAT335_ALERT_2_G Check Large C6 Ring C-C Range C15      -C20            0.17 Ang.
PLAT335_ALERT_2_G Check Large C6 Ring C-C Range C73      -C78            0.16 Ang.
PLAT343_ALERT_2_G Unusual sp3      Angle Range in Main Residue for        C99 Check
PLAT410_ALERT_2_G Short Intra H...H Contact  H111     ..H116     .       2.14 Ang.
                                                      x,y,z  =      1_555 Check
PLAT432_ALERT_2_G Short Inter X...Y Contact  O2       ..C117     .       3.01 Ang.
                                             1/2-x,1/2+y,-z  =      3_555 Check
PLAT432_ALERT_2_G Short Inter X...Y Contact  O7       ..C1C      .       2.79 Ang.
                                            1/2-x,1/2+y,1-z  =      3_556 Check
PLAT432_ALERT_2_G Short Inter X...Y Contact  O7       ..C117     .       2.98 Ang.
                                            1/2-x,1/2+y,1-z  =      3_556 Check
PLAT606_ALERT_4_G Solvent Accessible VOID(S) in Structure ........          ! Info
PLAT794_ALERT_5_G Tentative Bond Valency for Zn1       (II)      .       2.12 Info
PLAT860_ALERT_3_G Number of Least-Squares Restraints ............         551 Note
PLAT912_ALERT_4_G Missing # of FCF Reflections Above STh/L=  0.600        451 Note
PLAT913_ALERT_3_G Missing # of Very Strong Reflections in FCF ....          2 Note
PLAT916_ALERT_2_G Hooft y and Flack x Parameter Values Differ by .       0.16 Check
PLAT933_ALERT_2_G Number of HKL-OMIT Records in Embedded .res File          6 Note
PLAT978_ALERT_2_G Number C-C Bonds with Positive Residual Density.          0 Info
PLAT992_ALERT_5_G Repd & Actual _reflns_number_gt Values Differ by          6 Check
```

---

0 **ALERT level A** = Most likely a serious problem - resolve or explain
2 **ALERT level B** = A potentially serious problem, consider carefully
22 **ALERT level C** = Check. Ensure it is not caused by an omission or oversight
28 **ALERT level G** = General information/check it is not something unexpected

2 ALERT type 1 CIF construction/syntax error, inconsistent or missing data
32 ALERT type 2 Indicator that the structure model may be wrong or deficient
7 ALERT type 3 Indicator that the structure quality may be low
8 ALERT type 4 Improvement, methodology, query or suggestion
3 ALERT type 5 Informative message, check

It is advisable to attempt to resolve as many as possible of the alerts in all categories. Often the minor alerts point to easily fixed oversights, errors and omissions in your CIF or refinement strategy, so attention to these fine details can be worthwhile. In order to resolve some of the more serious problems it may be necessary to carry out additional measurements or structure refinements. However, the purpose of your study may justify the reported deviations and the more serious of these should normally be commented upon in the discussion or experimental section of a paper or in the "special_details" fields of the CIF. checkCIF was carefully designed to identify outliers and unusual parameters, but every test has its limitations and alerts that are not important in a particular case may appear. Conversely, the absence of alerts does not guarantee there are no aspects of the results needing attention. It is up to the individual to critically assess their own results and, if necessary, seek expert advice.

Publication of your CIF in IUCr journals

A basic structural check has been run on your CIF. These basic checks will be run on all CIFs submitted for publication in IUCr journals (*Acta Crystallographica*, *Journal of Applied Crystallography*, *Journal of Synchrotron Radiation*); however, if you intend to submit to *Acta Crystallographica Section C* or *E* or *IUCrData*, you should make sure that full publication checks are run on the final version of your CIF prior to submission.

Publication of your CIF in other journals

Please refer to the *Notes for Authors* of the relevant journal for any special instructions relating to CIF submission.

PLATON version of 12/09/2022; check.def file version of 09/08/2022

Datablock a - ellipsoid plot

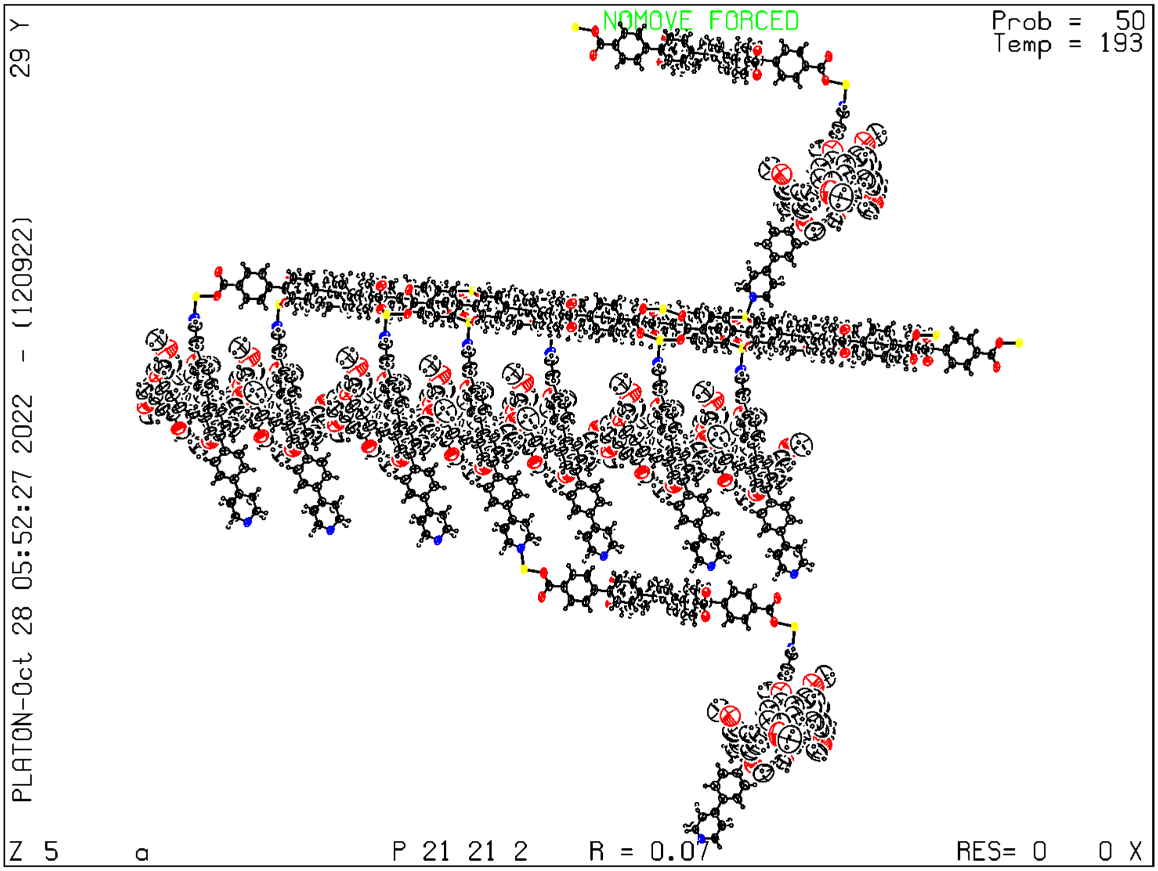

Supplement: Supplementary file 4 — Supplementary Data 1 [file pR-MeP5-MOF-2.pdf › Supplementary Data 1/pR-MeP5-MOF-2.pdf]
